# 3D Scanning of the Forearm for Orthosis and HMI Applications

**DOI:** 10.3389/frobt.2021.576783

**Published:** 2021-04-14

**Authors:** Joel C. Perry, Jacob R. Brower, Robert H. R. Carne, Melissa A. Bogert

**Affiliations:** Department of Mechanical Engineering, University of Idaho, Moscow, ID, United States

**Keywords:** 3D arm scanning, standardized orthosis design, physical human–machine interface, ellipse-fit forearm model, 3D point cloud, exoskeleton robotic interface

## Abstract

The rise of rehabilitation robotics has ignited a global investigation into the human machine interface (HMI) between device and user. Previous research on wearable robotics has primarily focused on robotic kinematics and controls but rarely on the actual design of the physical HMI (pHMI). This paper presents a data-driven statistical forearm surface model for designing a forearm orthosis in exoskeleton applications. The forearms of 6 subjects were 3D scanned in a custom-built jig to capture data in extreme pronation and supination poses, creating 3D point clouds of the forearm surface. Resulting data was characterized into a series of ellipses from 20 to 100% of the forearm length. Key ellipse parameters in the model include: normalized major and minor axis length, normalized center point location, tilt angle, and circularity ratio. Single-subject (SS) ellipse parameters were normalized with respect to forearm radiale-stylion (RS) length and circumference and then averaged over the 6 subjects. Averaged parameter profiles were fit with 3rd-order polynomials to create combined-subjects (CS) elliptical models of the forearm. CS models were created in the jig as-is (CS1) and after alignment to ellipse centers at 20 and 100% of the forearm length (CS2). Normalized curve fits of ellipse major and minor axes in model CS2 achieve *R*^2^ values ranging from 0.898 to 0.980 indicating a high degree of correlation between cross-sectional size and position along the forearm. Most other parameters showed poor correlation with forearm position (0.005 < *R*^2^ < 0.391) with the exception of tilt angle in pronation (0.877) and circularity in supination (0.657). Normalized RMSE of the CS2 ellipse-fit model ranged from 0.21 to 0.64% of forearm circumference and 0.22 to 0.46% of forearm length. The average and peak surface deviation between the scaled CS2 model and individual scans along the forearm varied from 0.56 to 2.86 mm (subject averages) and 3.86 to 7.16 (subject maximums), with the peak deviation occurring between 45 and 50% RS length. The developed equations allow reconstruction of a scalable 3D model that can be sized based on two user measures, RS length and forearm circumference, or based on generic arm measurements taken from existing anthropometric databases.

## Introduction

The prevalence of robotic devices in upper extremity stroke rehabilitation has risen significantly in the last decade. Devices include end-effector designs where a patient is connected to a robot at the hand and/or forearm, and exoskeleton designs where the robot may connect to the user at multiple points along the arm and hand. Exoskeletons mimic the length and structure of human limb segments in order to move synchronously with the segments during tasks. This requires an intimate human-to-robot connection, also known as a physical human–machine interface (pHMI), in order to distribute loading safely to adjacent biological tissues.

The main goal of a pHMI is to ensure synchronous movementbetween the user and robot through a comfortable and safe connection. Misalignment between a user and robot are potential comfort and safety issues that can increase the risk of injury (Amigo et al., 2011; Gopura et al., 2016) and lower system performance. Furthermore, misalignment from an offset or improperly sized pHMI can induce high concentrations of load on the skin, resulting in localized oxygen deprivation or cell death. In addition to safety concerns, prolonged discomfort from the physical connection to a robotic device can reduce motivation to train, and impair range of motion as well as robot tracking accuracy, all of which can degrade the performance and effectiveness of the device.

Despite the importance of pHMI design on device function, the methods and specifications associated with developing pHMI components have been largely ignored in published literature. An extensive review reveals that details on the pHMI tend to fall into one of three categories: (a) authors refer to an orthosis but the design is either not described or only briefly described in terms of appearance (Perry et al., 2007; Jarrassé et al., 2008; Klein et al., 2008; Pylatiuk et al., 2009; Gmerek, 2012; Ohnishi et al., 2013; Rohm et al., 2013; Vaca Benitez et al., 2013; Herrnstadt and Menon, 2016; Sangha et al., 2016); (b) authors describe a generic orthosis design with added compliance to mitigate forces due to misalignment or dissimilarities in anthropometric shape, in which case the design elements focus on kinematics (Jarrassé and Morel, 2012); or (c) authors explain human interface details but do not report on the shape of their orthosis design (Jackson et al., 2007; Rocon et al., 2007; Gupta et al., 2008; Vanderniepen et al., 2009; Ragonesi et al., 2010; Vitiello et al., 2013; Alavi et al., 2017; Ates et al., 2017). In other words, most papers present the mechanical design and/or controller design, but very little content, if any, is focused on the design of the physical interface with the user. In many cases, the design of the pHMI connection to the robot is addressed as an afterthought in comparison to the extensive effort that goes into the kinematic, mechanical, and control system design. However, inadequate attention to the interface components can greatly hamper effectiveness and usability of a wearable or collaborative robot design.

A well-designed pHMI for the forearm should safely and comfortably support the arm throughout the desired range of motion, minimize tracking error, be easy to don and doff, and have reasonable manufacturability. In the traditional approach to orthosis design, an individual's arm is cast and the cast orthosis is then modified to improve comfort in vulnerable regions (Jacobs and Austin, 2014; Coppard and Lohman, 2015; Webster and Murphy, 2018). Although the process achieves an intimate fit with arm geometry, it requires the user's arm during casting, produces a model that may not fit comfortably on other individuals, and takes skilled time to develop. In contrast, a standardized orthosis approach requires an intimate knowledge of the anthropomorphic topology and variations within the target population. Ideally, a single orthosis that performs well with all users would simplify design, reduce cost, and improve alignment consistency. However, a single standardized orthosis means that some level of fit (e.g., tightness and coverage) must be sacrificed in order to accommodate a wider range of individuals.

In order to develop a design tool to assist in the development of standardized orthoses, knowledge of arm shape, deformation, and movement patterns throughout the desired range of motion are needed. Existing data on forearm shape, including effects of pronation and supination movements, were not found in the literature. Therefore, a set of experiments were designed and conducted as outlined in Section Materials and Methods. The aim of the experiments was to gather scanned point cloud data of the forearm with the arm in two poses: (1) a nearly extreme supination pose, and (2) a nearly extreme pronation pose. Point cloud data was normalized, curve-fit with an elliptical model, combined, and used to generate a model of the human forearm for orthosis design purposes. The normalized results, presented in Section Results, characterize the general shape of the forearm and develop a nondimensionalized, thus scalable, model of the human arm.

## Materials and Methods

The protocol followed in this study has been approved by the University of Idaho Institutional Review Board (IRB#:19-087). The study involves digital and analog collection of anthropometric forearm data from human subjects (*N* = 6) that represent a convenience sample of the population. The approach taken to quantify arm geometry is based on white-light scanning and ellipse-fit modeling of the human forearm. A commercial white-light scanner was used to capture arm geometry from 6 healthy participants while their upper arm, forearm, and hands were supported in a desired posture using a custom testing apparatus. To evaluate the error of the 3D scanner, an easily measurable base object, a coffee cup, was chosen to have measurements taken by both 3D scanning methods and using Vernier calipers for comparison. Two forearm poses, pronation and supination, were examined with participants attached to the apparatus. The scanned data measurements were fit with ellipses using a least-squares ellipse-fitting code and compared to anthropometer measurement databases.

### Testing Apparatus

**Figure 1:**
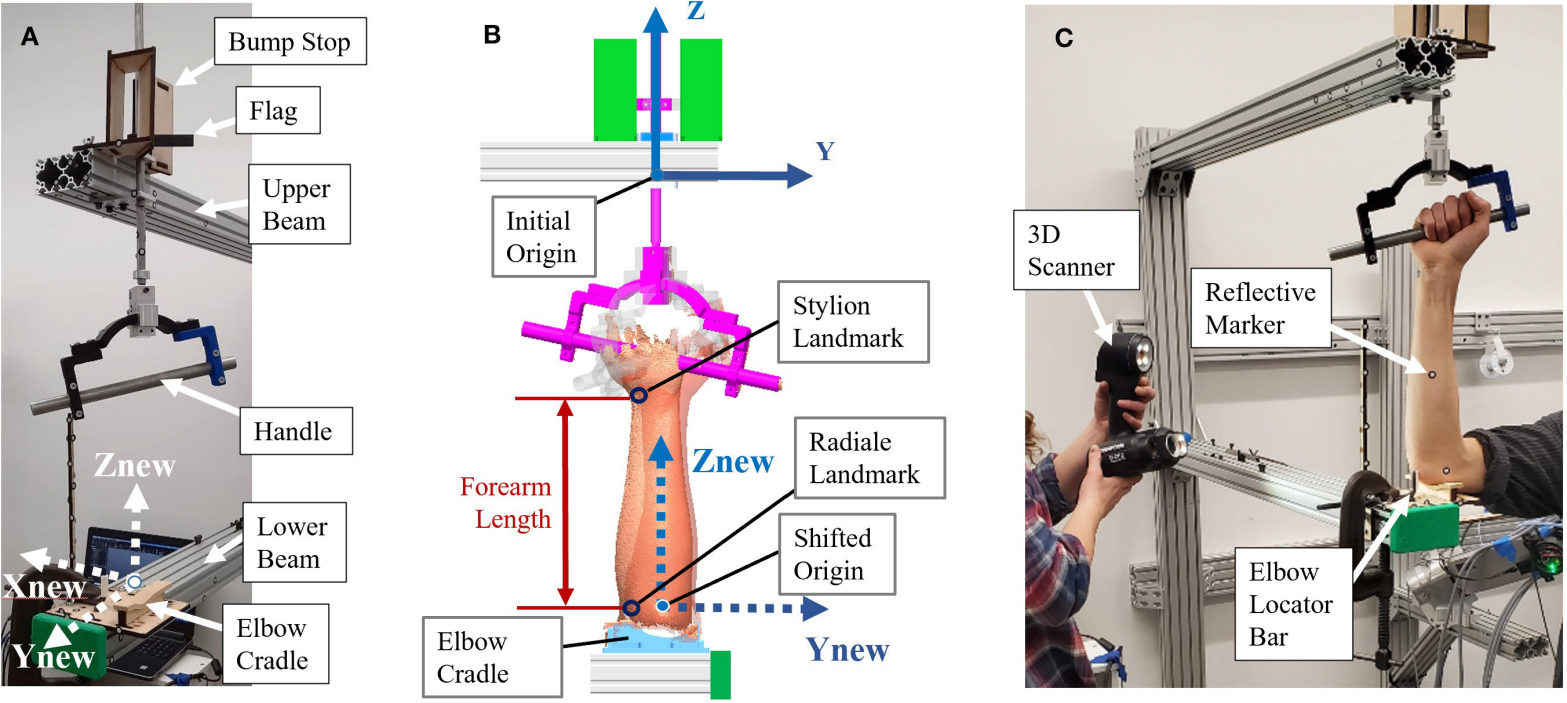
Experimental forearm scanning test setup: **(A)** Test setup components; **(B)** Location of origins, coordinate frames, and key landmarks; **(C)** Subject in setup being scanned.

The relationship between anthropometric landmarks and the rotation axis of the forearm was not found in literature, so a testing apparatus was constructed for this purpose. The apparatus contains a forearm jig, consisting of a support structure and a swiveling handle (Figure 1) that mimics the rotational axis commonly used in upper-limb robots to support pronation and supination. The testing structure was composed of two (upper and lower) extruded aluminum beams extending out from a wall. These beams were used to secure the hand and elbow of subjects while allowing line-of-sight access around subject forearms during scanning. The jig setup (Figure 1) consisted of a rotational handle with the grip angled 12° from horizontal, as recommend by Tilley (2001). A 12.70-mm diameter (½-inch) steel rod was attached to the handle, aligned vertically through its center, and extended upward through each of two brackets mounted to the upper extruded aluminum beam. A second extruded aluminum beam, directly below the first, held a humeral cradle made of a 12.70-mm (½-inch) thick piece of profile-cut plywood covered in 6.35-mm (¼-inch) thick medical foam. The profile of this cradle was taken from a section of scanned arm and offset to provide clearance for larger arms. A rigid bar, that will be referred to as a “flag,” was clamped to the vertical rod, and two bump stops were fixed to the upper aluminum beam. The flag contacted one stop in supination and the other stop in pronation to provide consistent rotations between subjects. The aluminum beam of the testing structure extended from the wall roughly one meter to allow space to walk around the subject during the scanning process. The interface of the aluminum structure was designed to secure the elbow in the apparatus while enabling wrist pronation and supination. The supination stop was set at roughly 80° of supination and the pronation stop at 40° of pronation representing a functional forearm ROM (Figure 1).

### Scanning Procedure

The testing apparatus and forearm was scanned using a Go!SCAN 50, 3D scanner from Creaform. (Creaform Inc, 2015). The object and targets are recognized by the scanner from a coded pattern of light projected from a white light (LED) source and target 3D locations of stickers are reported to the scanning software to further improve accuracy of intra-scan feature alignment (Creaform Inc, 2015). The use of positioning targets reduces scan error by providing a rigid and accurate locating feature. An initial scan of the apparatus marked with adhesive targets provided a starting template to which subsequent arm scans were located by the scanner software. This automatically placed all scans in the same location and orientation with respect to the setup. Targets were placed on the aluminum beams and the scanning aid wand to assist in scan capture. The scanner could not capture targets on both beams at the same time, so the scanning aid wand targets gave the scanner a rigid reference as it scanned down the arm to improve scanner registration. These targets were first scanned without a subject and saved as a template from which subject scans were later run. In this template, a coordinate system was placed with the *Z*-direction pointing upward through the handle pronosupination rotation axis (defined as the axis of a cylinder made from sampled scan points of the rod). The origin was located on the bottom side of the upper aluminum beam, and the positive *X*-direction was placed normal to the side face of the aluminum beam toward the subject (Figure 1B).

**Figure 2:**
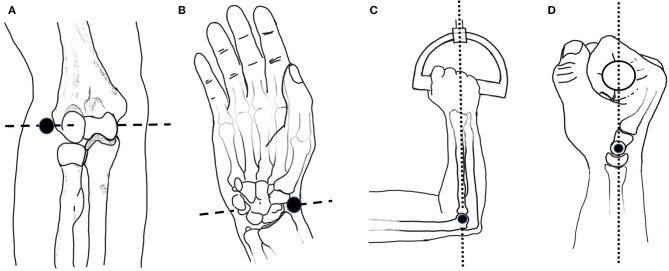
Anatomical landmarks and rotation axes: **(A)** Elbow axis (dashed line) and radiale landmark (black dot); **(B)** Wrist flexion/extension axis (dashed line) and stylion landmark (black dot); **(C)** Upper arm at horizontal with elbow at 90 degrees and handle rotation axis (dotted line) passing through the 4^th^ metacarpophalangeal joint; **(D)** Grip alignment between wrist flexion-extension axis (black dot) and handle rotation axis (dotted line). Images adapted from Neumann (2017).

Five males and one female between the ages of 22 and 41 participated in the arm scan study and represent a convenience sample of the general population. During a pre-scanning procedure, optical positioning targets were placed on the subject's arm at the subject's wrist flexion and extension rotational axis, and elbow rotational axis as estimated by the radiale and stylion landmarks, respectively (Figure 2). These landmarks were chosen to lie along the radial and ulnar centerlines in supination, which have good palpable features and therefore have good identification and repeatability. The targets were placed in the supination pose with the subject's arm bent to 90° and the palm facing posteriorly to reduce target movement due to skin sliding relative to bone structure between anatomical positions. Radiale-stylion length measures were taken from these landmarks, and forearm circumference measures were taken at the elbow crease in this same pose. Subject anthropometric measures are reported in Table 1. Positioning target stickers placed on the subject's skin in these key locations provide reference points for data analysis. The origin of this system is located at the *Z* location of the positioning sticker placed on the radiale landmark. The positioning target placed on the stylion landmark was used to trim the dataset. The elbow crease, as determined visually, was also a dataset trimming location.

**Table 1:** The anthropometric data collected from the six subjects in the study.

**Subject no**.	**Height (m)**	**BMI (kg/m^2^)**	**Radiale-Stylion length (mm)**	**Relaxed forearm circumference (mm)**	**Flexed forearm circumference (mm)**	**Wrist circumference (mm)**
1	1.778	24.0	251	296	306	-
2	1.562	24.7	239	269	269	162
3	1.575	28.1	228	279	279	150
4	1.702	19.9	243	268	272	161
5	1.807	22.8	233	268	275	161
6	1.949	20.2	275	283	286	171

Each subject scan was performed with the subject's forearm placed in the apparatus in the desired pose. Once positioned, each subject was instructed how to perform the desired movements and encouraged to get comfortable in the jig and swivel between both poses to check for comfort. The subject was also questioned on the comfort of the rotational constraint imposed by the jig. The wrist was left unrestrained and the subject was instructed to try to keep their wrist angle (i.e., flexion/extension) the same during the experiment while maintaining a consistent elbow location between scans. The chair height was adjusted to allow the upper arm to be horizontal and the elbow at 90° in the setup (Figure 2C). In the coronal plane, the subject's wrist and elbow rotational centers were aligned with the rotational axis (Figure 2D). The subject's upper arm was placed in the humeral cradle to help maintain elbow position. A locator bar was adjusted to contact the subject's anterior elbow surface, and the subject was directed to maintain contact with the bar during scanning.

Scanning was done in two stages to allow the operator to completely scan the forearm. One stage swept from the left side of the subject clockwise to the aluminum beam, and the other stage swept from subject side of the aluminum beam to the right side of the subject. Each stage started with scanning setup targets to initialize the new stage and then was swept continuously from top to bottom. Coverage from each stage was overlapped to ensure a complete surface was captured. The handle of the setup jig was set at a fixed value of ~12°, so the orientation of the wrist deviation axis was considered to remain constant. Subjects were scanned once in supination and once in pronation. One subject participated in a repeated scan study in which supination was scanned twice, then pronation twice, and then supination and pronation were each scanned a final time. During the repeated scan study, the subject remained in the setup the whole time, alternating between poses when instructed. Subjects were instructed to maintain a pose while the scan data was visually checked for holes and completeness and rescanned as needed before the being allowed to move.

Scans were taken and processed using a proprietary software for Creaform scanners (VXElements VX8). The “semi-rigid positioning” and “use natural features” settings within the VXElements software were selected to normalize data point spread and improve scan registration. The “semi-rigid positioning” setting allows extra error in the scanner registration algorithm to accommodate scanning people because people move slightly even when trying to remain still (Crennen, 2017). The “use natural features” setting lets the scanner use features it scans as registration landmarks (Creaform Inc, 2015). Scanner resolution was set at 0.500 mm. 3D mesh geometry of the object is created in VXmodel by the software from each camera frame captured by the scanner.

### Anthropometric Measurements

Numerous anthropometric databases are available that provide general measures of size, shape, and composition for particular populations (National Center for Health Statistics, 2011; Gordon et al., 2014; Fryar et al., 2016; Löffler-Wirth et al., 2016). However, the data provided lacks the information necessary to design for a close fit with arm topography. Bone, muscle, skin, and subcutaneous tissue are the main components that make up the resulting peripheral shape. As the arm and underlying bones (i.e., radius and ulna) move, the muscles involved in their movement change shape and location. These structures play an integral part in determining how well a user's arm will fit a particular orthosis.

**Figure 3:**
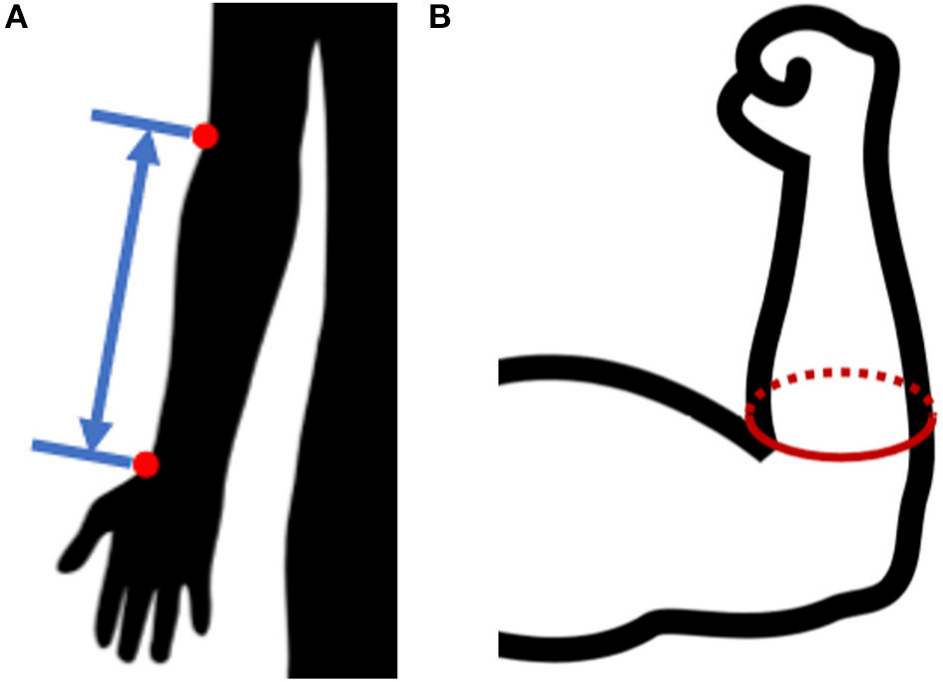
Anthropometric forearm measurements: **(A)** radiale-stylion length, and **(B)** flexed forearm circumference.

The present study collected basic anthropometric measures in conjunction with 3D scan data to allow correlation with databases like the 2011-2014 NHANES 3 study by the CDC (Fryar et al., 2016) and the 2012 ANSUR 2 study by the US Army (Gordon et al., 2014). Measures recorded included: age, gender, height, weight, radiale-stylion length (Figure 3A), flexed forearm circumference, relaxed forearm circumference, and wrist circumference. Measures were taken following to the procedures laid out in the ANSUR 2 manual (Gordon et al., 2014), using a combination of a measuring tape and a set of jumbo-sized Vernier calipers. Throughout the remainder of the paper, radiale-stylion length, or RS length, will be used to represent forearm length (Figure 3A), and forearm circumference will be measured as the circumference around the forearm just above the junction between the upper arm and the forearm (Figure 3B). The forearm circumference measure is taken with the upper arm extended horizontally forward, elbow flexed to 90°, and fist clenched with palm facing the head (as outlined in Gordon et al., 2014). All subject data was anonymized by a subject number.

### Ellipse Fitting

**Figure 4:**
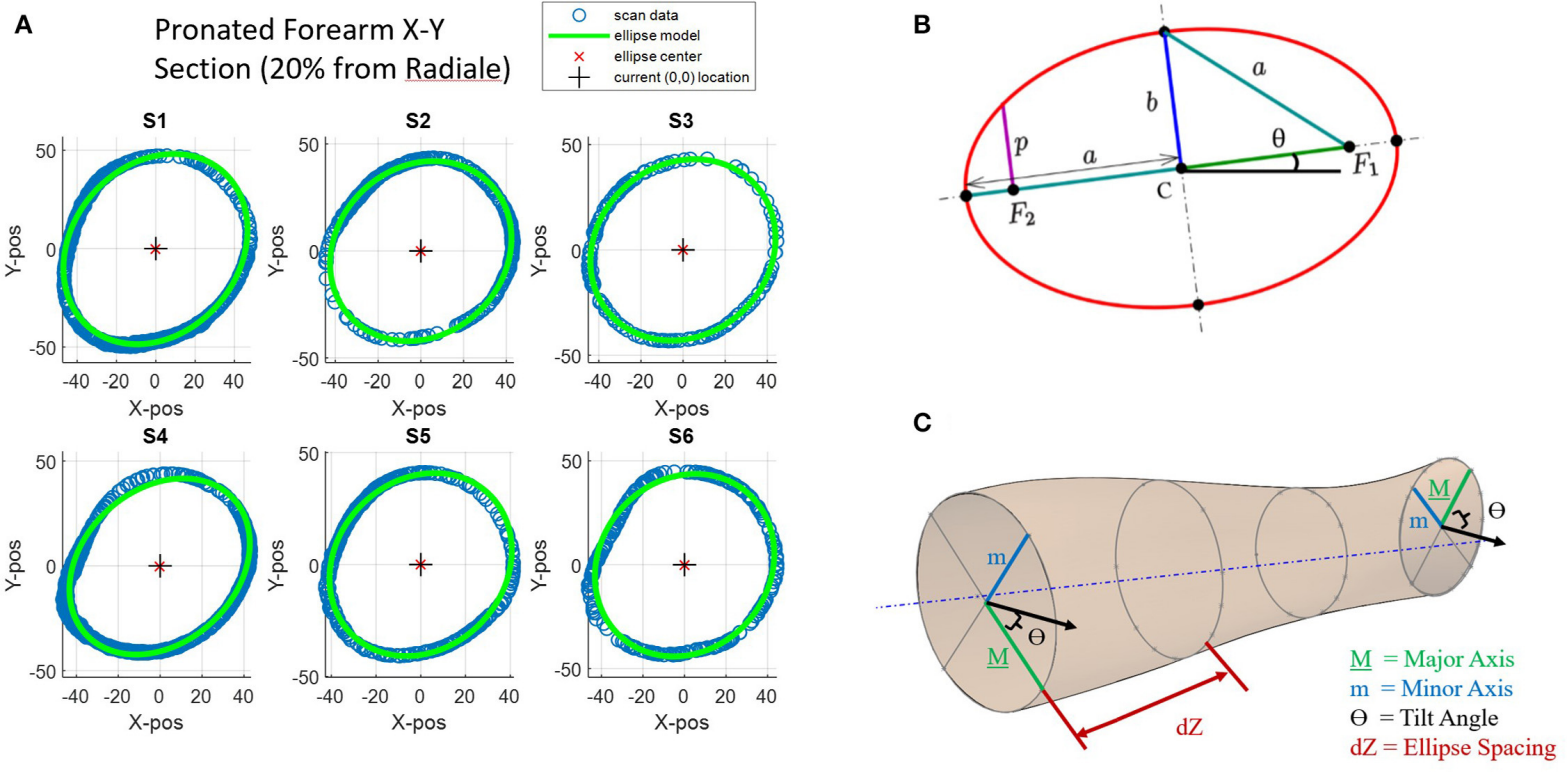
Key ellipse-fitting parameters and approach: **(A)** Transverse slices of forearm data for subjects 1–6 (S1–S6) and 2D ellipse-fit models. **(B)** General parameters of a 2D ellipse include major diameter *a*, minor diameter *b*, angle of tilt θ, center point *C*, and foci *F*_1_ and *F*_2_. **(C)** Ellipse-fit parameters used to generate 2D elliptical slices in increments *dZ* along the longitudinal axis generate a 3D forearm model.

Scanner datasets are unorganized point clouds and therefore make inefficient surface models. Noise and superfluous data points in the data set have been reduced or removed through regional downsampling and fitting elliptical profiles to the downsampled datasets. Downsampling was achieved by moving the ellipse center to the origin, converting to polar coordinates, and then averaging values within 10° windows around the scan. Once downsampled, elliptical profiles were fit to transverse slices of point cloud scan data along the length of the forearm rotation axis. The ellipse fitting method uses a least-squares ellipse-fitting code written by Gal (2003). Ellipse parameters from fitted ellipses convert 3D coordinates into meaningful 2D shape characteristics as illustrated in Figure 4. This 2D approach was continued down the length of the arm modeling thin slices of arm scan data at each of 17 forearm locations from 20 to 100% RS length. At each interval, an ellipse was fit to data spanning a ±1% RS length band. Together, ellipses at normalized arm locations from each subject (Figure 4A) produce a set of ellipse parameters (Figure 4B) at each slice that can be used to recreate a 3D surface of forearm geometry (Figure 4C). Ellipse parameters were normalized, curve-fit with 3rd-order polynomials as a function of axial distance from the radiale landmark.

Ellipses are conics defined by a common quadratic polynomial and appear many places in nature including planetary orbits. The circle is a special case of an ellipse where the major and minor axes are equal in length. Key parameters of an ellipse (Figure 4B) are the center point location (point C), the major axis (dimension *a*), the minor axis (dimension *b*), and the foci (points *F*_1_, *F*_2_) (Downs, 2003). For this experiment, a tilted ellipse is considered, so tilt angle (θ) is also included. A major characteristic not found in Figure 4 is circularity. Circularity is defined for this study as the ratio of minor axis length (*b*) to major axis length (*a*). A circularity value of 1 indicates that the ellipse has equal major and minor axes and is therefore a circle. As the circularity of the ellipse nears 1, the ellipse tilt angles become unstable as small errors in axis lengths can cause large angular errors.

### Single-Subject vs. Combined-Subjects Models

Ellipse-fit models were created first for individual subjects, a single-subject (SS) model, based on individual scan data. Longitudinal slice locations and ellipse center locations for each SS model are normalized based on subject RS length, while ellipse major and minor axes are normalized based on forearm circumference. At each cross-sectional slice, ellipse parameters include: (1) normalized major axis length, (2) normalized minor axis length, (3) normalized center point location in *X*, (4) normalized center point location in *Y*, (5) ellipse tilt angle, and (6) circularity ratio. The six normalized SS models were then combined by averaging ellipse parameters into combined-subjects (CS) models.

Two CS models were developed. A first model was based on averaged SS models from the setup as-is (i.e., using the elbow cradle and the self-selected wrist orientation). A second model was developed based on averaged SS models after each SS model had first been re-aligned to common centers near the wrist and elbow. The first combined-subjects model (CS1) assumes the jig rotation axis of the experimental setup represents the anatomical pronosupination axis of each subject and averages out variations in wrist placement to arrive at a general population model. The second combined-subjects model (CS2) first aligns the scans of individuals to common centers at each end of the forearm before averaging ellipse parameters. These alignment locations were chosen at 20 and 100% RS length, as a distance of 20% RS length was reliably above the forearm crease, thereby avoiding scan artifacts from the biceps.

Although the two CS models are largely similar, a significant difference lies in the location of the ellipse center points and their standard deviations. Standard deviations in CS1 provide insight into the variability in self-selected arm placement within the setup, while the overall model of CS2 provides the best overall representation of normalized forearm shape for development of a generalized forearm model and for use in orthosis design.

### Scanning Error Evaluation

The Go!SCAN 50 takes 550,000 measurements per second at a resolution of 0.500 mm with a reported accuracy up to 0.100 mm and a volumetric accuracy of 0.300 mm/m if positioning targets are used and the object presents adequate geometry or color texture (Creaform Inc). Details on how this error was evaluated were not specified, so a static rigid object of measurable size was used to evaluate measurement error in the scanning setup.

**Figure 5:**
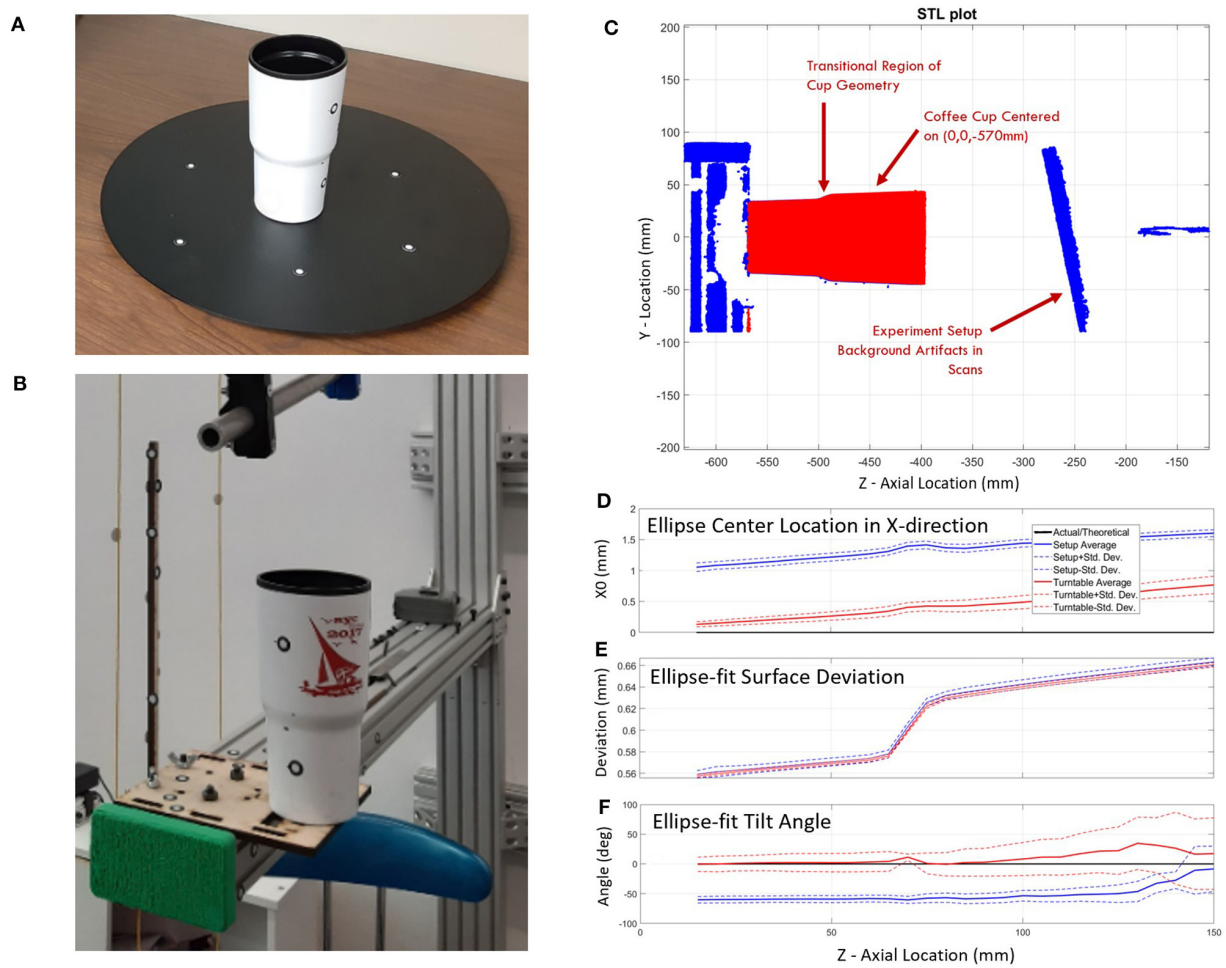
Scanning error study using a coffee cup of known dimensions: **(A)** on turntable and **(B)** in the experimental setup. A comparison between the turntable and experimental setup scan **(C)** show: **(D)** an X-direction bias in the center location of the ellipse, **(E)** increased deviations from the ellipse-fit surface for larger diameters and higher cup heights, and **(F)** stable ellipse tilt angles on both setups over the first 100 mm and increasing variability over the last 50 mm.

Scanner registration error can be largely affected by object shape and visible positioning targets. A test was performed to evaluate the error of the 3D scanner using a stationary object with known dimensions. A coffee cup was chosen for this study as it has a roughly cylindrical shape similar to a human arm. Two scanning methods were used to create the point mesh data of the coffee cup: (1) a traditional scanning method using a turntable, and (2) the experimental setup (Figure 5). The turntable (Figure 5A) represented a well-controlled traditional scanning environment that had six positioning targets with at least three visible to the scanner throughout the scan. The experiment setup (Figure 5B) was less controlled and involved the operator walking around the coffee cup to complete the scan. This setup had obstacles that interrupted the scanner path and limited positioning target visibility. Data points from both studies were run through the ellipse-fitting code to evaluate the error of the scanner in each environment. Dimensions were also recorded manually from the coffee cup using Vernier calipers.

## Results

The results are split into five sections: Error Evaluation, Single-Subject Modeling, Combined-Subjects Modeling, Shape Changes between Pronation and Supination, and Application of the Ellipse-Fit Model. Section Error Evaluation provides the expected error using our proposed method. Section Single Subject (SS) Forearm Modeling illustrates the form of raw data from single subjects and the presence of misalignments. Section Combined-Subjects (CS) Forearm Modeling presents: (a) two types of combined-subjects models, with and without additional alignment, and (b) the primary results of this research in the form of a set of equations that construct a scalable 3D model of the human forearm. The fourth topic (Section Shape Changes between Pronation and Supination) emphasizes the variation in forearm shape during pronation and supination. Section Application of the Ellipse Fit provides an example of using the forearm model in analyzing the model performance with respect to the scan data and implications on the design of orthoses from the tabulated equations and data from the ANSUR 2 database.

### Error Evaluation

Error evaluation included consideration of different scanning approaches and inconsistencies between repeated scans. Error introduced by different scanning methods was evaluated by capturing a static object (a coffee cup) with two different scanning methods, whereas error introduced by the scanner and subject were evaluated by taking repeated scans of the same subject with a single scanning method.

#### Scanning Method Comparison

The error evaluation study compared scanning accuracy of a plastic coffee cup using two different scanning methods: the traditional turntable scanning method and the experimental scanning method. In the coffee cup experiment, it was assumed that the cup had a perfectly circular cross-section due to perceived quality of manufacturing tolerances. This near perfect circularity would cause unstable ellipse tilt values where small length errors can cause large tilt angle errors. This was seen in the datasets of all experiments as the major-to-minor axis ratio neared 1. Ellipse center coordinates in the *X* direction showed an average starting bias of 0.13 mm that drifted 0.65 mm over the 150 mm cup length for the turntable setup while the experimental setup showed an average starting bias of 1.06 mm that drifted 0.55 mm over the same distance (Figure 5D). *Y*-direction center coordinates started with average biases of 0.05 and 0.07 mm and drifted to 0.43 and 0.72 mm for the turntable and experimental setup, respectively. The maximum mean deviation between the raw data and the fit ellipses is <0.68 mm (Figure 5E). The mean deviation of the data cloud from ellipse-fit is minimal at the lower side of the transitional section of the cup and maximal farthest from the base plane. Deviation grew sharply at the transition feature. The standard deviation of distance from raw data to ellipse fits shows a similar pattern but stays below 0.39 mm. Ellipse axis lengths of both setups were within 2% of diameters found using calipers. The ellipse tilt angle remained relatively stable in both setups (0° for the turntable and −60° for the experimental setup) over the first 100 mm of the cup's axial location and displays a pronounced increase in variability over the last 50 mm (Figure 5F).

#### Error in Repeated Scans

Experimental data captured using a 3D scanner includes scanner instrument error (precision and accuracy), scanning registration errors from arm geometry and positioning target spacing, and inclusion of erroneous data points captured during both voluntary and involuntary human movement (e.g., breathing). A repeatability check measured the error between successive scans of the same individual in the same session (i.e., without leaving the setup). In this check, a randomly selected subject was scanned three times in each pronated and supinated pose to evaluate scanner errors. The subject stayed in the setup the entire time, while attempting to hold the pose and alternate between poses when instructed. Subtle shifts were noticed in targets between repeat scans down the length of the arm. Ellipse parameter data was curve-fit with 3rd-order polynomials, and goodness-of-fit statistics were found for the resulting curve fits to quantify data variation. *R*^2^ values are above 0.93, and root mean square error (RMSE) range from 0.66 to 0.97 mm for distance measurements. Tilt angle RMSE range from 2.5° to 4.0°. These represent baseline variations of the scanner experiment and ellipse-fit method. The study has a mean deviation between raw and ellipse-fit data of <1.5 mm with a standard deviation of <0.8 mm. These values are obtained from an SS model without additional data manipulation for scan alignment.

### Single Subject (SS) Forearm Modeling

**Figure 6:**
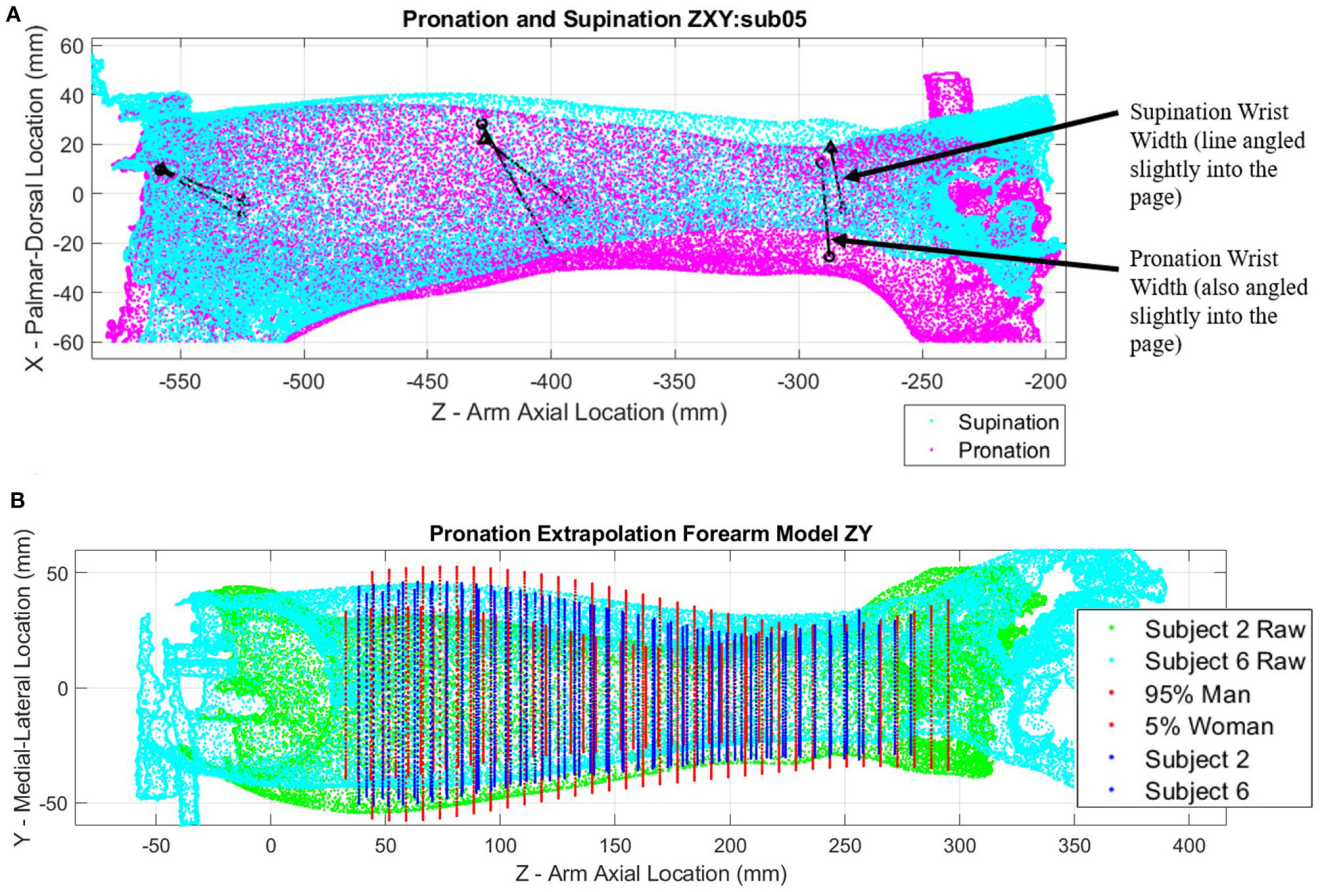
Point clouds of arm scans and ellipse fit extrapolation modeling: **(A)** Pronation (magenta) and supination (cyan) point cloud poses with landmark target locations (black points) connected by black lines for subject 5; **(B)** Extrapolated models of forearms constructed with ellipses evenly spaced along forearm length for subjects 2 (green point cloud) and 6 (cyan point cloud), as well as a 5th percentile female and 95th percentile male for comparison.

The right forearms of six subjects were scanned in the testing apparatus in 40° of pronation and 80° of supination. Raw arm surface data including target positions for different poses and subjects were plotted and overlaid for visual inspection. Scans from Subject 5 show relatively large shifts in target positions near the wrist indicating a large misalignment with the rotation axis of the apparatus (Figure 6A). Differences are illustrated by black lines that connect common pairs of medial and lateral scanning targets in each pose. Similar plots of raw scan data for each subject are available in Supplementary Figures SF1.1 through SF1.6.

Figure 6B shows a relative comparison of two arm scans for subjects of differing stature alongside estimates of 5th percentile female and 95th percentile male models. The shape of each forearm scan is represented by a series of ellipses at 5 mm spacing along the length of the forearm, with each ellipse being fit through a cross-sectional slice of forearm data points. Ellipse parameters without normalization are compared side by side in Supplementary Figures SF2.1–SF2.3 (pronation) and Supplementary Figures SF3.1–SF3.3 (supination).

**Figure 7:**
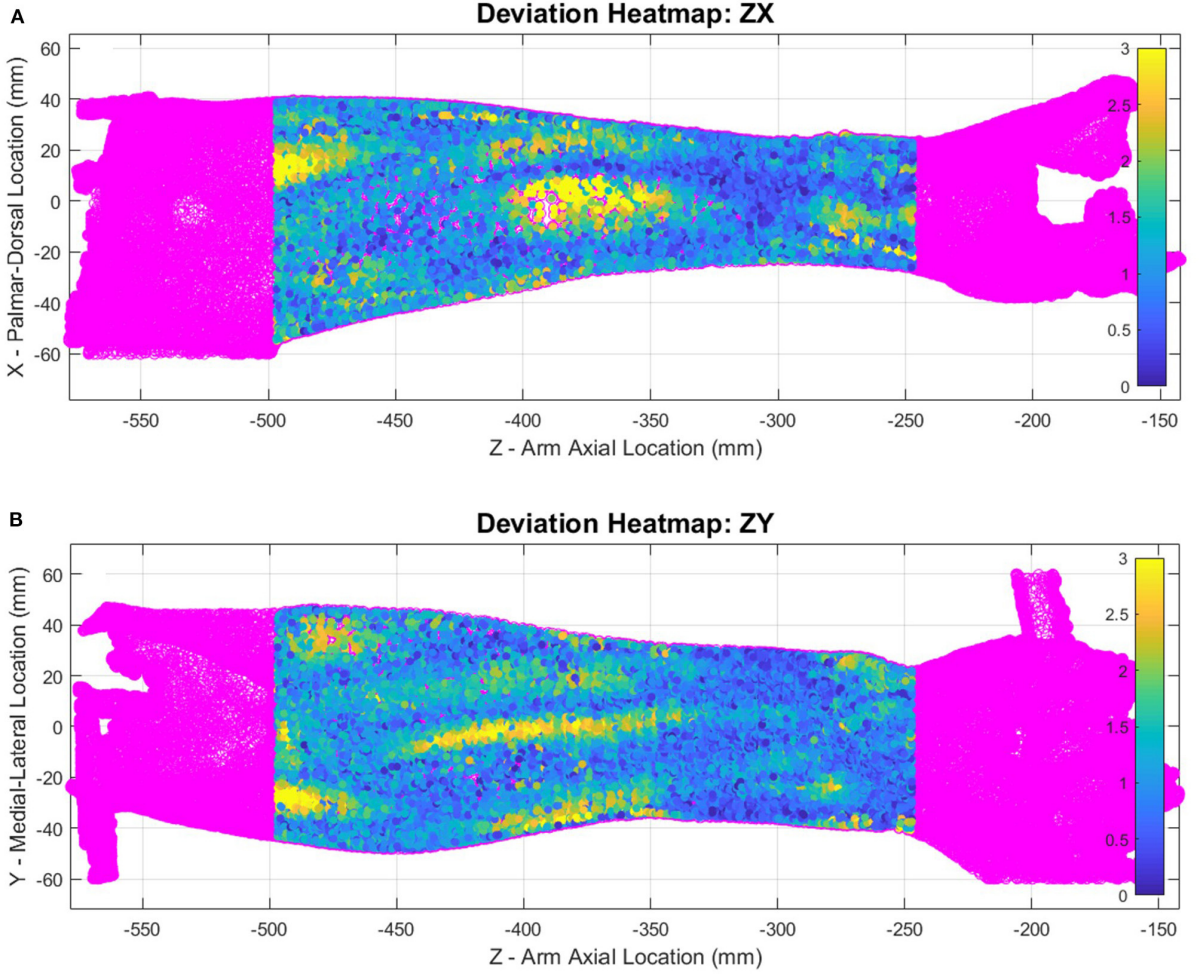
Supination pose heatmap of deviation between scanned data and ellipse-fit model of subject 6, shown in *ZX* plane **(A)** and *ZY* plane **(B)**. The heat map represents mm of deviation.

Heatmap plots of the deviation values of the raw data points were plotted in Cartesian coordinates (Figure 7) to visualize how well the SS ellipse-fit models described the actual arm scan data. Yellow regions indicate places where the arm structure deviates the most from the ellipse fit. Most arms show regions of highest deviation near muscle bellies between the elbow and mid-arm in both poses and at bony prominences near the wrist in pronation. The subject with the most pronounced deviations, subject 6, is shown in Figure 7. Comparisons of single-subject models to raw data are provided for the remaining subjects in Supplementary Figures SF4.1–SF4.6 (pronation) and Supplementary Figures SF5.1–SF5.6 (supination).

### Combined-Subjects (CS) Forearm Modeling

**Figure 8:**
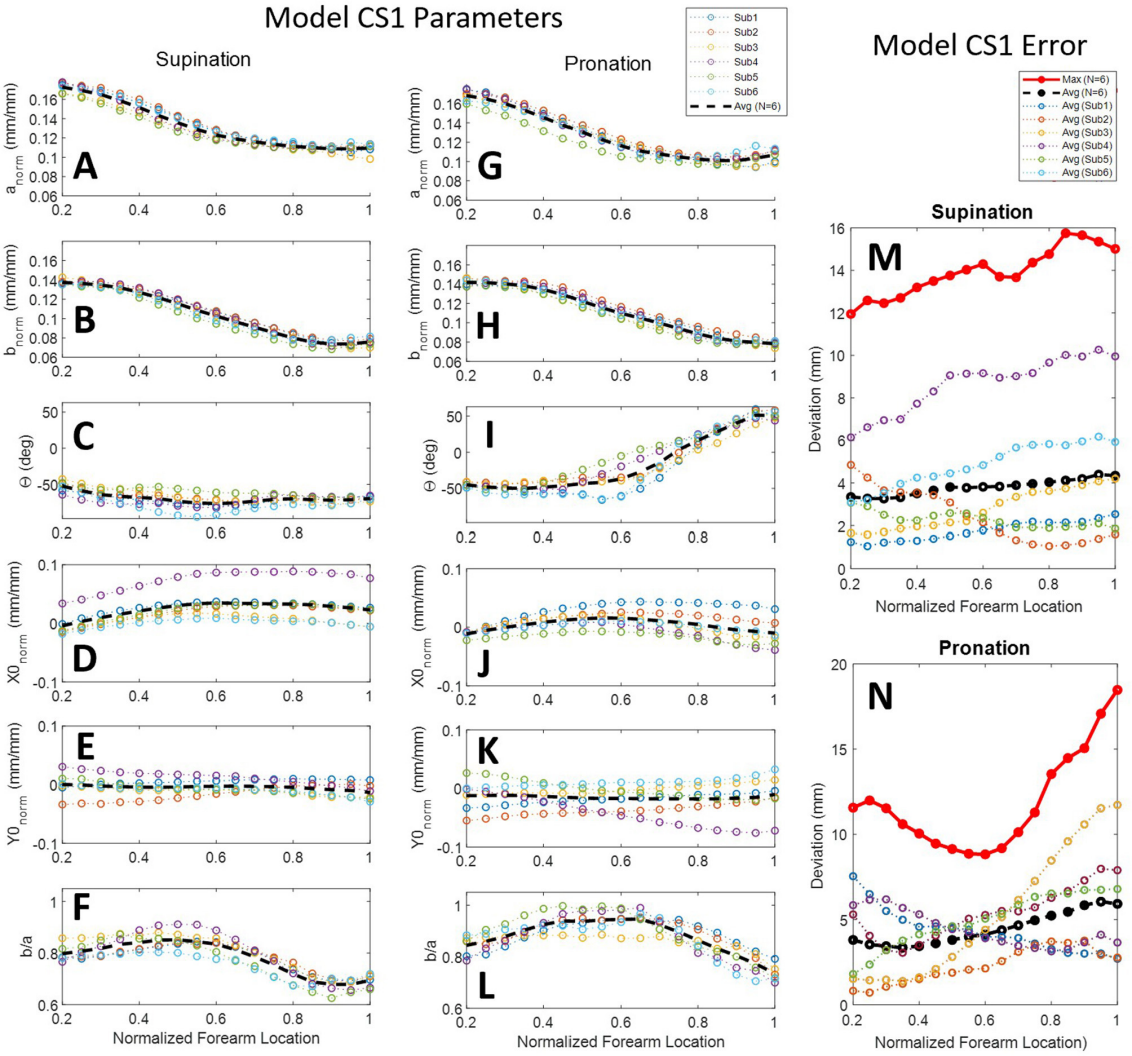
Ellipse-fit model parameters and model error for the combined subjects model CS1: Model parameters for individual subjects, their averages, and their 3rd-order polynomial fit for supination **(A–F)** and pronation **(G–L)** scans; Resulting modeling errors of comparing 3D models generated by the 3rd-order polynomials to the original subject scans are shown in supination **(M)** and pronation **(N)**. Legend for panels **(A–L)** shown above panel **(G)**. Legend for panels **(M,N)** shown above panel **(M)**.

Scan data from all six subjects were normalized and combined into a combined-subjects (CS1) elliptical model for comparison. Normalized major and minor axis lengths, tilt angle, normalized center point coordinates, and circularity for all subjects are illustrated in Figure 8 for supination (Figures 8A–F) and pronation (Figures 8G–L). Average and maximum error at each slice along the forearm are shown in Figures 8M,N. As seen in the figure, parameters are highly consistent between the models for all but the location of ellipse centers. Despite having similar overall profiles, shifts in the data both at the elbow and the wrist indicate variations in alignment between subjects. Misalignment of ellipse centers results in average deviations of 1–12 mm and peak deviations of 8–18 mm between the CS1 model and individual subject scans. CS1 alignment, ellipse parameters, and maximum model error are provided in Supplementary Figures SF6, SF10, SF14 for pronation, and Supplementary Figures SF7, SF11, SF15 for supination, respectively. CS1 scan data, ellipse fit, and downsampled data along the arm from 20 to 100%RS length are available in Supplementary Figures SF18.1 through SF18.17 (pronation) and SF19.1 through SF19.17 (supination). Average model error for each subject is plotted in Supplementary Figure SF22 (pronation), and Supplementary Figure SF23 (supination).

**Figure 9:**
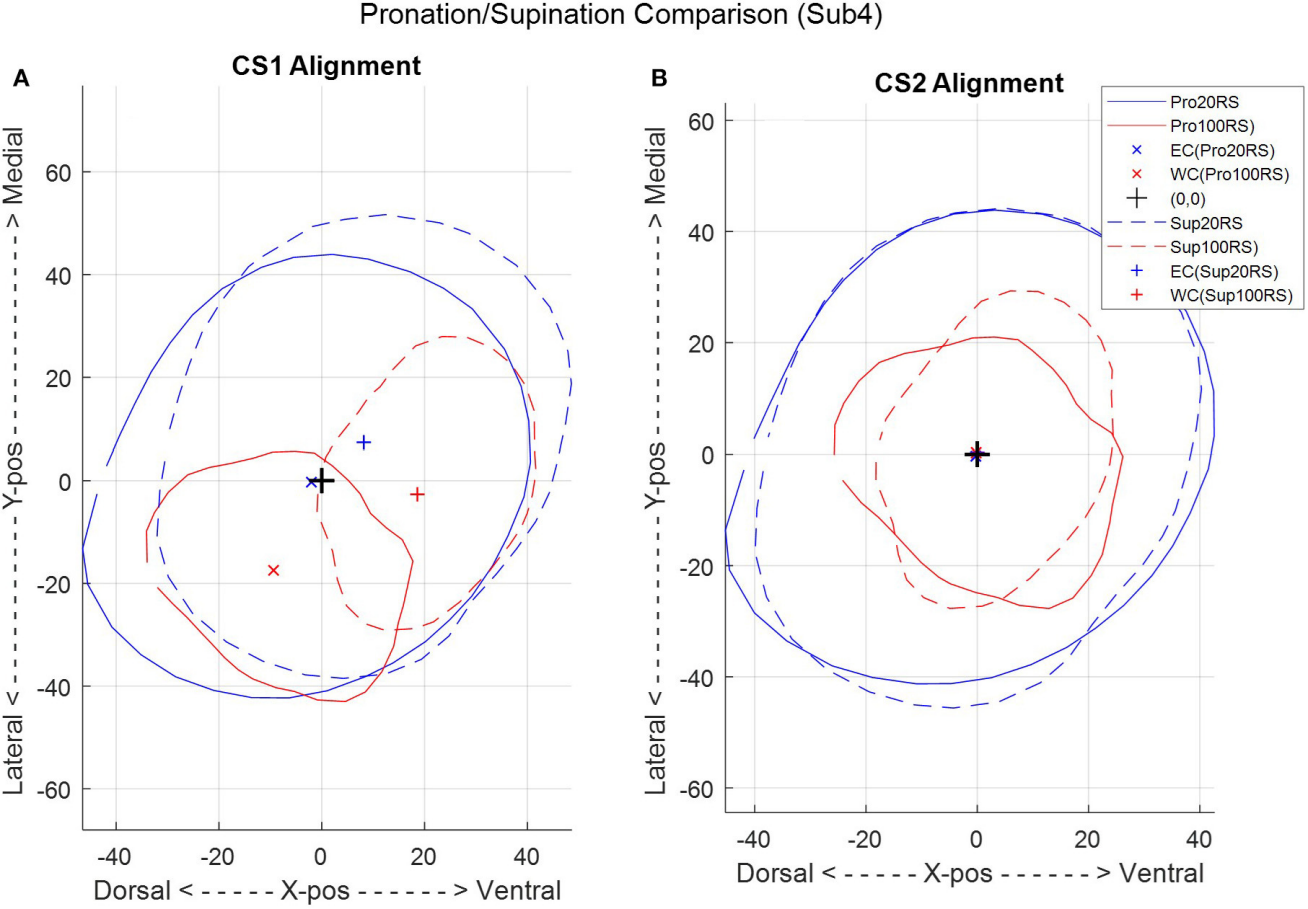
A comparison between the two combined-subject ellipse-fit models, CS1 **(A)** and CS2 **(B)**, for a single subject (subject 4) in pronation (Pro) and supination (Sup) at 20 and 100% radiale-stylion (RS) length from the radiale landmark.

**Figure 10:**
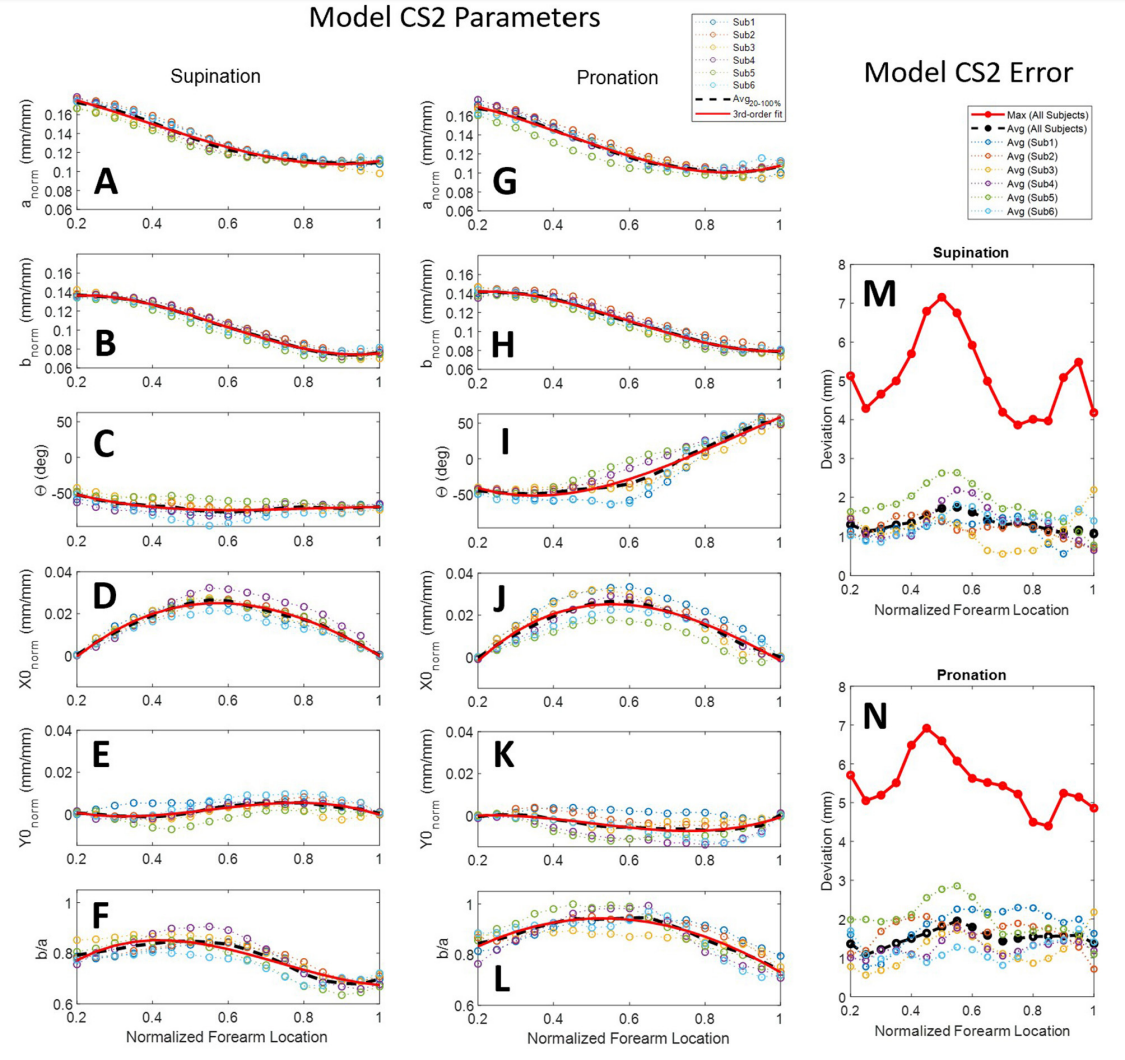
Ellipse-fit model parameters and model error for the aligned combined subjects model CS2: Model parameters for individual subjects, their averages, and their 3rd-order polynomial fit for supination **(A–F)** and pronation **(G–L)** scans; Resulting modeling errors of comparing 3D models generated by the 3rd-order polynomials to the original subject scans are shown in in supination **(M)** and pronation **(N)**. Legend for panels **(A–L)** shown above panel **(G)**. Legend for panels **(M,N)** shown above panel **(M)**.

A second combined-subjects model (CS2) was developed after alignment of the forearm scan with a common vertical axis. Scans were aligned based on the ellipse centers of slices at normalized forearm locations of 0.2 mm/mm (i.e., 20% RS length from the radiale) and 1 mm/mm (i.e., at the stylion). A comparison between forearm alignment in CS1 and CS2 is shown in Figure 9 for subject 4, a subject with one of the largest misalignments to the rotation axis of the apparatus. CS1 (Figure 9A) represents the average of arm locations in the apparatus based on a self-selected wrist placement. The aligned model of CS2 (Figure 9B) provides a more accurate representation of average arm geometry. The resulting ellipse parameters and model error for each subject over the length of the forearm are provided for CS2 in Figure 10. Alignment of the ellipse centers in CS2 lowered average deviations to 1–3 mm and peak deviations to 4–7 mm between the CS2 model and individual subject scans. CS2 alignment, ellipse parameters, and maximum model error are provided in Supplementary Figures SF8, SF12, SF16 for pronation, and SF9, SF13, and SF17 for supination, respectively. CS2 scan data, ellipse fit, and downsampled data along the arm from 20 to 100%RS length are available in Supplementary Figures SF20.1–SF20.17 (pronation) and Supplementary Figures SF21.1–SF21.17 (supination). Average model error for each subject is plotted in Supplementary Figure SF24 (pronation), and Supplementary Figure SF25 (supination).

The statistical *R*^2^ correlations of fit and root-mean-squared error (RMSE) between 3rd-order models and average CS2 ellipse parameters along the normalized forearm axial length are provided in Table 2. The best-fit polynomial equations for CS2 ellipse parameters are given in Table 3. Equations from the table, and two inputs (forearm RS length and circumference), are sufficient to construct a mathematical model of the forearm. The input values can be obtained from a specific individual or from the ANSUR 2 dataset to represent a particular percentile of the population. Similarly, an orthosis model can be generated using these same inputs over the desired region where the orthosis is to be placed. For example, an individual whose RS length is 250 mm with a desired orthosis location along the forearm from 50 to 100 mm from the radiale, would use values for *x* of 0.2 and 0.4 to generate ellipses at either end of the orthosis. An orthosis could then be made in a computer-aided design software by lofting a surface between subsequent elliptical sketches. Additional model resolution can be achieved by generating additional ellipses between the two end sketches, further refining the lofted orthosis surface. It is important to note that the equations use normalized forearm dimensions based on radiale-stylion length and flexed forearm circumference, as described in section Ellipse Fitting.

**Table 2:** RMSE of CS2 3rd-order ellipse-fit model parameter with six-subject averages and *R*^2^ correlation with normalized forearm location.

**Pose**	**Variable**	**3rd-order fit with averaged (*N* = 6) ellipse parameter profiles**			***R*^2^ correlation with normalized forearm location (*Z*)**
		**R^**2**^**	**RMSE (Units)**	**Normalized w.r.t**.	
Pronation	Normalized Major Axis	0.998	0.0041 (mm/mm)	Forearm Circumference	0.898
Supination		0.995	0.0064 (mm/mm)	Forearm Circumference	0.926
Pronation	Normalized Minor Axis	0.999	0.0029 (mm/mm)	Forearm Circumference	0.980
Supination		0.999	0.0021 (mm/mm)	Forearm Circumference	0.972
Pronation	Tilt Angle	0.988	0.285 (rad)	n/a	0.877
Supination		0.941	0.106 (rad)	n/a	0.381
Pronation	Normalized *X* Center	0.985	0.0046 (mm/mm)	RS length	0.020
Supination		0.991	0.0031 (mm/mm)	RS length	0.005
Pronation	Normalized *Y* Center	0.941	0.0028 (mm/mm)	RS length	0.369
Supination		0.951	0.0022 (mm/mm)	RS length	0.391
Pronation	Circularity	0.983	0.0334	n/a	0.279
Supination		0.942	0.0609	n/a	0.657

*Pronation fits highlighted in blue for clarity*.

**Table 3:** Forearm model equations include elliptical parameter best-fit equations from a 3rd-order polynomial fit through the CS2 ellipse parameters, *Y*, vs. normalized axial location, *x*.

**Pose**	**Dependent variable (Y)**	**Independent variable (x)**	**Equation**
**Pronation**	Normalized Major Axis, *a_*norm*_*	Normalized Forearm Location	*Y* = 0.2536 *x*^3^ – 0.3264 *x*^2^ – 0.0004 *x* + 0.1808
	Normalized Minor Axis, *b_*norm*_*	Normalized Forearm Location	*Y* = 0.2882 *x*^3^ – 0.5210 *x*^2^ + 0.1894 *x* + 0.1225
	Tilt Angle, *theta*	Normalized Forearm Location	*Y* = −4.8876 *x*^3^ + 12.8539 *x*^2^ – 7.1845 *x* + 0.2399
	Normalized X Center, *X_0,*norm*_*	Normalized Forearm Location	*Y* = 0.1019 *x*^3^ – 0.3504 *x*^2^ + 0.2942 *x* – 0.0475
	Normalized Y Center, *Y_0,*norm*_*	Normalized Forearm Location	*Y* = 0.1085 *x*^3^ – 0.1620 *x*^2^ + 0.0588 *x* – 0.0062
	Circularity, *b/a*	Normalized Forearm Location	*Y* = 0.0977 *x*^3^ – 1.1571 *x*^2^ + 1.1395 *x* + 0.6501
**Supination**	Normalized Major Axis, *a_*norm*_*	Normalized Forearm Location	*Y* = 0.1713 *x*^3^ – 0.1990 *x*^2^ – 0.0535 *x* + 0.1922
	Normalized Minor Axis, *b_*norm*_*	Normalized Forearm Location	*Y* = 0.3329 *x*^3^ – 0.5798 *x*^2^ + 0.2068 *x* + 0.1156
	Tilt Angle, *theta*	Normalized Forearm Location	*Y* = −2.3585 *x*^3^ + 5.6793 *x*^2^ – 4.2738 *x* – 0.2692
	Normalized X Center, *X_0,*norm*_*	Normalized Forearm Location	*Y* = 0.0573 *x*^3^ – 0.2607 *x*^2^ + 0.2423 *x* – 0.0390
	Normalized Y Center, *Y_0,*norm*_,*	Normalized Forearm Location	*Y* = −0.1361 *x*^3^ + 0.2266 *x*^2^ – 0.1043 *x* + 0.0135
	Circularity, *b/a*	Normalized Forearm Location	*Y* = 1.4204 *x*^3^ – 3.1362 *x*^2^ + 1.8798 *x* + 0.5104
**Both**	Actual major axis, *a*	Normalized major axis, *a_*norm*_*	*Y* = (User Forearm Circumference in mm) · *x*
	Actual minor axis, *b*	Normalized minor axis, *b_*norm*_*	*Y* = (User Forearm Circumference in mm) · *x*
	Actual *X* center, *X*_0_	Normalized *X* center, *X*_0,norm_	*Y* = (User Radiale-Stylion Length in mm) · *x*
	Actual *Y* center, *Y*_0_	Normalized *Y* center, *Y*_0,norm_	*Y* = (User Radiale-Stylion Length in mm) · *x*
	Actual forearm location	Normalized forearm location	*Y* = (User Radiale-Stylion Length in mm) · *x*
	Circularity, *b/a*	Circularity, *b_*norm*_*/*a_*norm*_*	*Y* = *x*

*The last six equations provide parameters for both poses based on forearm measures of circumference or radiale-stylion length. Equations corresponding to pronation, supination, and both poses are separated by blue highlight for clarity*.

### Shape Changes Between Pronation and Supination

**Figure 11:**
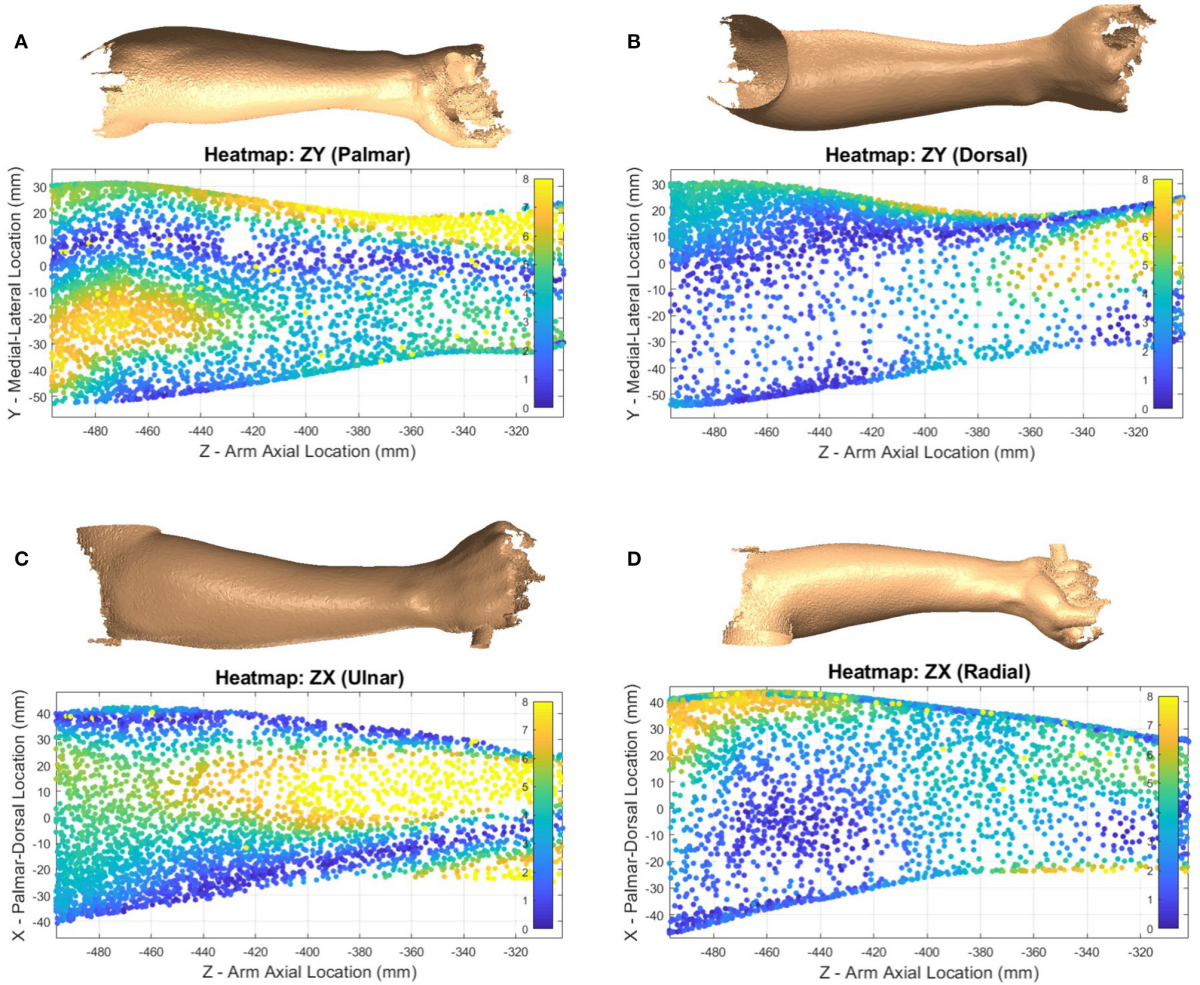
Heatmap showing supination ellipse-fit data from model CS1 compared to the raw pronation data showing evaluate volume changes between poses for subject 2. Heatmaps shown from palmar side ZY plane **(A)**, dorsal side ZY plane **(B)**, ulnar side ZX plane **(C)**, and radial side ZX plane **(D)** represent the amount of deviation between pronation and supination in millimeters.

The forearm shape changes significantly between pronation and supination poses. The size of deviations for a single subject (subject 2) are illustrated with heatmaps in Figure 11. Blue regions show where there is little positional change of the arm surface between poses, while yellow regions show where the shape changes by at least 8 mm. Data was trimmed using the origin *X* and *Y* planes to remove far side data points from view. Each view (palmar, dorsal, ulnar, and radial) was named by hand directions and forearm bone landmarks to indicate which surface is shown.

Another major difference between poses can be seen in the tilt angle, θ, of Figure 10. The angle varies substantially between pronation and supination poses, staying relatively constant from 20 to 60% RS length around −50°, but in pronation increases almost linearly from 60 to 100% RS length to +50°. Circularity is also lower in supination indicating a more elliptical shape with larger major axis and smaller minor axis.

### Application of the Ellipse Fit

**Figure 12:**
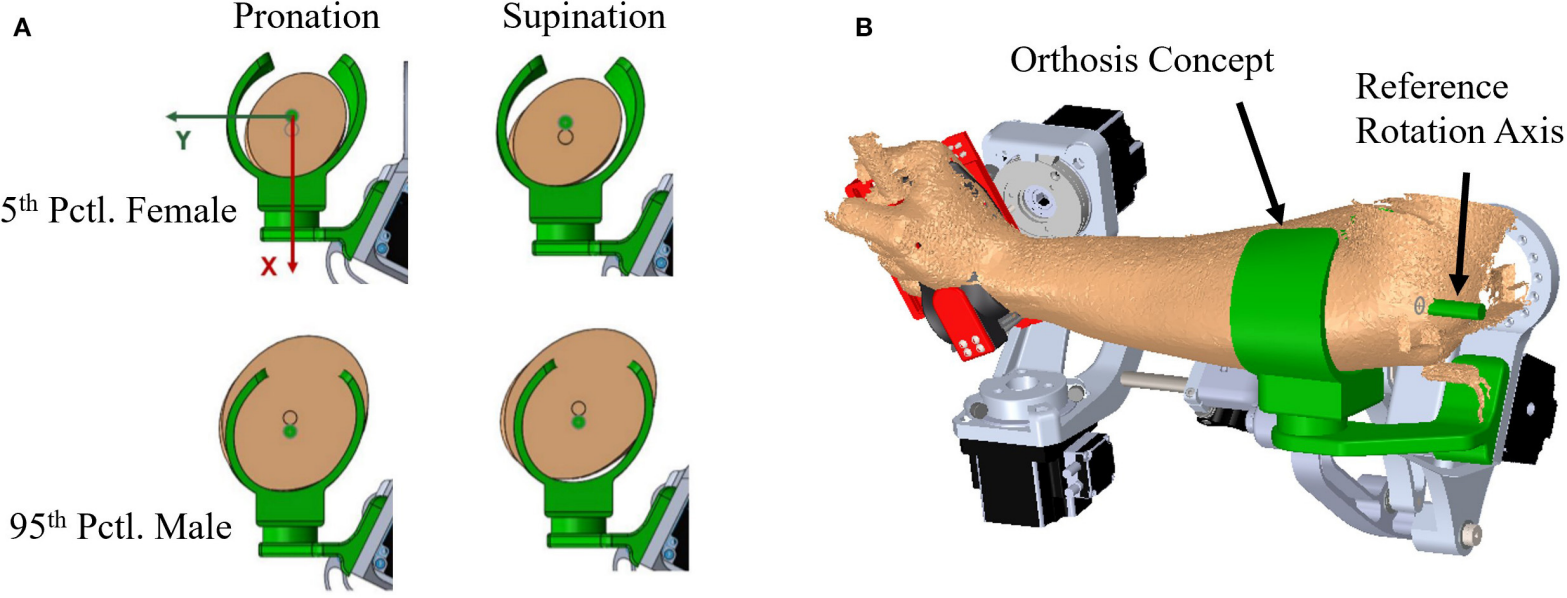
Extrapolated forearm model comparison: **(A)** Models in pronation and supination for 5th percentile female and 95th percentile male are compared to an orthosis model that has been sized using the proposed method to fit scanned arm data from one of the experimental subjects **(B)**.

To illustrate current upper-limb rehabilitation robot practices and the importance of pHMI fit with the user, a single thermoplastic C-channel orthosis of a mean individual was designed in SolidWorks. The orthosis was built using the proposed pronation extrapolation model for use with a robotic exoskeleton system called Blue Sabino. The orthosis attaches to the robot via the inferior side of the cuff, and would secure to the user via a set of hook-and-loop Velcro straps. The orthosis was designed with 165° of wrap on the medial side of the cuff and 120° on the lateral side. The large angle of wrap around the forearm has been used to illustrate the shape provided to support the arm under a wide array of model orientations. However, it should be noted that a cuff of this geometry would not allow easy donning and doffing by users, particularly if rigid. Extrapolation models for both the smallest (i.e., 5th percentile female) and largest (i.e., 95th percentile male) individuals were placed in the model to visualize the geometric performance of the concept (Figure 12). Although a medium-sized cuff has been designed based on the ellipse-fit model, subject evaluations of fit and comfort have not yet taken place.

## Discussion

Geometric analysis of the proposed forearm model provides insight into the design of a standardized orthosis. Forearm scans show significant change in shape between supination and pronation poses. The ellipse-fit model proves useful in constructing a mathematically-driven, scalable, generalized surface model of the human forearm for use in sizing and developing orthosis designs.

### Scanner Error

The coffee cup experiment provides a relatively controlled reference study to quantify the error of the scan and compare scanner error to traditional caliper measurements. The turntable and experiment setup scans were manually aligned to caliper measurements which introduced small errors in dataset location, but has no net effect on each dataset relative to itself. Both setups produced a similar increase in error as the scanner moved away from the turntable or experiment frame where most of the position targets were located.

The experimental setup showed a pronounced ellipse center-point shift in the *X* direction (Figure 5D) that was not present in the *Y* direction nor the turntable data. This 1.06–1.61 mm bias is likely an artifact of the experimental setup and is expected to slightly influence data collection. Turntable data aligns slightly better with the measured data plot, which is expected from a more controlled scan environment. The experiment setup had a pronounced ovalization with a consistent bias to ellipse tilt angle averaging about −60° for the first 100 mm of the cup's length (Figure 5F) suggesting a consistent shape bias due to scanner sensor readings, scanner software, and experiment procedure. This is likely related to the *X*-direction shift. Such a bias was not noticed in the turntable results, which more closely follow the axis ratio of 1 expected from the perfectly circular cross-section assumption. Similar or greater deviations than the discovered 0.39 mm between the raw data and the ellipse-fit cup are to be expected from forearm scans. The error of the ellipse best-fit model in our cup study is 2–3.5 times larger than the error found in other studies of static objects using similar scanners (Dickinson et al., 2016; Kersten et al., 2018; Polo et al., 2019). This is likely due to two of these studies using objects with better fit-locking geometric features, and the third using an abundance of positioning targets on the reference object. This identifies the importance of having tracking stickers close to the geometries being scanned and provides key insight into developing a forearm scanning environment. This supports the addition of the vertical wand with tracking stickers behind the arm that allows for additional visual reference points as the scanner moves along the forearm and away from tracking stickers attached to the extruded aluminum beam.

The one-subject repeatability check highlights the presence of variance in the data. Subtle shifts were seen between subsequent scans of the same subject in the same session. This phenomenon is expected due to a variety of potential causes, one being involuntary movement by the subject, such as breathing. Placement of positioning stickers, scanner movement patterns, and scanning speed are other potential sources that could contribute to the observed error. Identifying the contributions from each source would require an intensive study to isolate their effects. Contributions from involuntary human movement could be further reduced through immobilization of wrist flexion/extension. However, these shifts in the CS1 model were removed from the CS2 model through pre-alignment of the scans.

### Forearm Scans

The forearm is a highly dynamic and deformable mechanism, making it much more complicated to obtain repeatable measurements as compared to more traditional engineering objects. Variability studies using 3rd-order polynomial regression fits and statistics were used to characterize trends and indirectly comment on the quality of the study data in addition to the error studies previously discussed. The 3rd-order polynomial fit with normalized and averaged ellipse parameters of model CS2 have *R*-squared (*R*^2^) values that range from 0.941 to 0.999 with a root-mean-square error (RMSE) of 0.21 to 0.64% of their respective reference measurement used for normalization. *R*^2^ values of CS2 ellipse parameter correlations with normalized forearm location on the other hand have a much wider range. Ellipse axis lengths are highly correlated with forearm location having *R*^2^ values from 0.898 to 0.980. This suggests that forearm circumferential variability is well-explained by location along the length of the forearm and is likely due to a general shared arm structure among the sample population.

In addition to major and minor axes, arm shape is described by ellipse tilt angle and center location. Ellipse tilt angle elicited the most significant difference between pronation and supination, where the tilt angle in pronation was highly correlated with forearm location (*R*^2^ = 0.877) while supination was not (*R*^2^ = 0.381). Not surprisingly, circularity is only moderately correlated with forearm location (*R*^2^ = 0.657) in supination, and very poorly in pronation (*R*^2^ = 0.279). All other ellipse parameters are very poorly correlated with position along the forearm. Pronation generally had a more circular shape to its cross-sections than supination and was nearly circular for two of the subjects between 45 and 65% RS length, measured from the radiale. This resulted in significantly less data points in the scanned point cloud in these regions due to complications in tilt angle as the axis ratios neared 1, an artifact of using ellipses to fit nearly circular objects. The center point position RMSE varied from 0.31 to 0.46 % of RS length in the *X* direction and 0.22 to 0.28% of RS length in the *Y* direction. This equates to a center-to-center RMSE of 0.38–0.54% RS length between the model and the average subject, or 1.0–1.4 mm RMSE for a 50th percentile male.

The model parameters from model CS1 provide insight into the variability in subject positioning relative to a forearm rotation axis by an untrained technician that may be useful in estimating alignment error encountered in upper-limb robot donning. It was noticed that verbal direction to the subject to keep a consistent posture between poses was not sufficient. As a result, he forearm alignment of subjects widely varied. For this reason, the second aligned model CS2 was developed. This second model provides a close approximation of the average arm shape across the subject pool. Model CS2 should be used in developing forearm models for close-fitting orthoses. Model CS1 can be used to see the effects of subject misalignment in a semi-constrained environment. Adapting the handle assembly to use mechanical indexing features to constrain the ulnar and radial epicondyles of the wrist could be used to further reduce variability in alignment between supination and pronation pose scans in the experimental setup. A similar use of indexing features could be used at the elbow to reduce alignment variability of the humeral epicondyles.

### Forearm Deformation

Shape change can have major impacts on orthosis fit and comfort. Modeling the human arm based on optical scanning of tracking stickers is complicated by movement of the bones and muscles through pronosupination under the surface of the skin. Bones move relative to the surface and relative to one another causing subtle changes in location of surface markers. Skin artifacts in axial rotation tend to cause under-rotation of the wrist flexion-extension axis markers, as skin stretches. Mid-arm markers show relative motion of the skin but have little to do with underlying movements. It is also evident from scan geometry that the forearm shortens in length from the supination pose to pronation pose with wrist breadth changing as well. Wrist markers moved an average of 4.84 mm closer together in pronation than in supination, which indicates that the wrist changes size during forearm rotation. Measured distances between lateral pairs of skin markers averaged changes of 0.49 to 5.1 mm suggesting position targets affixed to the skin should not be treated as rigid landmarks if high accuracy is needed. For accurate deformation data and determination of actual rotation centers, skin markers are unsuitable with mid-arm targets especially prone to skin effects. These events suggest two things: (1) alignment of the anatomical rotation center to the fixture rotation axis was accommodated by wrist joint movement, as well as global arm movement instead of pure forearm rotation, and (2) using targets to find the rotation center of the arm is complicated by skin effects and bone topology.

Heatmap plots of a supinated forearm in Figure 7 identify the portions of scan data that deviate most from the SS ellipse-fit model. This particular subject had a low body fat percentage which may have contributed to the larger localized deviations. The deviation patterns in the figure suggest that, at least for thin subjects, bony prominences, superficial tendons, and muscle bellies may be the primary sources of model deviation. Similarly, heatmap plots of a pronated forearm in Figure 11 illustrate regions of largest difference between pronation and supination. The particular subject was fairly well-aligned in the setup and thus would produce a similar heatmap using either of alignments CS1 or CS2. From the figure, it appears that most of the difference near the elbow is caused by the pronator muscles, and most of the difference near the wrist is caused by the ulna and radius. The location of largest changes show regions where rigid orthoses would need the most padding to accommodate misalignment. Conversely, the regions of lowest change indicate regions where rigid orthoses may feasibly support the forearm comfortably during pronosupination movements. The specific regions of high and low change depend heavily on model alignment, thus appropriate selection of rotation axis placement is important.

### Ellipse Fit Application

Skin loading has a critical design limit for user comfort and safety. As illustrated in Figure 12A, a standardized HMI cannot intimately fit all users. A compensation mechanism is needed to ensure high fidelity force transmission to the robot while keeping forces on the user within safe limits. Example compensation mechanisms may include thermoplastic walls of the orthosis that are deformed by tightening Velcro straps until the orthosis fits the user snugly, orthosis walls that linearly slide to contact the user's arm, or a pneumatic cuff that is inflated until adequate contact with the user's arm is achieved. The difference between extreme individuals is a key design criteria that drives the requirements of foam or skin compression to achieve a proper fit. Multiple HMI sizes are commonly used to narrow the band of deviations that the compensation mechanism must accommodate. However, the accommodation bandwidth for any size can be increased by using an HMI profile that more closely matches the subject's anatomical form such as the mathematical forearm model of Table 3.

Figure 12B shows a simple concept of a thermoplastic C-channel-shaped orthosis based off the pronation model of Table 3. Similar designs with Velcro straps are used in other upper limb rehabilitation robots. The figure shows extreme users in a one-size-fits-all design based on the average model in a pronation pose. Several design issues are immediately apparent. First, the smallest and largest users are not aligned to the rotation axis because arm diameters are too small or too large for the designed orthosis. Second, the orthosis walls must be strained in order to contact each user. The model presented in this study allows these deflections to be estimated and included in the design. A third issue arises when the orthosis user rotates his/her arm from pronation to supination, in which case the orthosis no longer matches the user's arm shape. In a rigid shell, this could result in pressure concentrations, gaps, and/or arm alignment changes with potential consequences on arm tissue loading. If the user and robot kinematics stay aligned, this results in an enforced displacement problem where the geometric mismatch represents the desired design condition, and reaction forces can be solved if component material properties are known. Foam is commonly used in standardized orthoses to add comfort and soften the interaction of the orthosis on the user. In an enforced displacement scenario, a foam layer between the orthosis and the user can be used to reduce reaction forces. While the ellipse-fit model shows promise as a means to represent arm topology for arms in pronation and supination, the resulting performance of the model in terms of comfort and support in customized applications needs further evaluation by subjects during both static and non-static tasks.

The effectiveness of this model has yet to be evaluated for resulting fit and comfort with subjects. This model is purely a geometric comparison and neglects deformation and compliance in the human-to-robot system that will likely have impacts on comfort and tracking accuracy. Human variation in size and shape as well as skin properties require a larger sample size. Further refinement of the forearm rotation axis location is also likely needed to optimize performance over a wider range of forearm rotation. This should both improve the exoskeleton performance and patient comfort during rehabilitation. Although the model was implemented virtually through CAD, a physical model in a clinical environment will allow for feedback from patients. The patient feedback will both validate the model and outline areas of potential improvement. A more expansive study with a larger subject pool would also further refine the model to better represent the general population.

## Conclusion

This study provides a tool for assisting in the design of standardized orthoses for use in exoskeleton robotic applications. It establishes a closed-form, scalable model for the interface between the surface of the forearm and a physical human–machine interface. It provides data on both supination and pronation arm shapes allowing for the design of orthoses that accommodate a full functional range of forearm rotations. It also highlights the importance of considering the effects of pronosupination on arm size and shape in designing orthoses for exoskeletons. The developed model can be resized in length and width with a few simple measurements of arm geometry to quickly create a potential pHMI design for a user of arbitrary arm size. This data-driven model of the “average” forearm shape could help designers fabricate orthoses that provide a reasonable fit to a wider array of individuals and improve the generalized fit of prototype pHMIs in rehabilitation robotics research.

## Data Availability Statement

The raw data supporting the conclusions of this article will be made available by the authors, without undue reservation.

## Ethics Statement

The studies involving human participants were reviewed and approved by University of Idaho Institutional Review Board. The patients/participants provided their written informed consent to participate in this study.

## Author Contributions

JP is the senior researcher who guided developments, revised processing scripts, and prepared the final manuscript for submission. JB took the lead on drafting the preliminary manuscript. RC assisted JB with drafting the methods section. MB was the lead graduate student during experimental design, data collection, and initial processing, and provided periodic guidance to JB and RC throughout preparation of the preliminary manuscript. All authors contributed to and approved the final manuscript.

## Conflict of Interest

The authors declare that the research was conducted in the absence of any commercial or financial relationships that could be construed as a potential conflict of interest.
