# Supplementary figures and images for: 3D Scanning of the Forearm for Orthosis and HMI Applications (part 2 of 2)
Source: Front Robot AI. 2021 Apr 14;8:576783. doi: 10.3389/frobt.2021.576783 (PMC8079810; doi:10.3389/frobt.2021.576783)

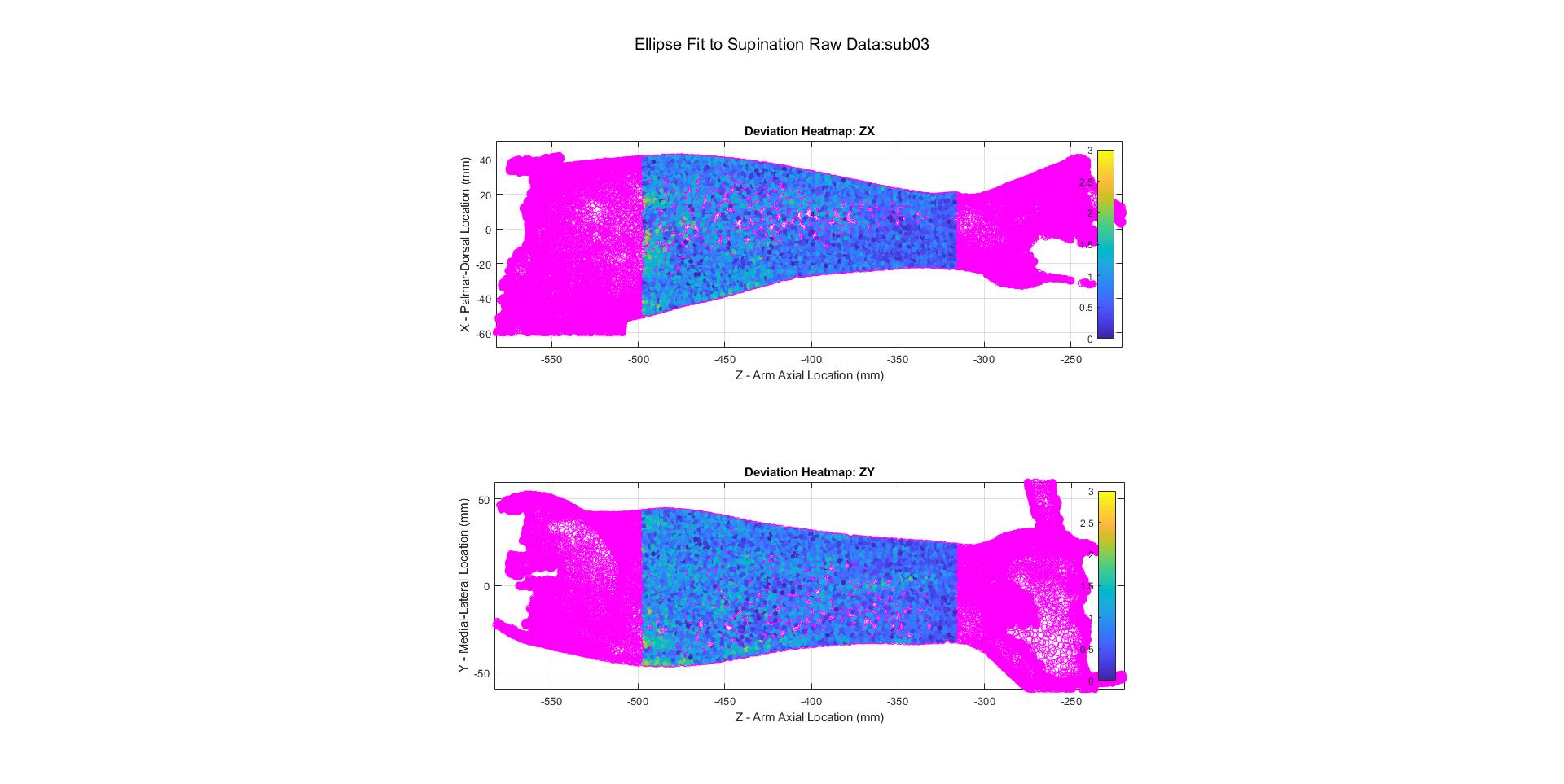

Supplement: Supplementary file 1 [file Data_Sheet_1.ZIP › SF5.3_Figure7_sub03_sup.jpg]

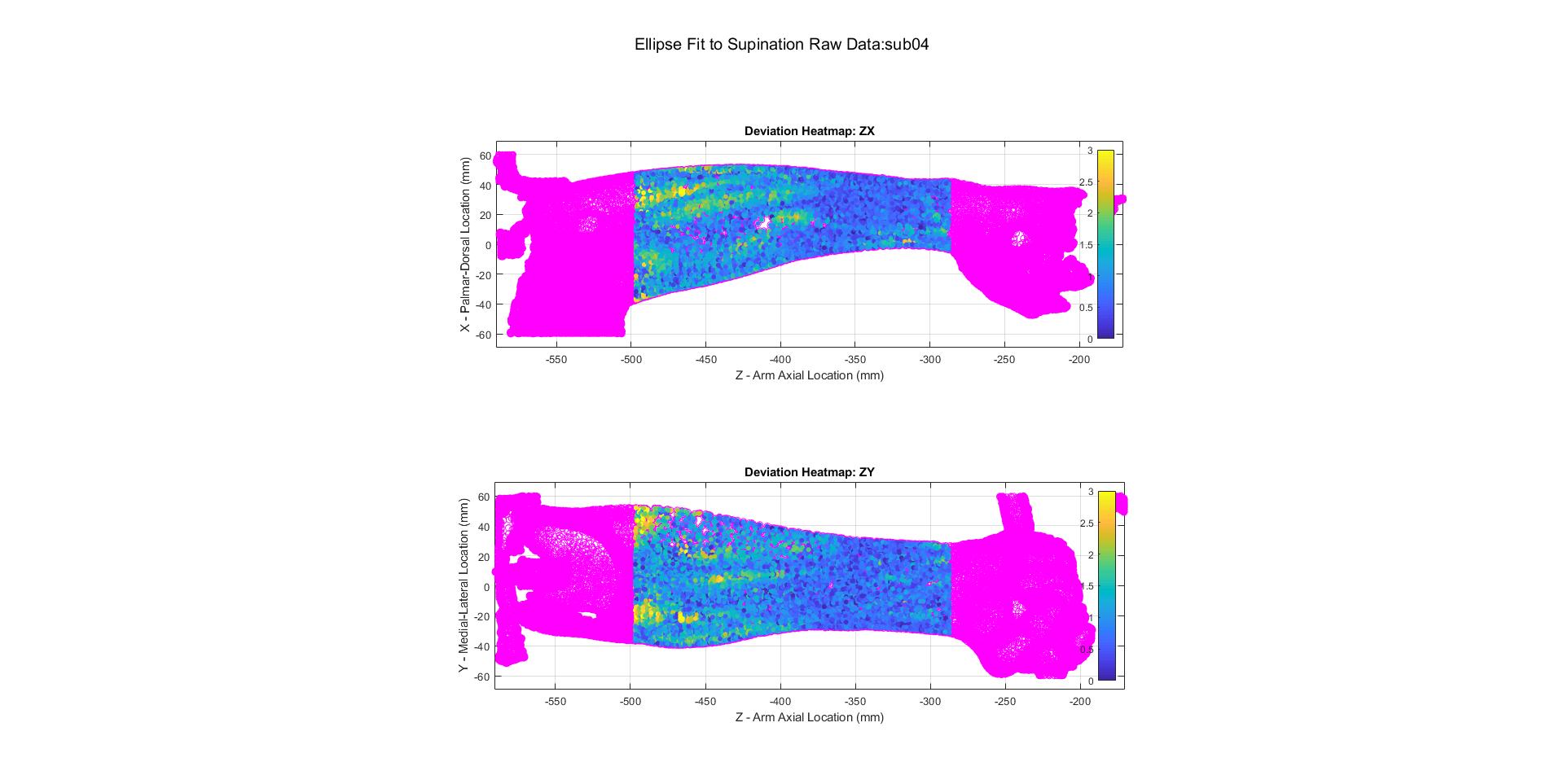

Supplement: Supplementary file 1 [file Data_Sheet_1.ZIP › SF5.4_Figure7_sub04_sup.jpg]

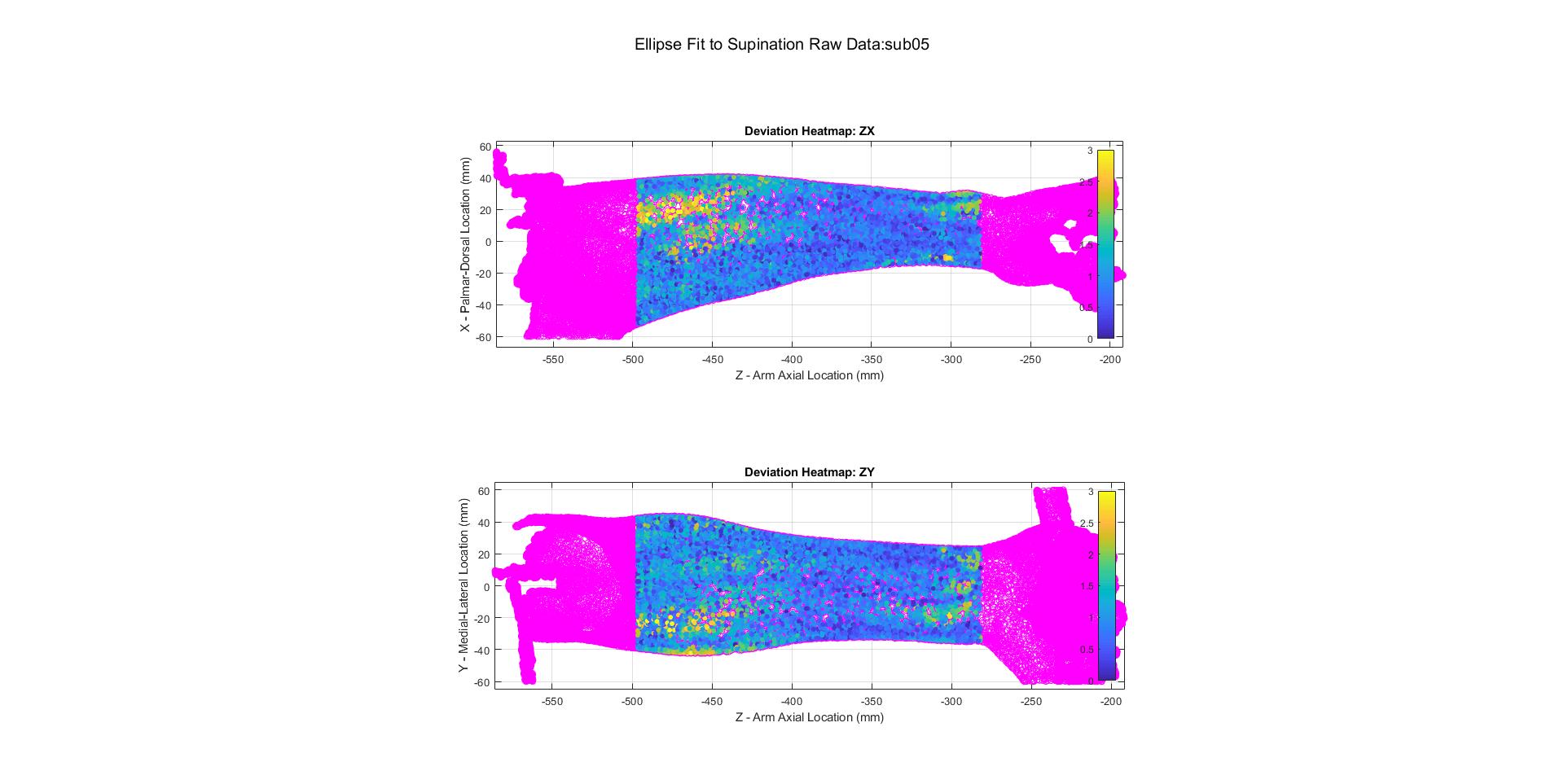

Supplement: Supplementary file 1 [file Data_Sheet_1.ZIP › SF5.5_Figure7_sub05_sup.jpg]

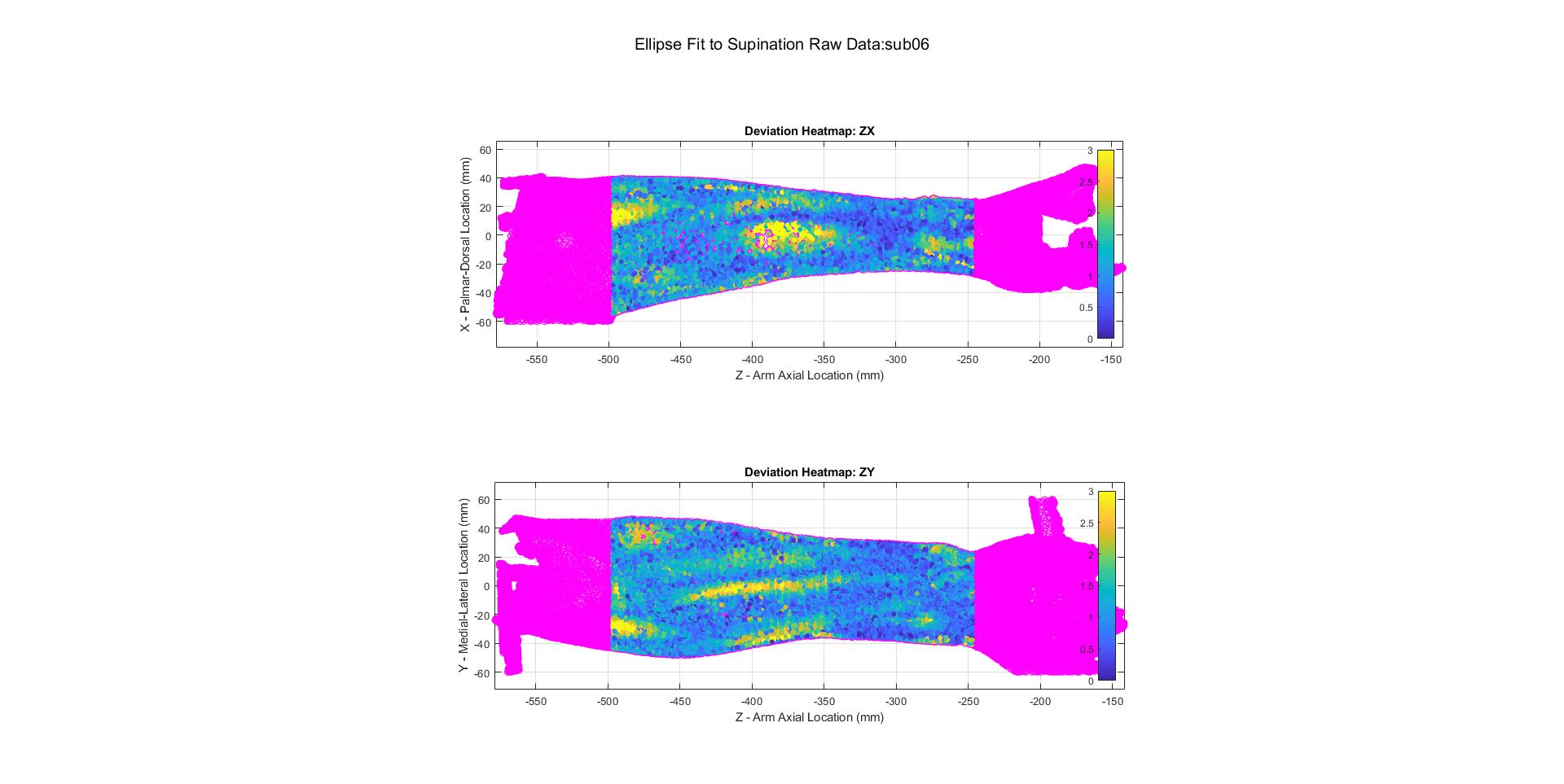

Supplement: Supplementary file 1 [file Data_Sheet_1.ZIP › SF5.6_Figure7_sub06_sup.jpg]

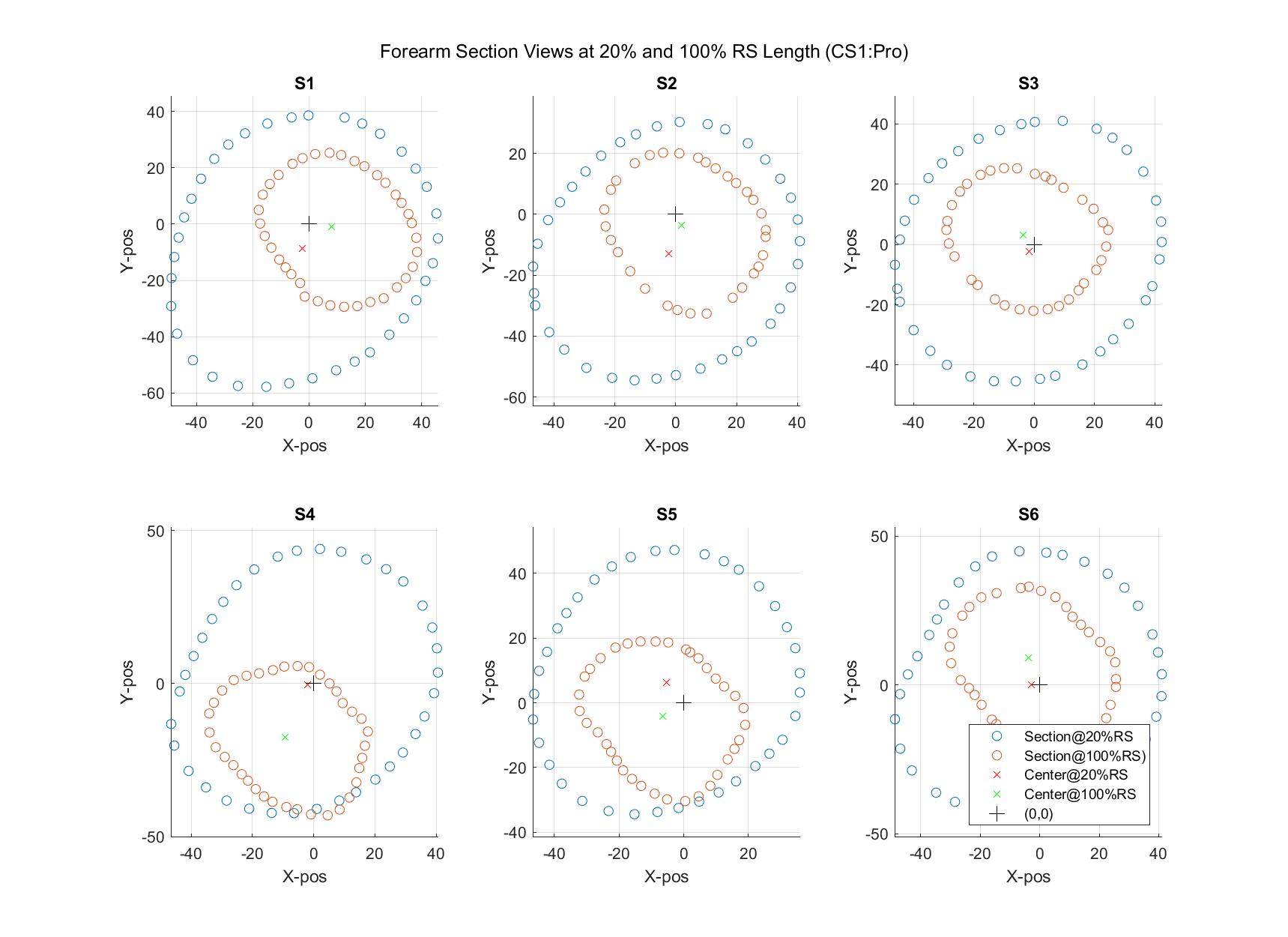

Supplement: Supplementary file 1 [file Data_Sheet_1.ZIP › SF6_AxialAlignment_at20and100%RS_CS1_Pro.jpg]

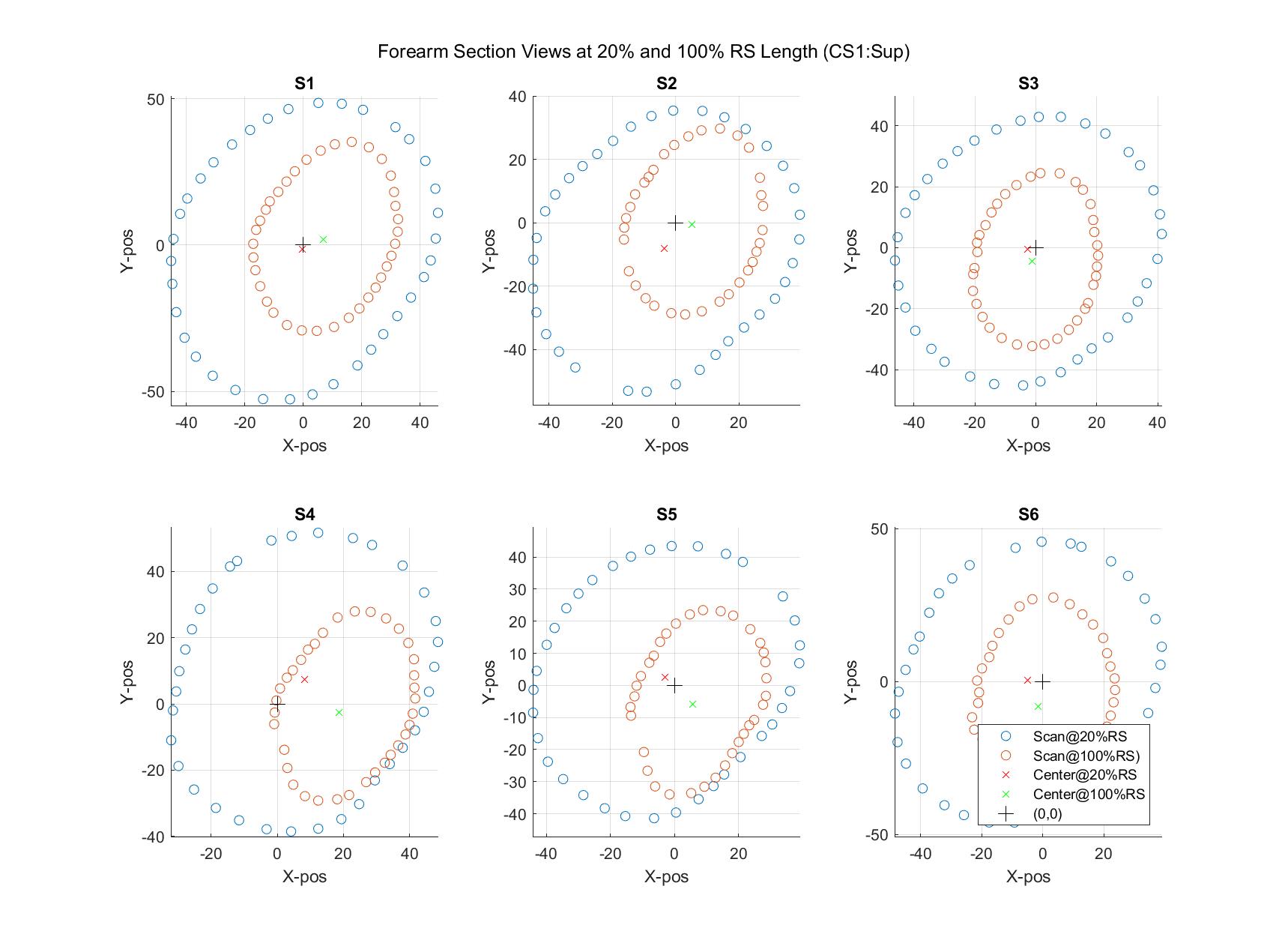

Supplement: Supplementary file 1 [file Data_Sheet_1.ZIP › SF7_AxialAlignment_at20and100%RS_CS1_Sup.jpg]

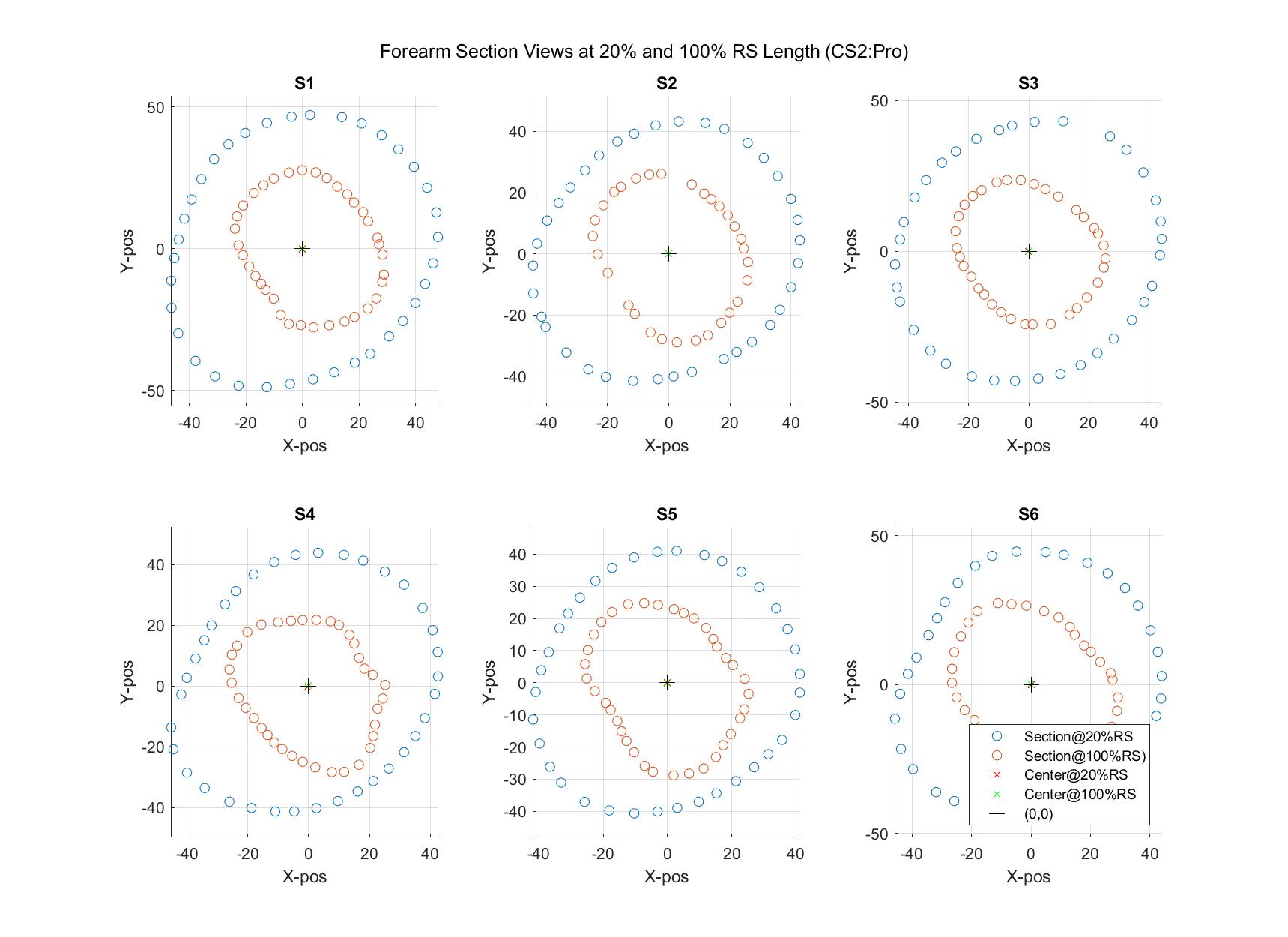

Supplement: Supplementary file 1 [file Data_Sheet_1.ZIP › SF8_AxialAlignment_at20and100%RS_CS2_Pro.jpg]

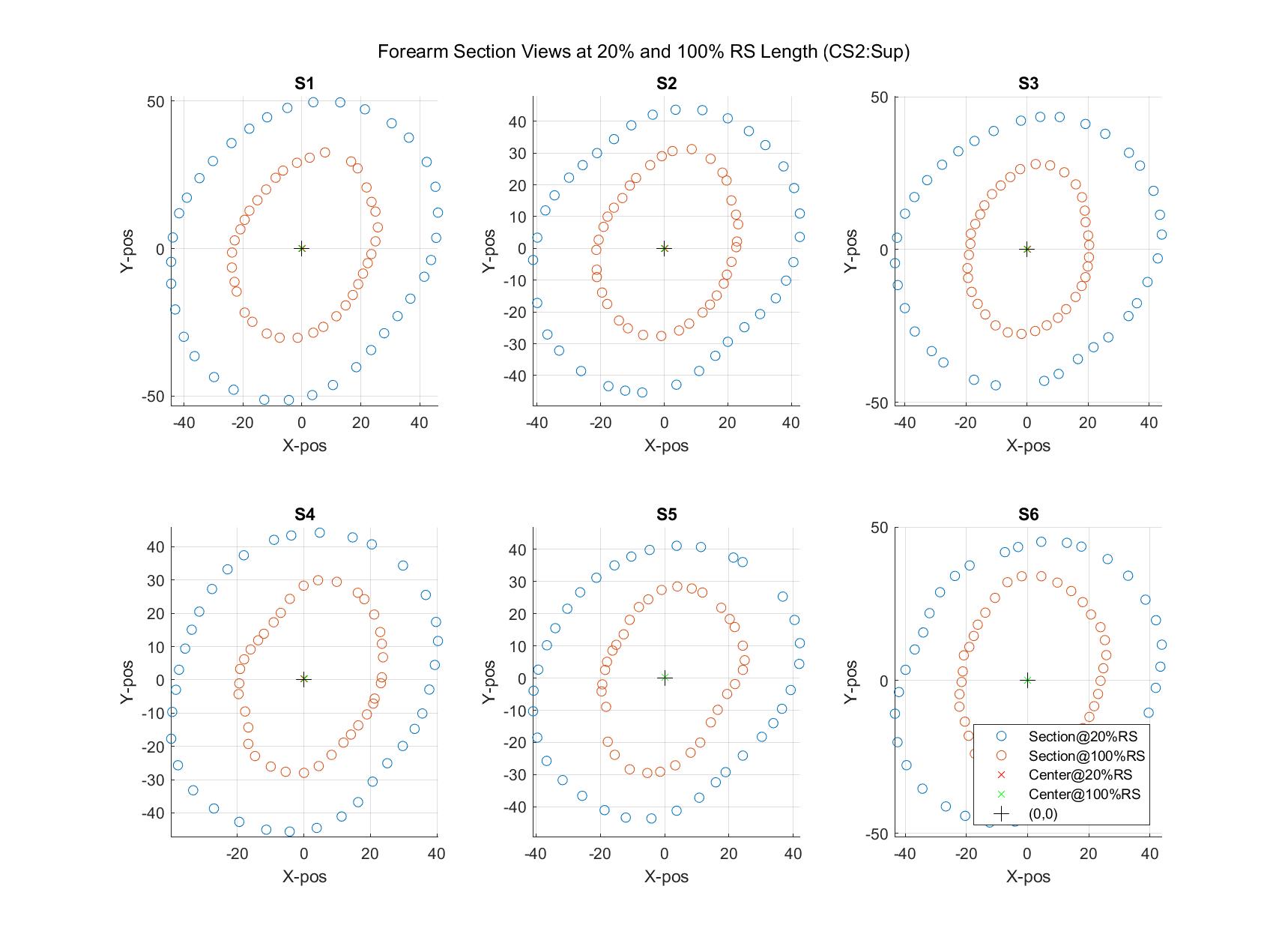

Supplement: Supplementary file 1 [file Data_Sheet_1.ZIP › SF9_AxialAlignment_at20and100%RS_CS2_Sup.jpg]
