# Supplementary figures and images for: 3D Scanning of the Forearm for Orthosis and HMI Applications (part 1 of 2)
Source: Front Robot AI. 2021 Apr 14;8:576783. doi: 10.3389/frobt.2021.576783 (PMC8079810; doi:10.3389/frobt.2021.576783)

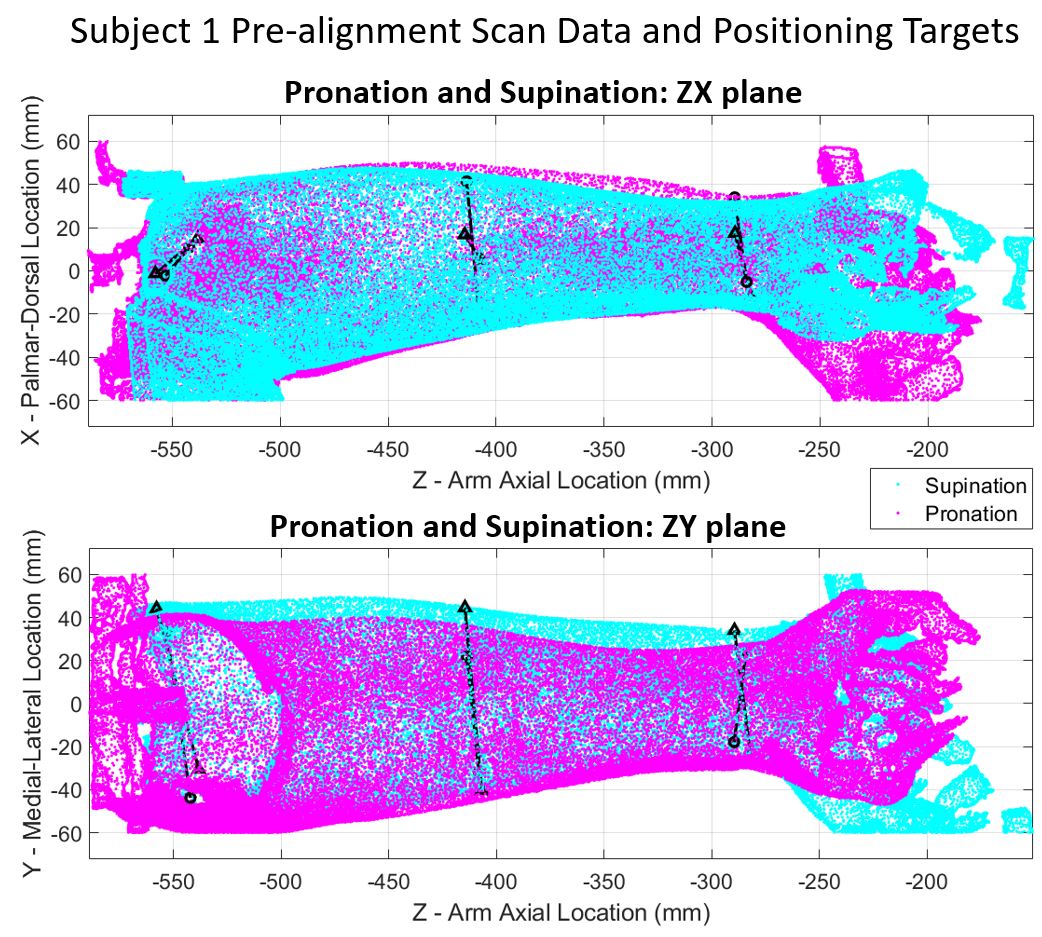

Supplement: Supplementary file 1 [file Data_Sheet_1.ZIP › SF1.1_Figure6A_RawScanAndTargets_S1.JPG]

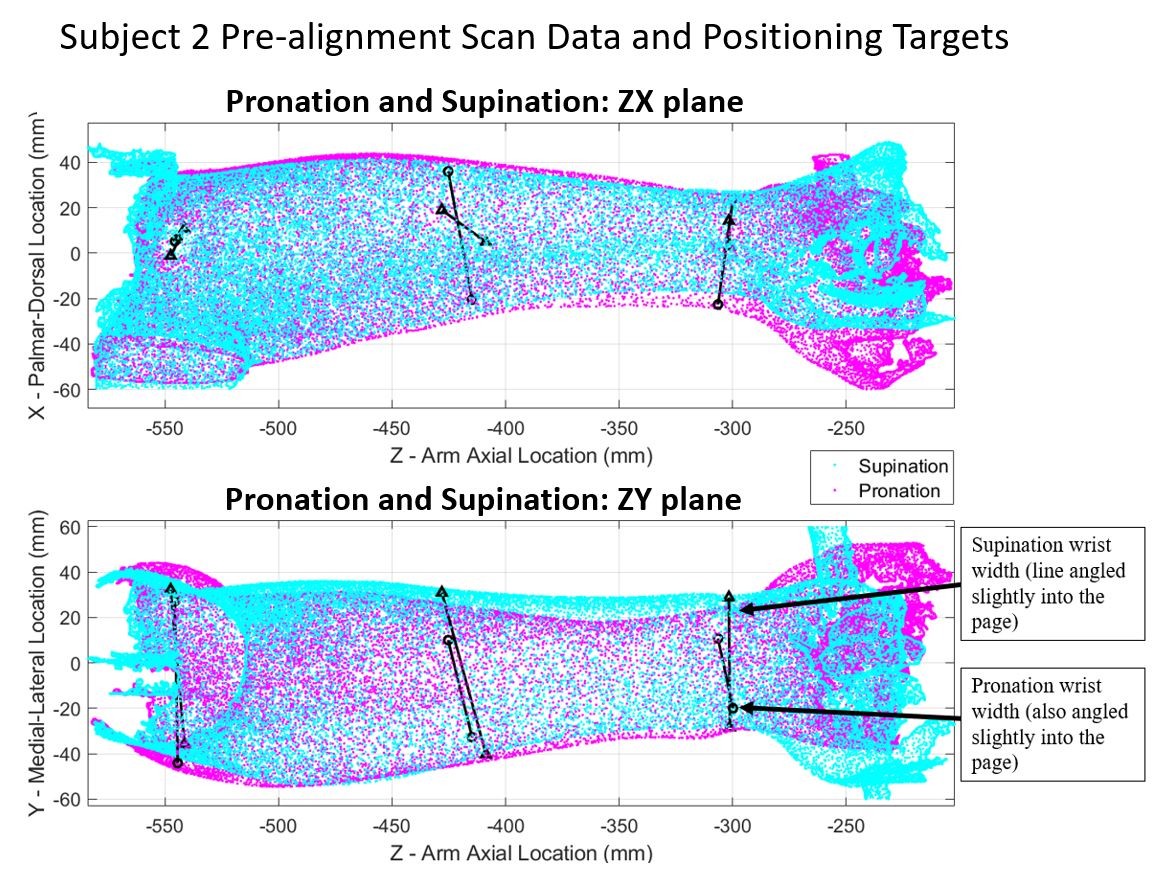

Supplement: Supplementary file 1 [file Data_Sheet_1.ZIP › SF1.2_Figure6A_RawScanAndTargets_S2.JPG]

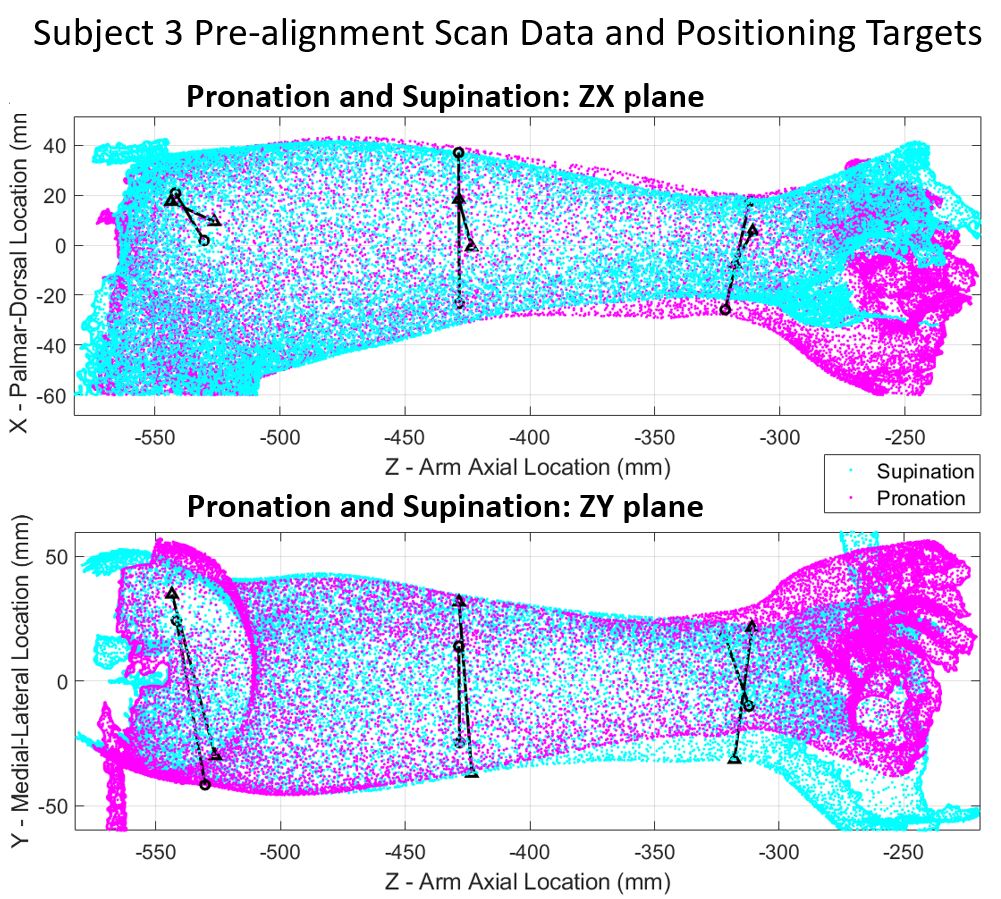

Supplement: Supplementary file 1 [file Data_Sheet_1.ZIP › SF1.3_Figure6A_RawScanAndTargets_S3.JPG]

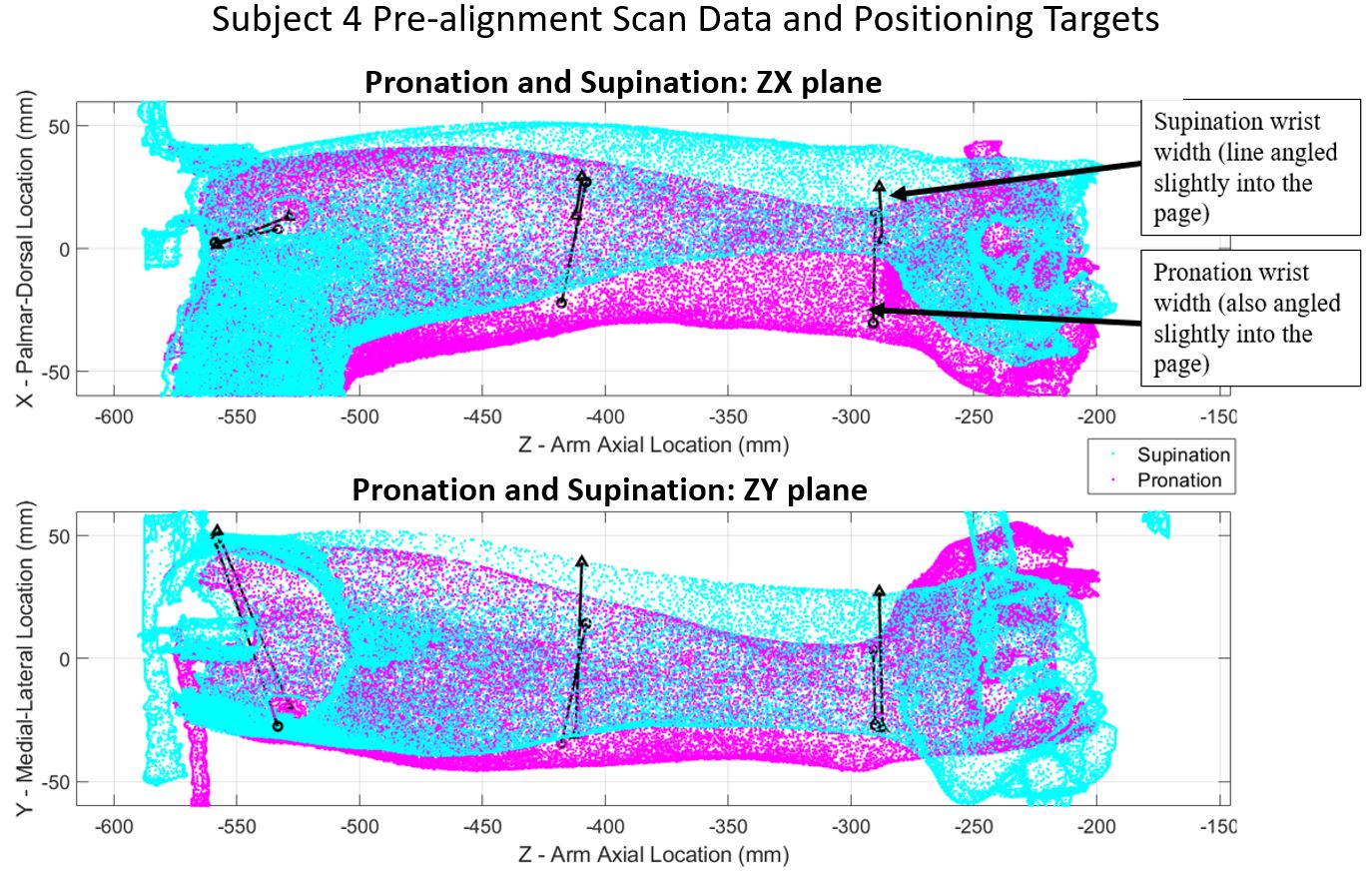

Supplement: Supplementary file 1 [file Data_Sheet_1.ZIP › SF1.4_Figure6A_RawScanAndTargets_S4.JPG]

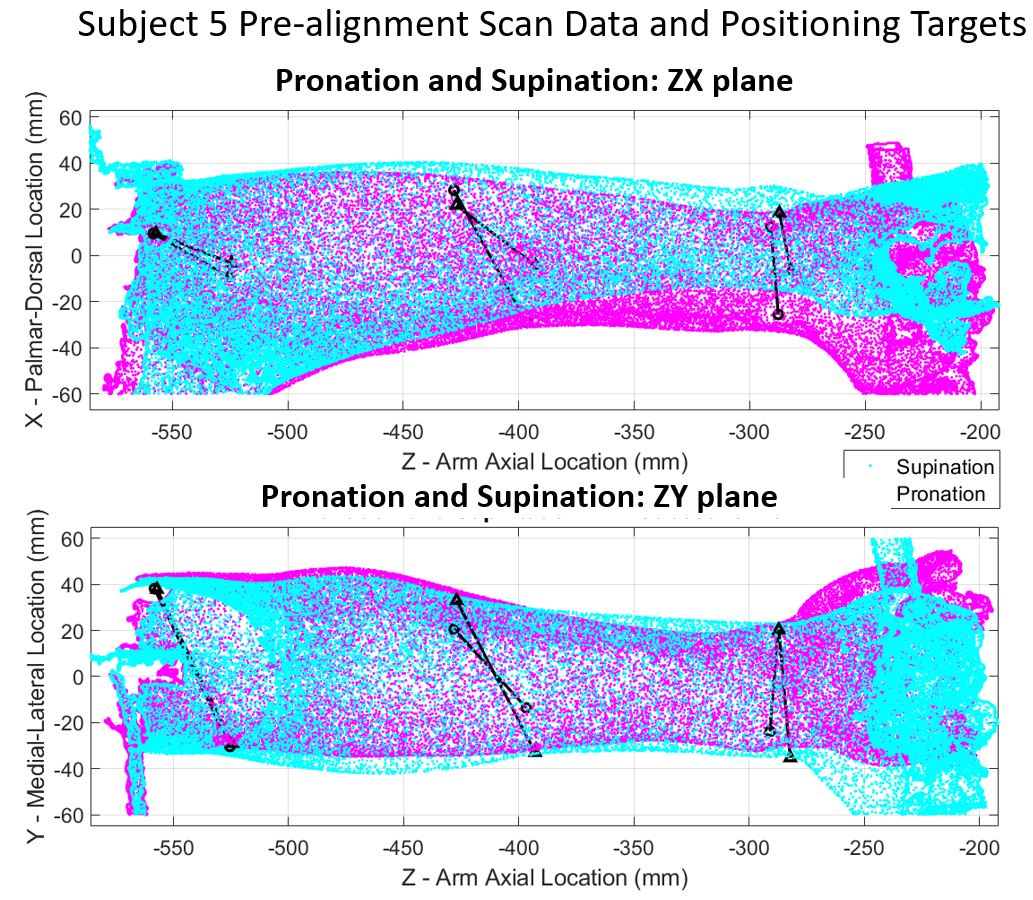

Supplement: Supplementary file 1 [file Data_Sheet_1.ZIP › SF1.5_Figure6A_RawScanAndTargets_S5.JPG]

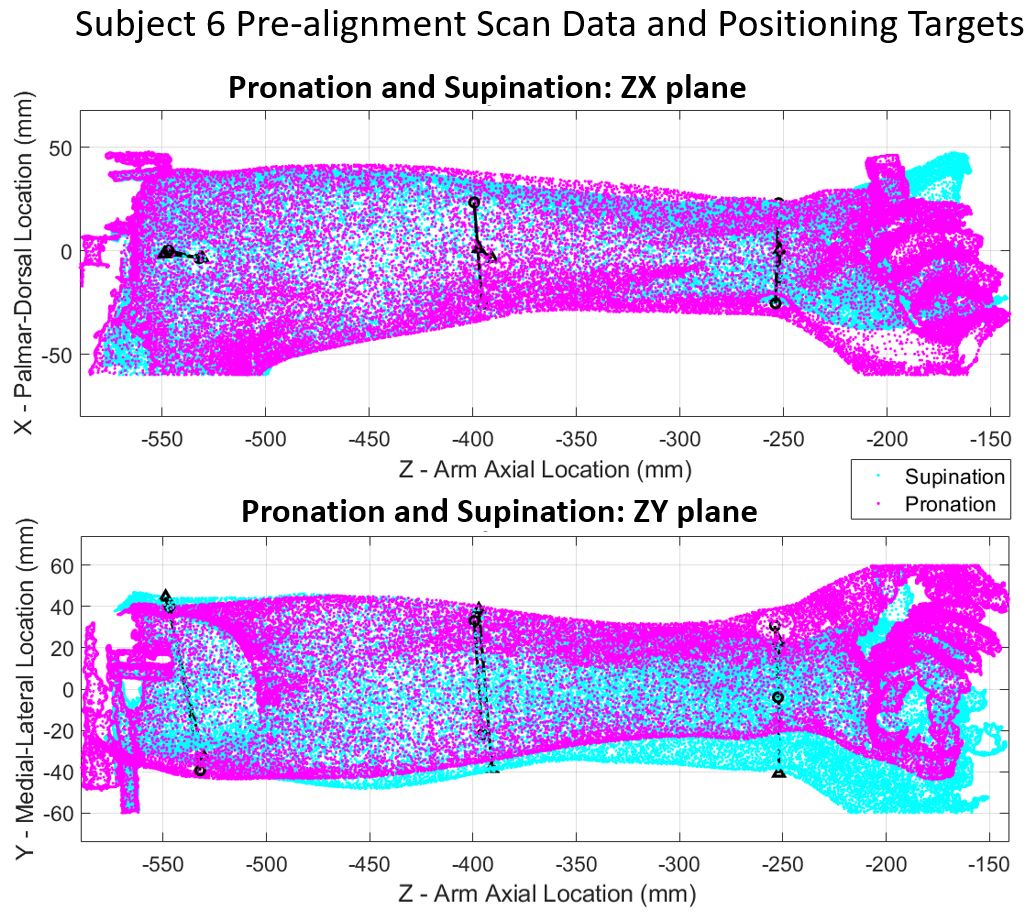

Supplement: Supplementary file 1 [file Data_Sheet_1.ZIP › SF1.6_Figure6A_RawScanAndTargets_S6.JPG]

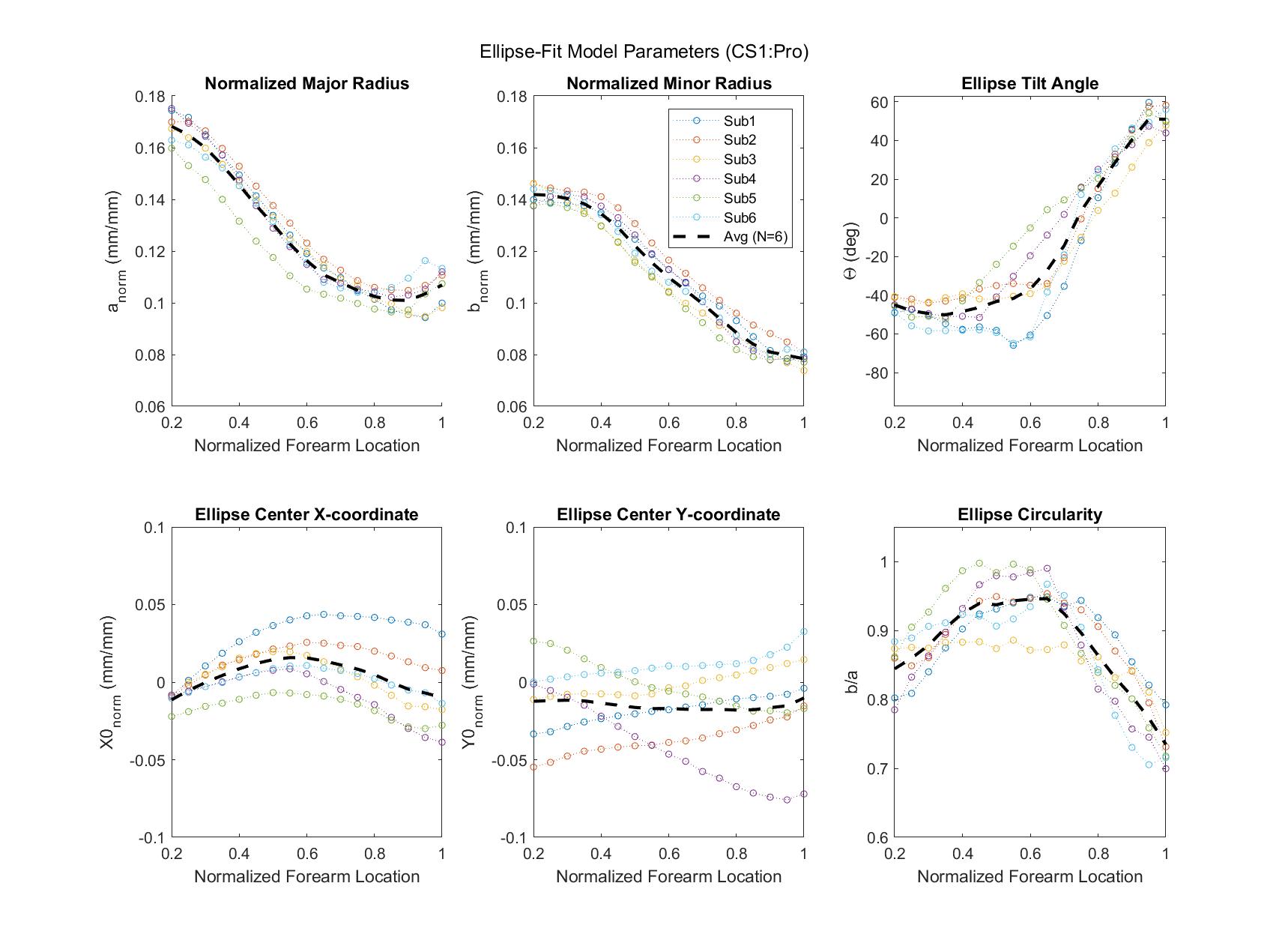

Supplement: Supplementary file 1 [file Data_Sheet_1.ZIP › SF10_EllipseParameters_CS1_Pro_avg.jpg]

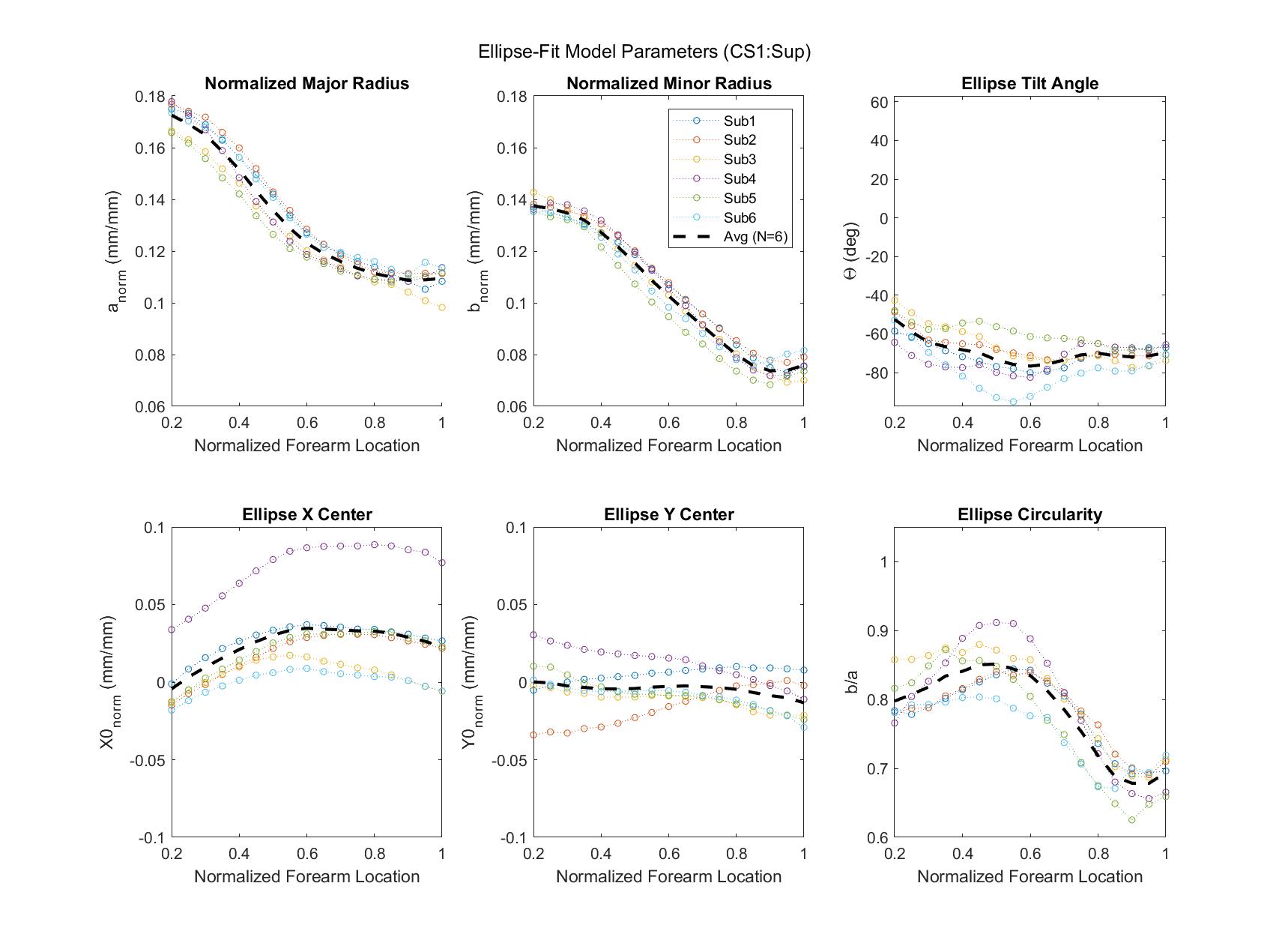

Supplement: Supplementary file 1 [file Data_Sheet_1.ZIP › SF11_EllipseParameters_CS1_Sup_avg.jpg]

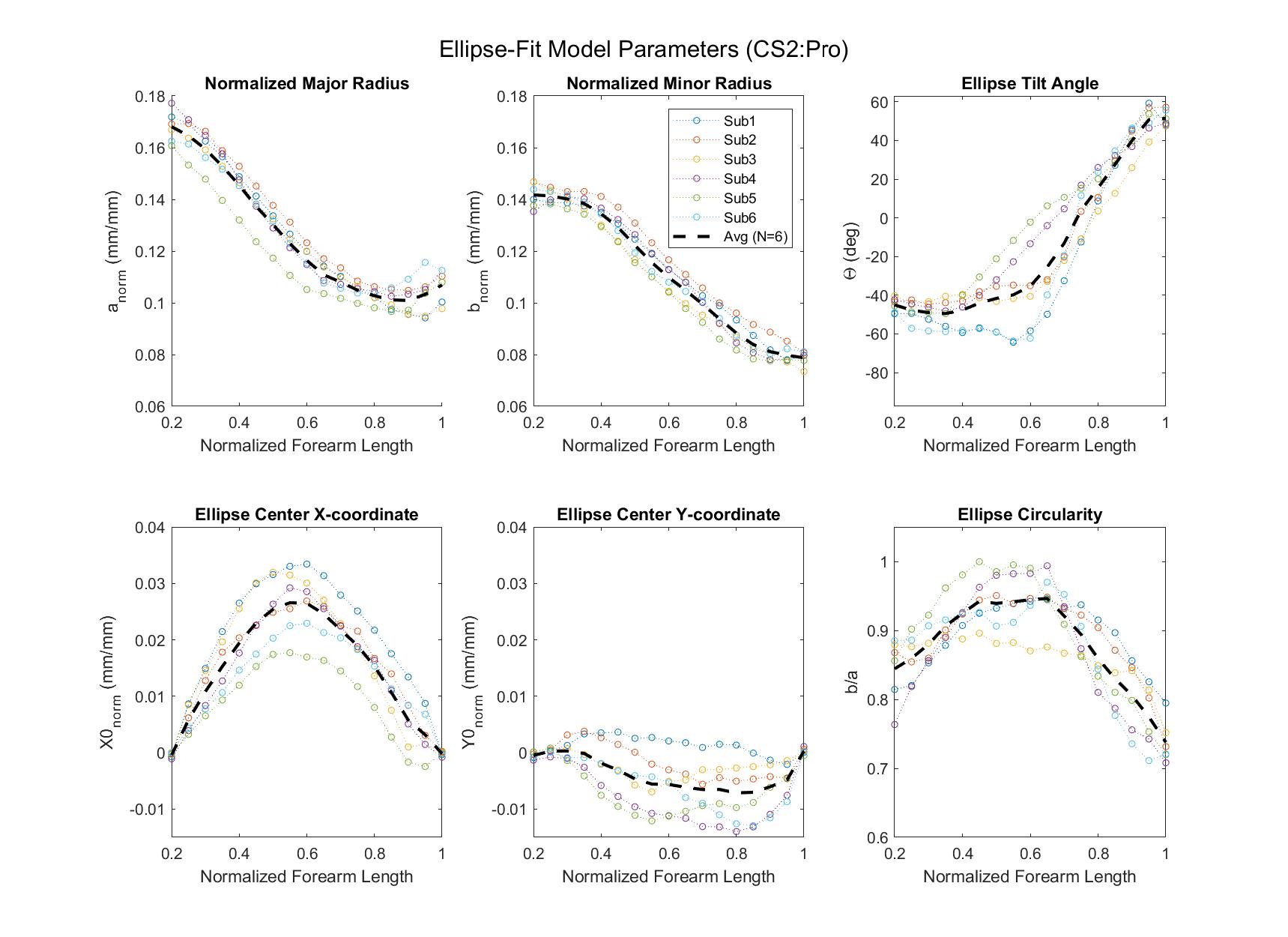

Supplement: Supplementary file 1 [file Data_Sheet_1.ZIP › SF12_EllipseParameters_CS2_Pro_avg.jpg]

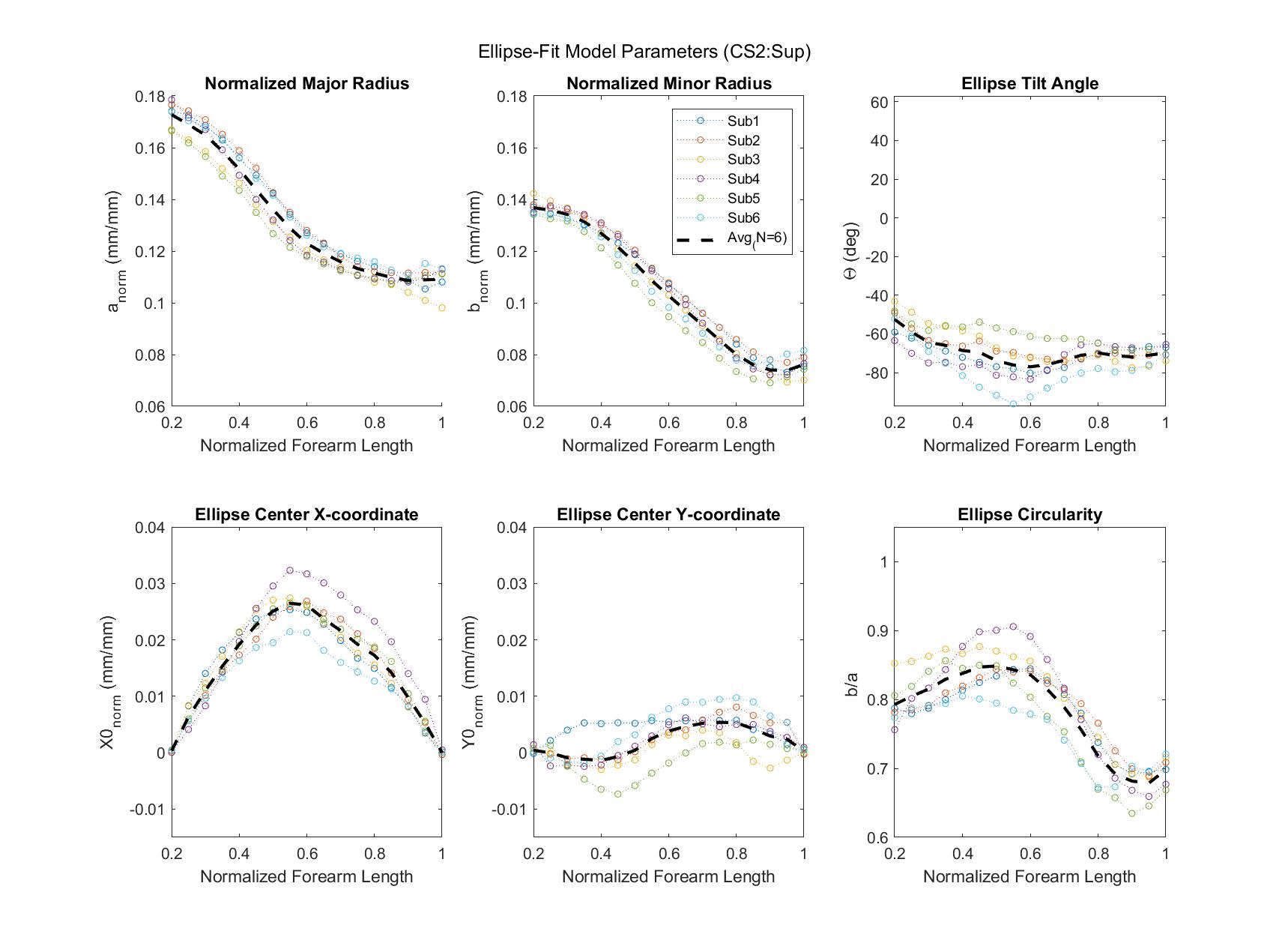

Supplement: Supplementary file 1 [file Data_Sheet_1.ZIP › SF13_EllipseParameters_CS2_Sup_avg.jpg]

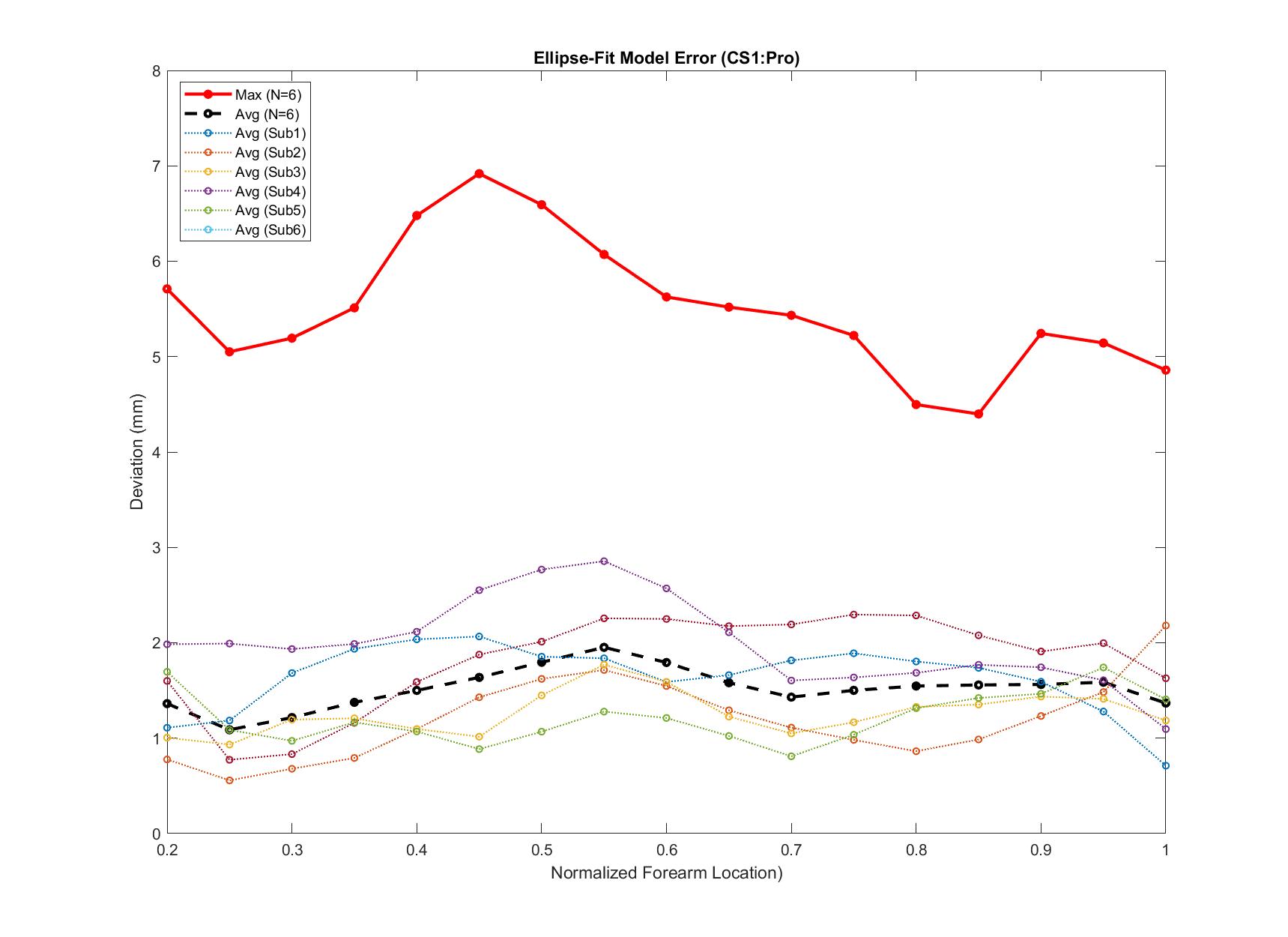

Supplement: Supplementary file 1 [file Data_Sheet_1.ZIP › SF14_ModelError_CS1_Pro.jpg]

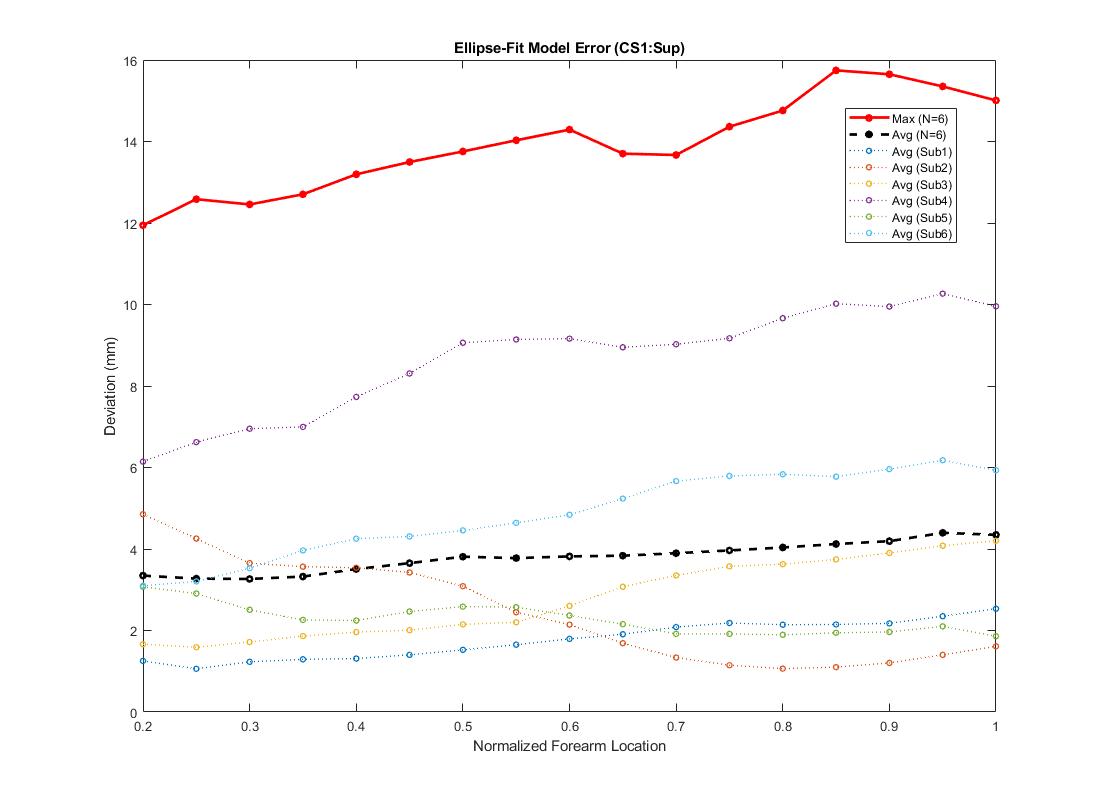

Supplement: Supplementary file 1 [file Data_Sheet_1.ZIP › SF15_ModelError_CS1_Sup.jpg]

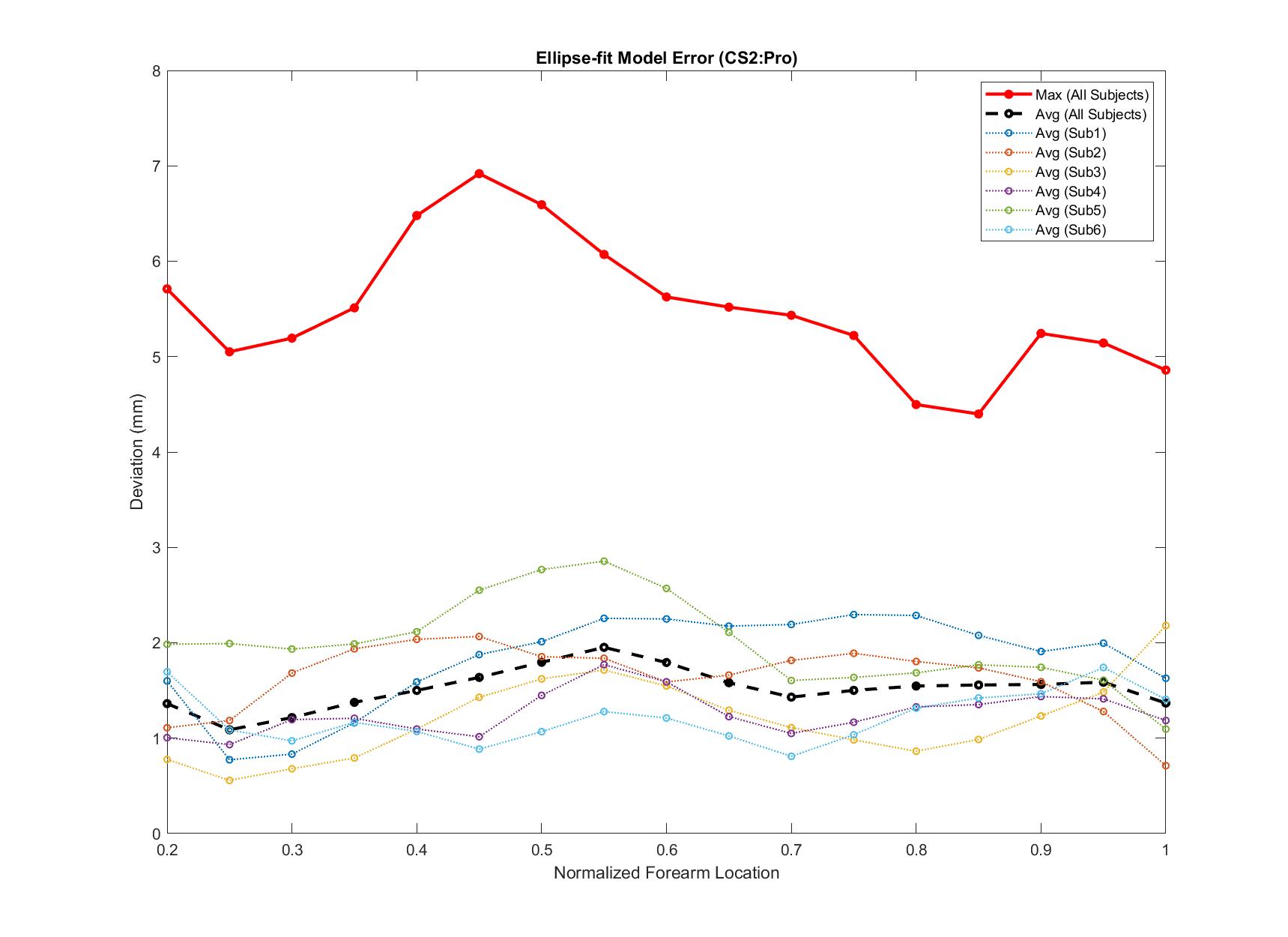

Supplement: Supplementary file 1 [file Data_Sheet_1.ZIP › SF16_ModelError_CS2_Pro.jpg]

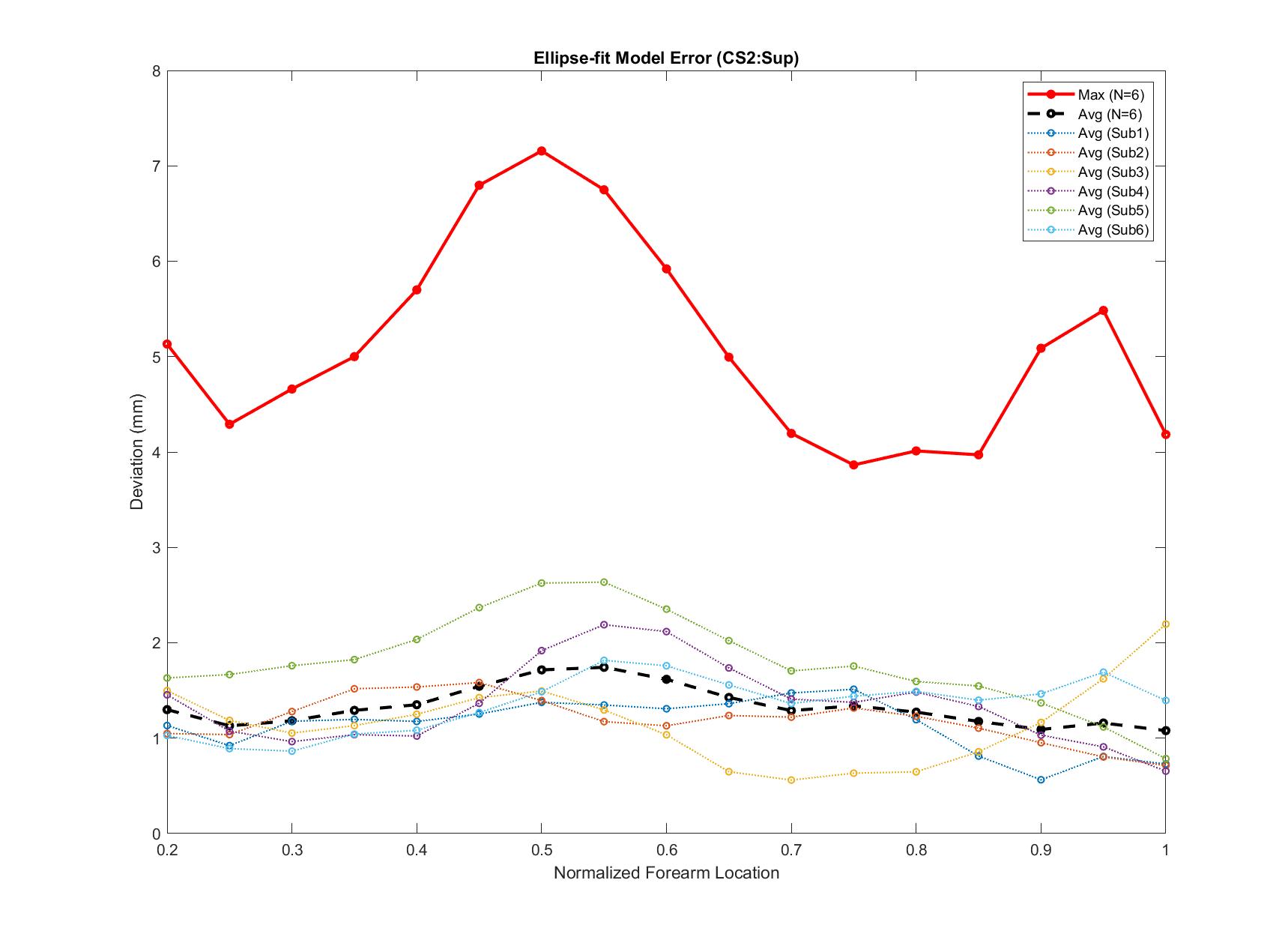

Supplement: Supplementary file 1 [file Data_Sheet_1.ZIP › SF17_ModelError_CS2_Sup.jpg]

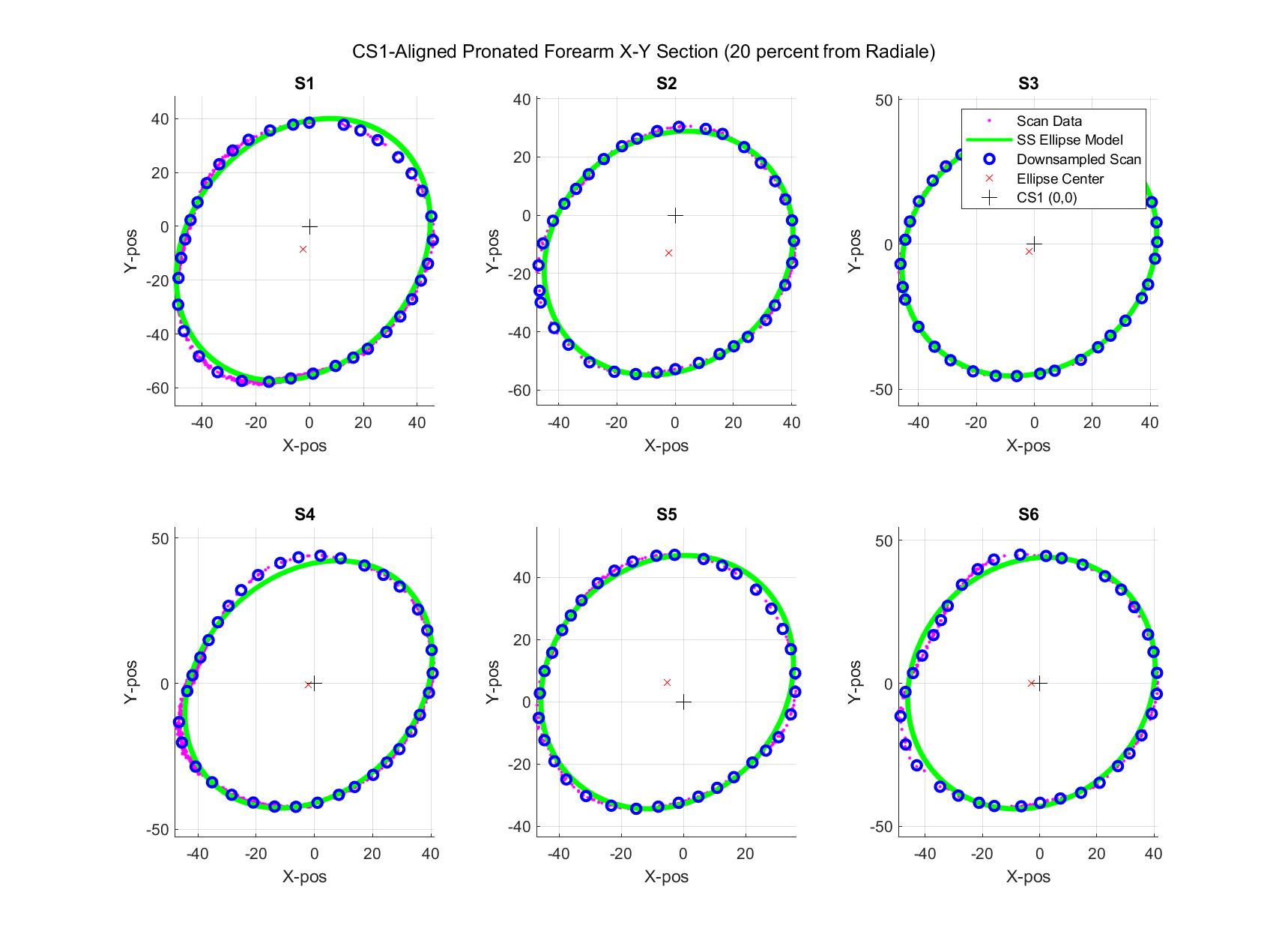

Supplement: Supplementary file 1 [file Data_Sheet_1.ZIP › SF18.1_SS_CS1_Downsampled_Cross-sectional Ellipse-Fit_20%RS_Pro.jpg]

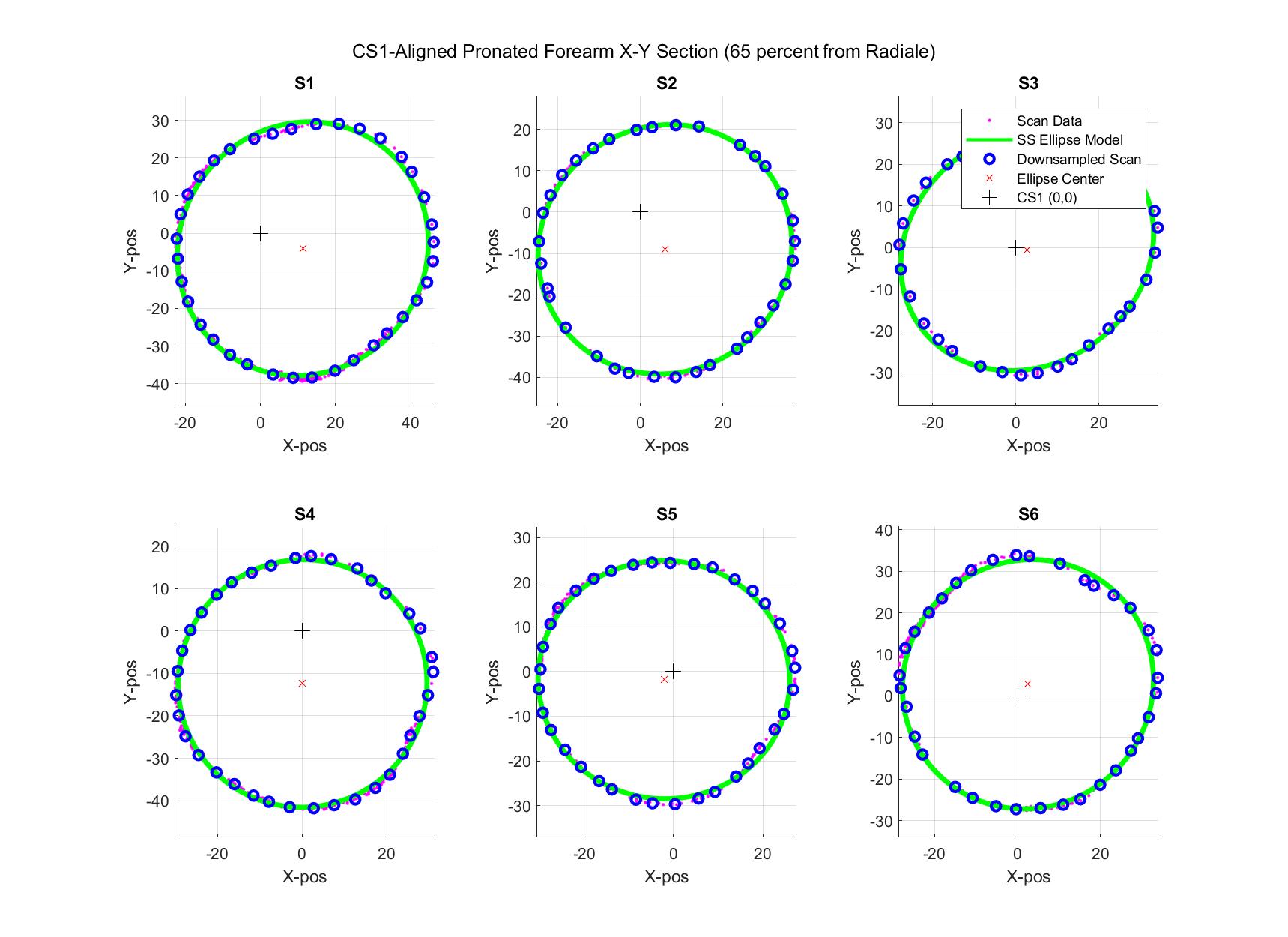

Supplement: Supplementary file 1 [file Data_Sheet_1.ZIP › SF18.10_SS_CS1_Downsampled_Cross-sectional Ellipse-Fit_65%RS_Pro.jpg]

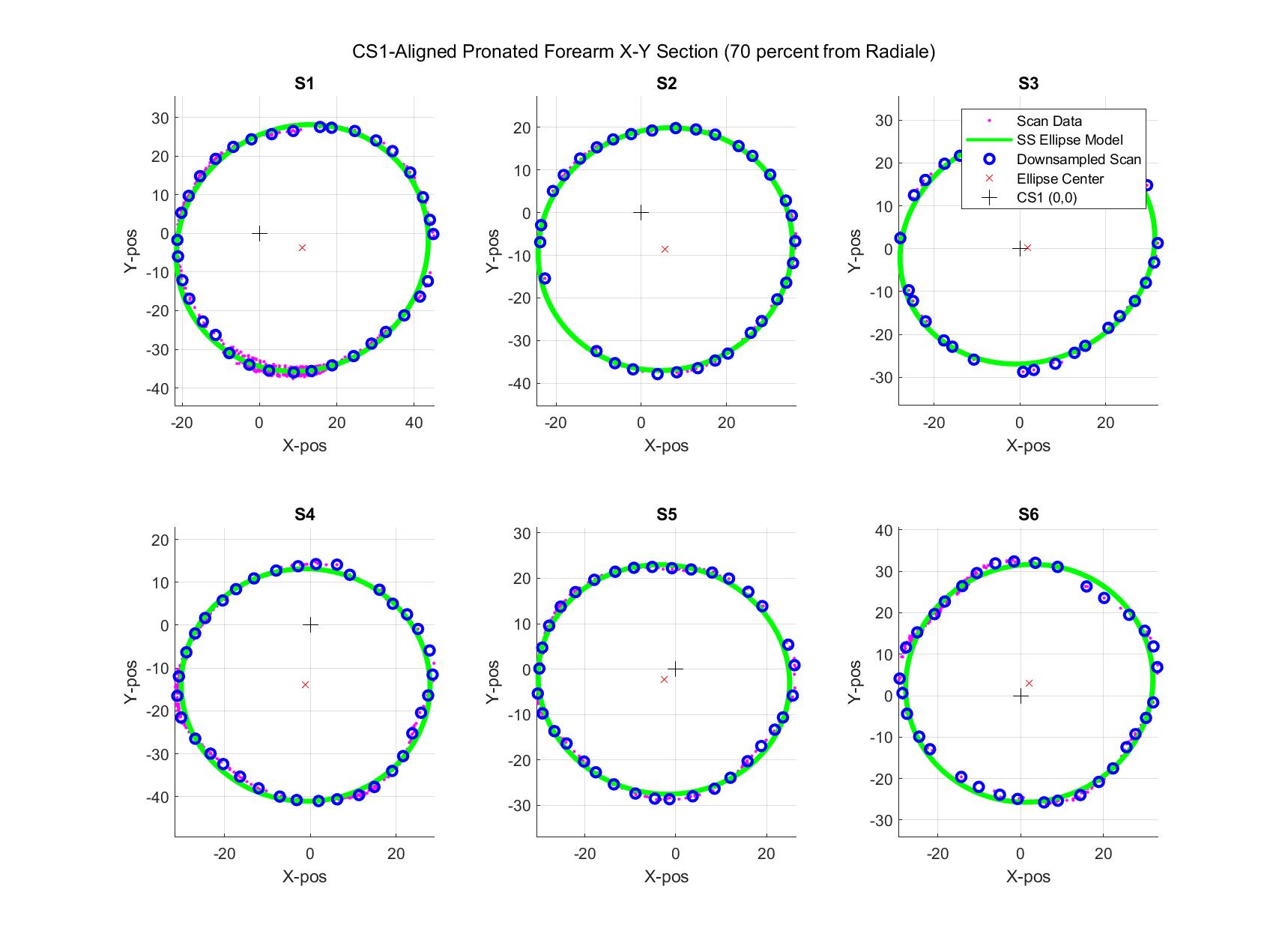

Supplement: Supplementary file 1 [file Data_Sheet_1.ZIP › SF18.11_SS_CS1_Downsampled_Cross-sectional Ellipse-Fit_70%RS_Pro.jpg]

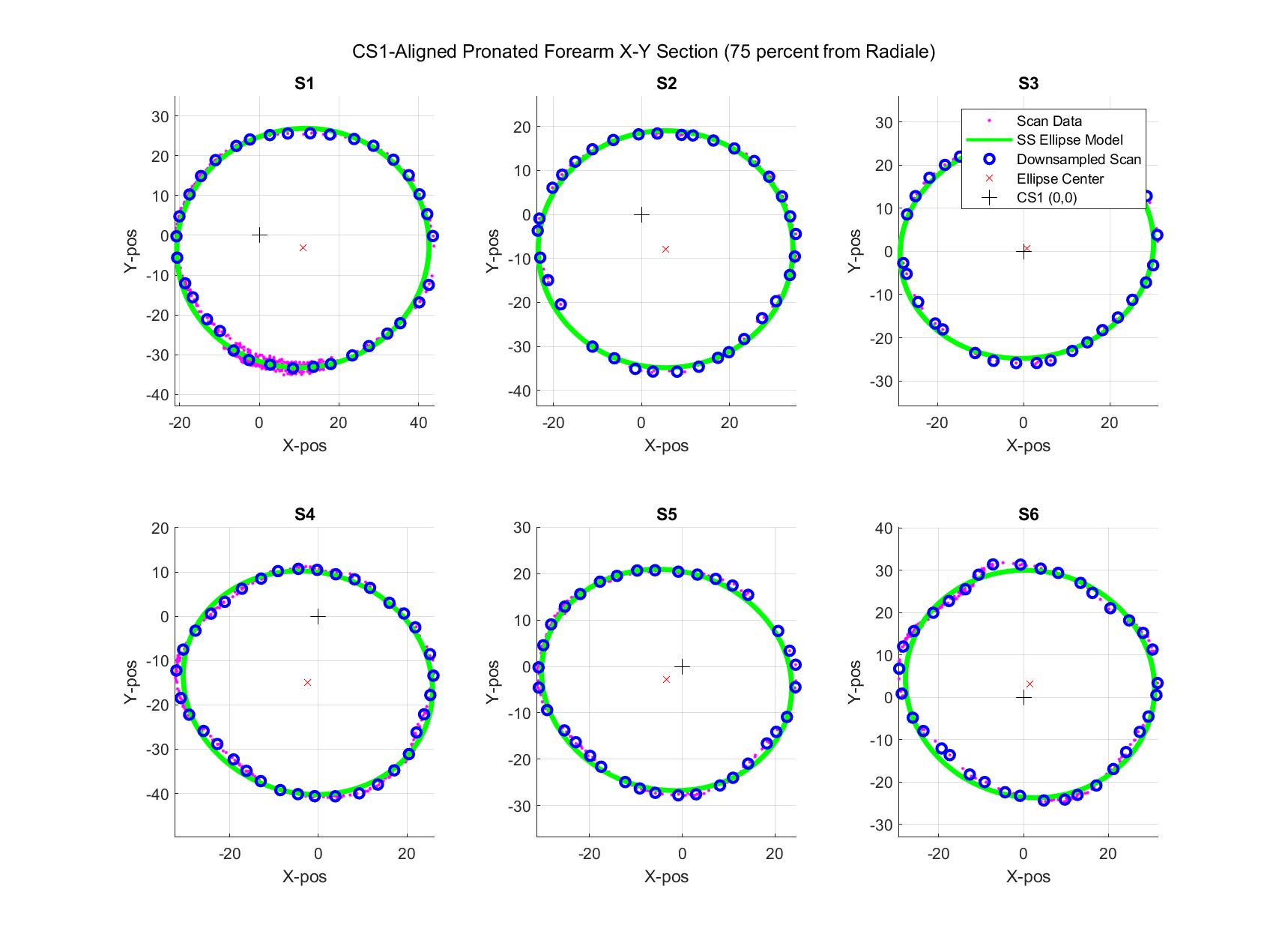

Supplement: Supplementary file 1 [file Data_Sheet_1.ZIP › SF18.12_SS_CS1_Downsampled_Cross-sectional Ellipse-Fit_75%RS_Pro.jpg]

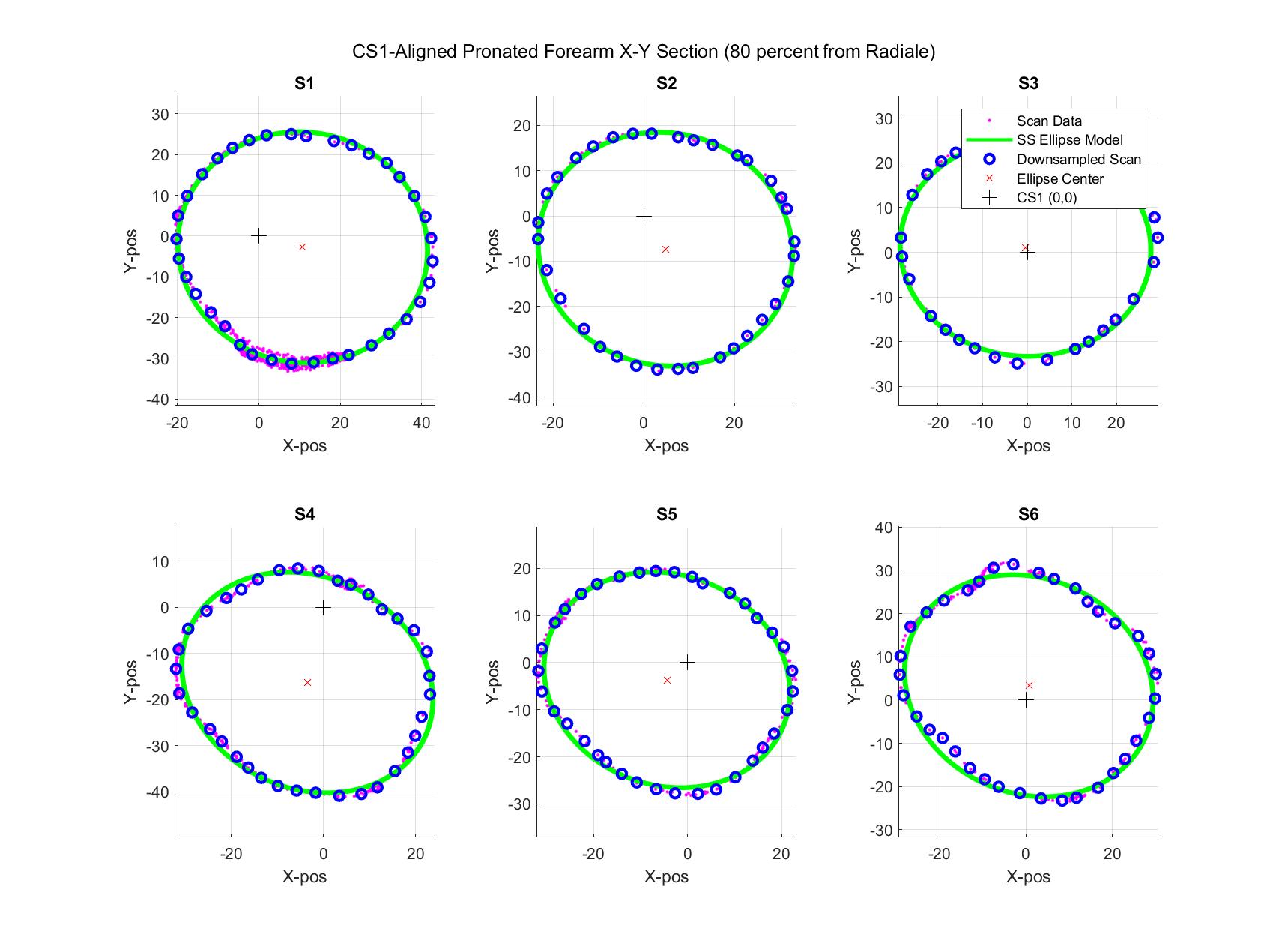

Supplement: Supplementary file 1 [file Data_Sheet_1.ZIP › SF18.13_SS_CS1_Downsampled_Cross-sectional Ellipse-Fit_80%RS_Pro.jpg]

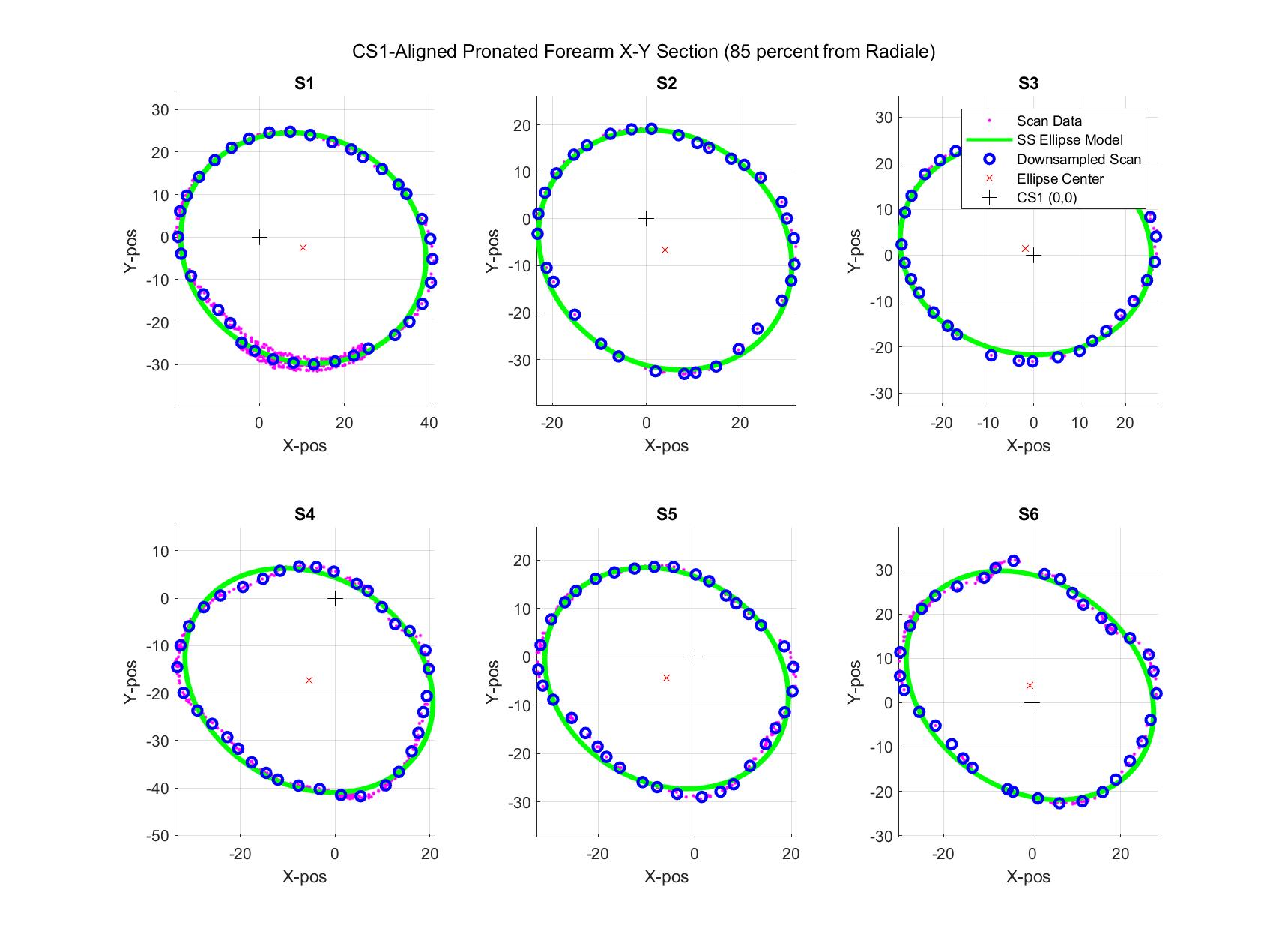

Supplement: Supplementary file 1 [file Data_Sheet_1.ZIP › SF18.14_SS_CS1_Downsampled_Cross-sectional Ellipse-Fit_85%RS_Pro.jpg]

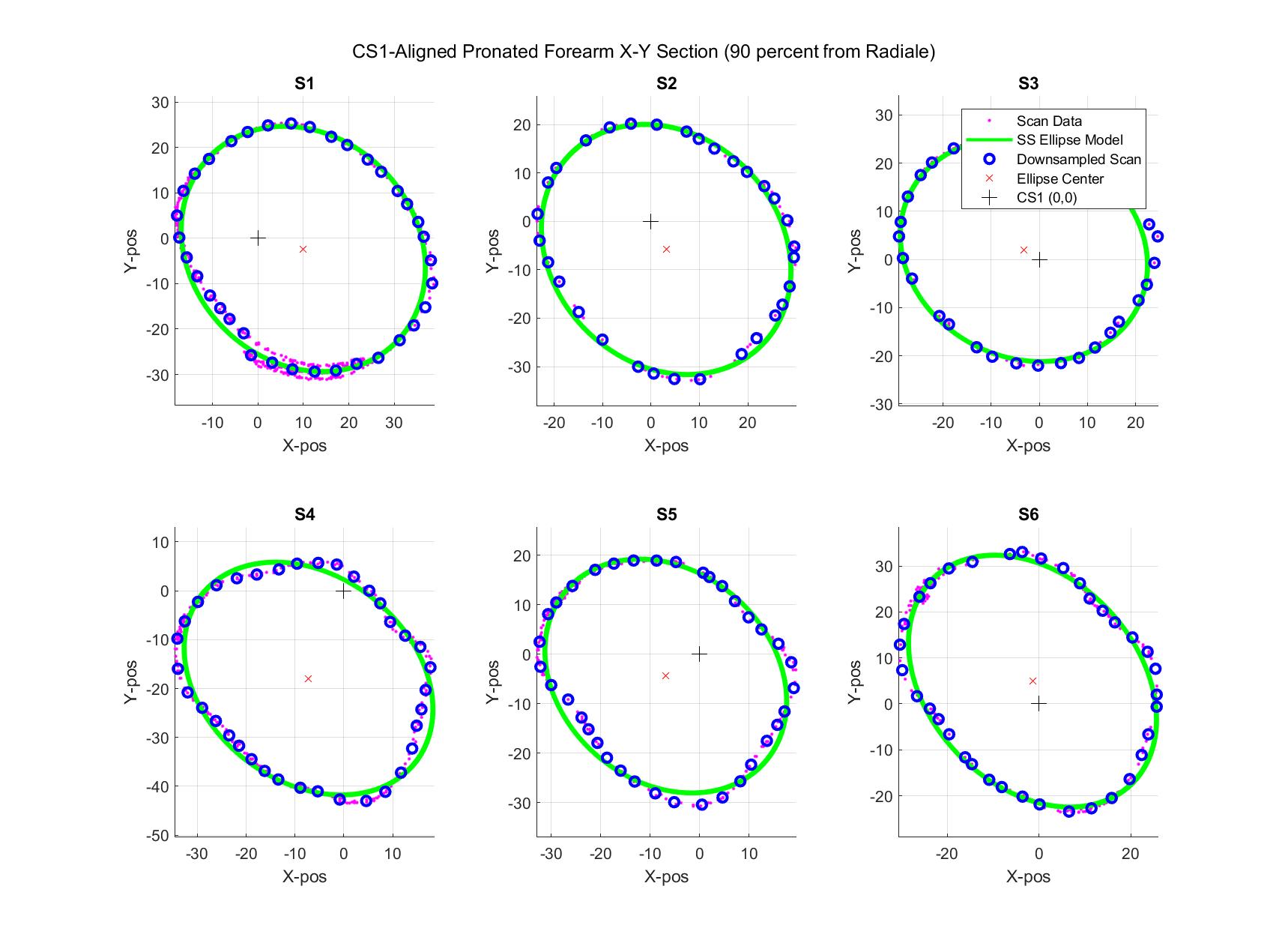

Supplement: Supplementary file 1 [file Data_Sheet_1.ZIP › SF18.15_SS_CS1_Downsampled_Cross-sectional Ellipse-Fit_90%RS_Pro.jpg]

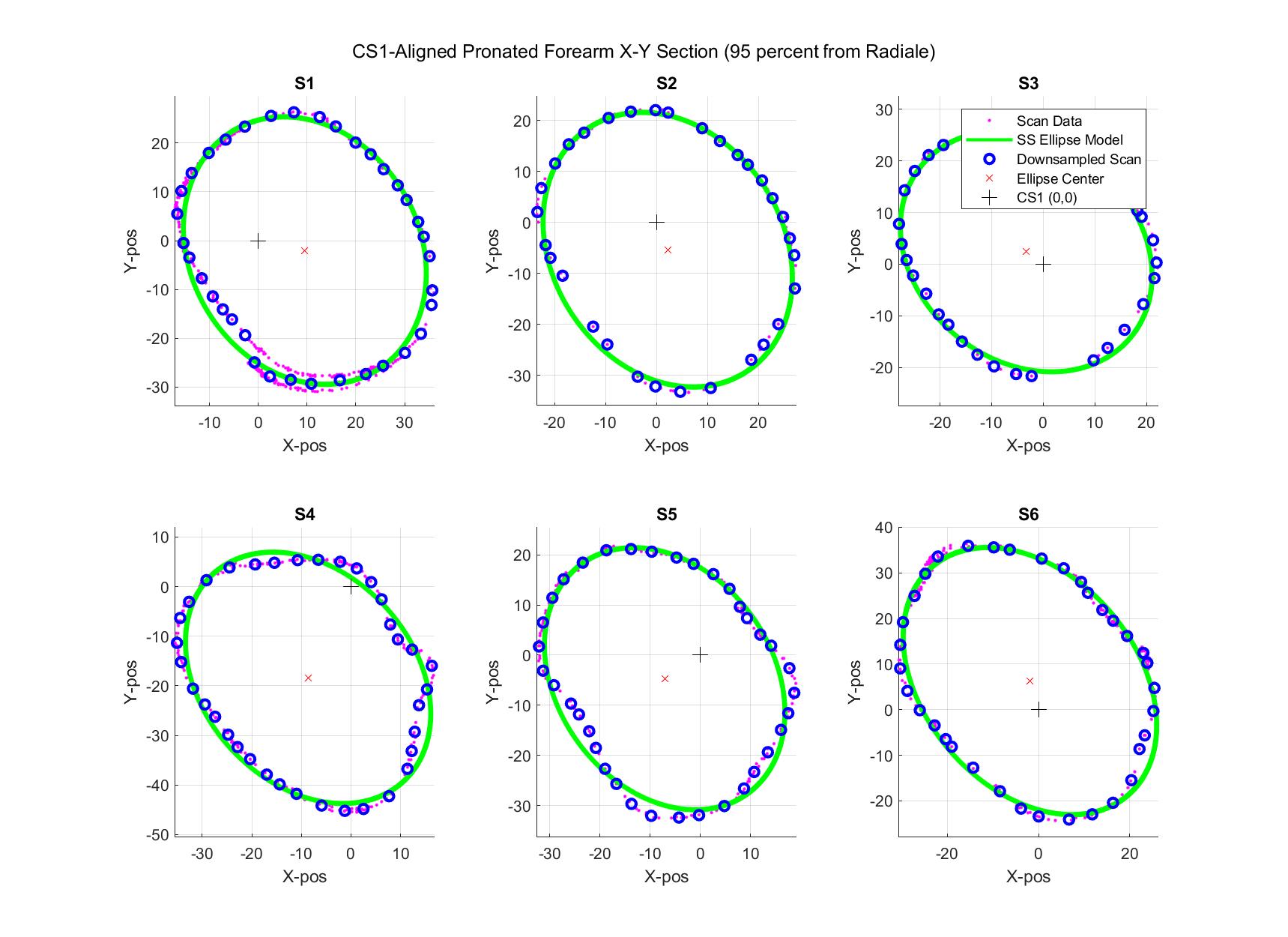

Supplement: Supplementary file 1 [file Data_Sheet_1.ZIP › SF18.16_SS_CS1_Downsampled_Cross-sectional Ellipse-Fit_95%RS_Pro.jpg]

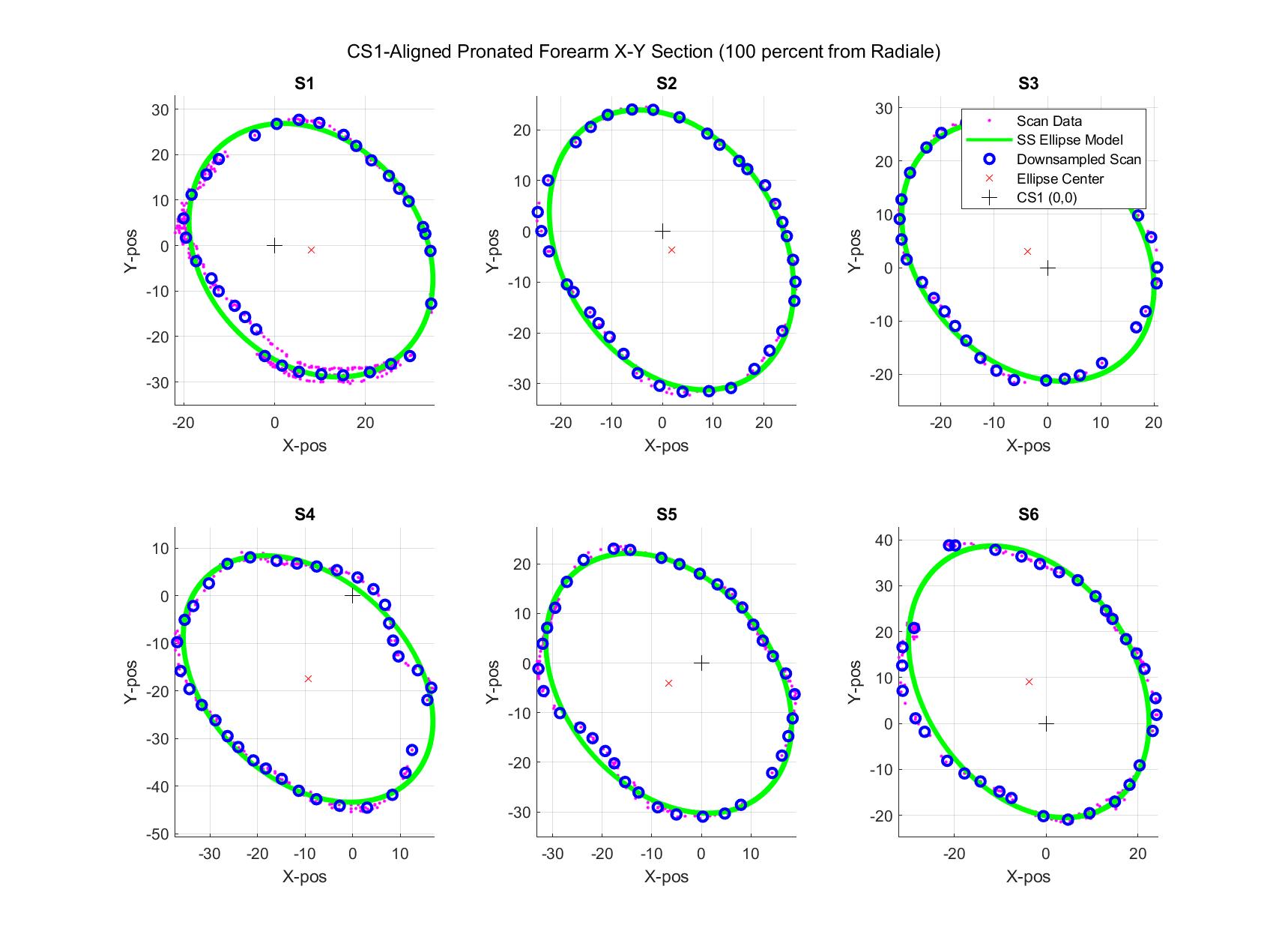

Supplement: Supplementary file 1 [file Data_Sheet_1.ZIP › SF18.17_SS_CS1_Downsampled_Cross-sectional Ellipse-Fit_100%RS_Pro.jpg]

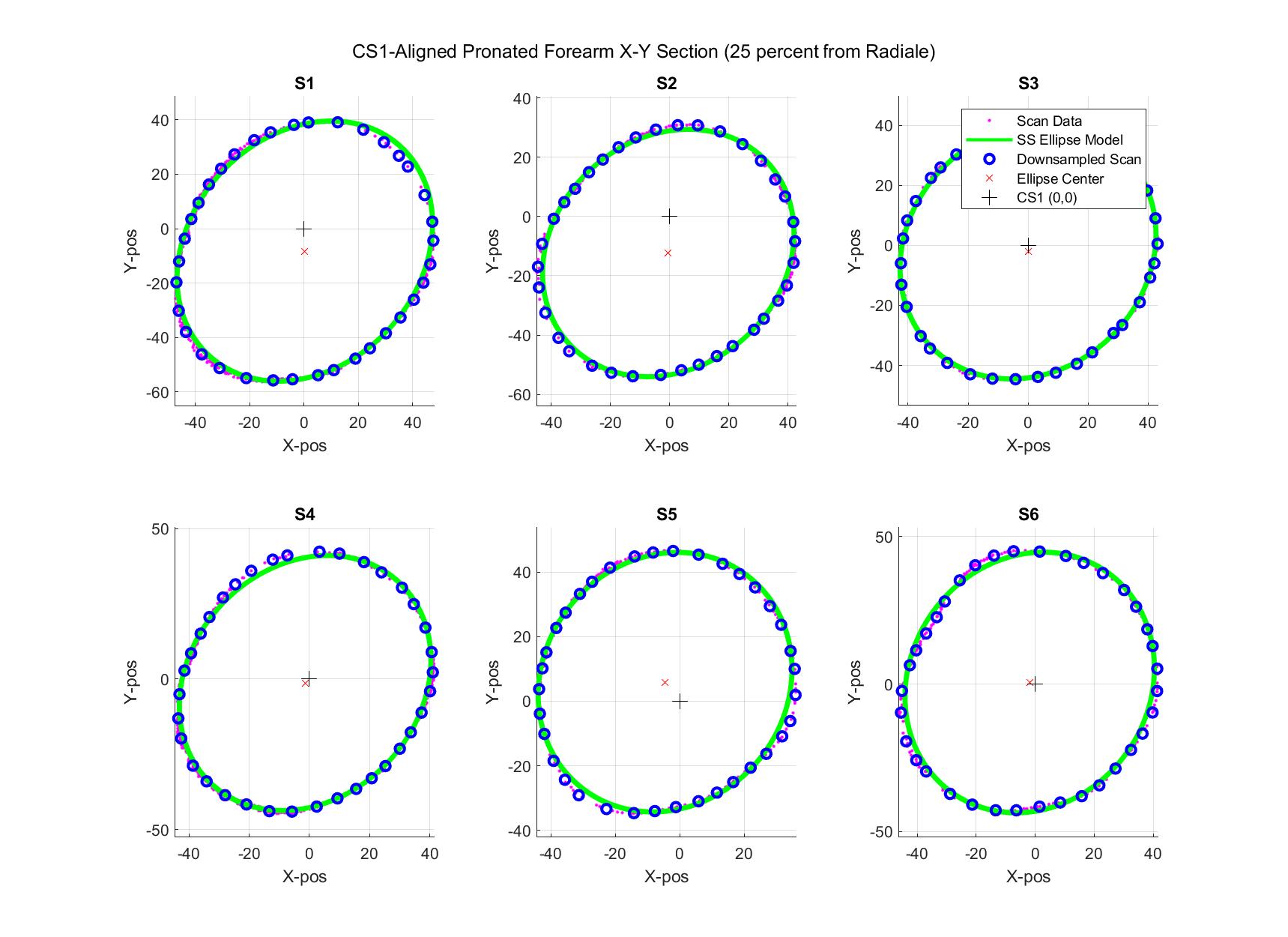

Supplement: Supplementary file 1 [file Data_Sheet_1.ZIP › SF18.2_SS_CS1_Downsampled_Cross-sectional Ellipse-Fit_25%RS_Pro.jpg]

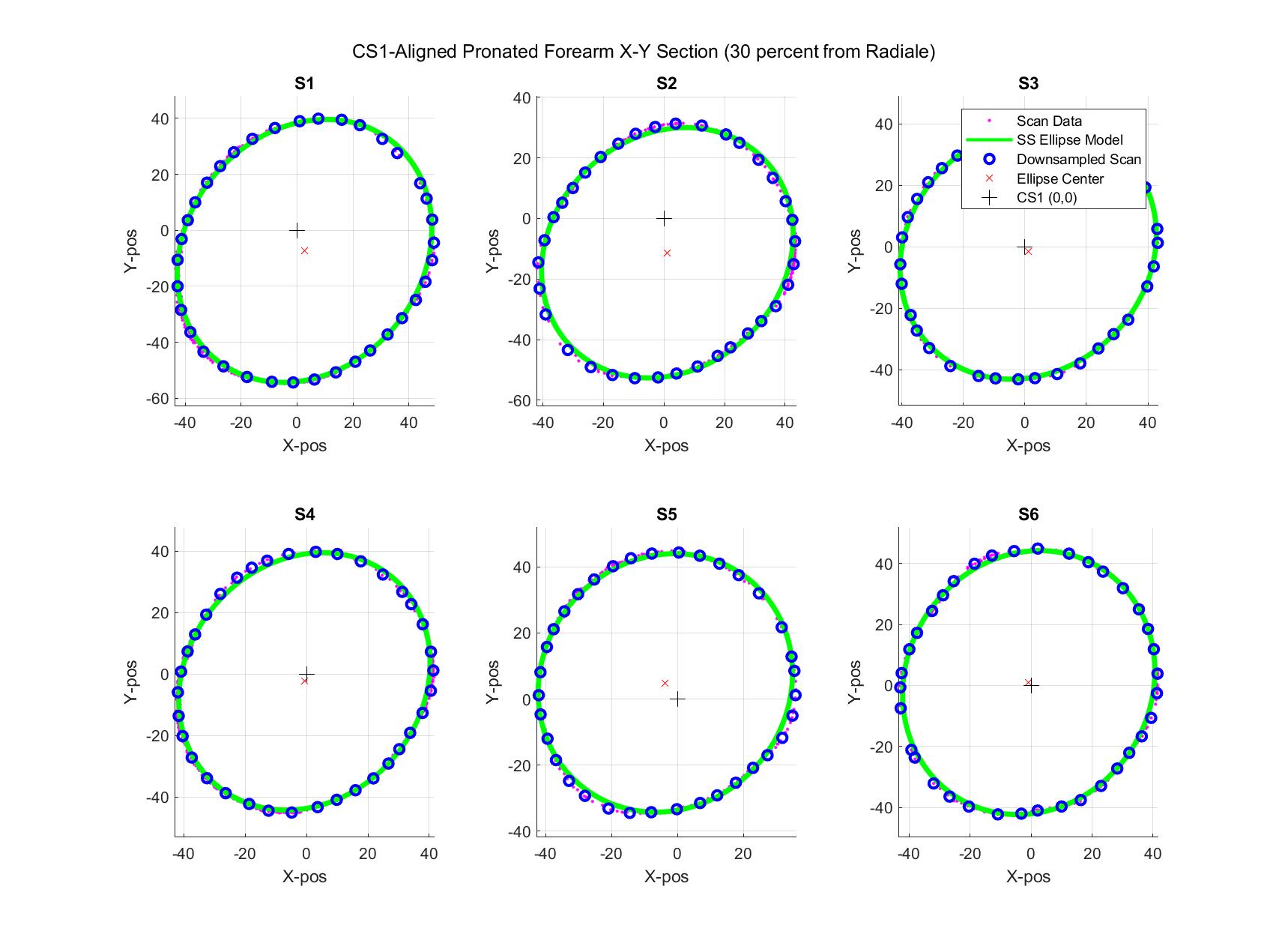

Supplement: Supplementary file 1 [file Data_Sheet_1.ZIP › SF18.3_SS_CS1_Downsampled_Cross-sectional Ellipse-Fit_30%RS_Pro.jpg]

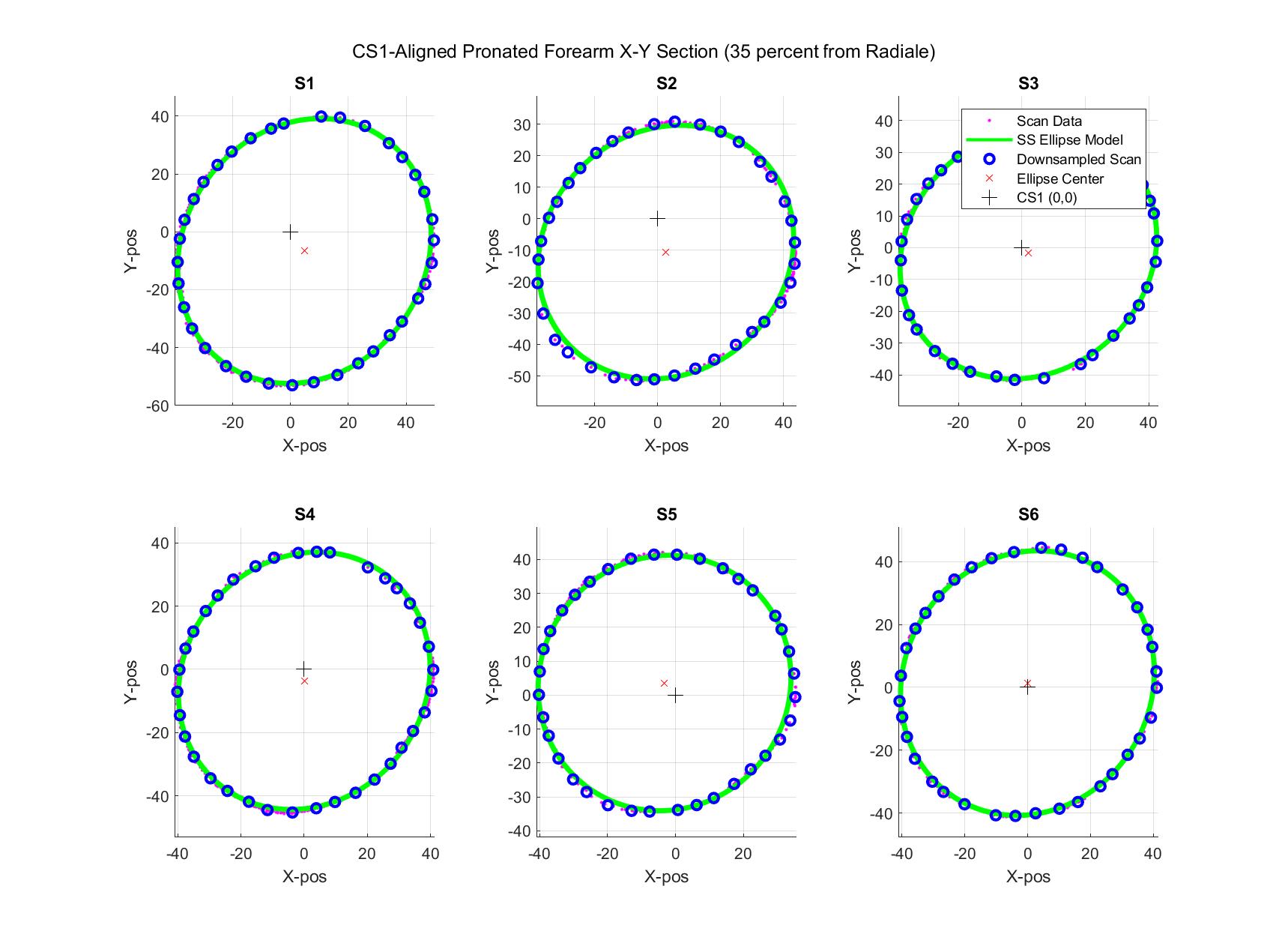

Supplement: Supplementary file 1 [file Data_Sheet_1.ZIP › SF18.4_SS_CS1_Downsampled_Cross-sectional Ellipse-Fit_35%RS_Pro.jpg]

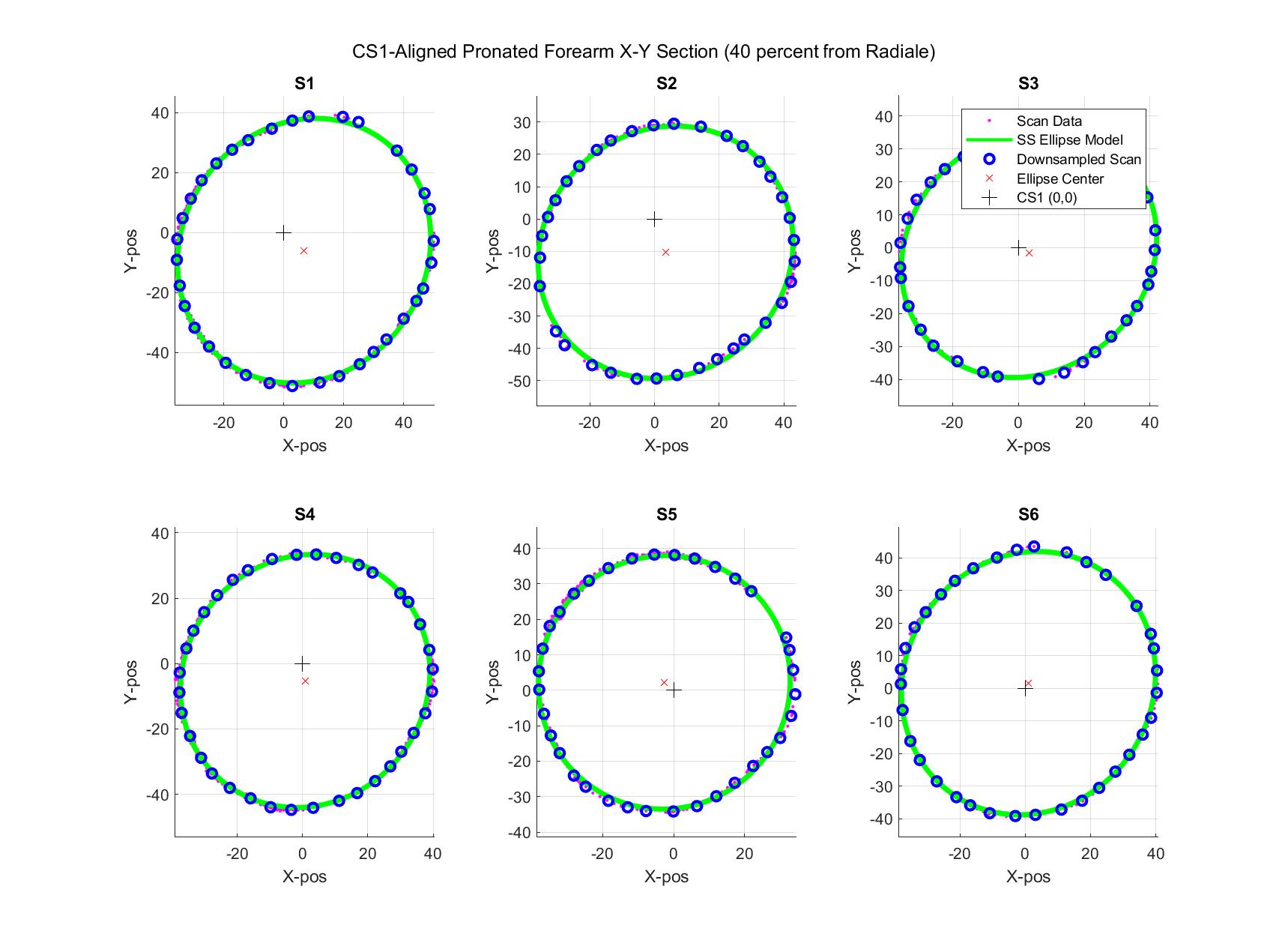

Supplement: Supplementary file 1 [file Data_Sheet_1.ZIP › SF18.5_SS_CS1_Downsampled_Cross-sectional Ellipse-Fit_40%RS_Pro.jpg]

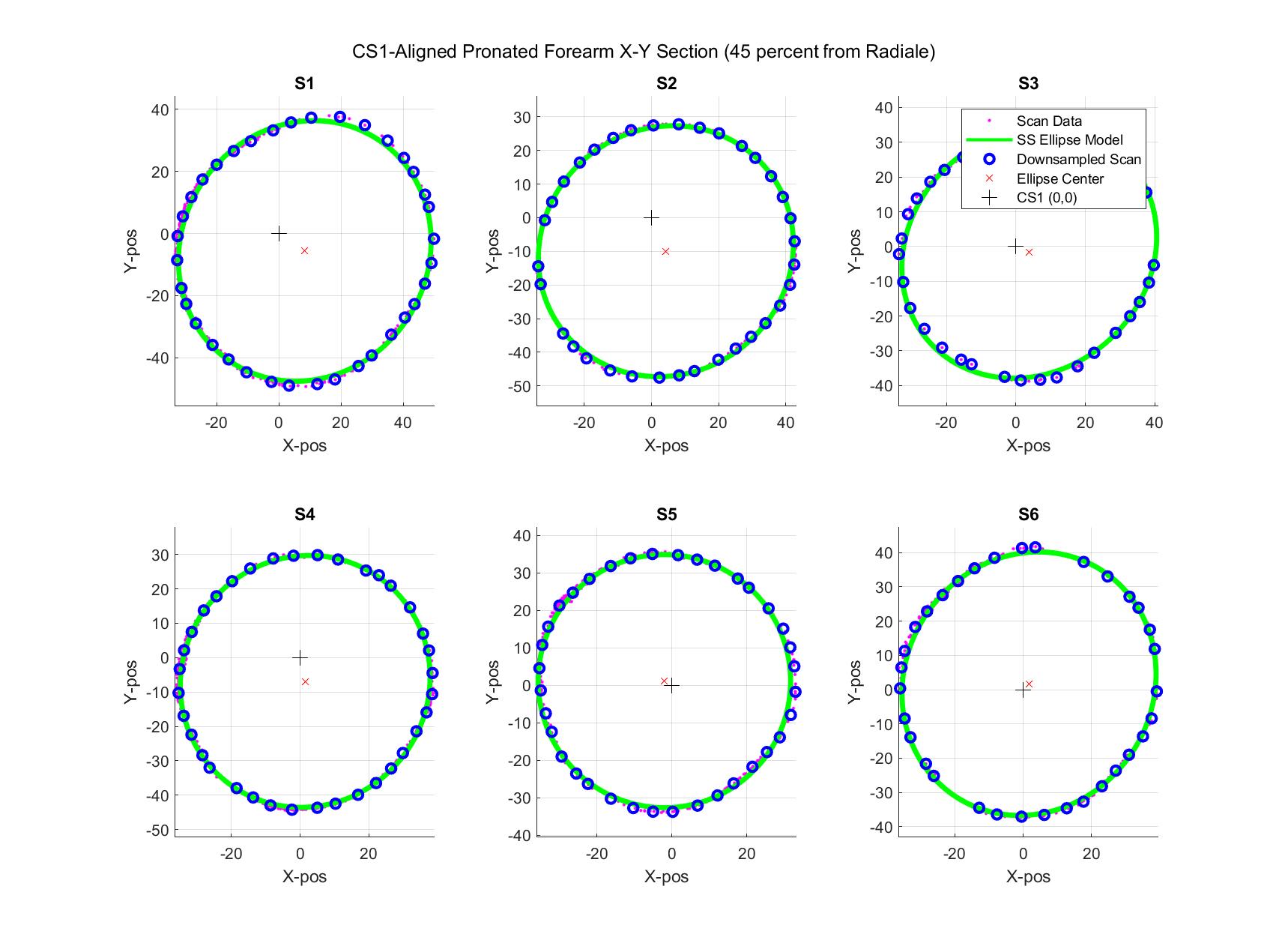

Supplement: Supplementary file 1 [file Data_Sheet_1.ZIP › SF18.6_SS_CS1_Downsampled_Cross-sectional Ellipse-Fit_45%RS_Pro.jpg]

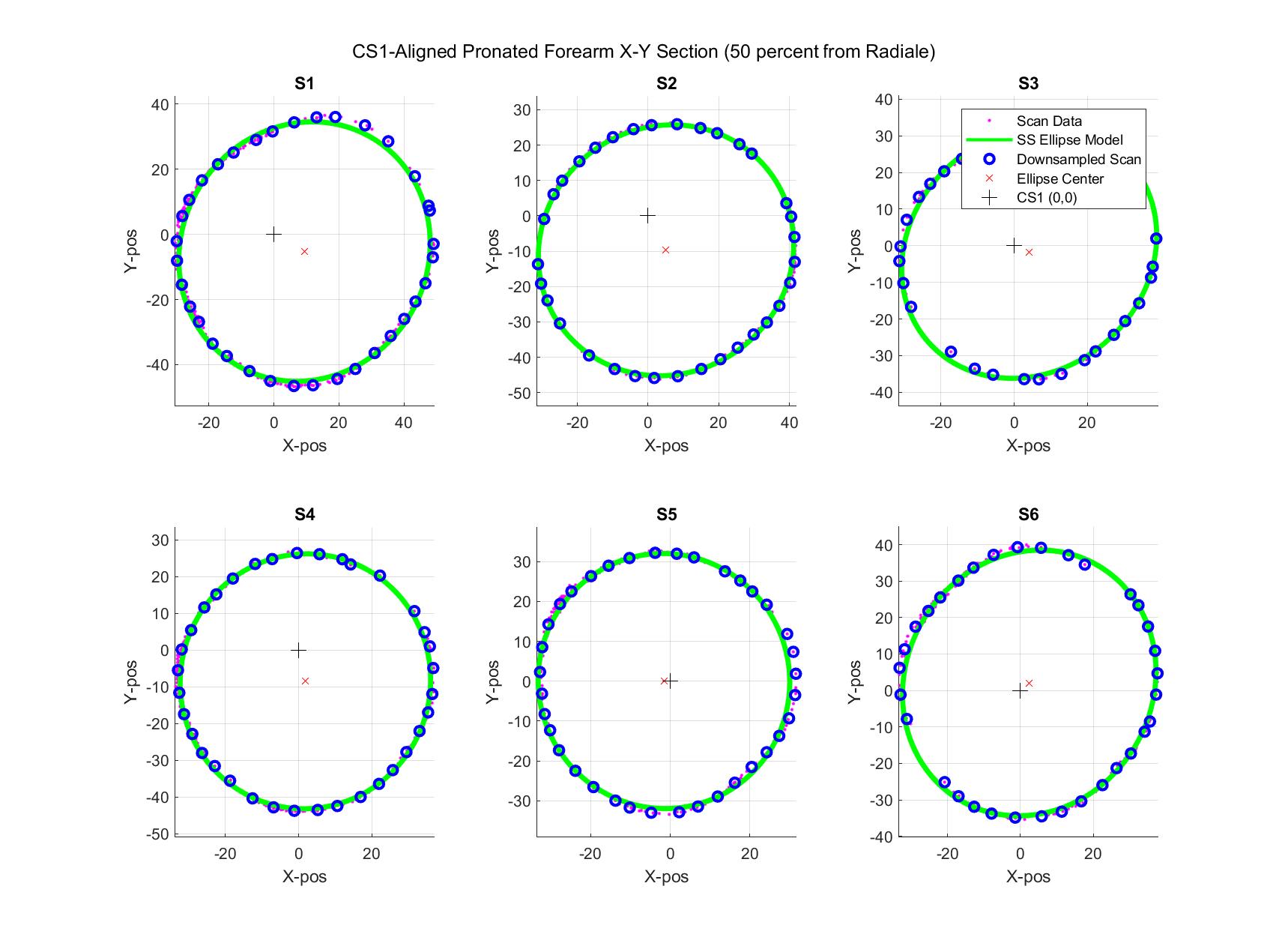

Supplement: Supplementary file 1 [file Data_Sheet_1.ZIP › SF18.7_SS_CS1_Downsampled_Cross-sectional Ellipse-Fit_50%RS_Pro.jpg]

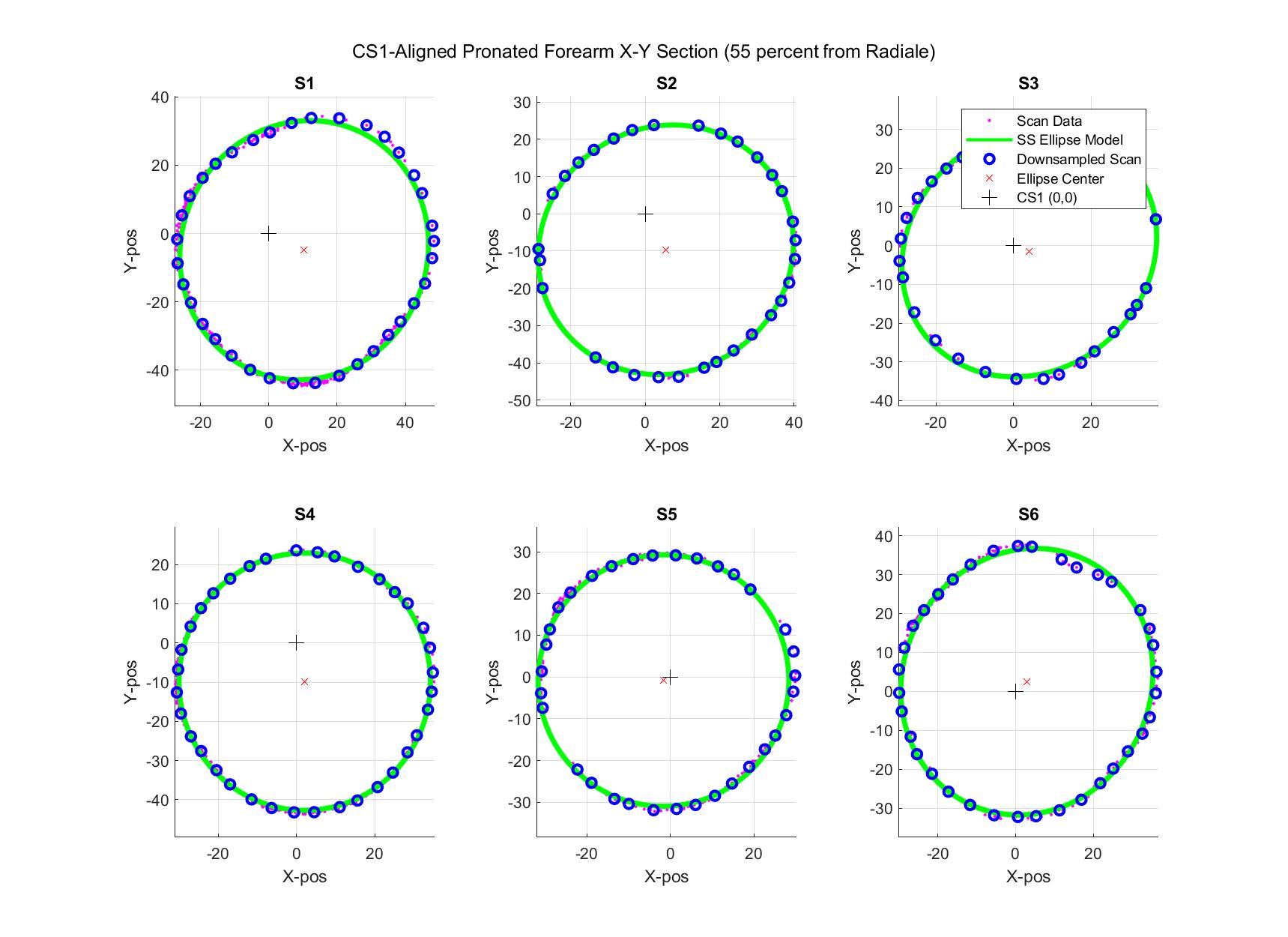

Supplement: Supplementary file 1 [file Data_Sheet_1.ZIP › SF18.8_SS_CS1_Downsampled_Cross-sectional Ellipse-Fit_55%RS_Pro.jpg]

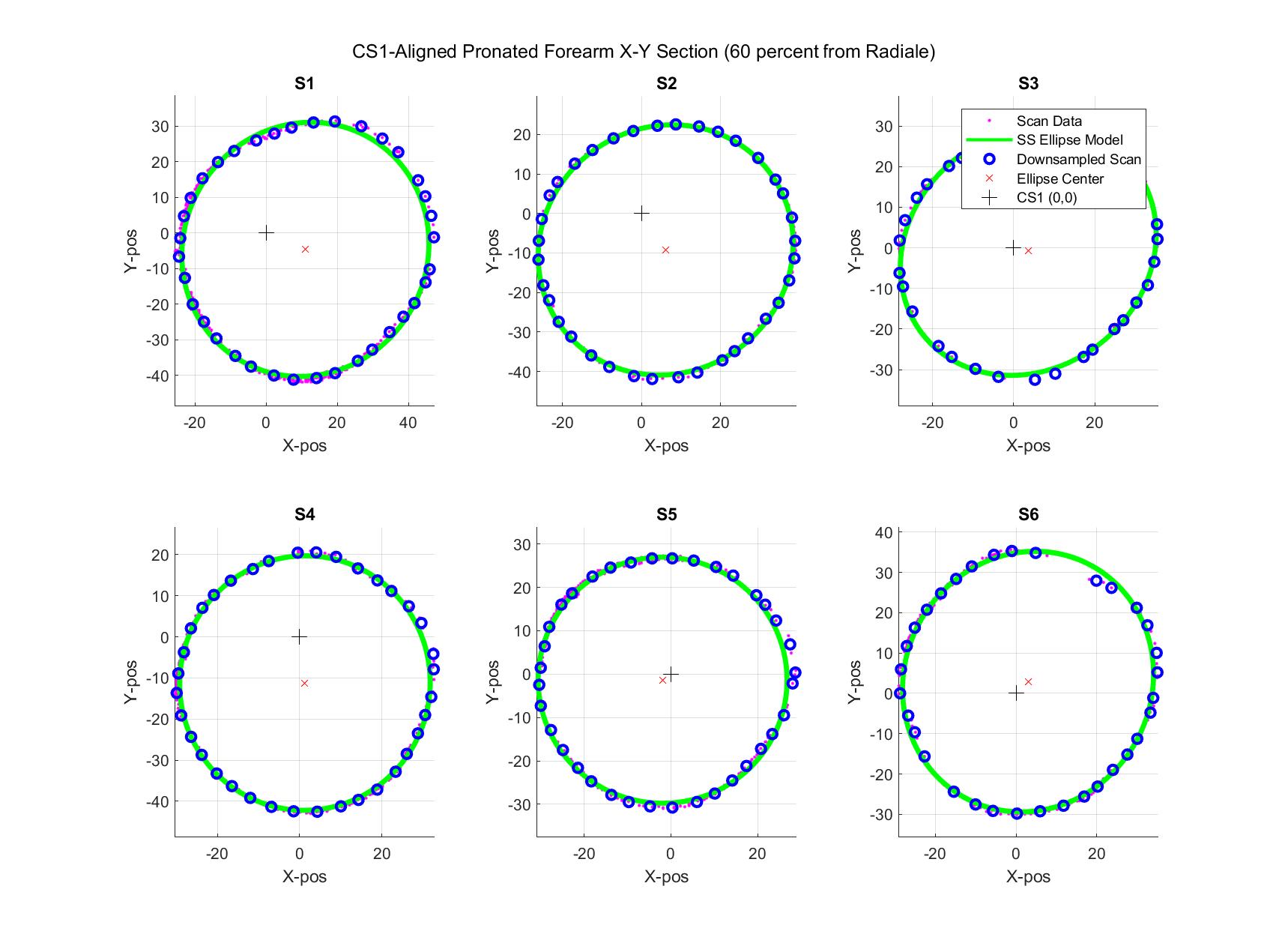

Supplement: Supplementary file 1 [file Data_Sheet_1.ZIP › SF18.9_SS_CS1_Downsampled_Cross-sectional Ellipse-Fit_60%RS_Pro.jpg]

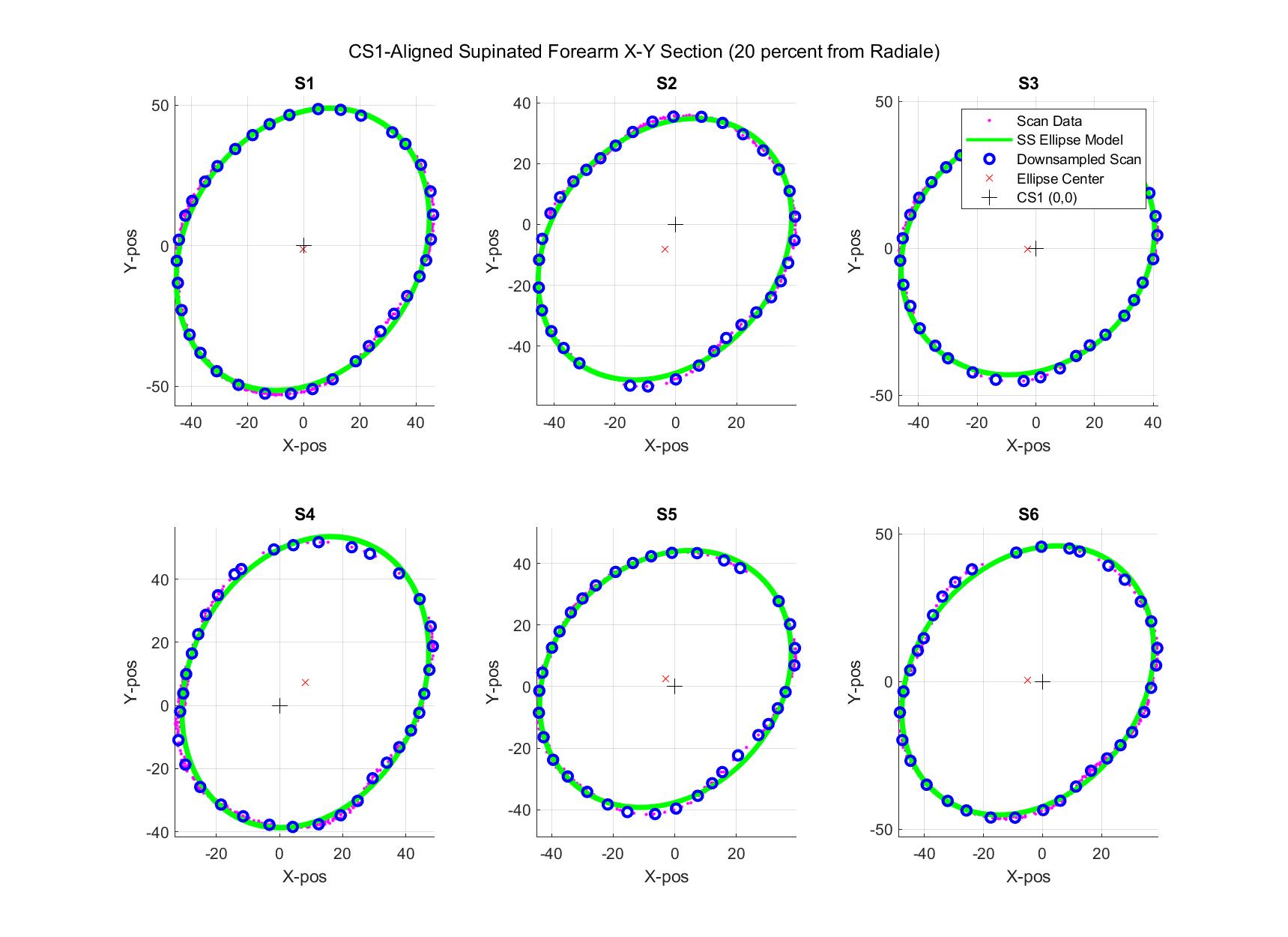

Supplement: Supplementary file 1 [file Data_Sheet_1.ZIP › SF19.1_SS_CS1_Downsampled_Cross-sectional Ellipse-Fit_20%RS_Sup.jpg]

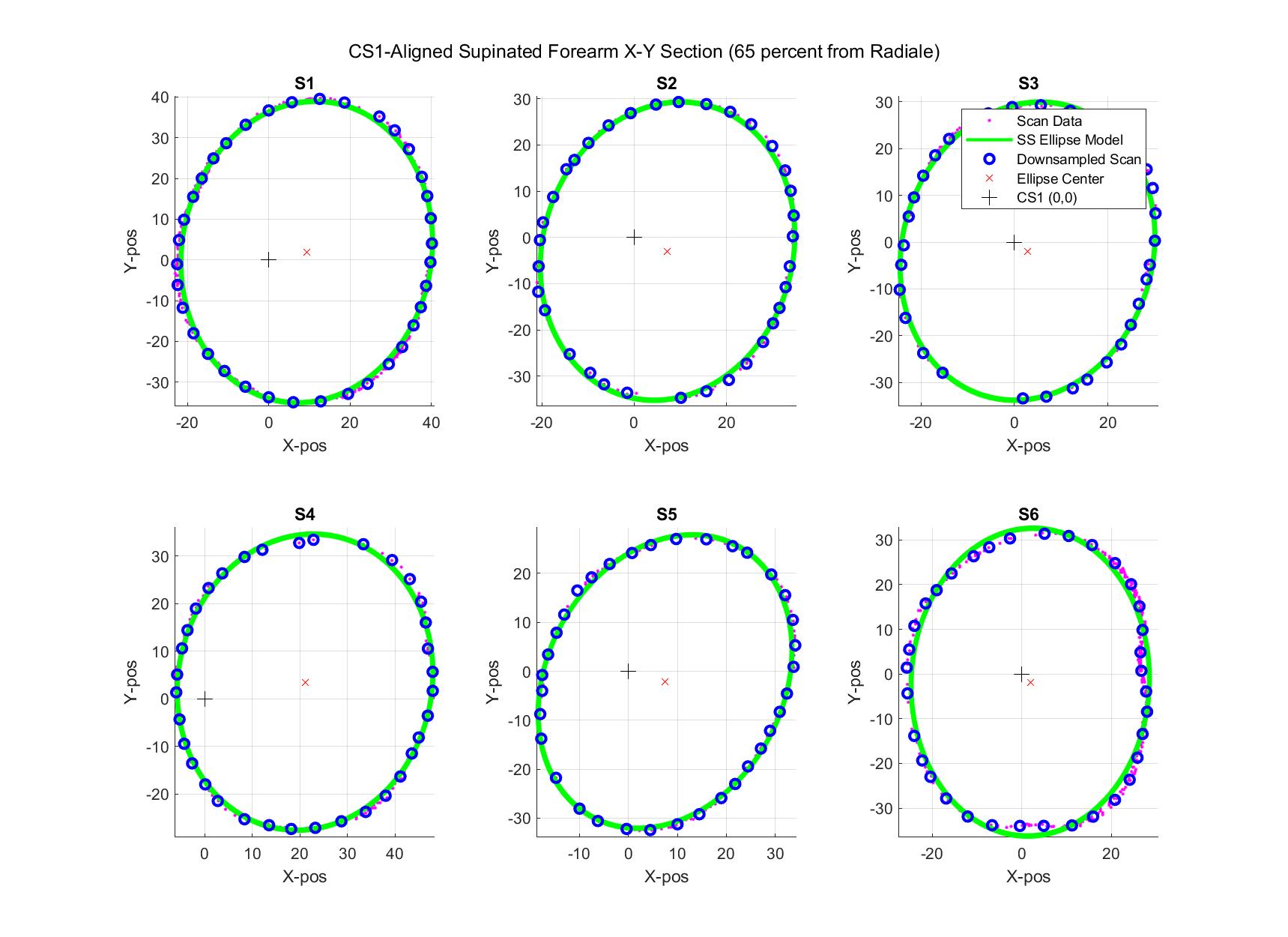

Supplement: Supplementary file 1 [file Data_Sheet_1.ZIP › SF19.10_SS_CS1_Downsampled_Cross-sectional Ellipse-Fit_65%RS_Sup.jpg]

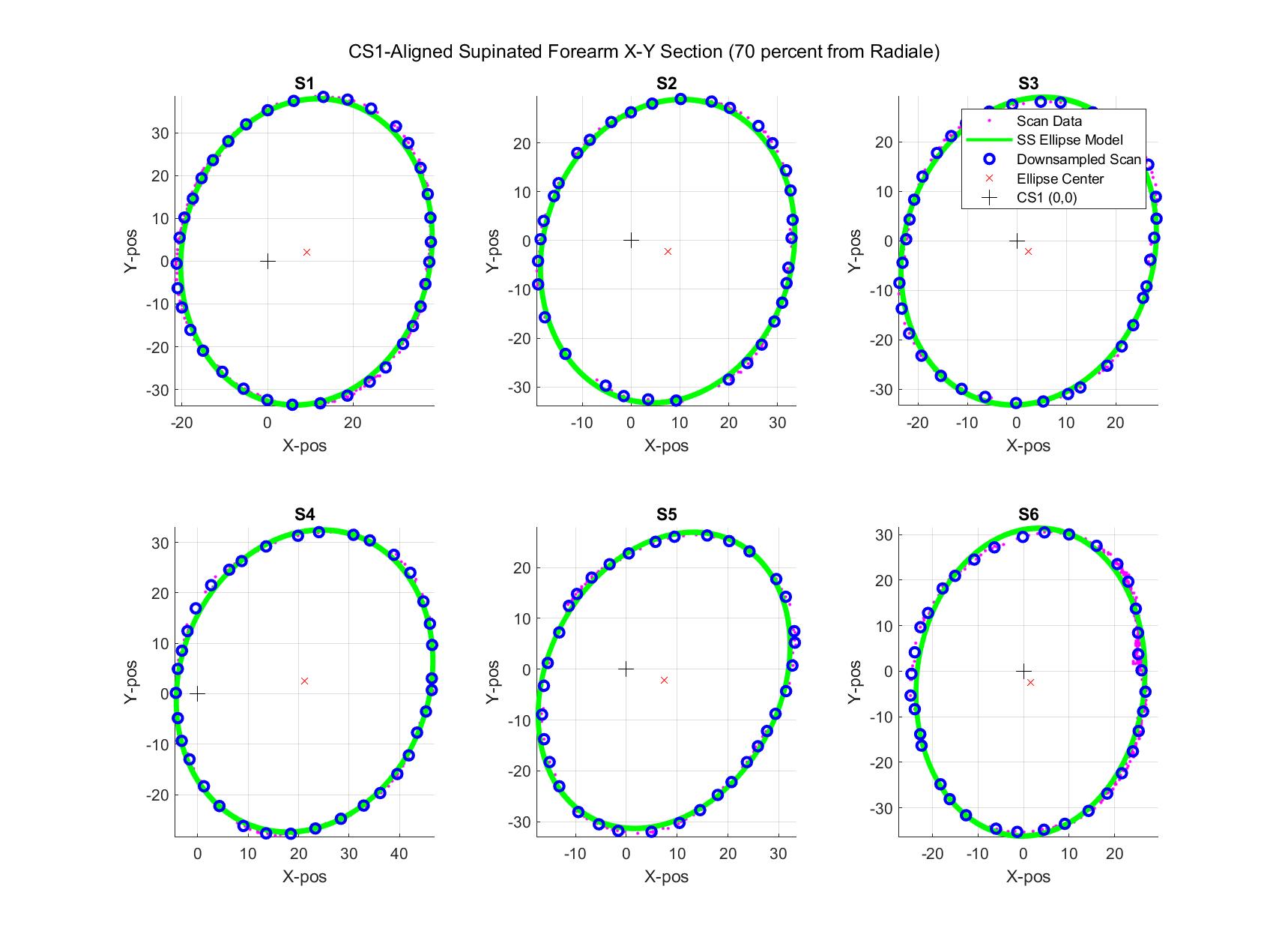

Supplement: Supplementary file 1 [file Data_Sheet_1.ZIP › SF19.11_SS_CS1_Downsampled_Cross-sectional Ellipse-Fit_70%RS_Sup.jpg]

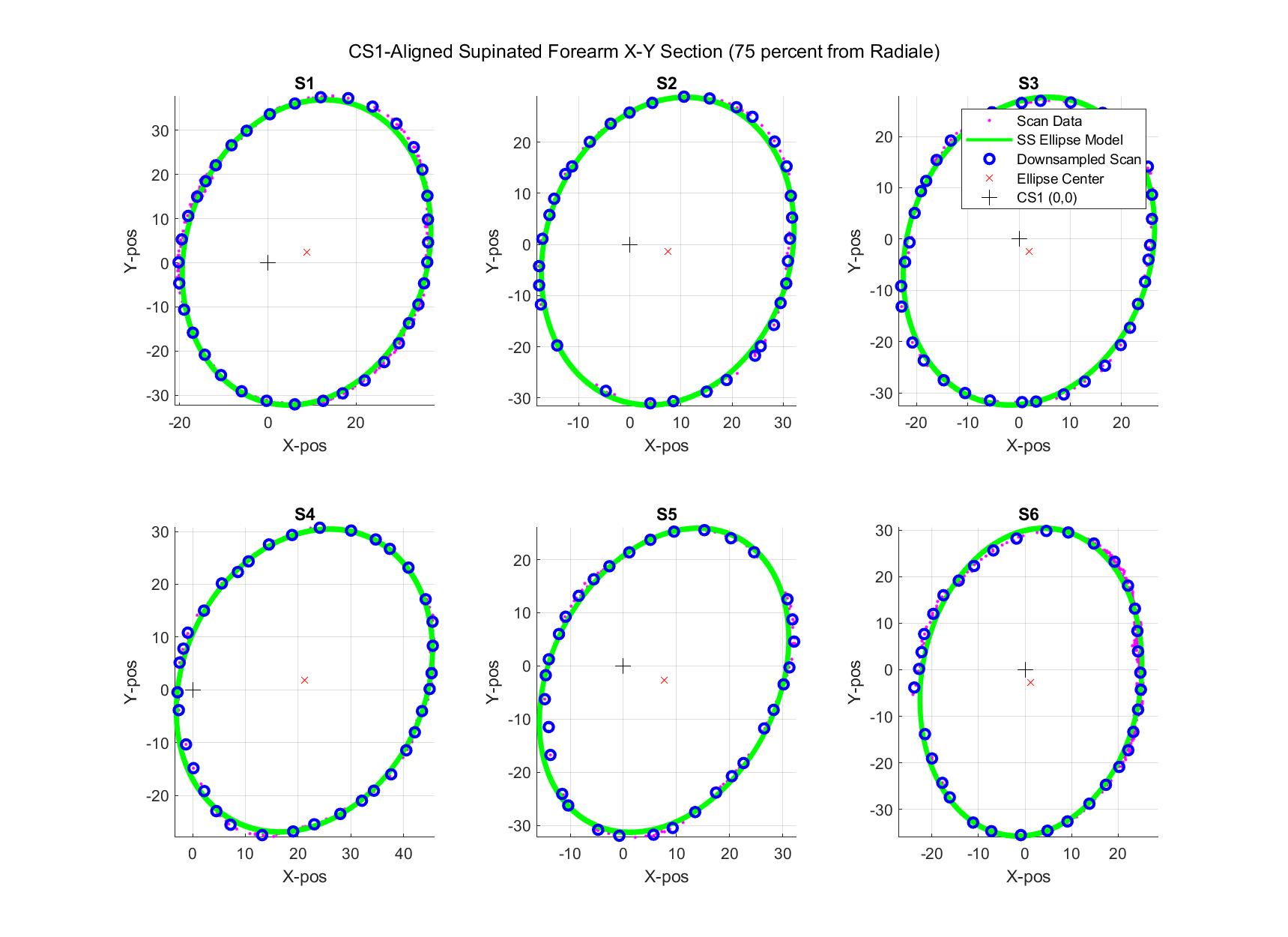

Supplement: Supplementary file 1 [file Data_Sheet_1.ZIP › SF19.12_SS_CS1_Downsampled_Cross-sectional Ellipse-Fit_75%RS_Sup.jpg]

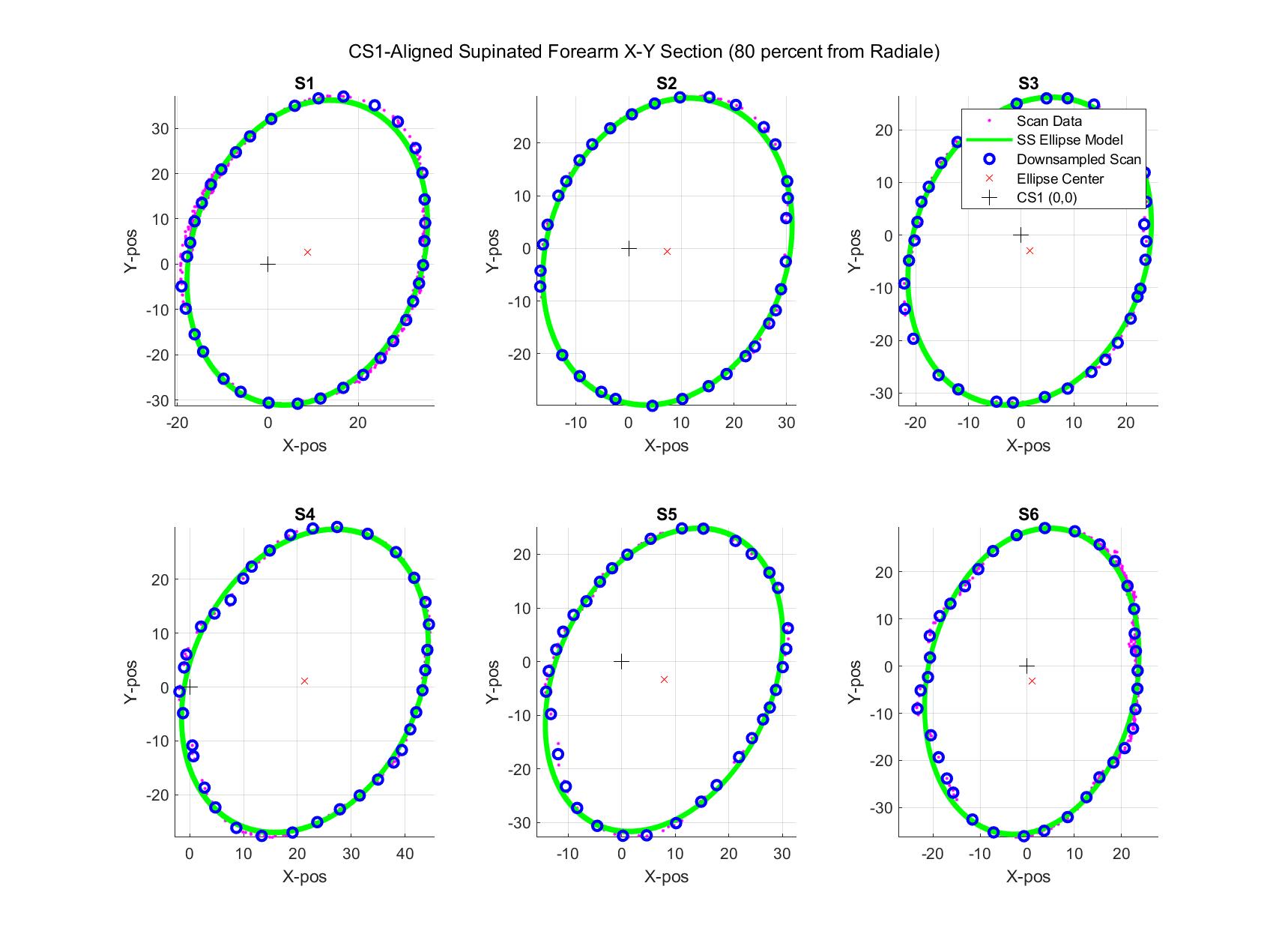

Supplement: Supplementary file 1 [file Data_Sheet_1.ZIP › SF19.13_SS_CS1_Downsampled_Cross-sectional Ellipse-Fit_80%RS_Sup.jpg]

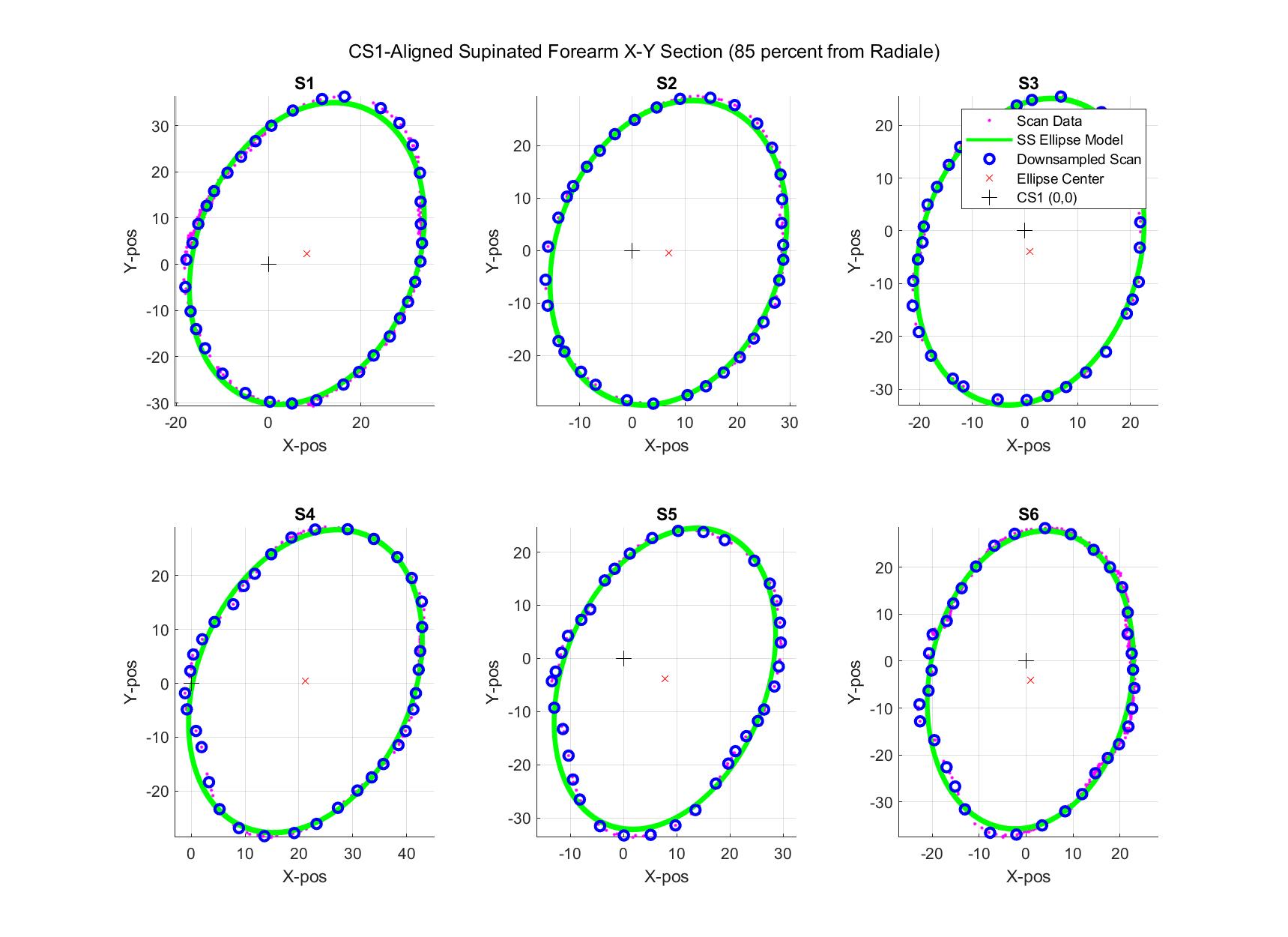

Supplement: Supplementary file 1 [file Data_Sheet_1.ZIP › SF19.14_SS_CS1_Downsampled_Cross-sectional Ellipse-Fit_85%RS_Sup.jpg]

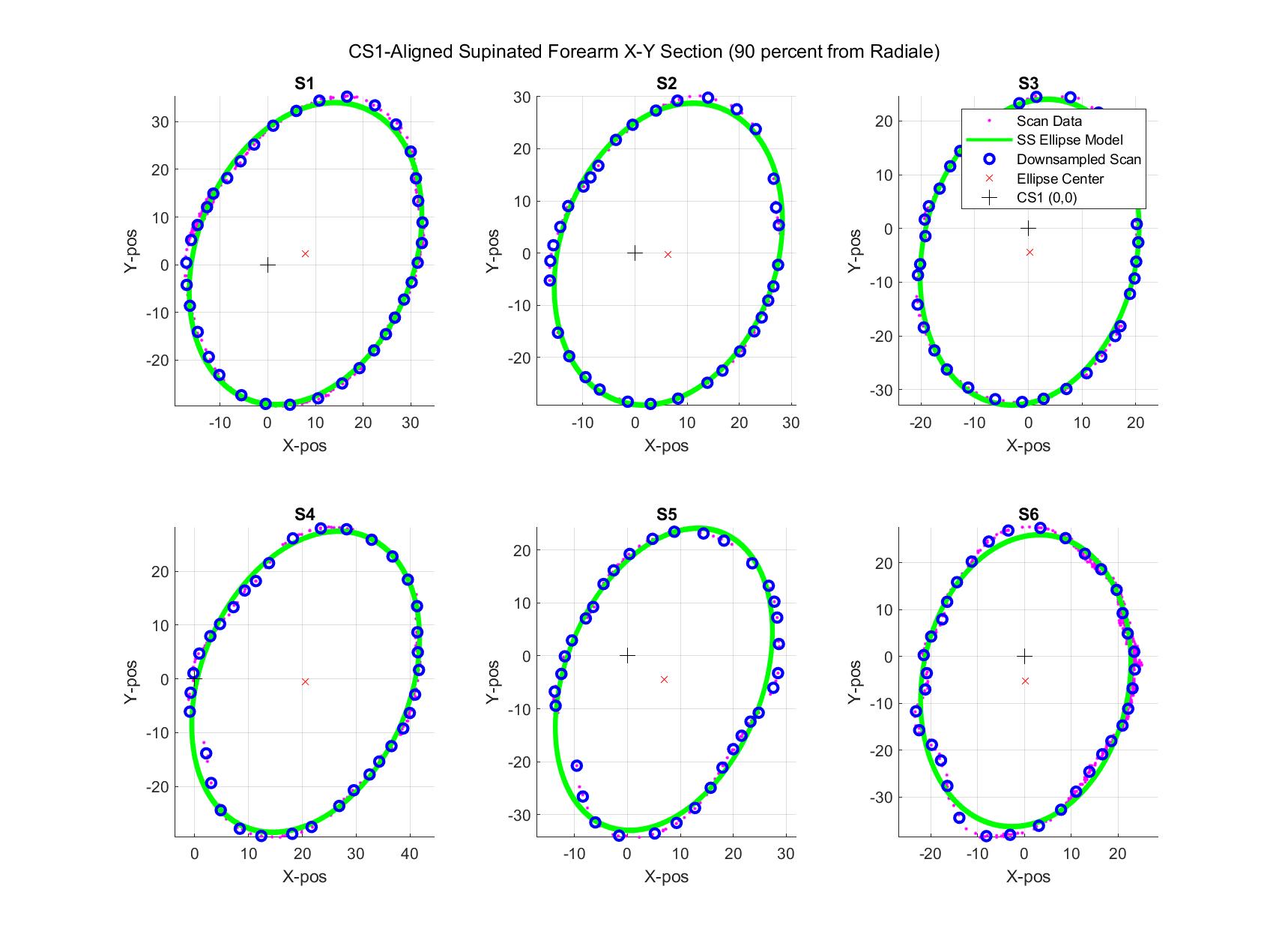

Supplement: Supplementary file 1 [file Data_Sheet_1.ZIP › SF19.15_SS_CS1_Downsampled_Cross-sectional Ellipse-Fit_90%RS_Sup.jpg]

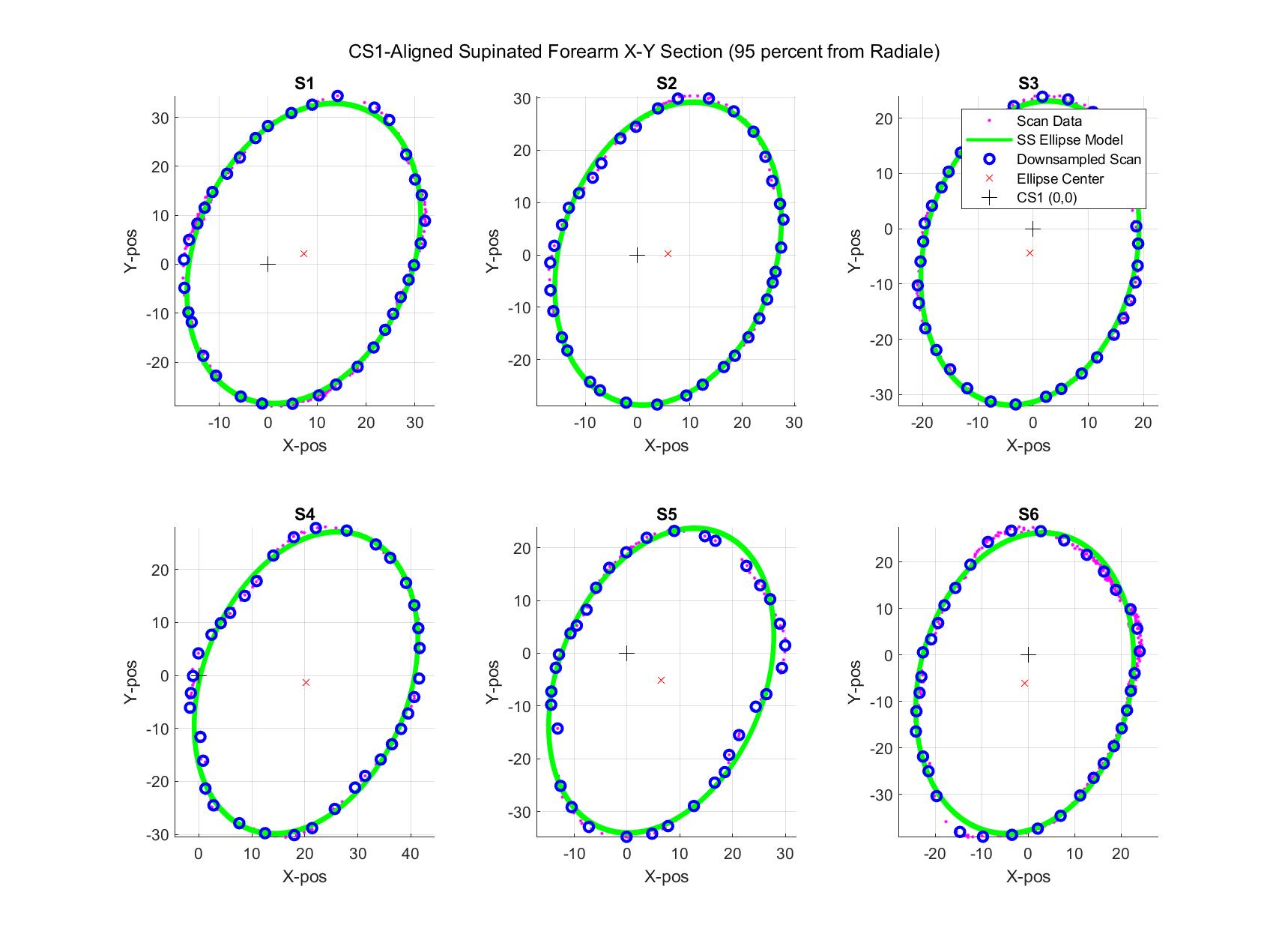

Supplement: Supplementary file 1 [file Data_Sheet_1.ZIP › SF19.16_SS_CS1_Downsampled_Cross-sectional Ellipse-Fit_95%RS_Sup.jpg]

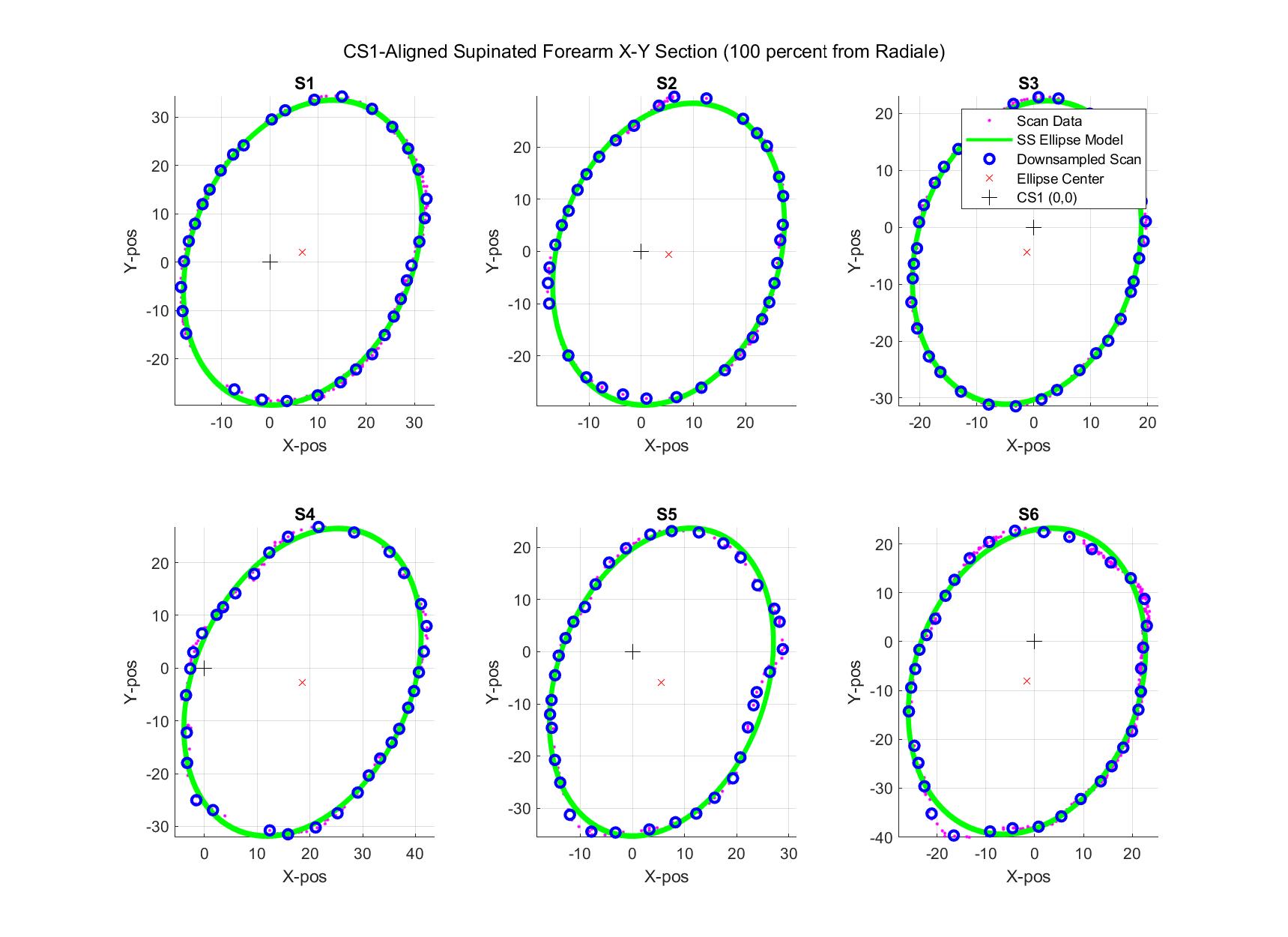

Supplement: Supplementary file 1 [file Data_Sheet_1.ZIP › SF19.17_SS_CS1_Downsampled_Cross-sectional Ellipse-Fit_100%RS_Sup.jpg]

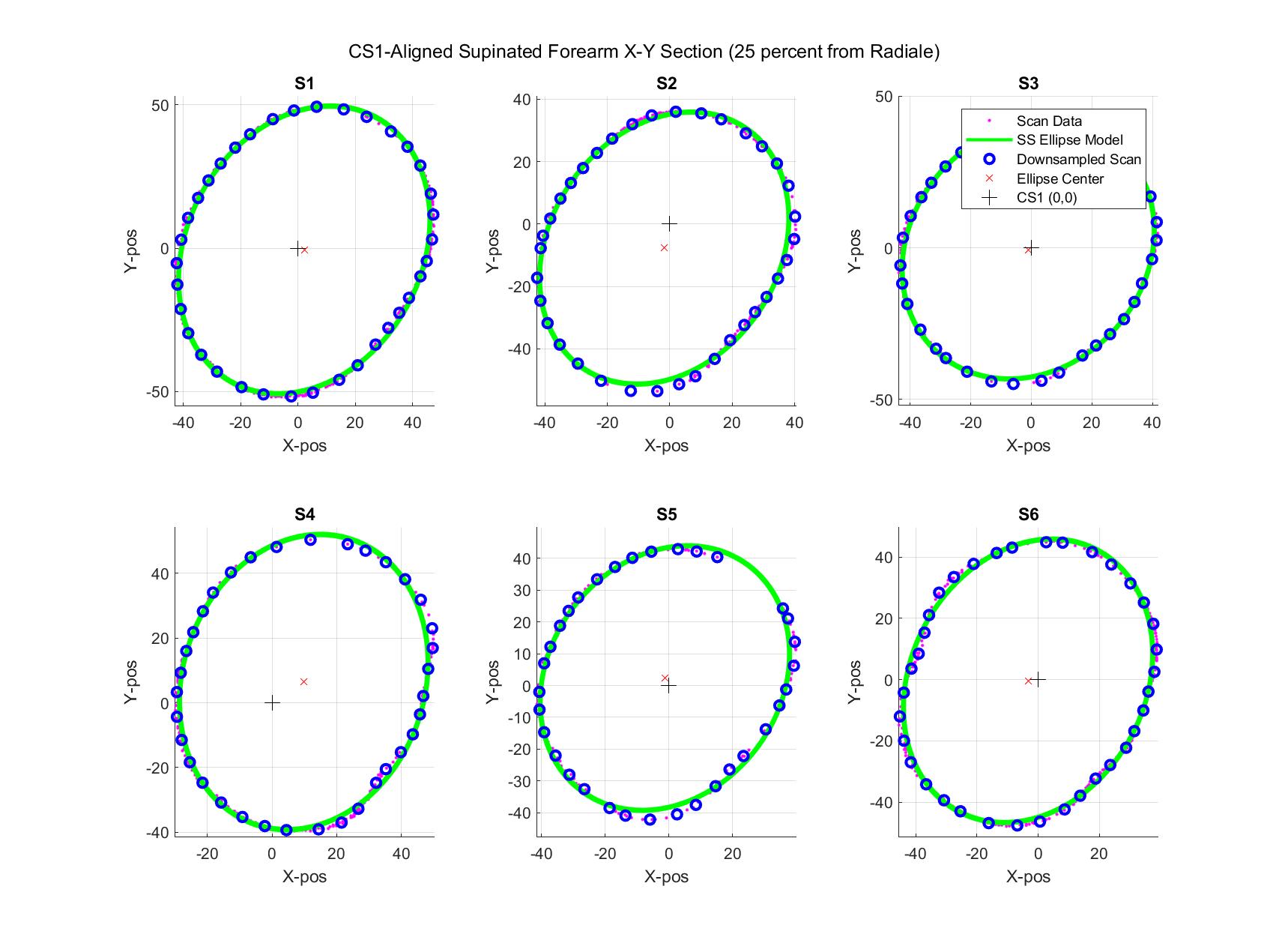

Supplement: Supplementary file 1 [file Data_Sheet_1.ZIP › SF19.2_SS_CS1_Downsampled_Cross-sectional Ellipse-Fit_25%RS_Sup.jpg]

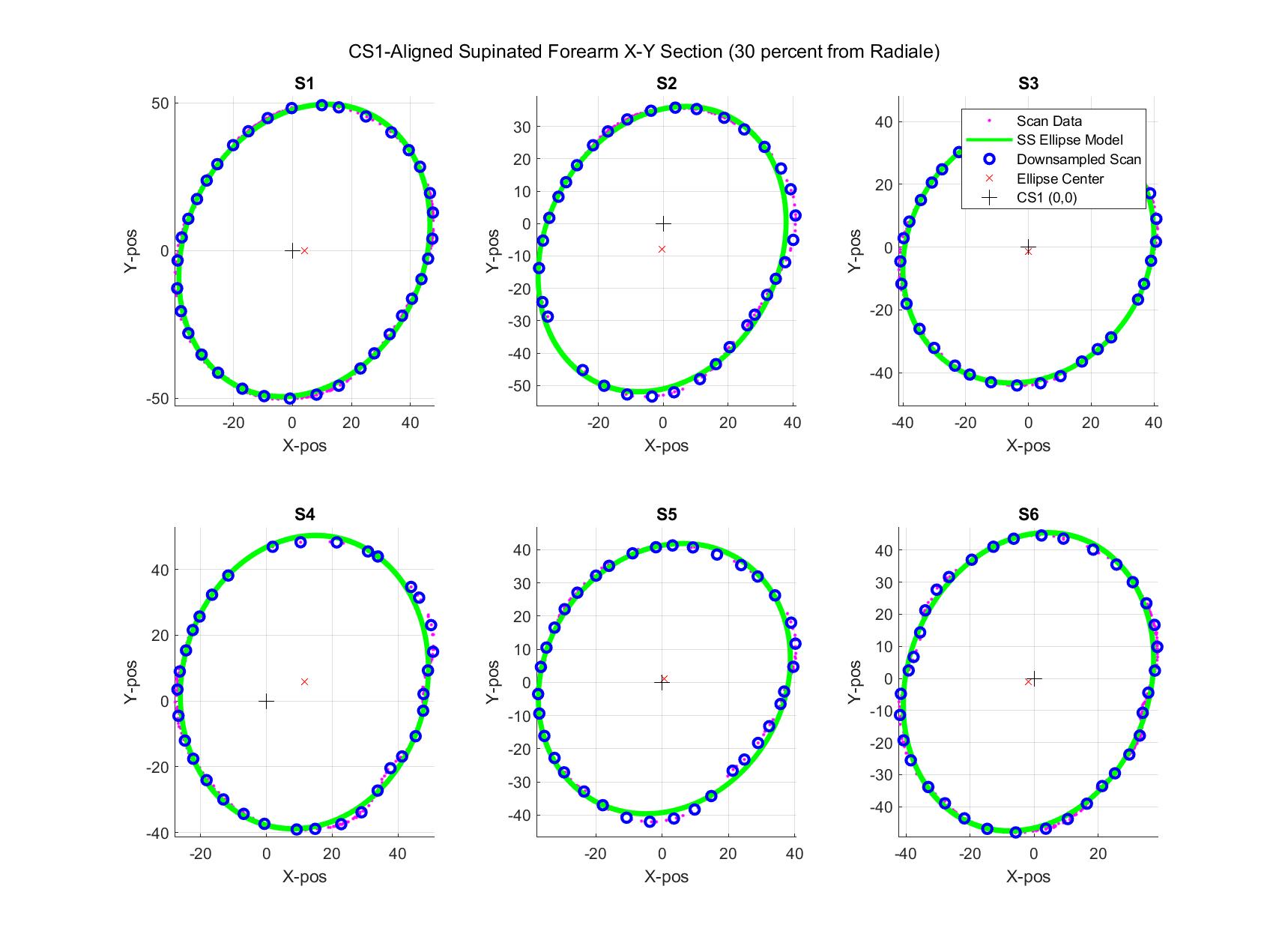

Supplement: Supplementary file 1 [file Data_Sheet_1.ZIP › SF19.3_SS_CS1_Downsampled_Cross-sectional Ellipse-Fit_30%RS_Sup.jpg]

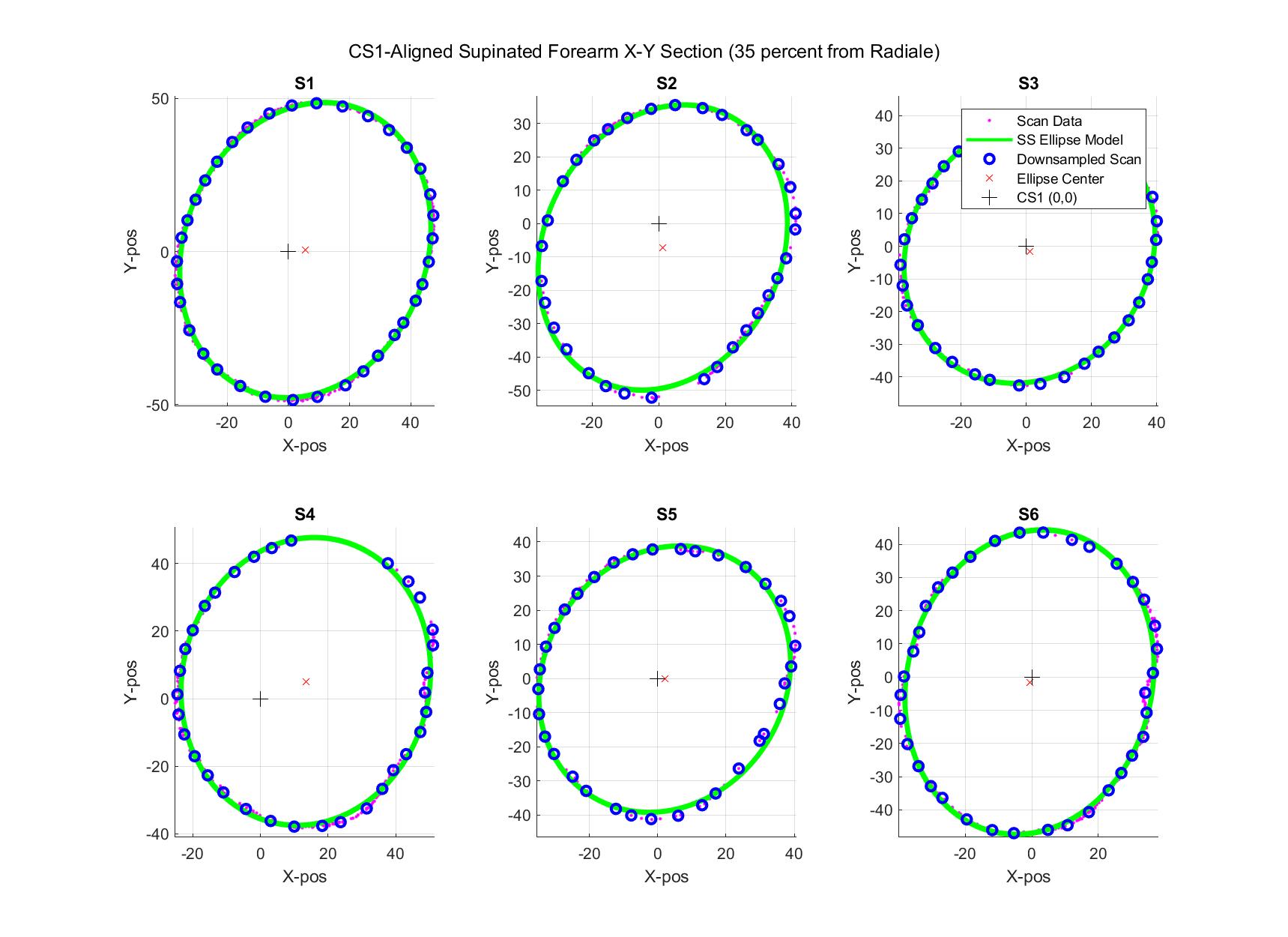

Supplement: Supplementary file 1 [file Data_Sheet_1.ZIP › SF19.4_SS_CS1_Downsampled_Cross-sectional Ellipse-Fit_35%RS_Sup.jpg]

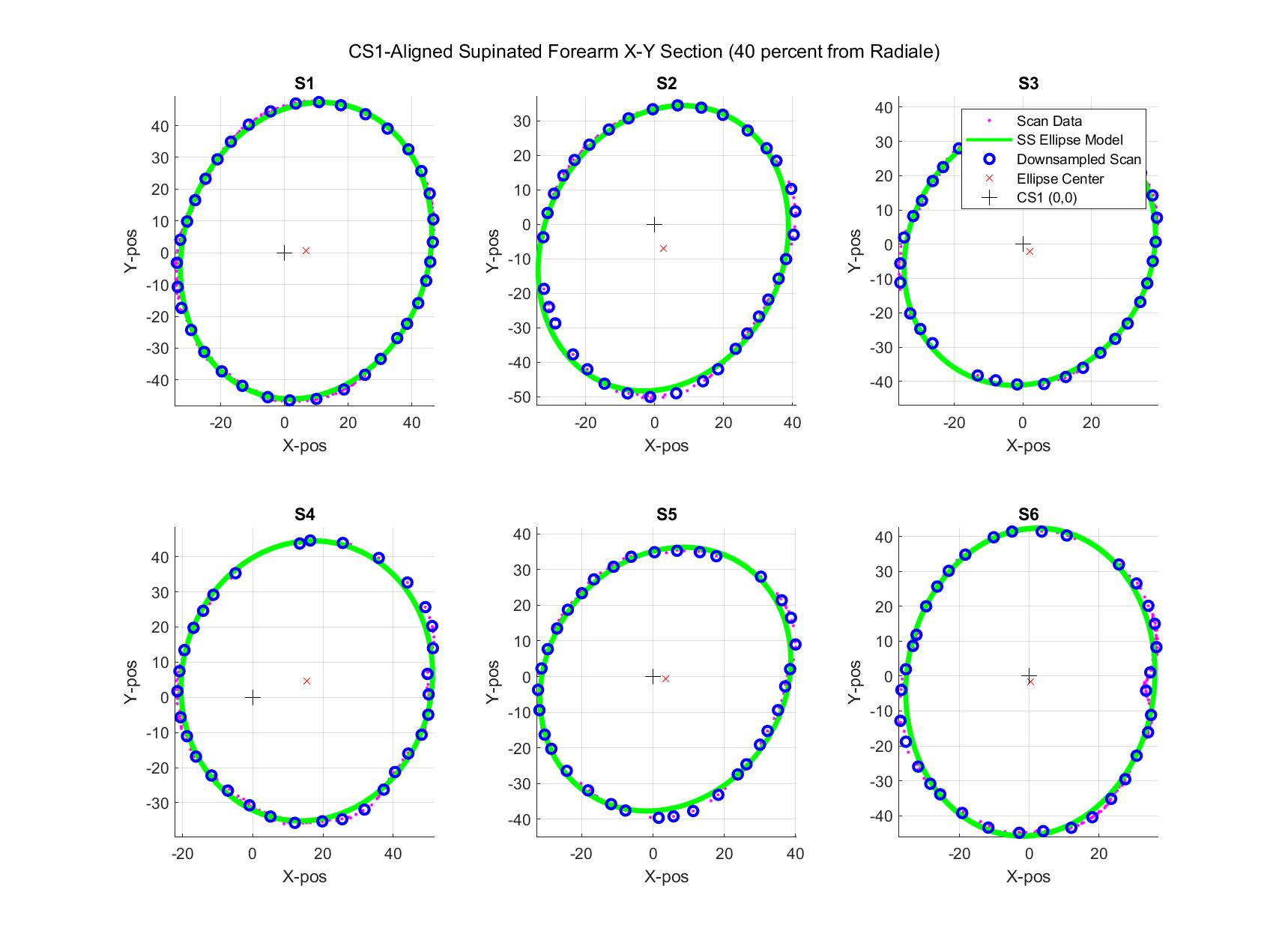

Supplement: Supplementary file 1 [file Data_Sheet_1.ZIP › SF19.5_SS_CS1_Downsampled_Cross-sectional Ellipse-Fit_40%RS_Sup.jpg]

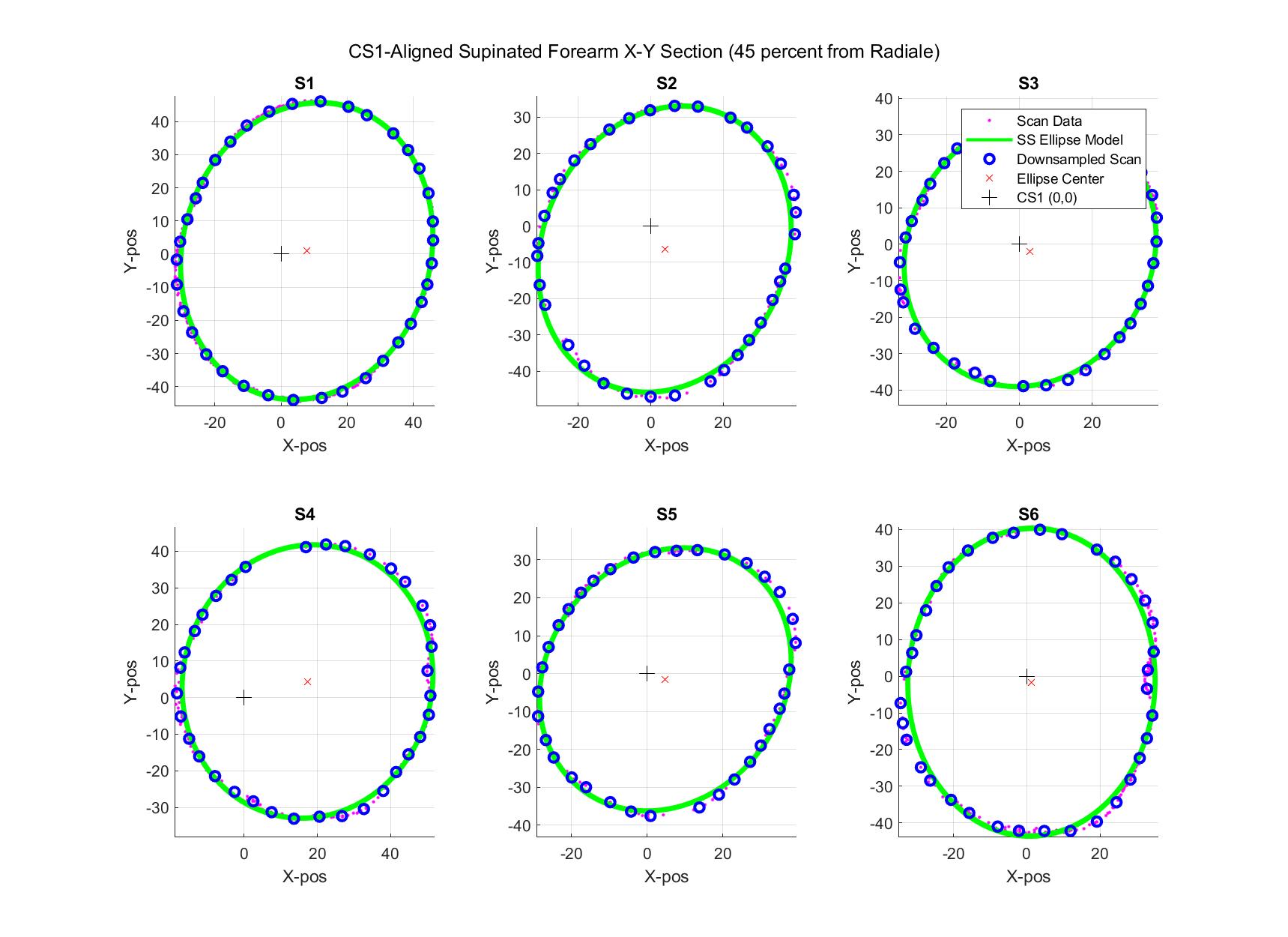

Supplement: Supplementary file 1 [file Data_Sheet_1.ZIP › SF19.6_SS_CS1_Downsampled_Cross-sectional Ellipse-Fit_45%RS_Sup.jpg]

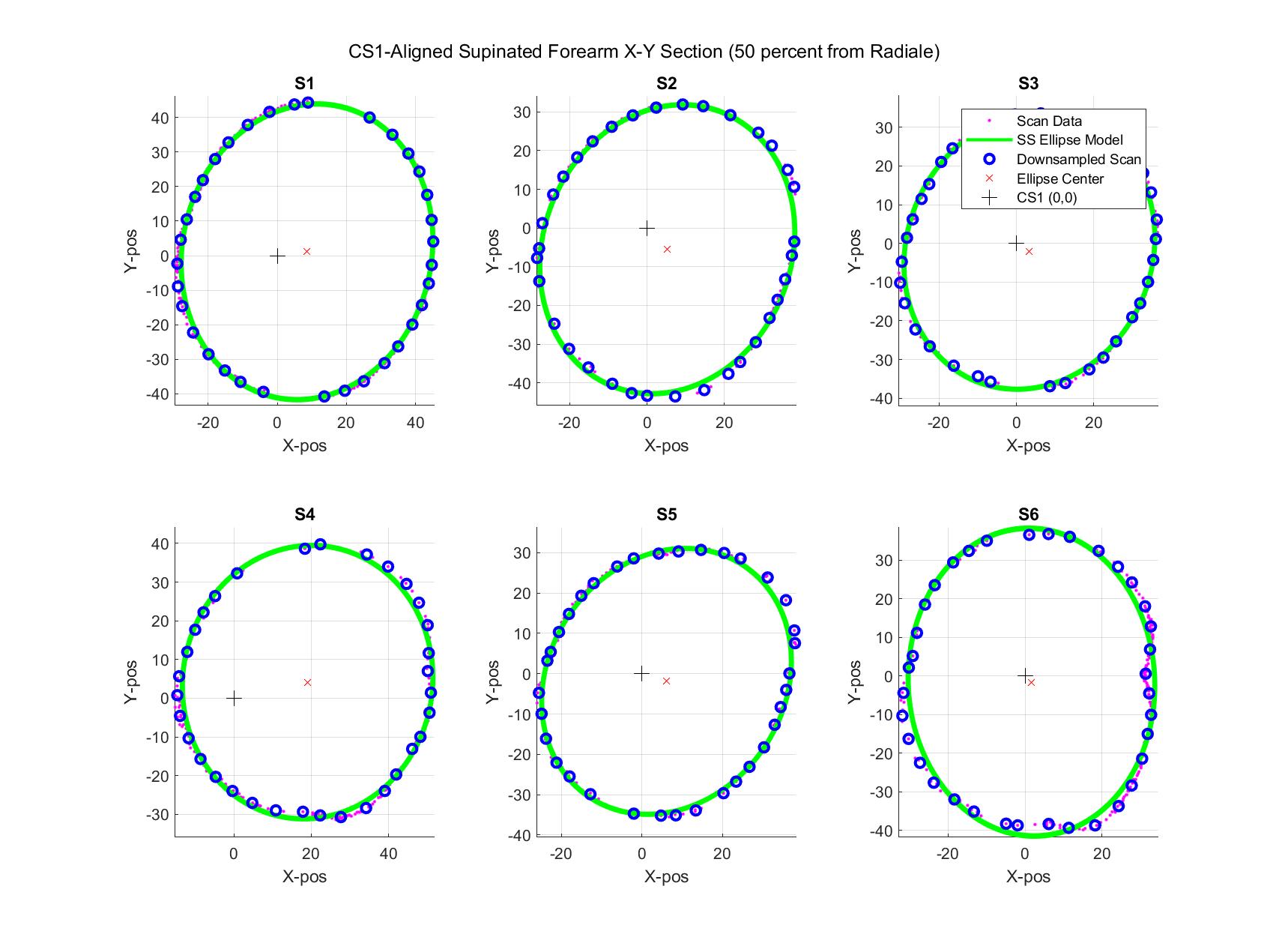

Supplement: Supplementary file 1 [file Data_Sheet_1.ZIP › SF19.7_SS_CS1_Downsampled_Cross-sectional Ellipse-Fit_50%RS_Sup.jpg]

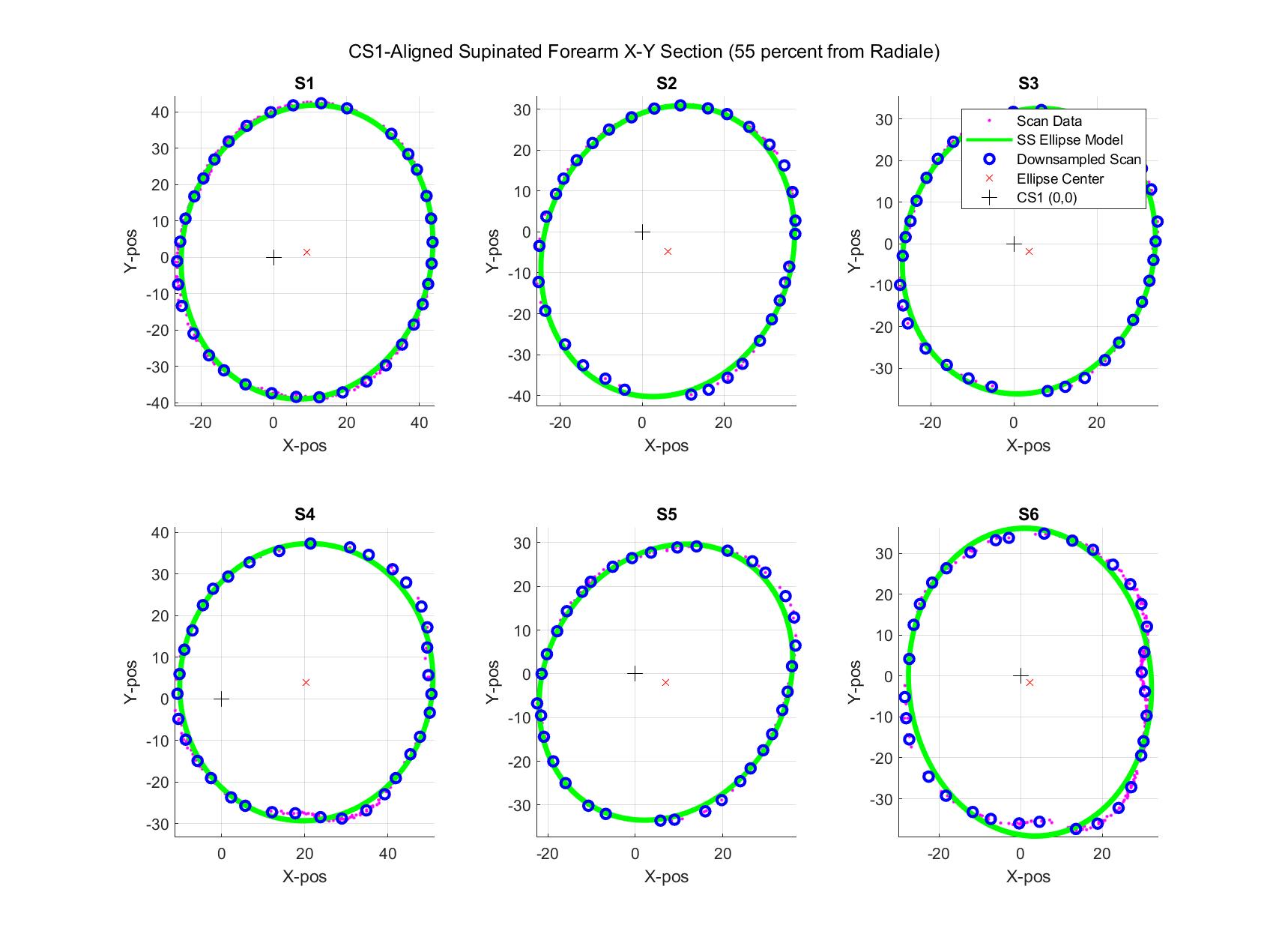

Supplement: Supplementary file 1 [file Data_Sheet_1.ZIP › SF19.8_SS_CS1_Downsampled_Cross-sectional Ellipse-Fit_55%RS_Sup.jpg]

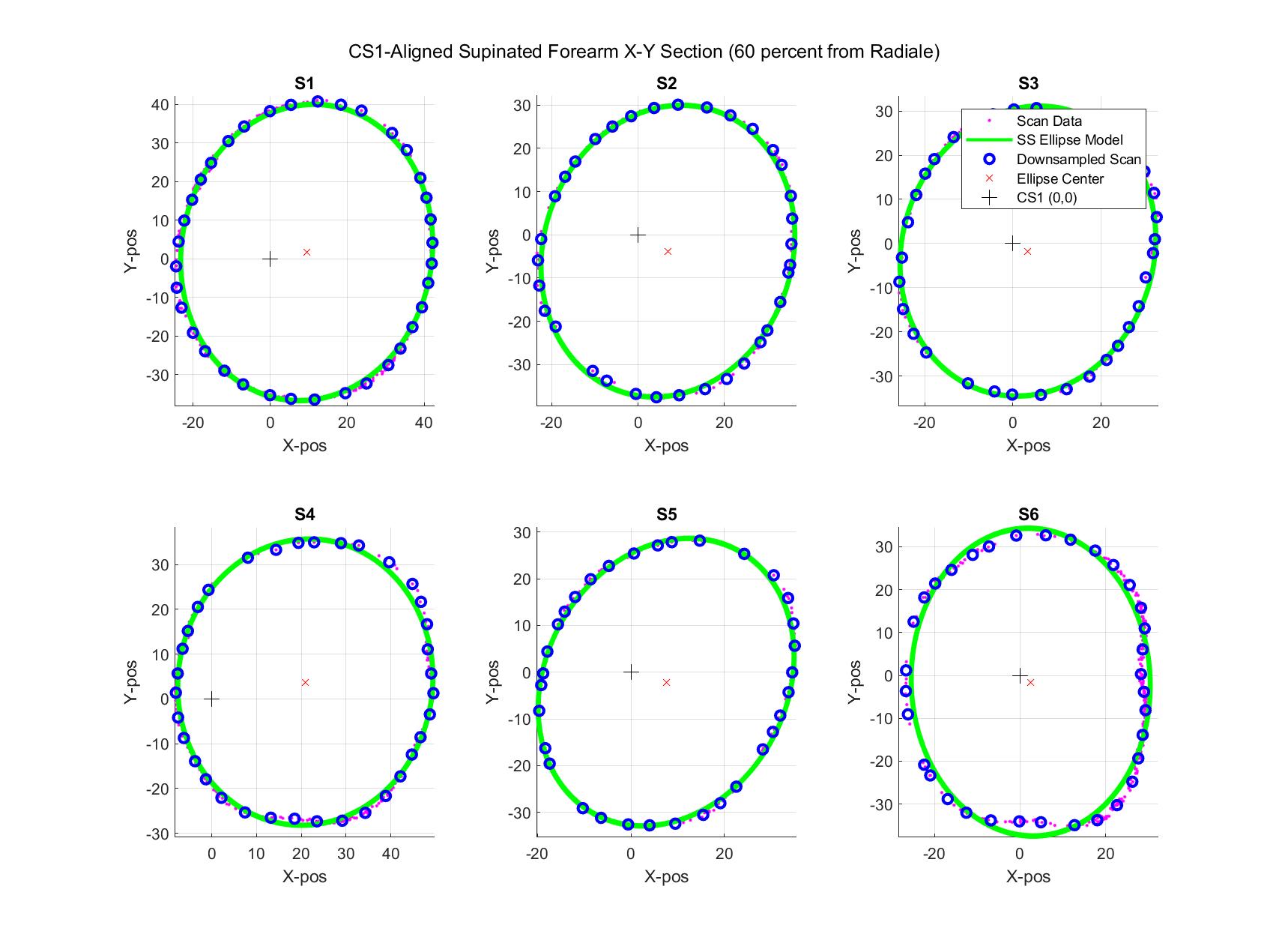

Supplement: Supplementary file 1 [file Data_Sheet_1.ZIP › SF19.9_SS_CS1_Downsampled_Cross-sectional Ellipse-Fit_60%RS_Sup.jpg]

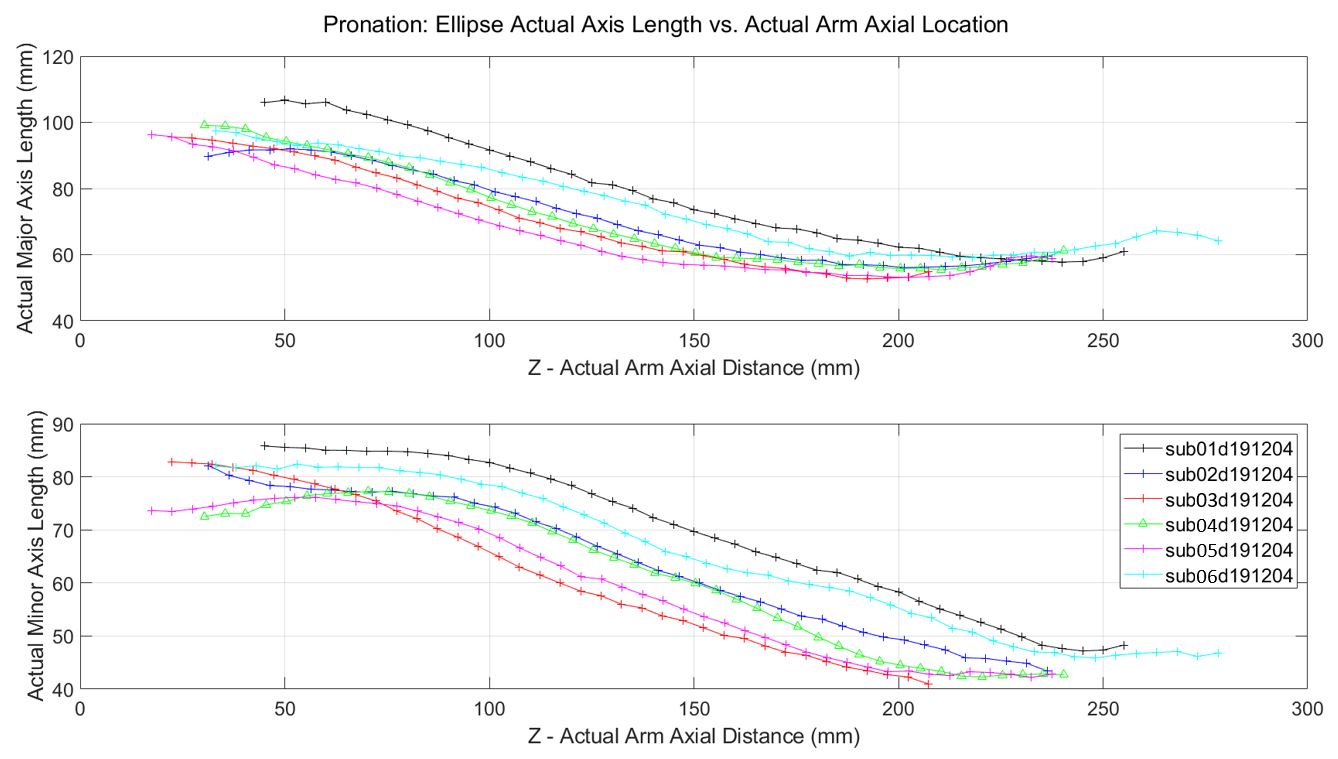

Supplement: Supplementary file 1 [file Data_Sheet_1.ZIP › SF2.1_Raw_AxisLengths_ALL_Pro.JPG]

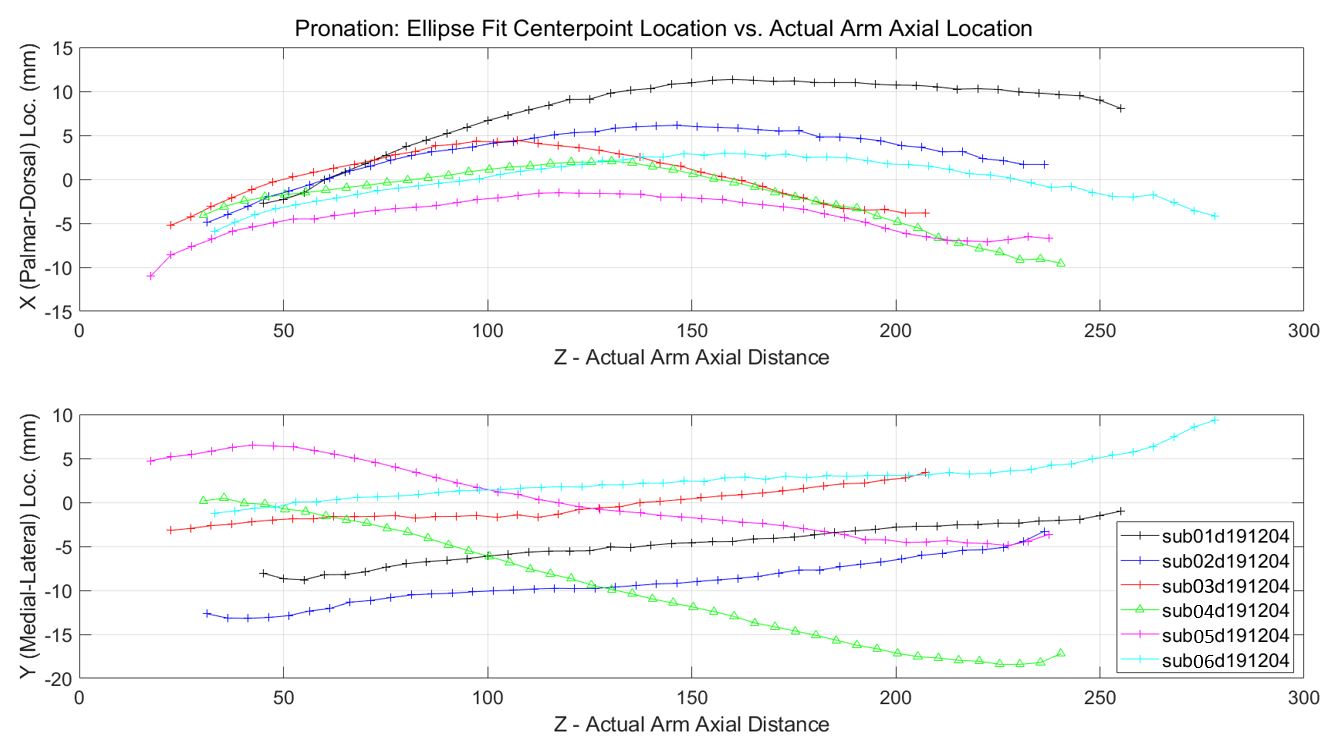

Supplement: Supplementary file 1 [file Data_Sheet_1.ZIP › SF2.2_Raw_CenterLocation_ALL_Pro.JPG]

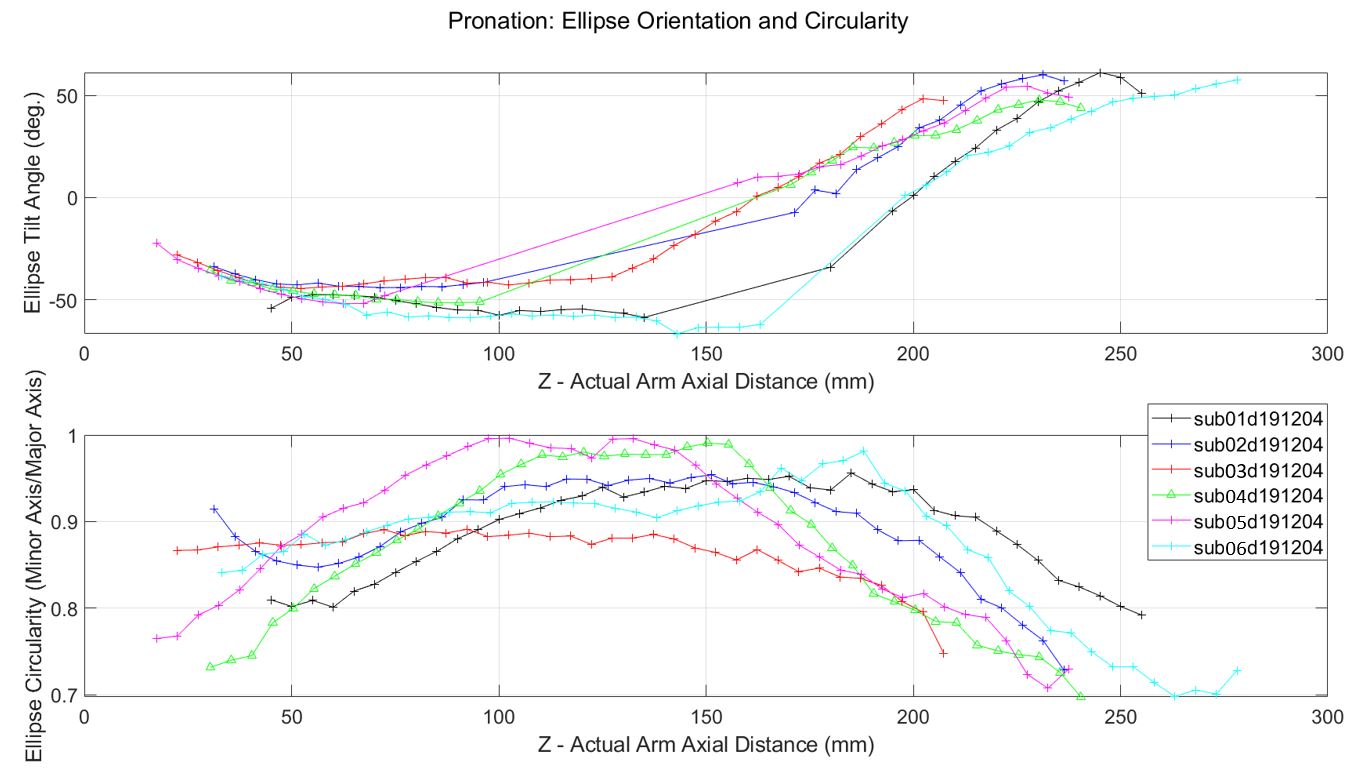

Supplement: Supplementary file 1 [file Data_Sheet_1.ZIP › SF2.3_Raw_TiltAndCircularity_ALL_Pro.JPG]

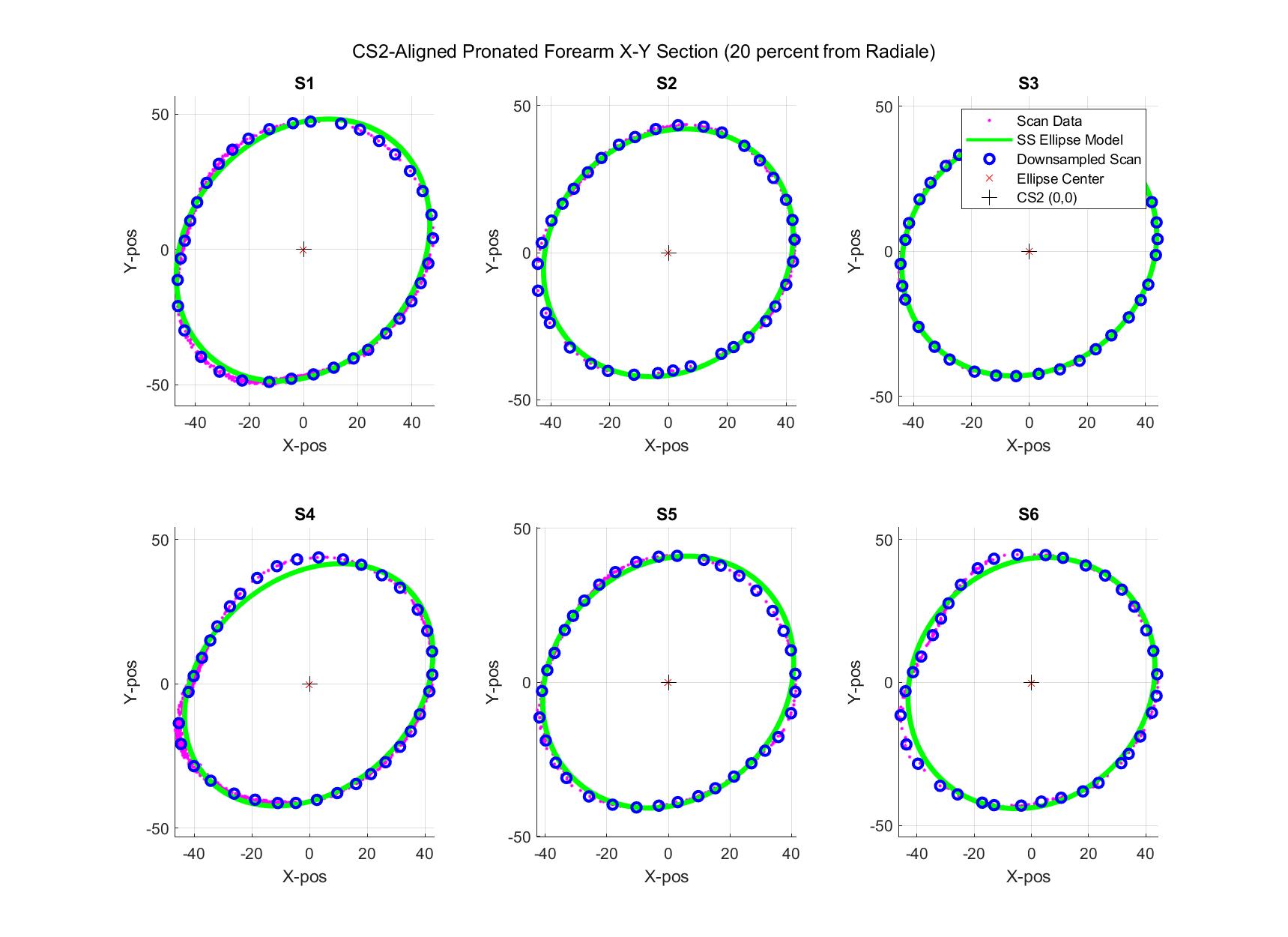

Supplement: Supplementary file 1 [file Data_Sheet_1.ZIP › SF20.1_SS_CS2_Downsampled_Cross-sectional Ellipse-Fit_20%RS_Pro.jpg]

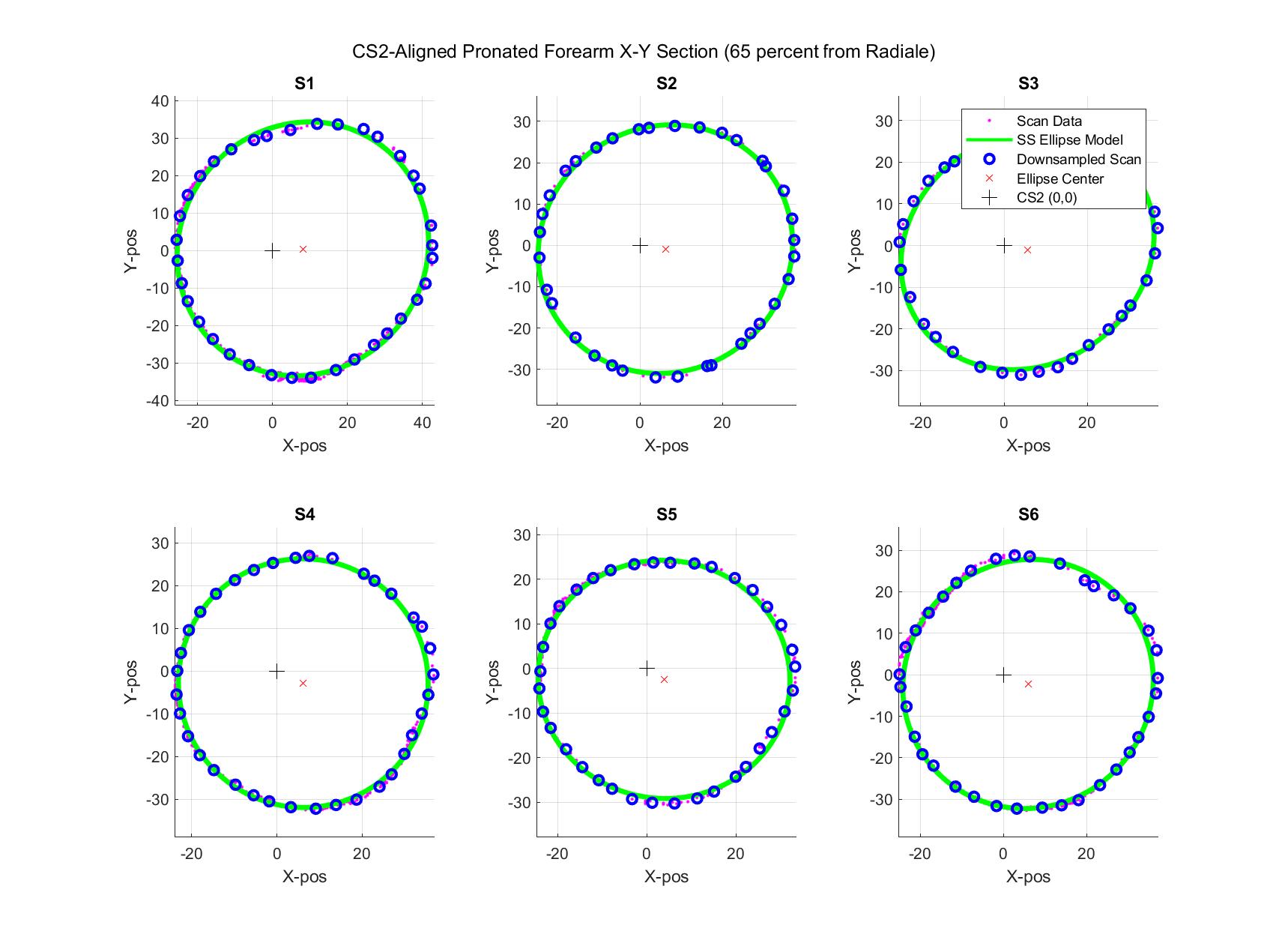

Supplement: Supplementary file 1 [file Data_Sheet_1.ZIP › SF20.10_SS_CS2_Downsampled_Cross-sectional Ellipse-Fit_65%RS_Pro.jpg]

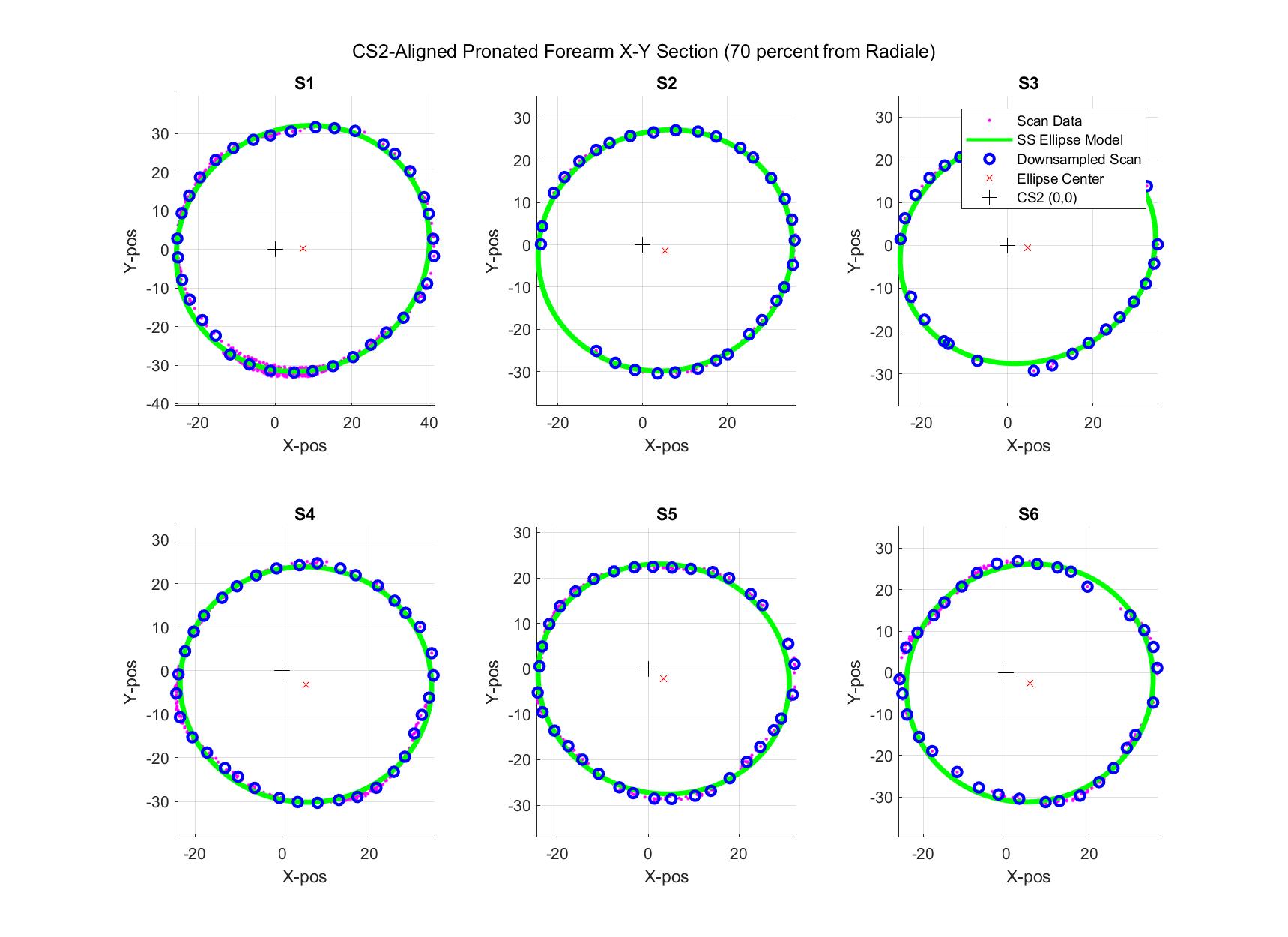

Supplement: Supplementary file 1 [file Data_Sheet_1.ZIP › SF20.11_SS_CS2_Downsampled_Cross-sectional Ellipse-Fit_70%RS_Pro.jpg]

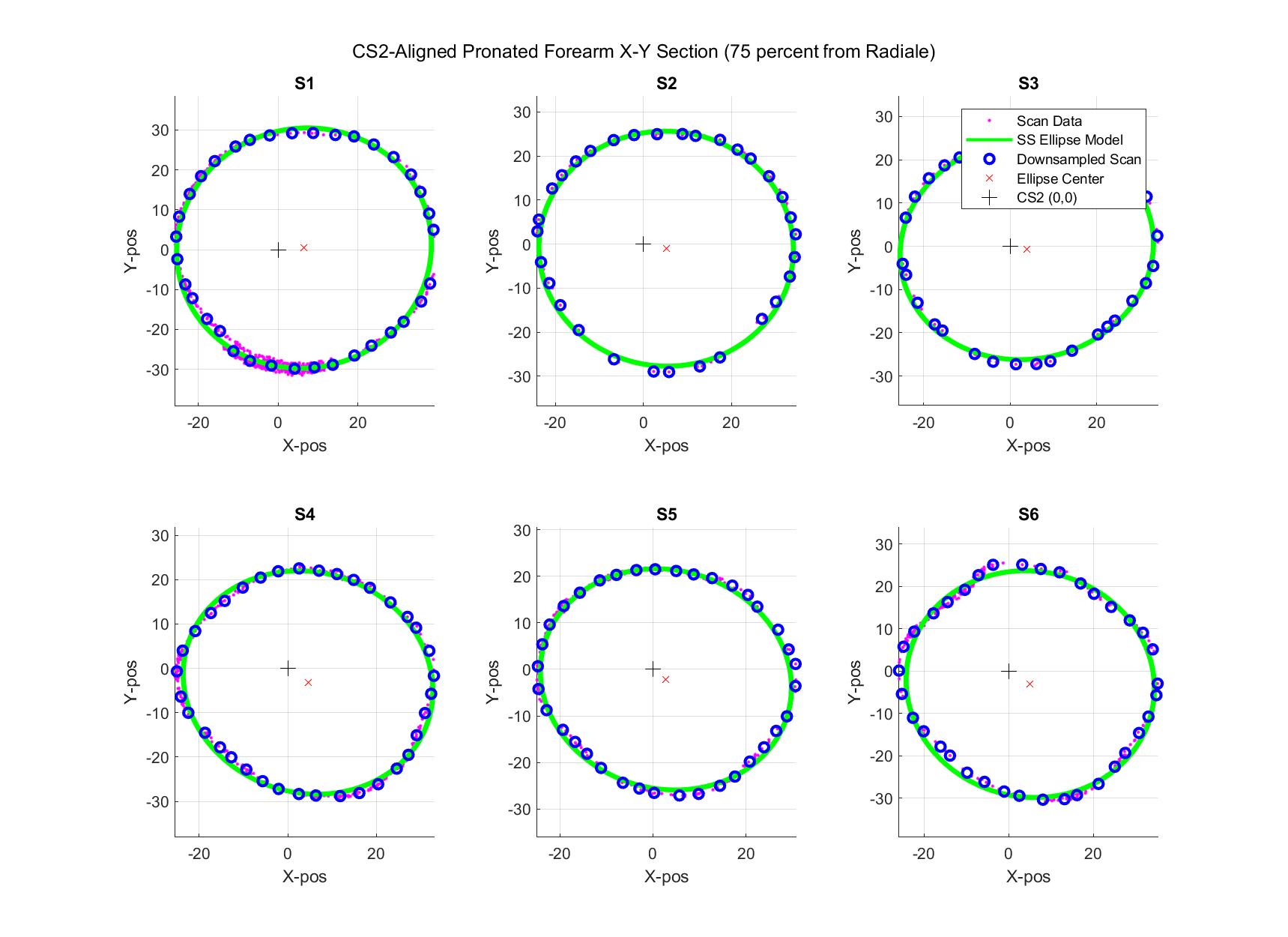

Supplement: Supplementary file 1 [file Data_Sheet_1.ZIP › SF20.12_SS_CS2_Downsampled_Cross-sectional Ellipse-Fit_75%RS_Pro.jpg]

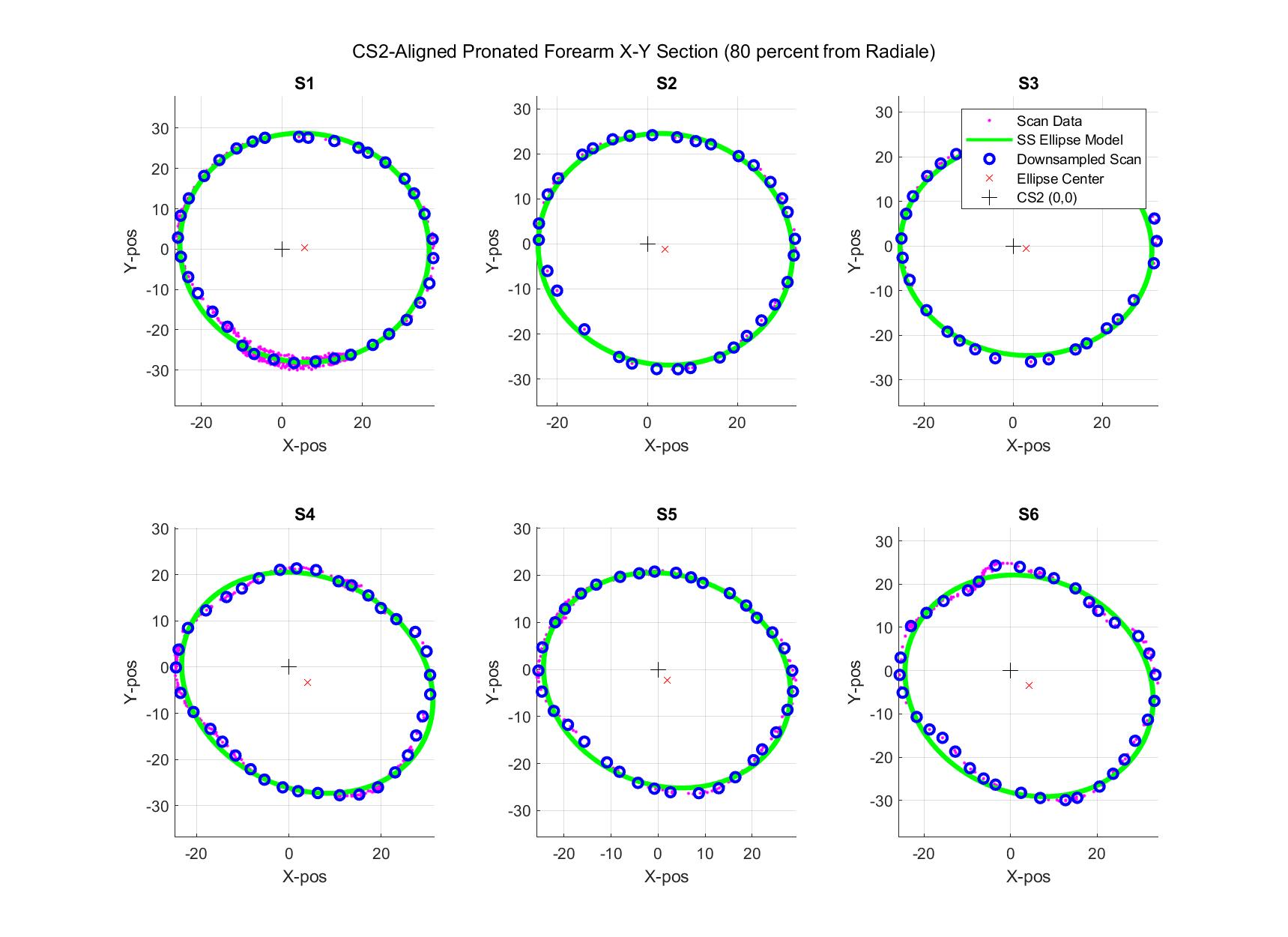

Supplement: Supplementary file 1 [file Data_Sheet_1.ZIP › SF20.13_SS_CS2_Downsampled_Cross-sectional Ellipse-Fit_80%RS_Pro.jpg]

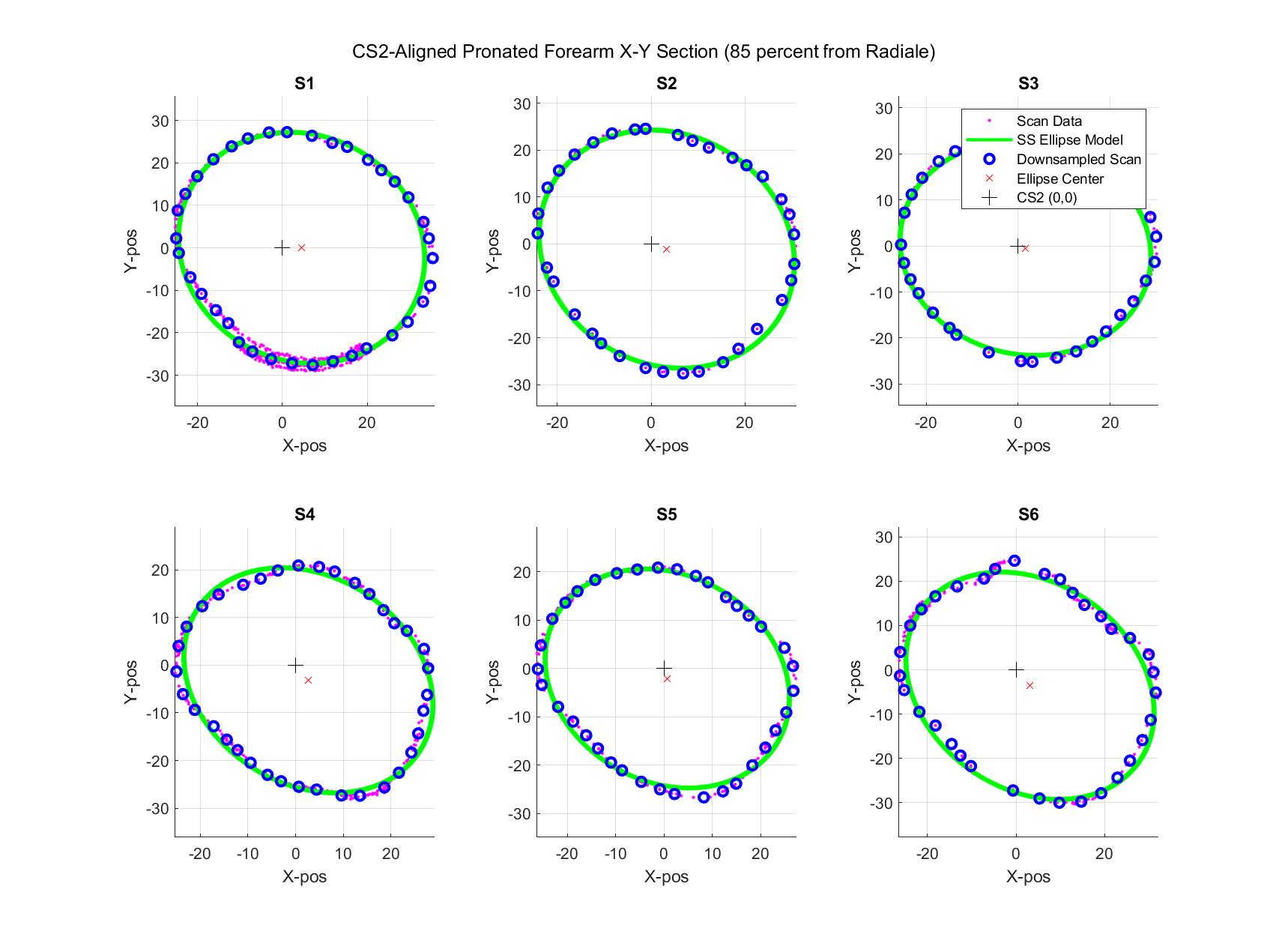

Supplement: Supplementary file 1 [file Data_Sheet_1.ZIP › SF20.14_SS_CS2_Downsampled_Cross-sectional Ellipse-Fit_85%RS_Pro.jpg]

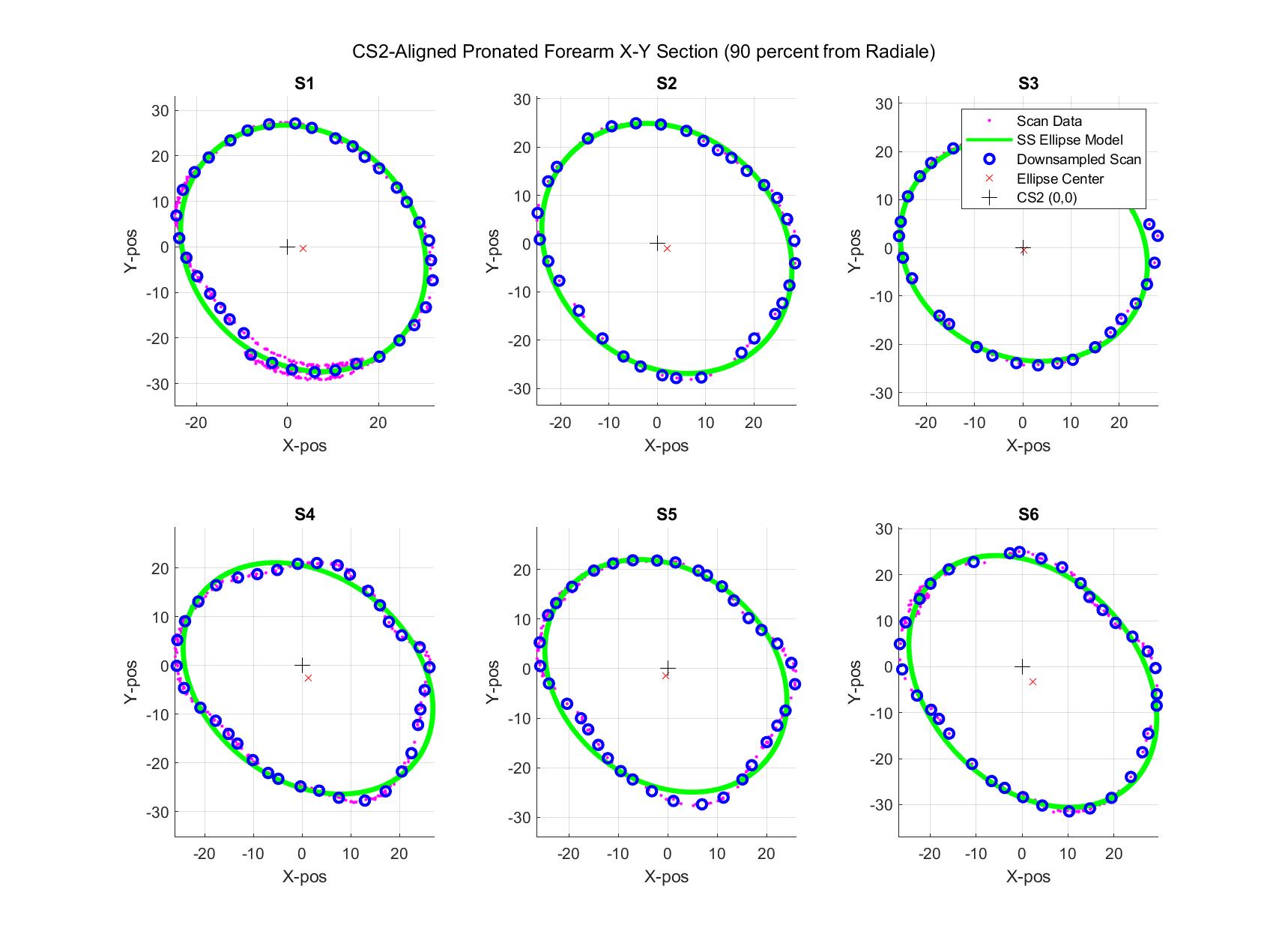

Supplement: Supplementary file 1 [file Data_Sheet_1.ZIP › SF20.15_SS_CS2_Downsampled_Cross-sectional Ellipse-Fit_90%RS_Pro.jpg]

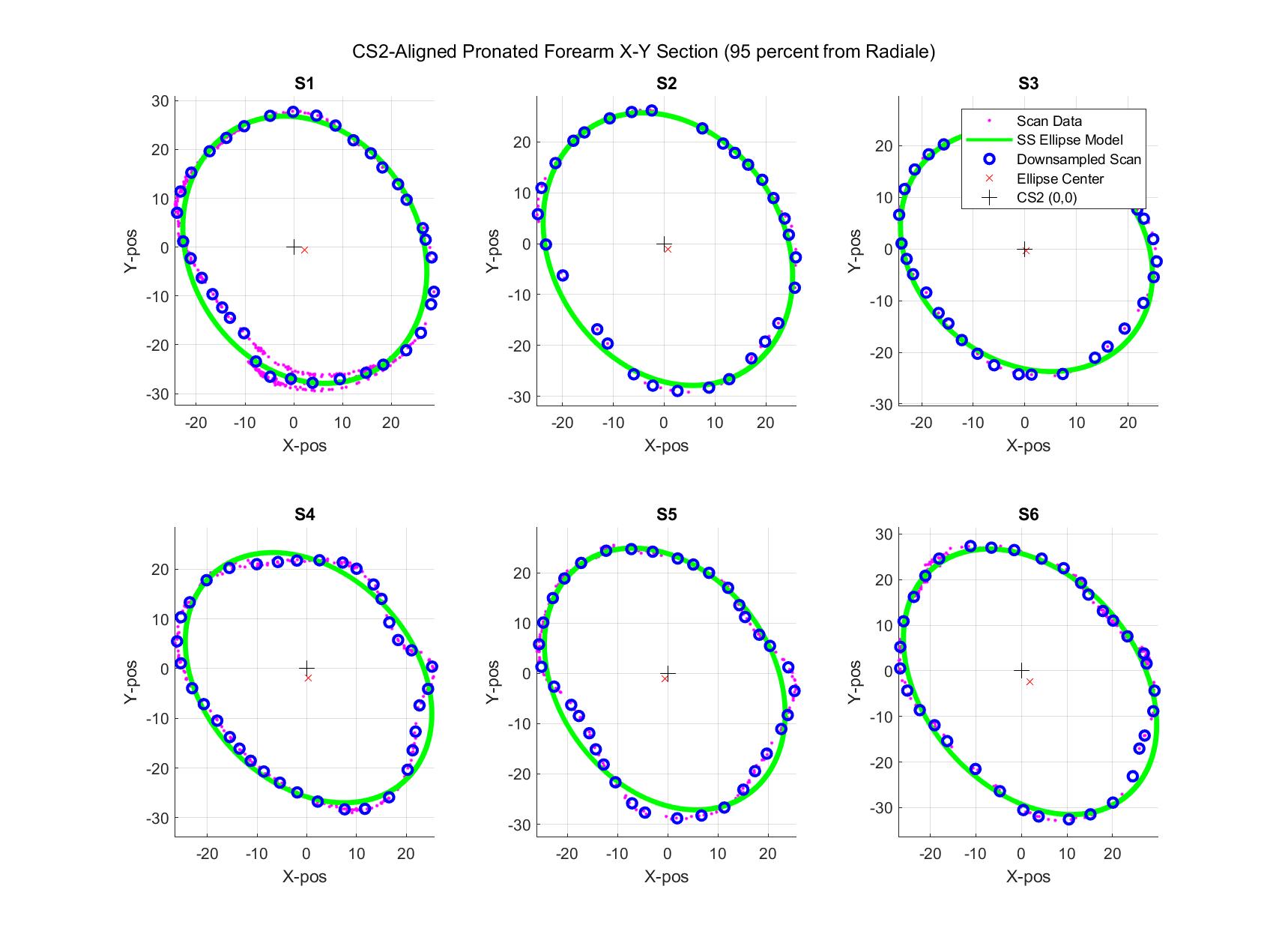

Supplement: Supplementary file 1 [file Data_Sheet_1.ZIP › SF20.16_SS_CS2_Downsampled_Cross-sectional Ellipse-Fit_95%RS_Pro.jpg]

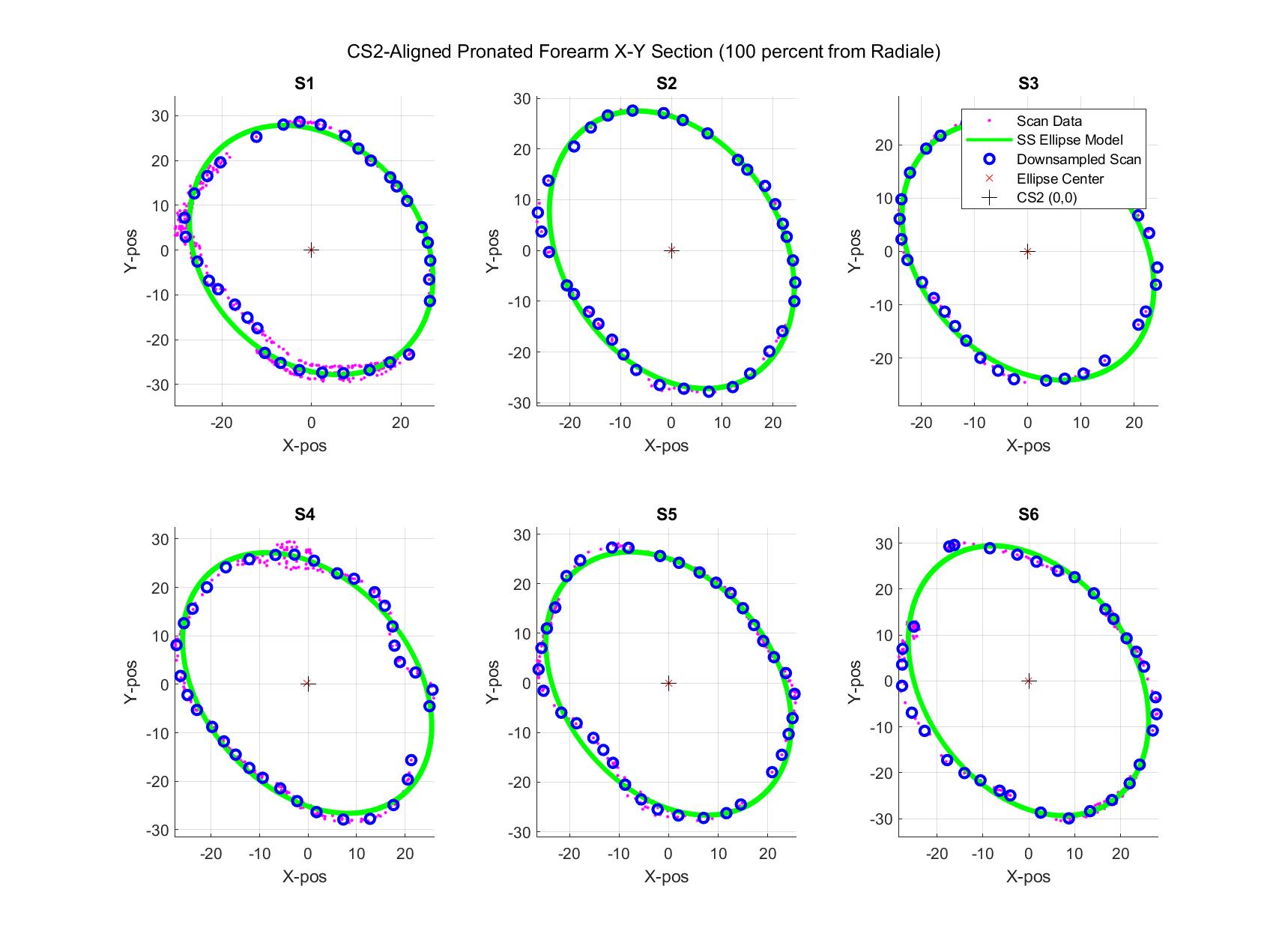

Supplement: Supplementary file 1 [file Data_Sheet_1.ZIP › SF20.17_SS_CS2_Downsampled_Cross-sectional Ellipse-Fit_100%RS_Pro.jpg]

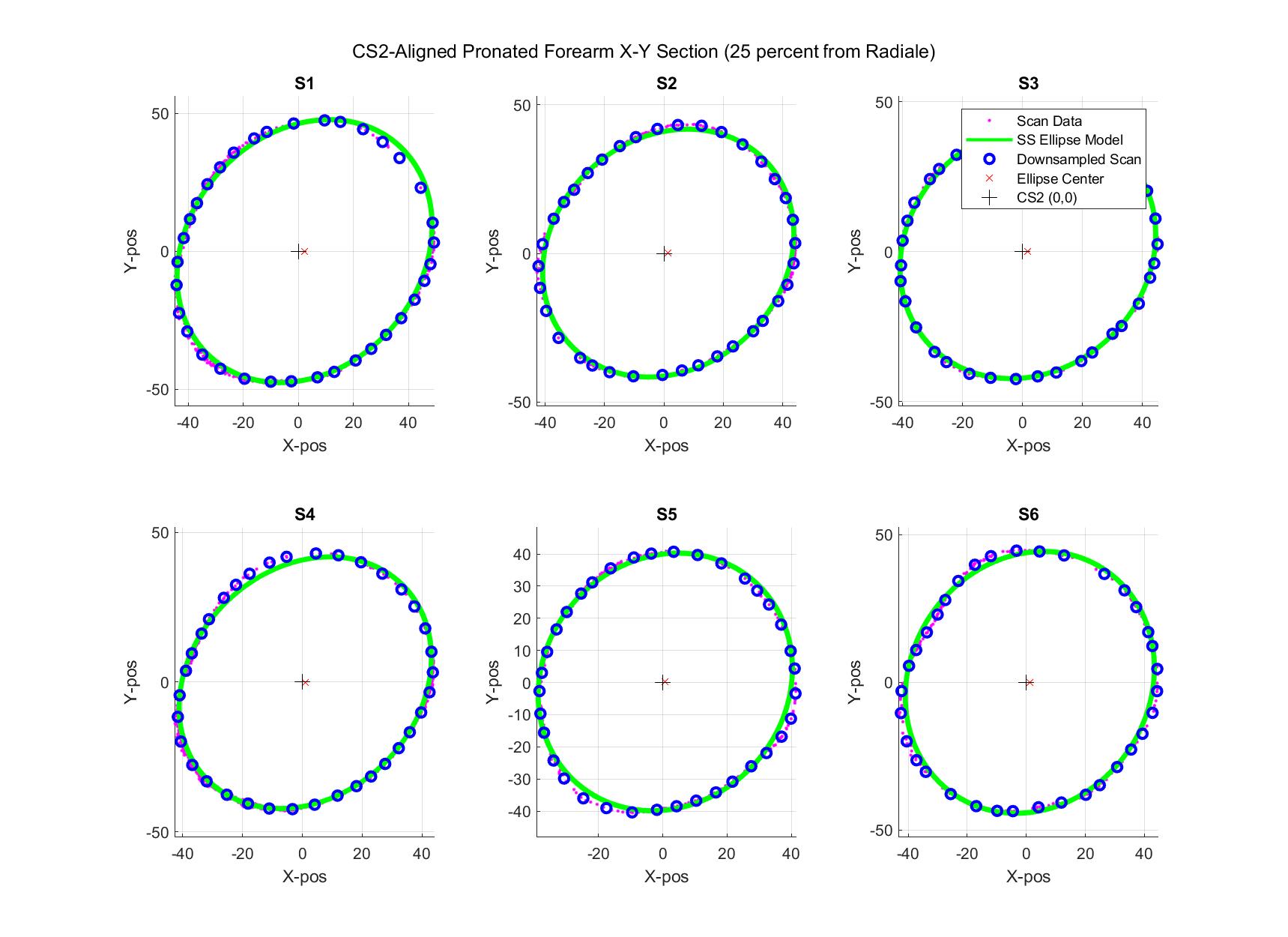

Supplement: Supplementary file 1 [file Data_Sheet_1.ZIP › SF20.2_SS_CS2_Downsampled_Cross-sectional Ellipse-Fit_25%RS_Pro.jpg]

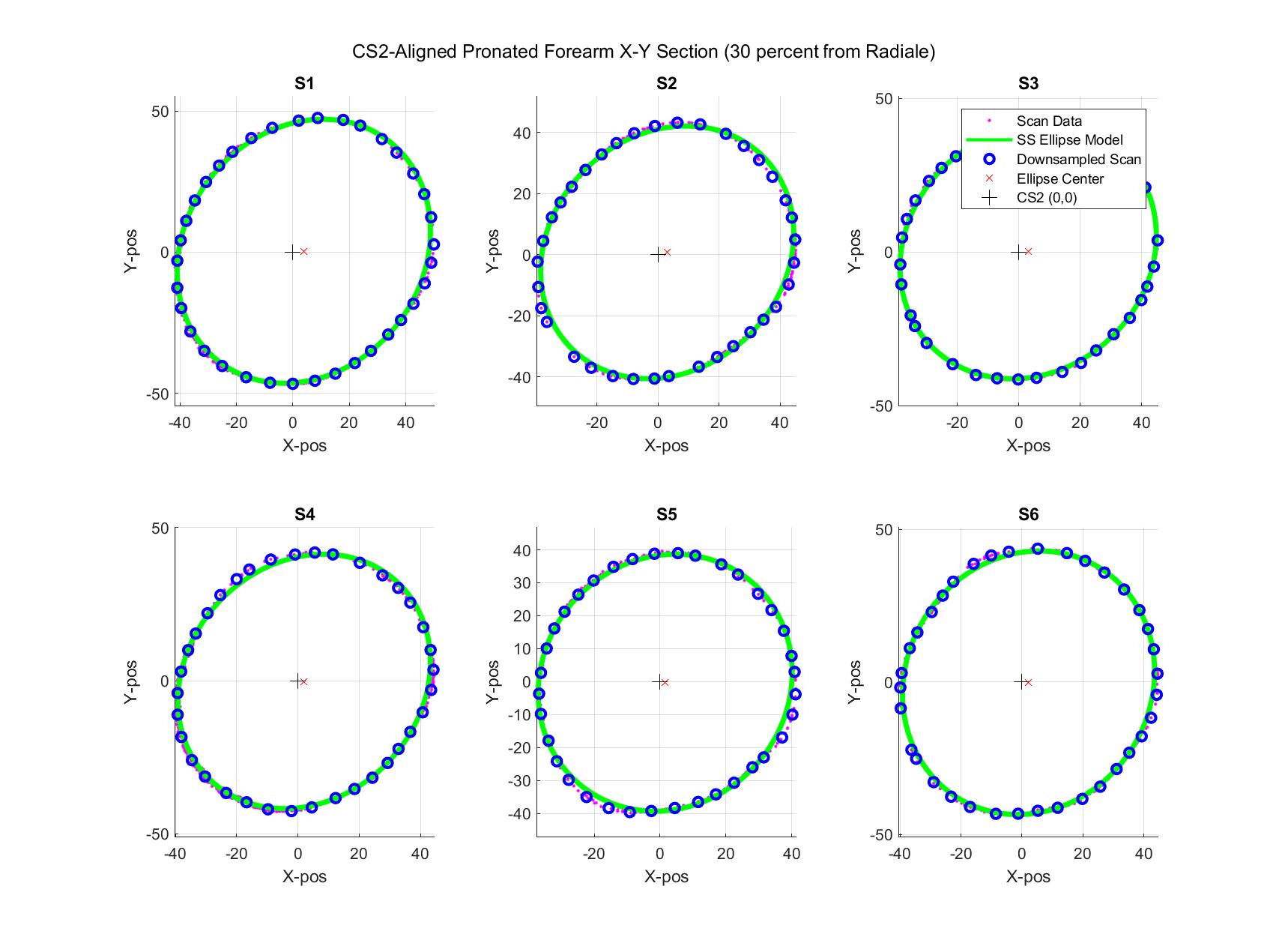

Supplement: Supplementary file 1 [file Data_Sheet_1.ZIP › SF20.3_SS_CS2_Downsampled_Cross-sectional Ellipse-Fit_30%RS_Pro.jpg]

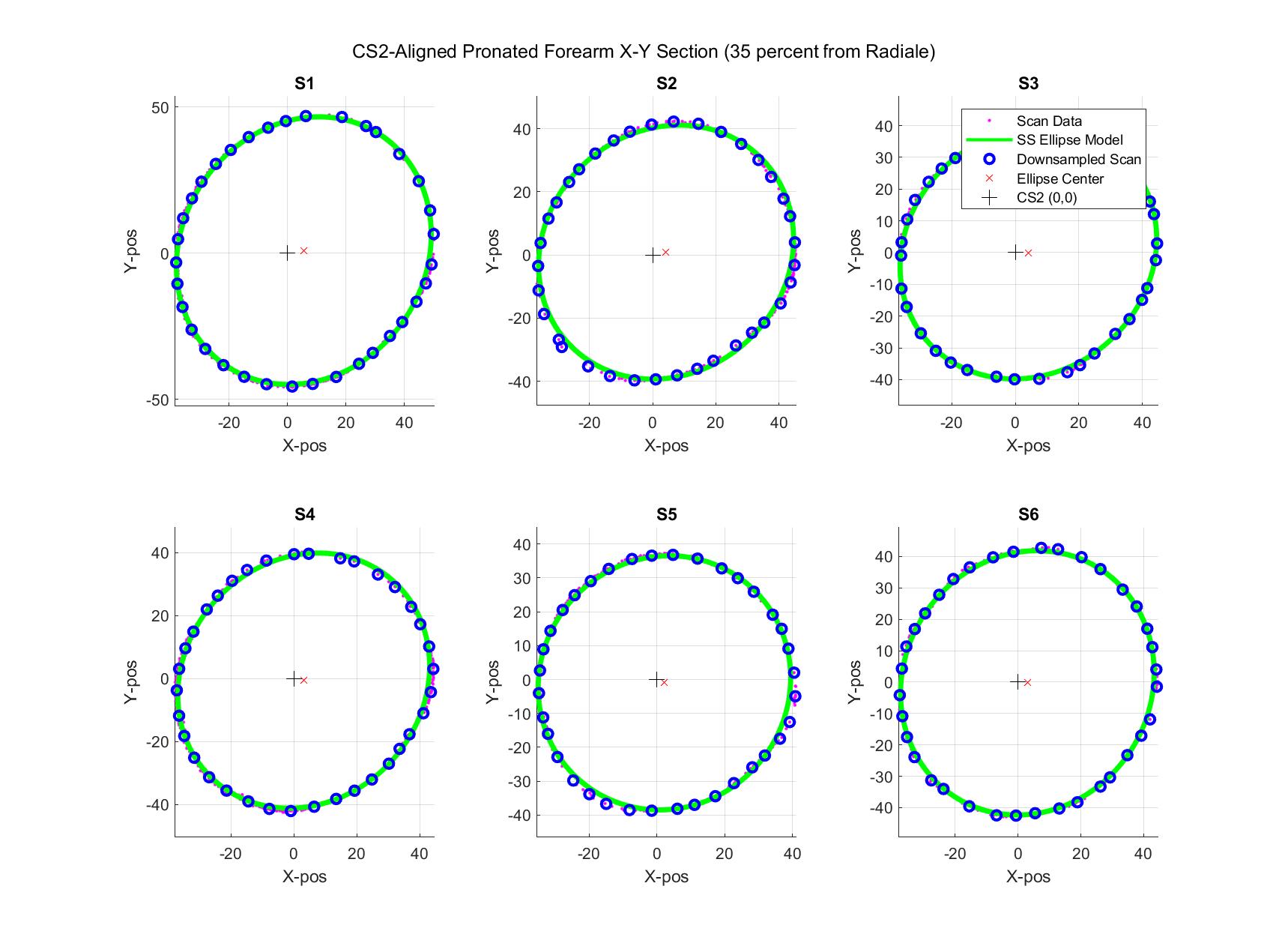

Supplement: Supplementary file 1 [file Data_Sheet_1.ZIP › SF20.4_SS_CS2_Downsampled_Cross-sectional Ellipse-Fit_35%RS_Pro.jpg]

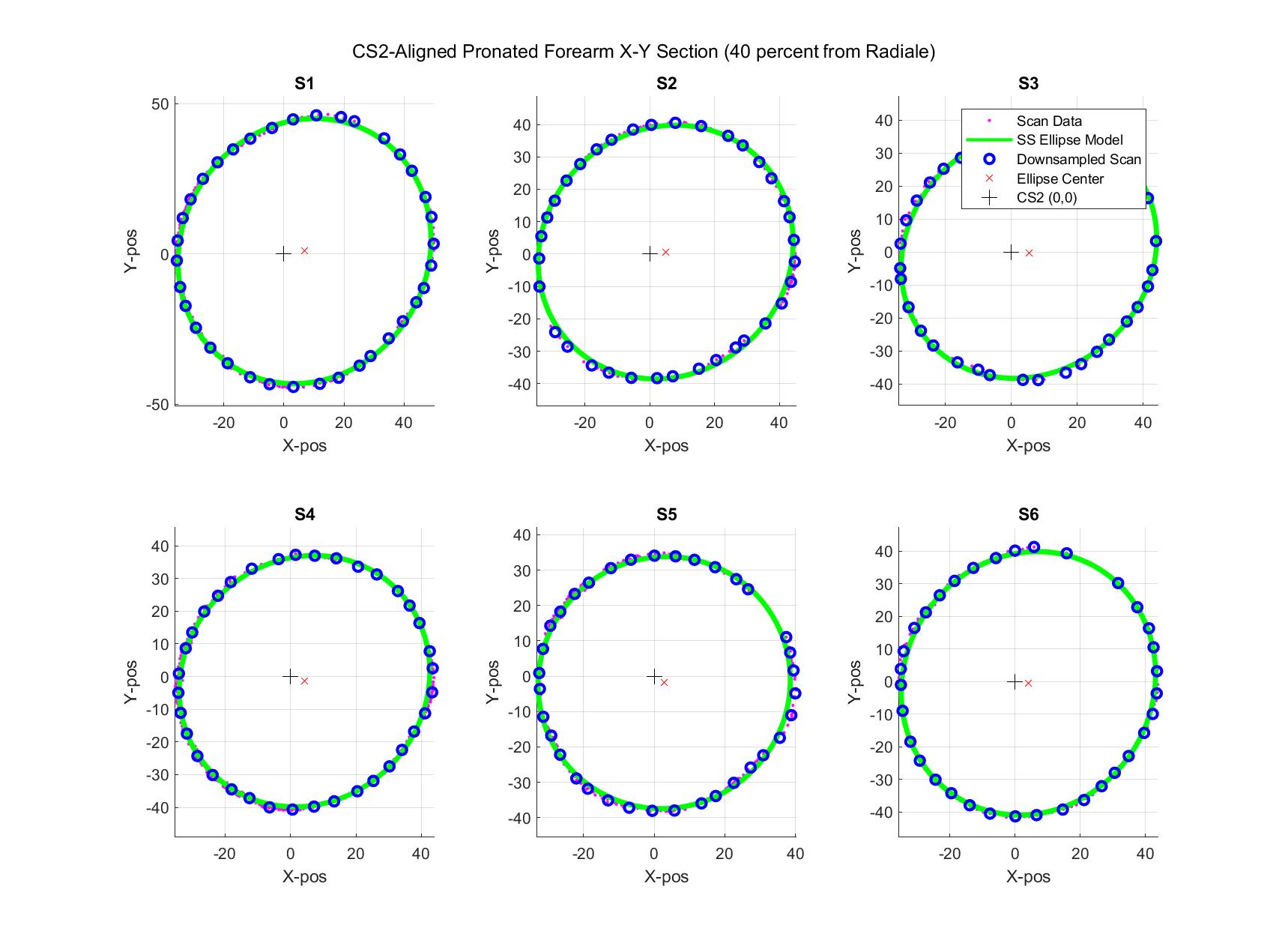

Supplement: Supplementary file 1 [file Data_Sheet_1.ZIP › SF20.5_SS_CS2_Downsampled_Cross-sectional Ellipse-Fit_40%RS_Pro.jpg]

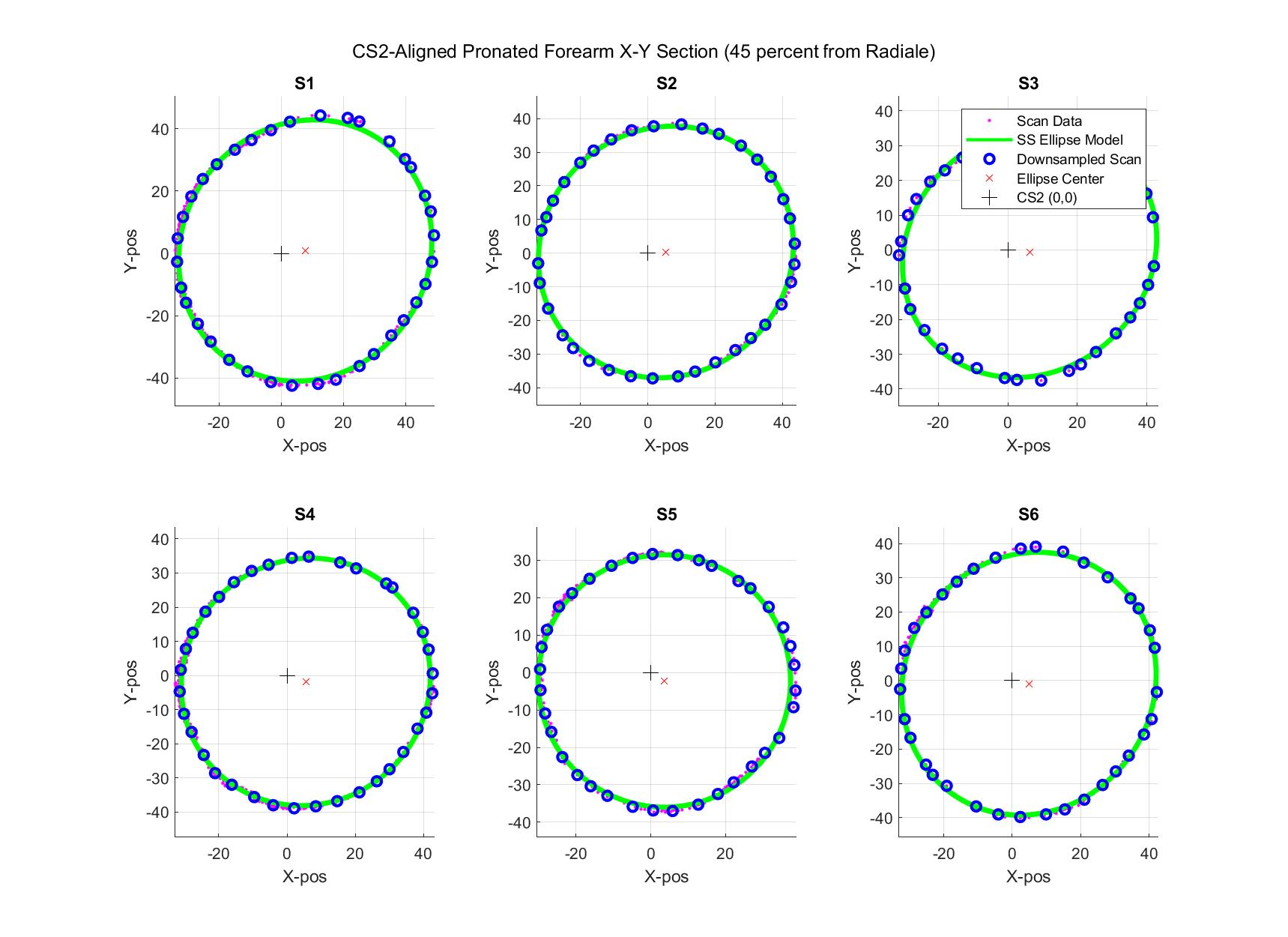

Supplement: Supplementary file 1 [file Data_Sheet_1.ZIP › SF20.6_SS_CS2_Downsampled_Cross-sectional Ellipse-Fit_45%RS_Pro.jpg]

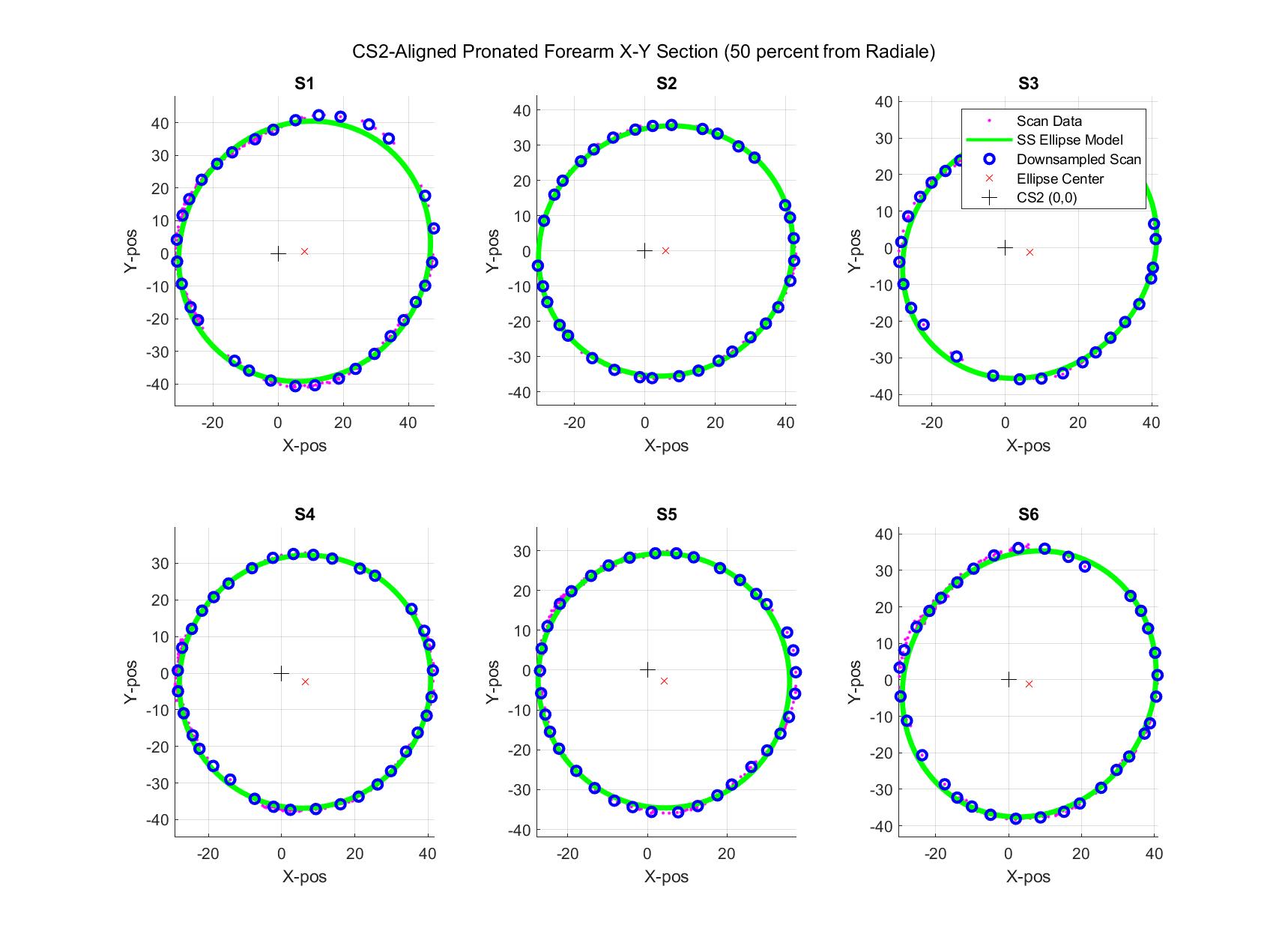

Supplement: Supplementary file 1 [file Data_Sheet_1.ZIP › SF20.7_SS_CS2_Downsampled_Cross-sectional Ellipse-Fit_50%RS_Pro.jpg]

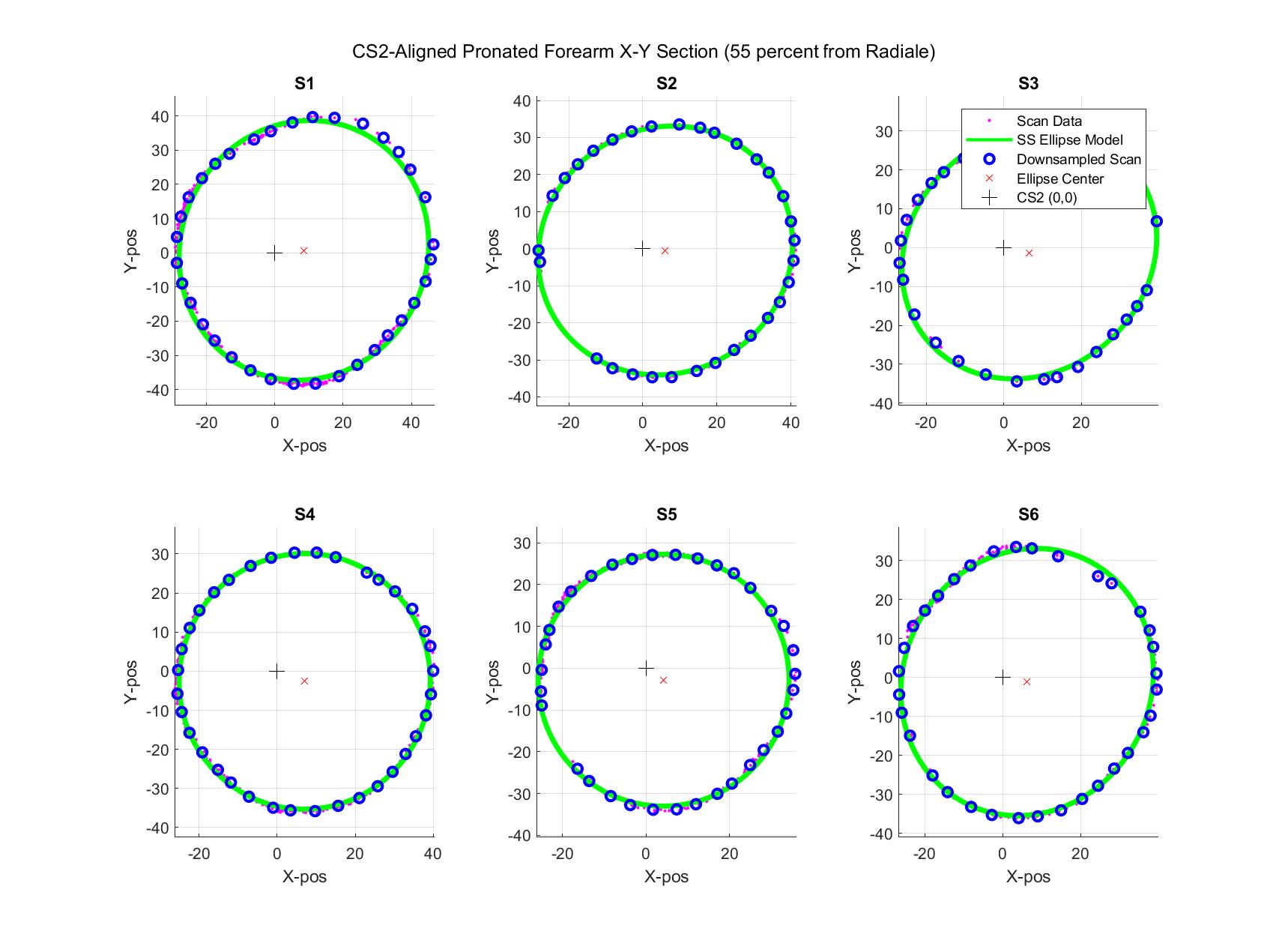

Supplement: Supplementary file 1 [file Data_Sheet_1.ZIP › SF20.8_SS_CS2_Downsampled_Cross-sectional Ellipse-Fit_55%RS_Pro.jpg]

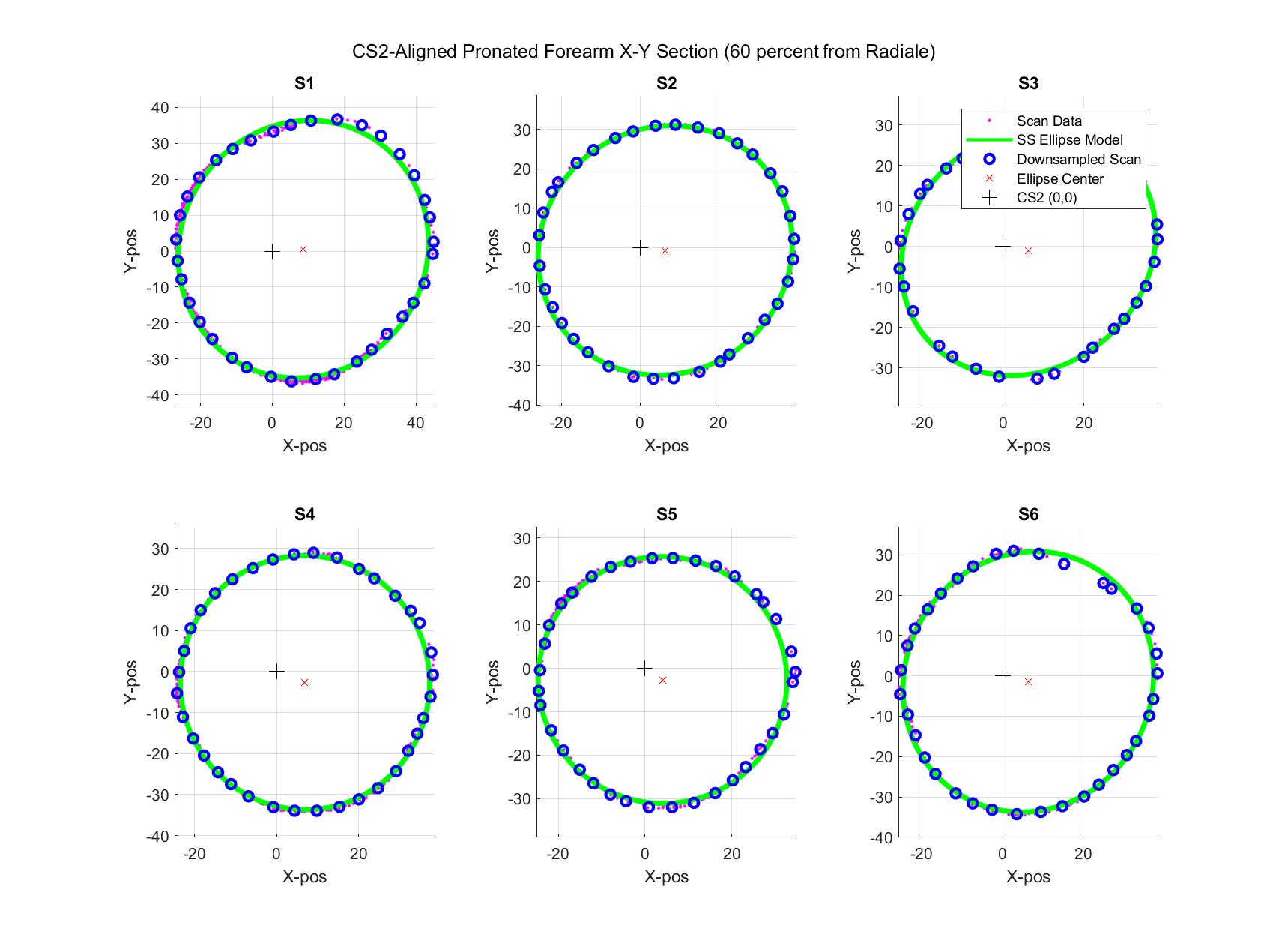

Supplement: Supplementary file 1 [file Data_Sheet_1.ZIP › SF20.9_SS_CS2_Downsampled_Cross-sectional Ellipse-Fit_60%RS_Pro.jpg]

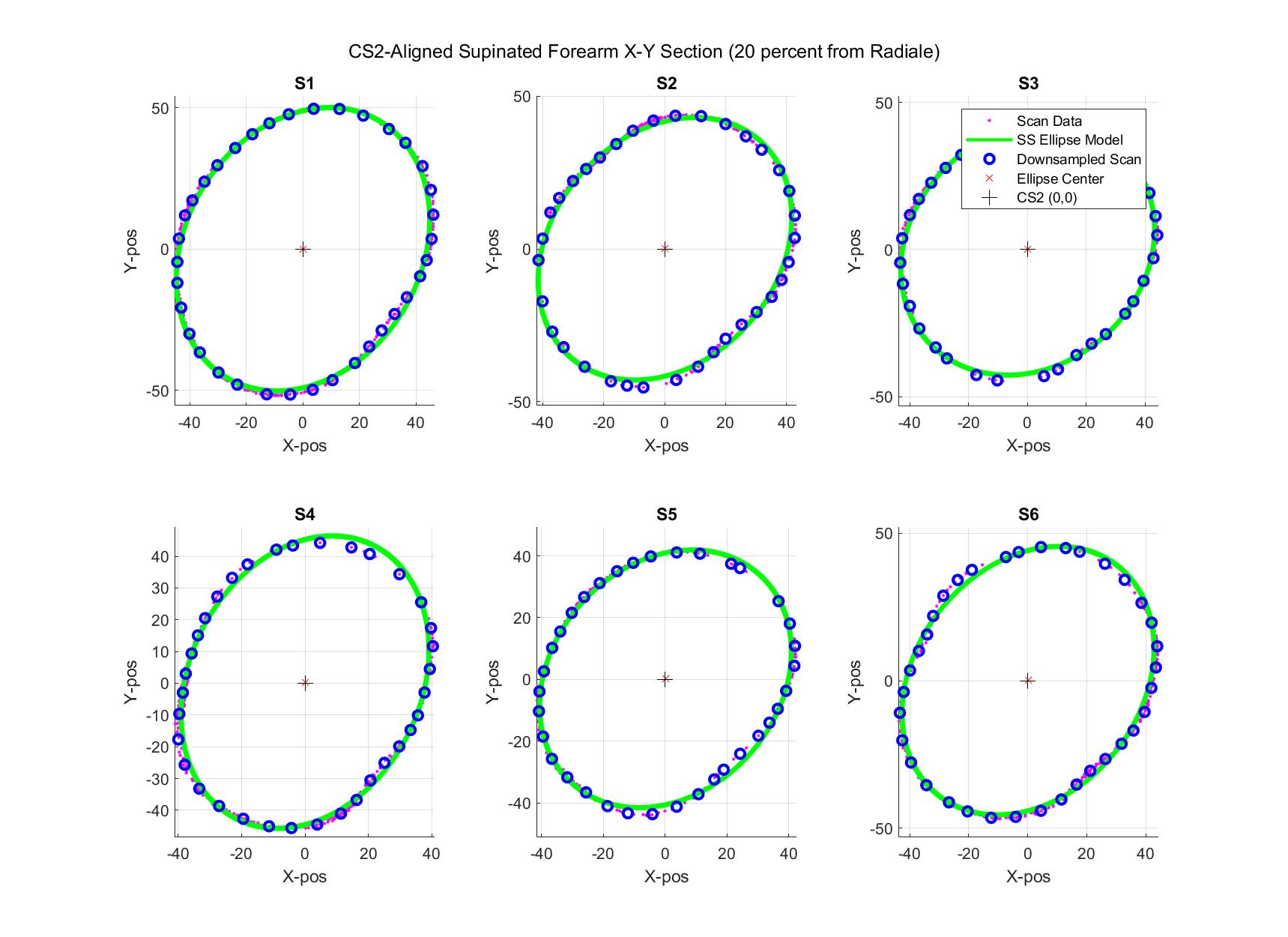

Supplement: Supplementary file 1 [file Data_Sheet_1.ZIP › SF21.1_SS_CS2_Downsampled_Cross-sectional Ellipse-Fit_20%RS_Sup.jpg]

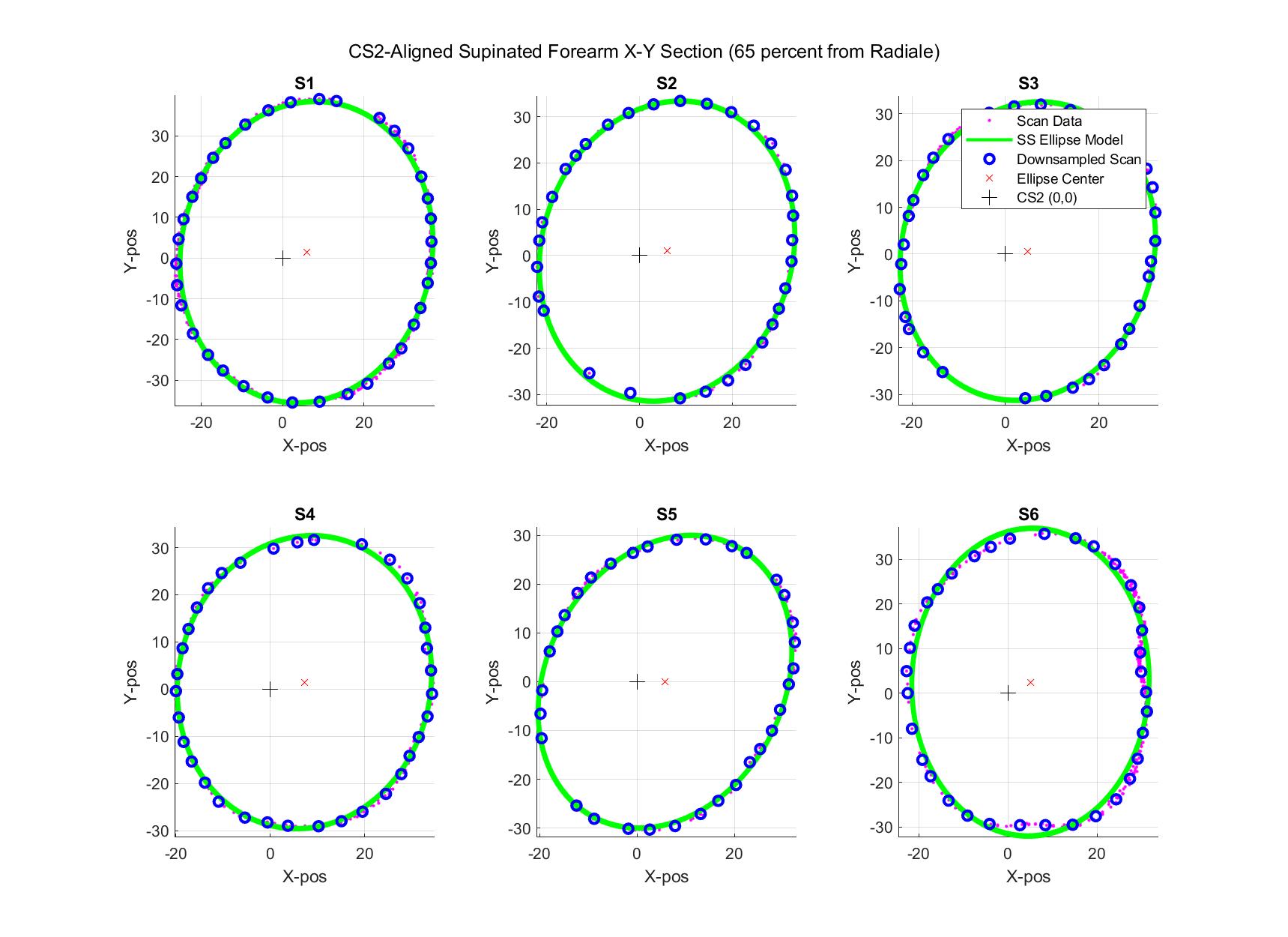

Supplement: Supplementary file 1 [file Data_Sheet_1.ZIP › SF21.10_SS_CS2_Downsampled_Cross-sectional Ellipse-Fit_65%RS_Sup.jpg]

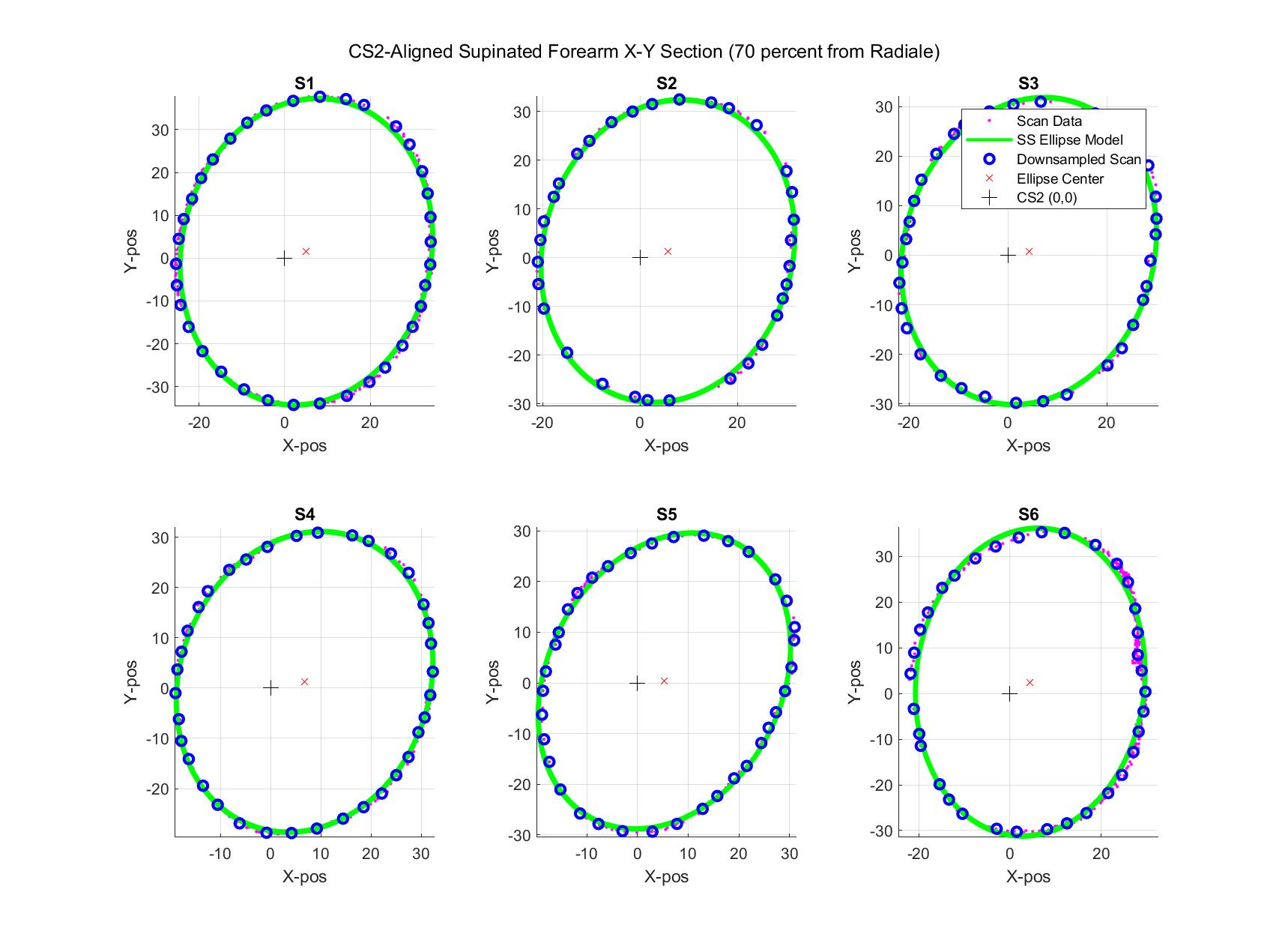

Supplement: Supplementary file 1 [file Data_Sheet_1.ZIP › SF21.11_SS_CS2_Downsampled_Cross-sectional Ellipse-Fit_70%RS_Sup.jpg]

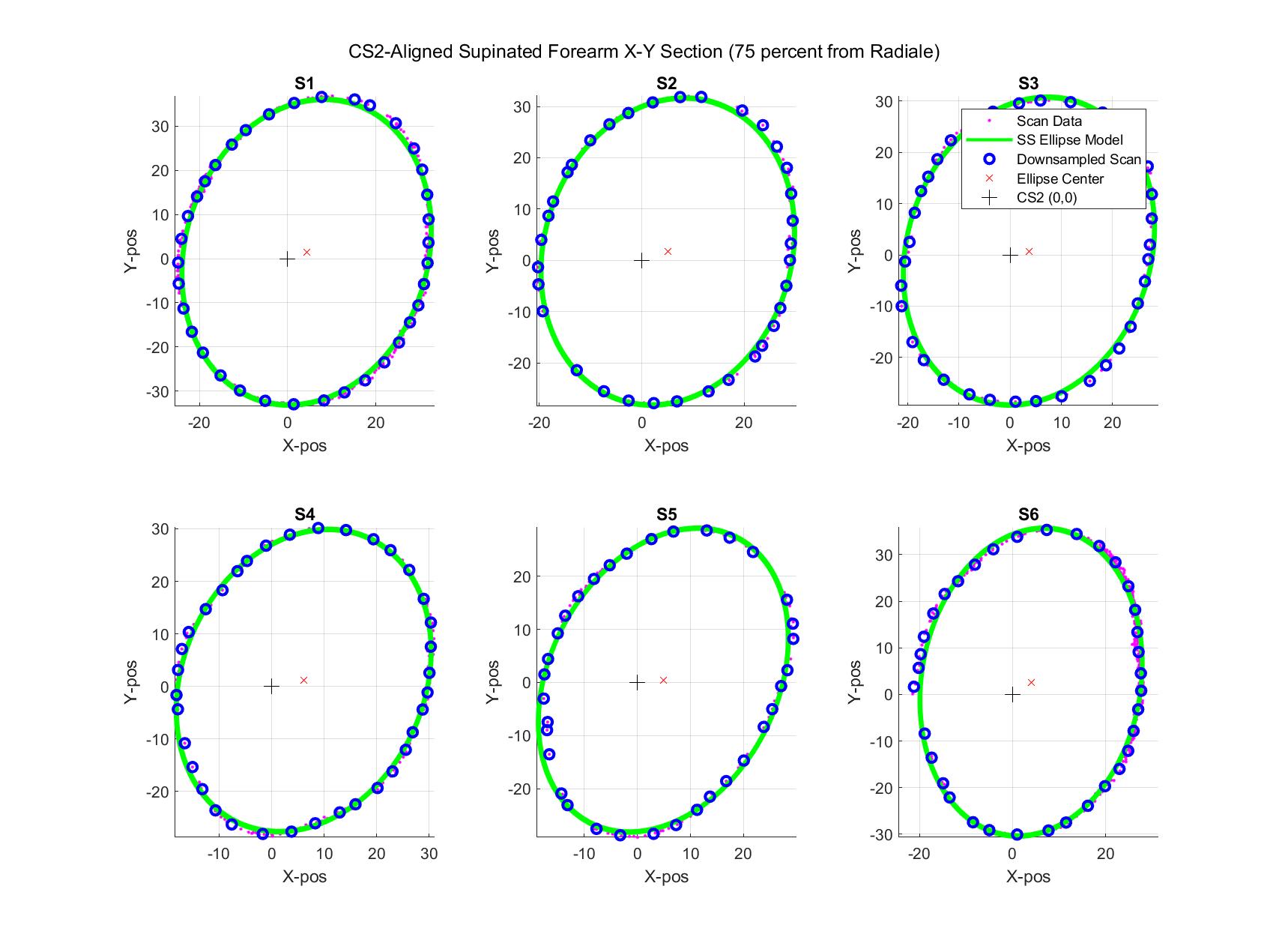

Supplement: Supplementary file 1 [file Data_Sheet_1.ZIP › SF21.12_SS_CS2_Downsampled_Cross-sectional Ellipse-Fit_75%RS_Sup.jpg]

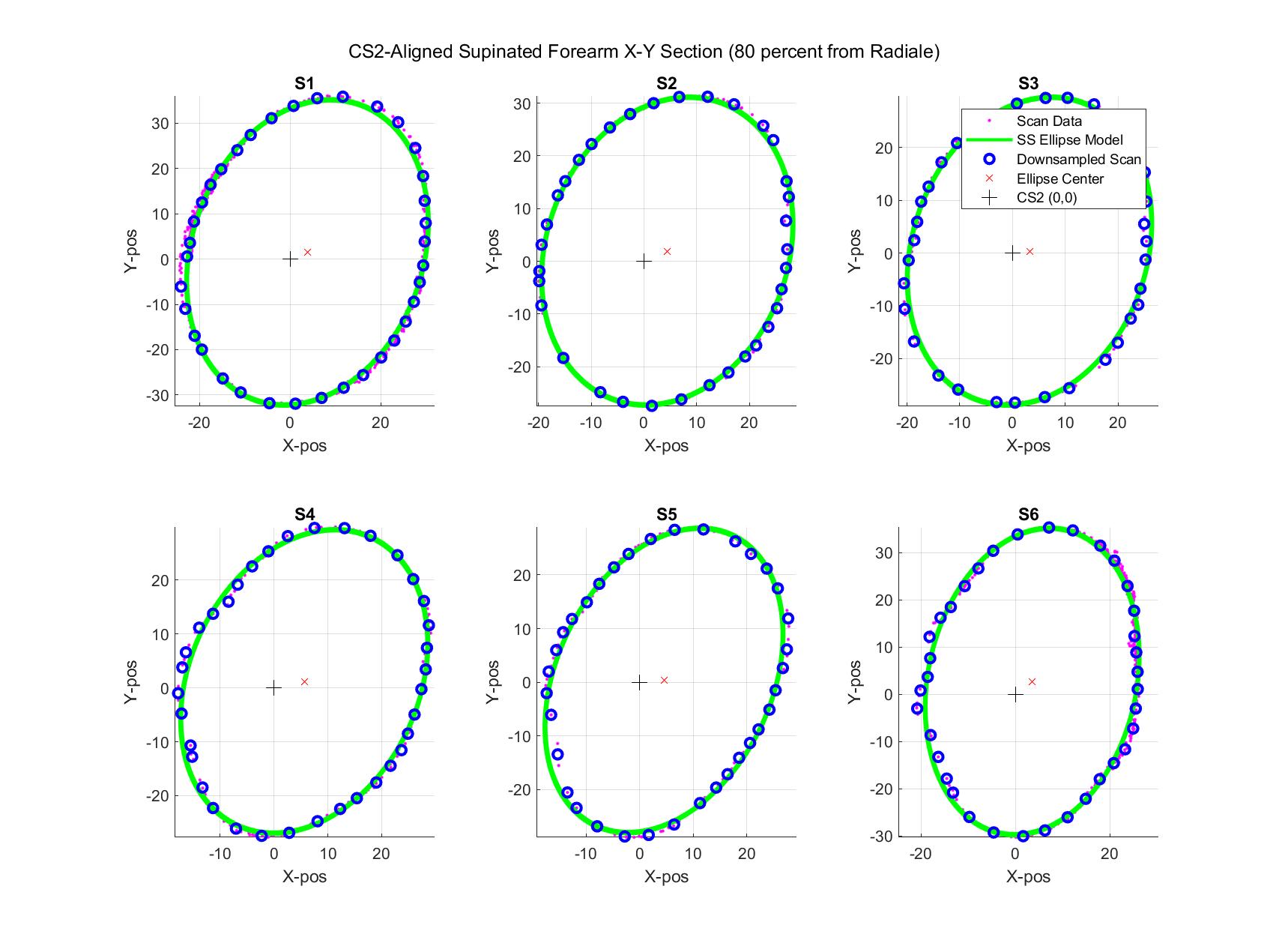

Supplement: Supplementary file 1 [file Data_Sheet_1.ZIP › SF21.13_SS_CS2_Downsampled_Cross-sectional Ellipse-Fit_80%RS_Sup.jpg]

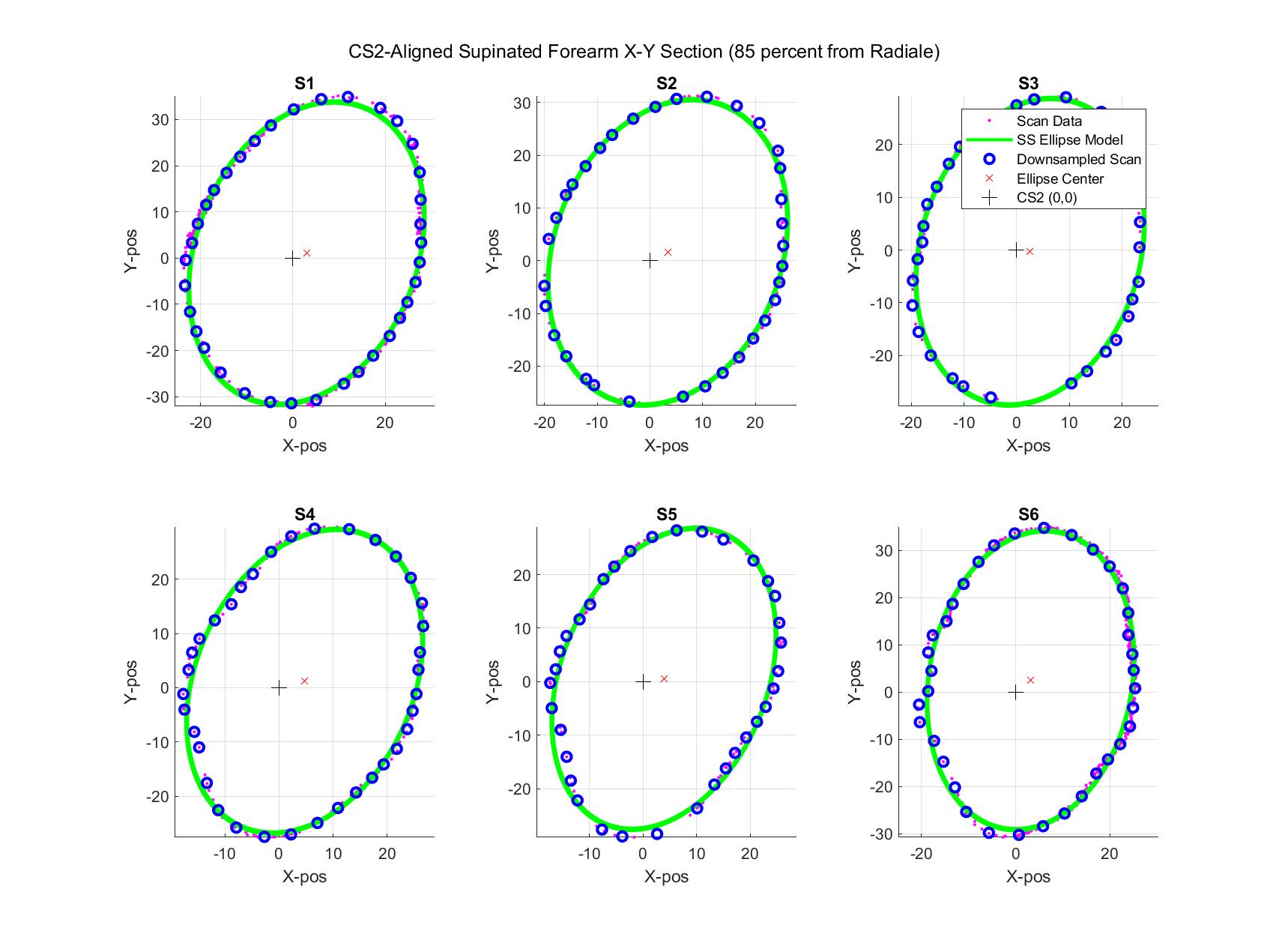

Supplement: Supplementary file 1 [file Data_Sheet_1.ZIP › SF21.14_SS_CS2_Downsampled_Cross-sectional Ellipse-Fit_85%RS_Sup.jpg]

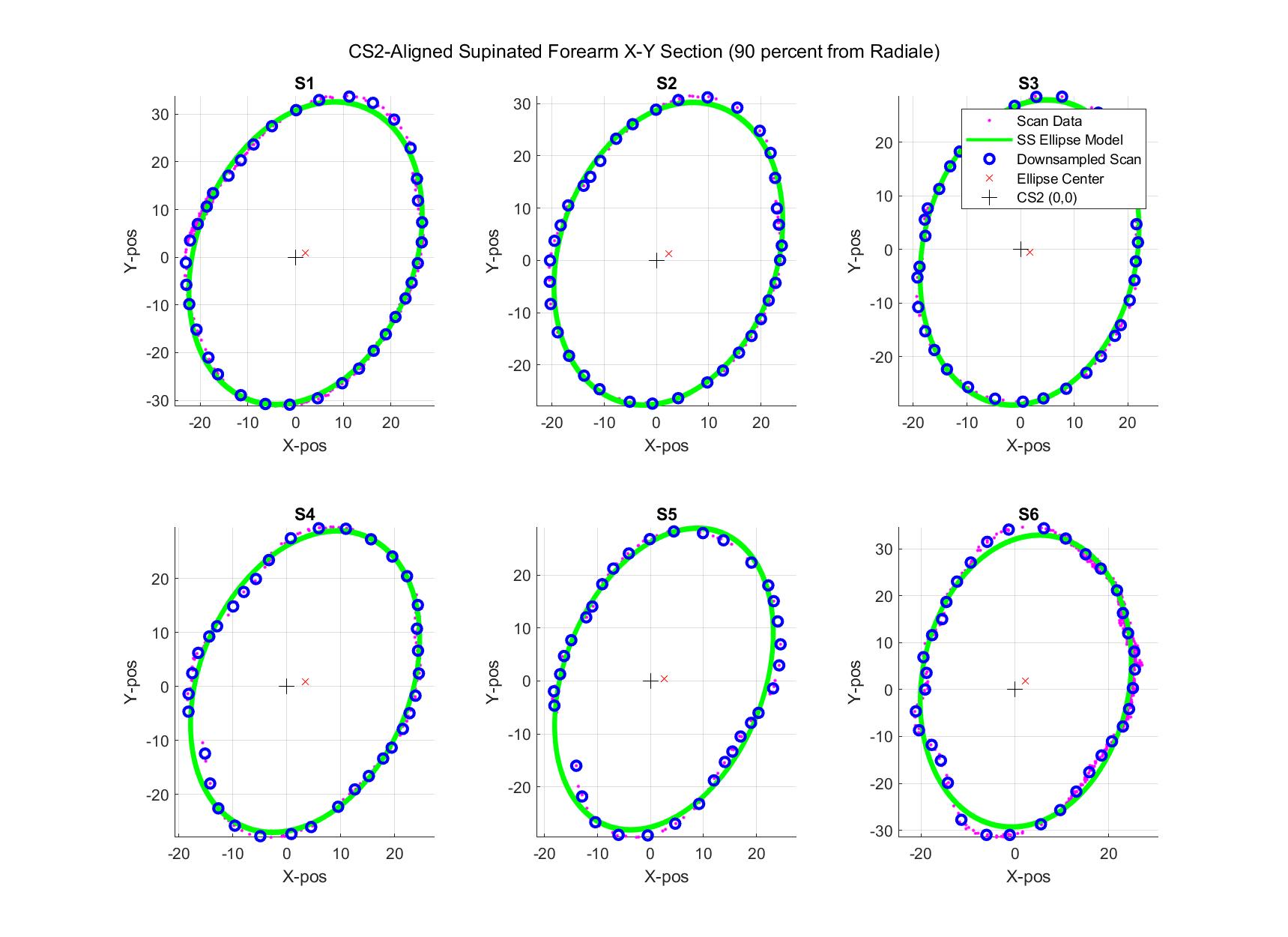

Supplement: Supplementary file 1 [file Data_Sheet_1.ZIP › SF21.15_SS_CS2_Downsampled_Cross-sectional Ellipse-Fit_90%RS_Sup.jpg]

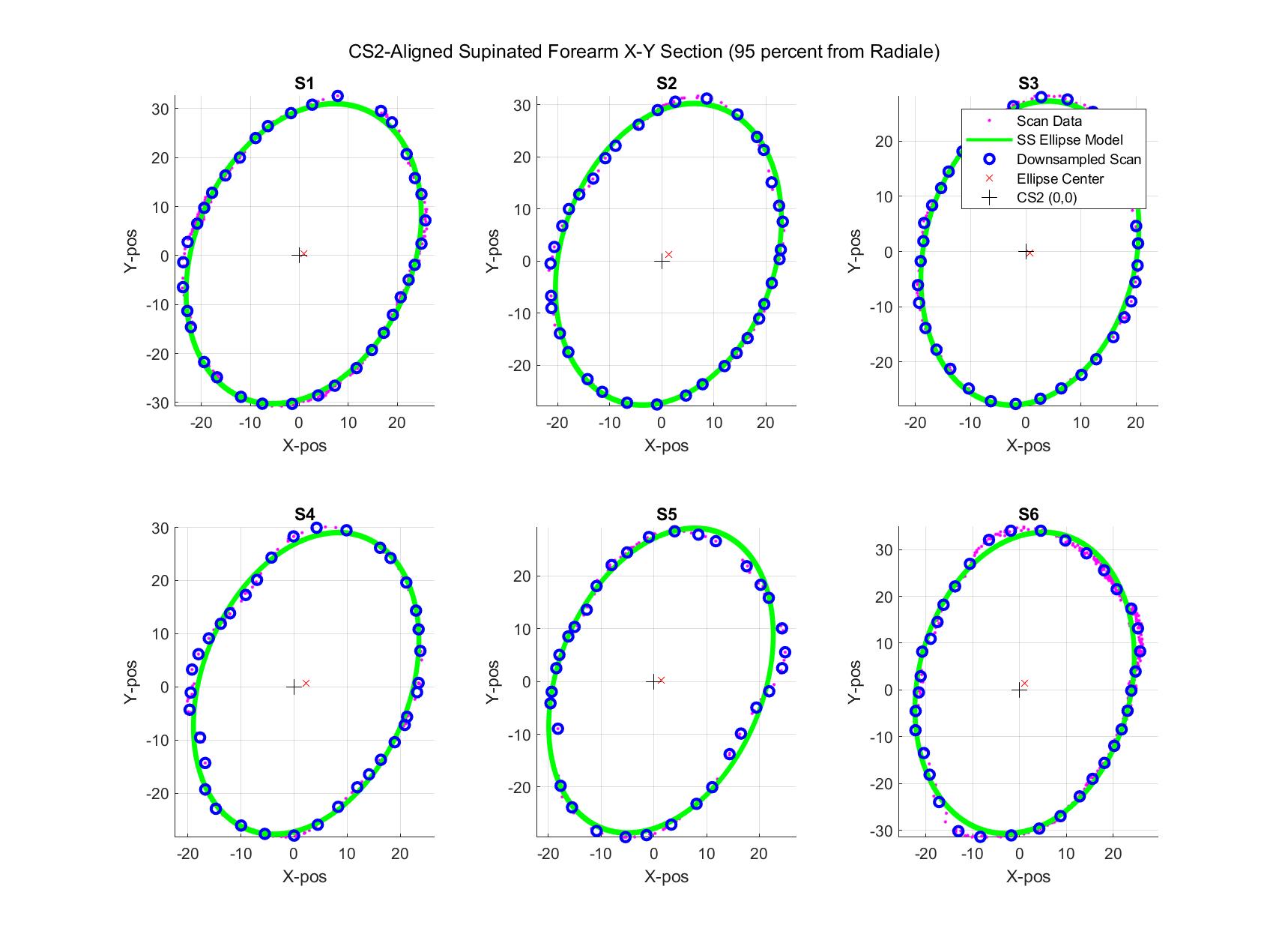

Supplement: Supplementary file 1 [file Data_Sheet_1.ZIP › SF21.16_SS_CS2_Downsampled_Cross-sectional Ellipse-Fit_95%RS_Sup.jpg]

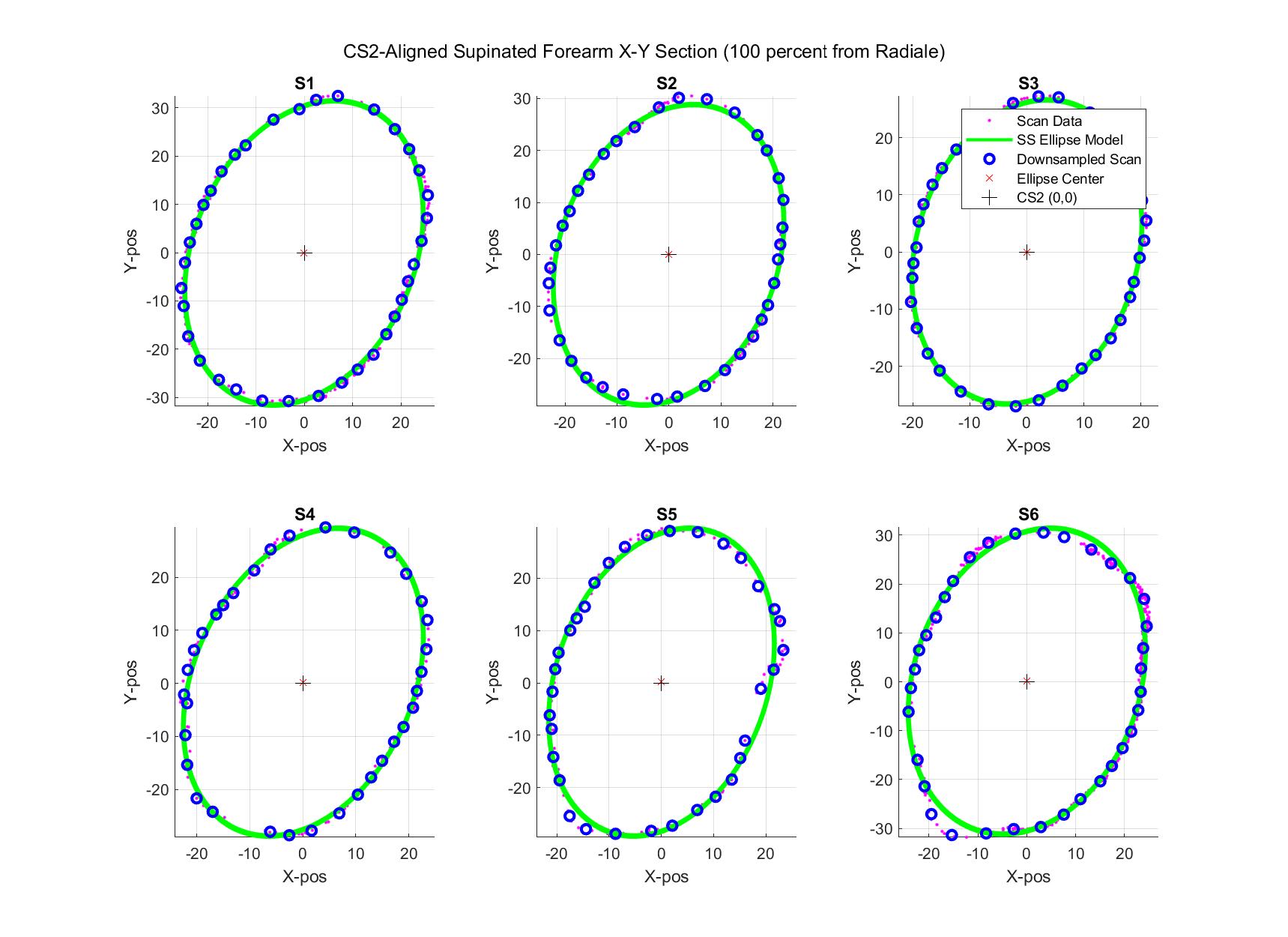

Supplement: Supplementary file 1 [file Data_Sheet_1.ZIP › SF21.17_SS_CS2_Downsampled_Cross-sectional Ellipse-Fit_100%RS_Sup.jpg]

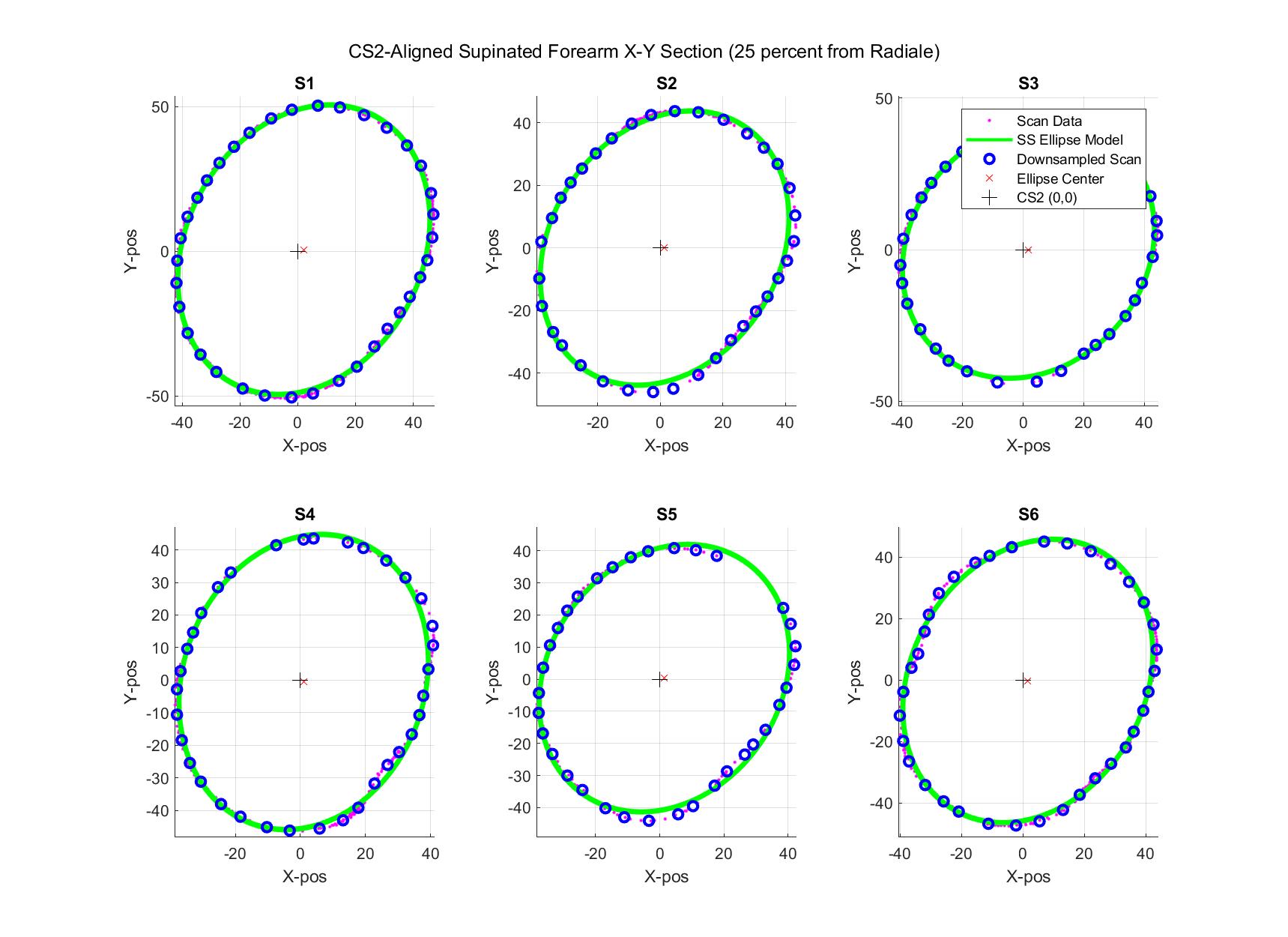

Supplement: Supplementary file 1 [file Data_Sheet_1.ZIP › SF21.2_SS_CS2_Downsampled_Cross-sectional Ellipse-Fit_25%RS_Sup.jpg]

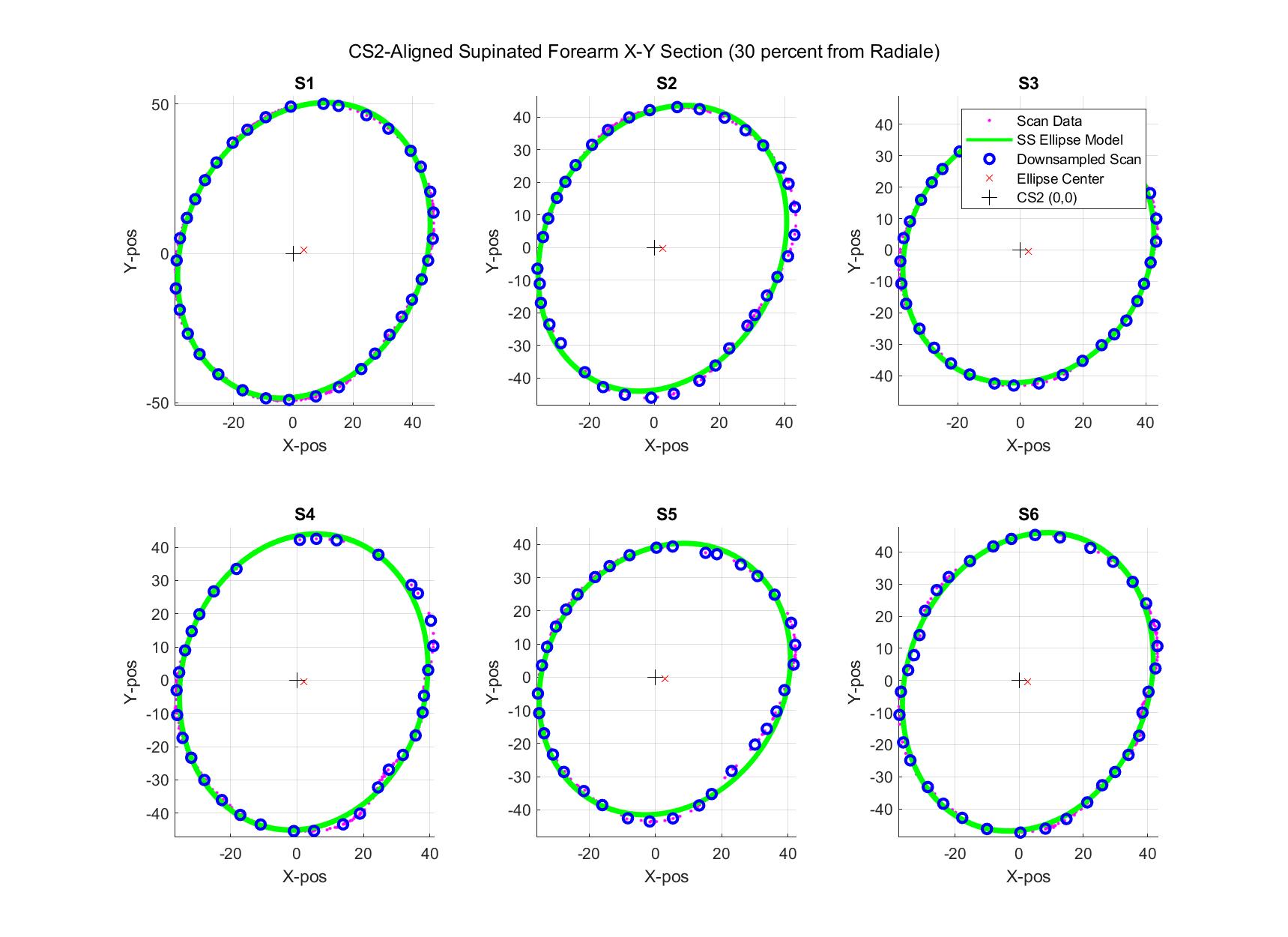

Supplement: Supplementary file 1 [file Data_Sheet_1.ZIP › SF21.3_SS_CS2_Downsampled_Cross-sectional Ellipse-Fit_30%RS_Sup.jpg]

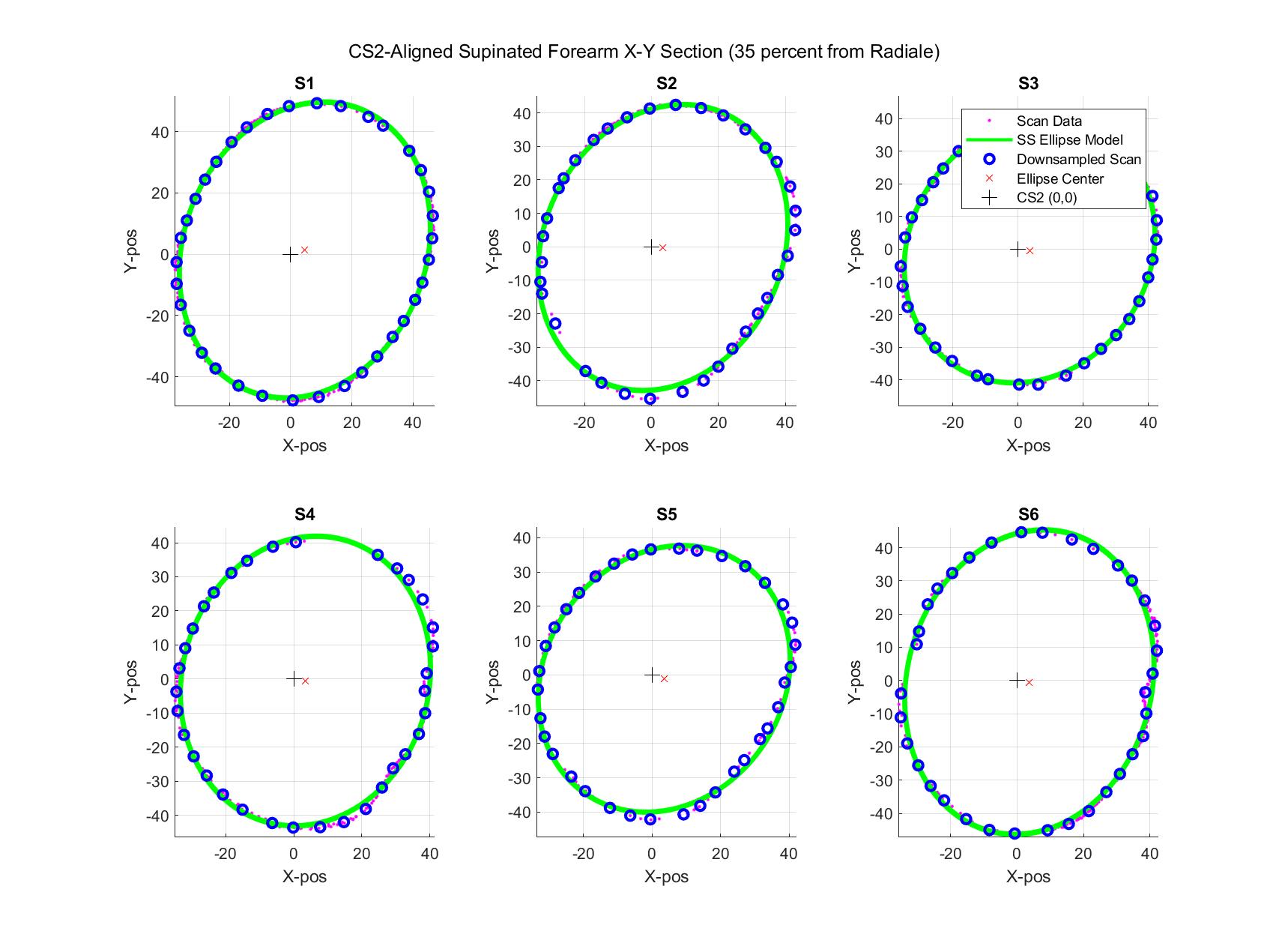

Supplement: Supplementary file 1 [file Data_Sheet_1.ZIP › SF21.4_SS_CS2_Downsampled_Cross-sectional Ellipse-Fit_35%RS_Sup.jpg]

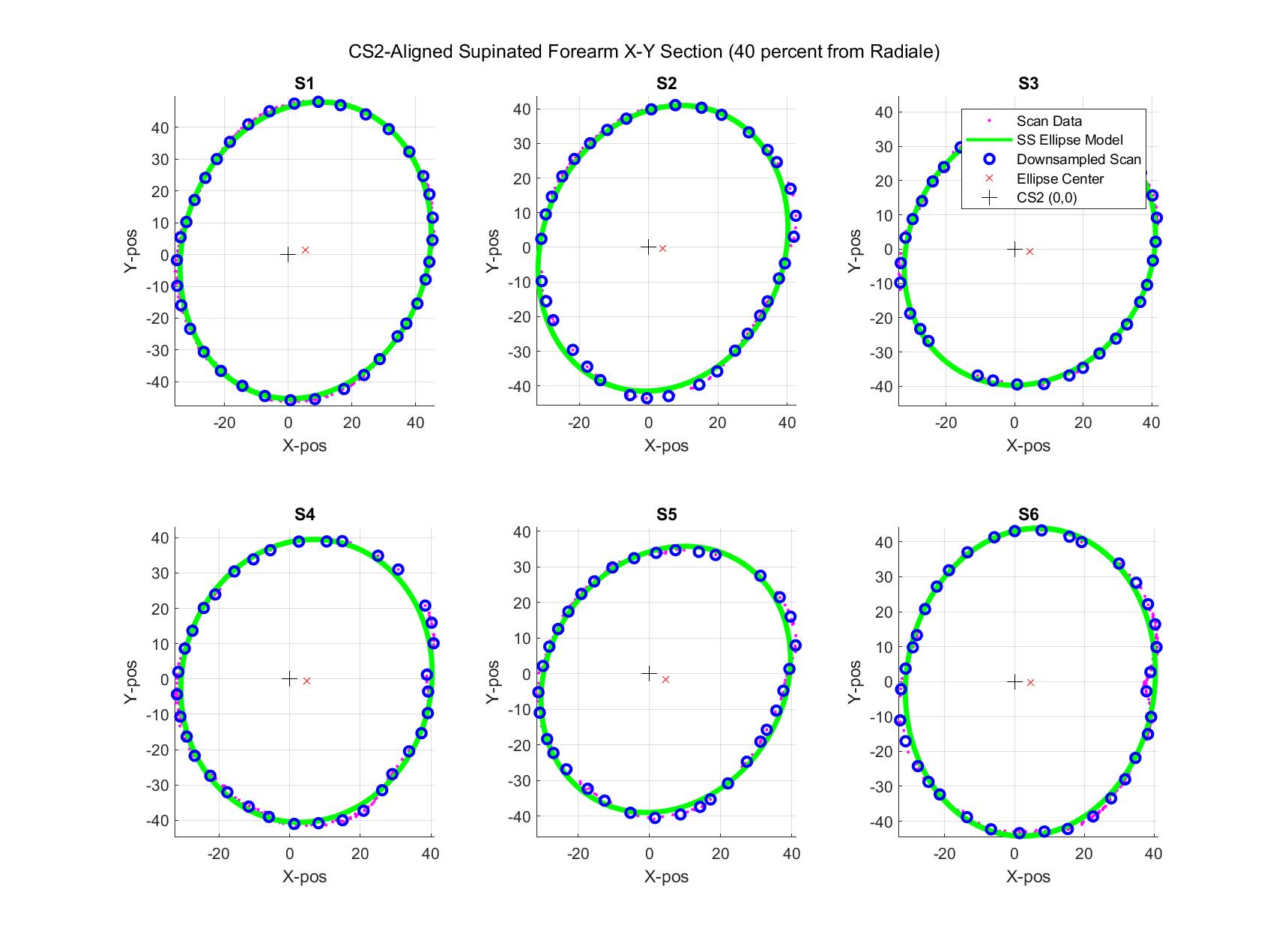

Supplement: Supplementary file 1 [file Data_Sheet_1.ZIP › SF21.5_SS_CS2_Downsampled_Cross-sectional Ellipse-Fit_40%RS_Sup.jpg]

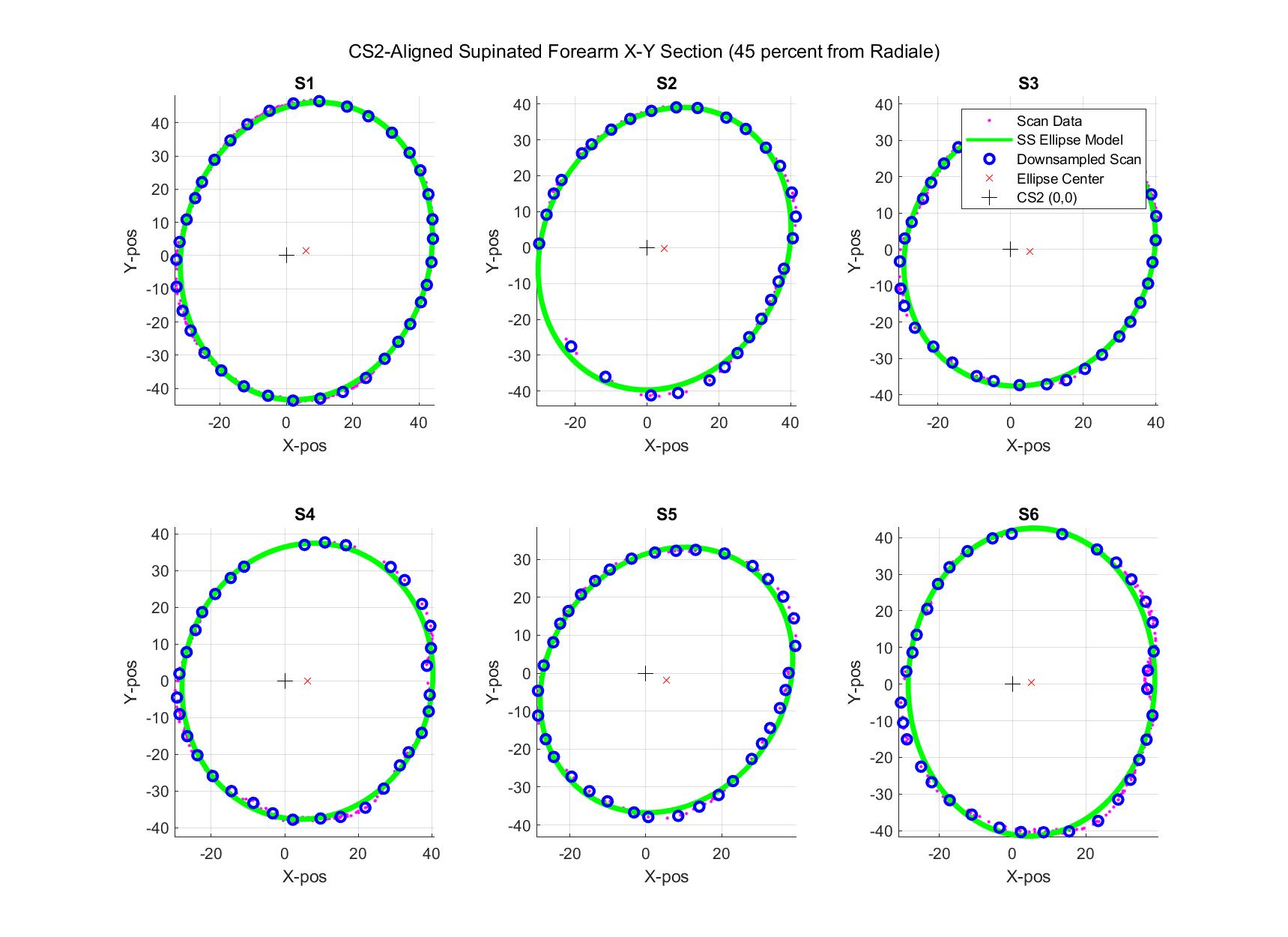

Supplement: Supplementary file 1 [file Data_Sheet_1.ZIP › SF21.6_SS_CS2_Downsampled_Cross-sectional Ellipse-Fit_45%RS_Sup.jpg]

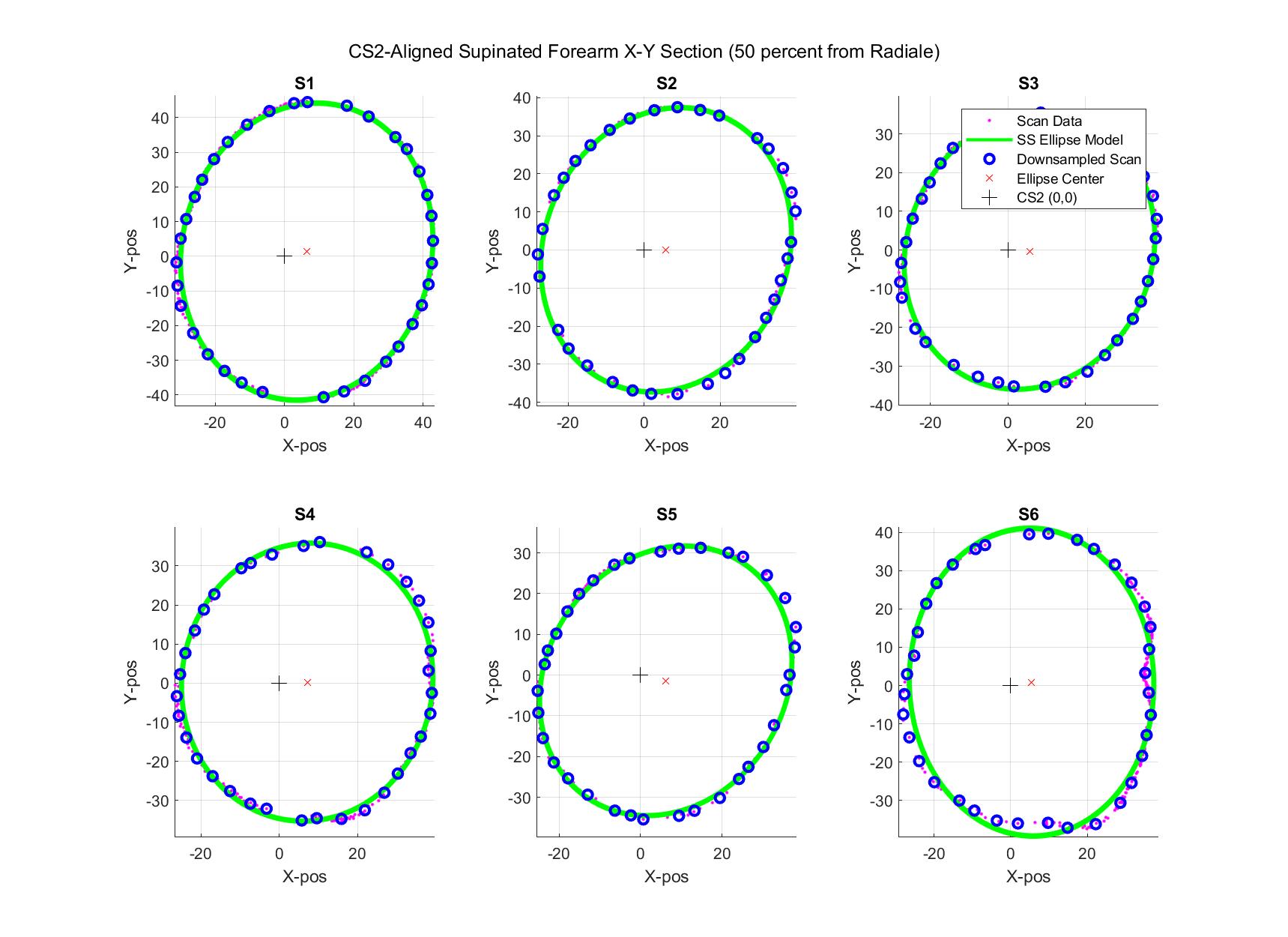

Supplement: Supplementary file 1 [file Data_Sheet_1.ZIP › SF21.7_SS_CS2_Downsampled_Cross-sectional Ellipse-Fit_50%RS_Sup.jpg]

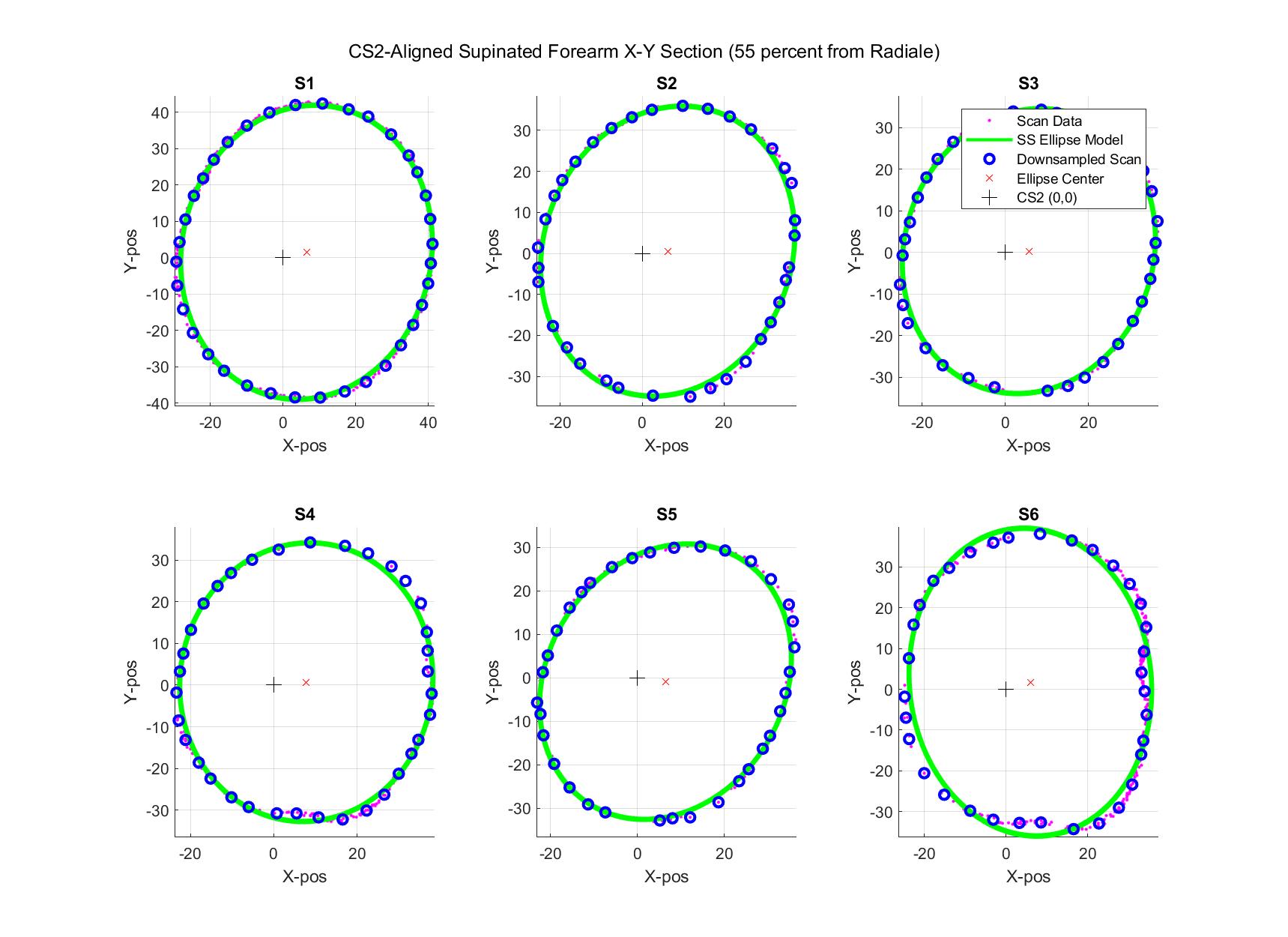

Supplement: Supplementary file 1 [file Data_Sheet_1.ZIP › SF21.8_SS_CS2_Downsampled_Cross-sectional Ellipse-Fit_55%RS_Sup.jpg]

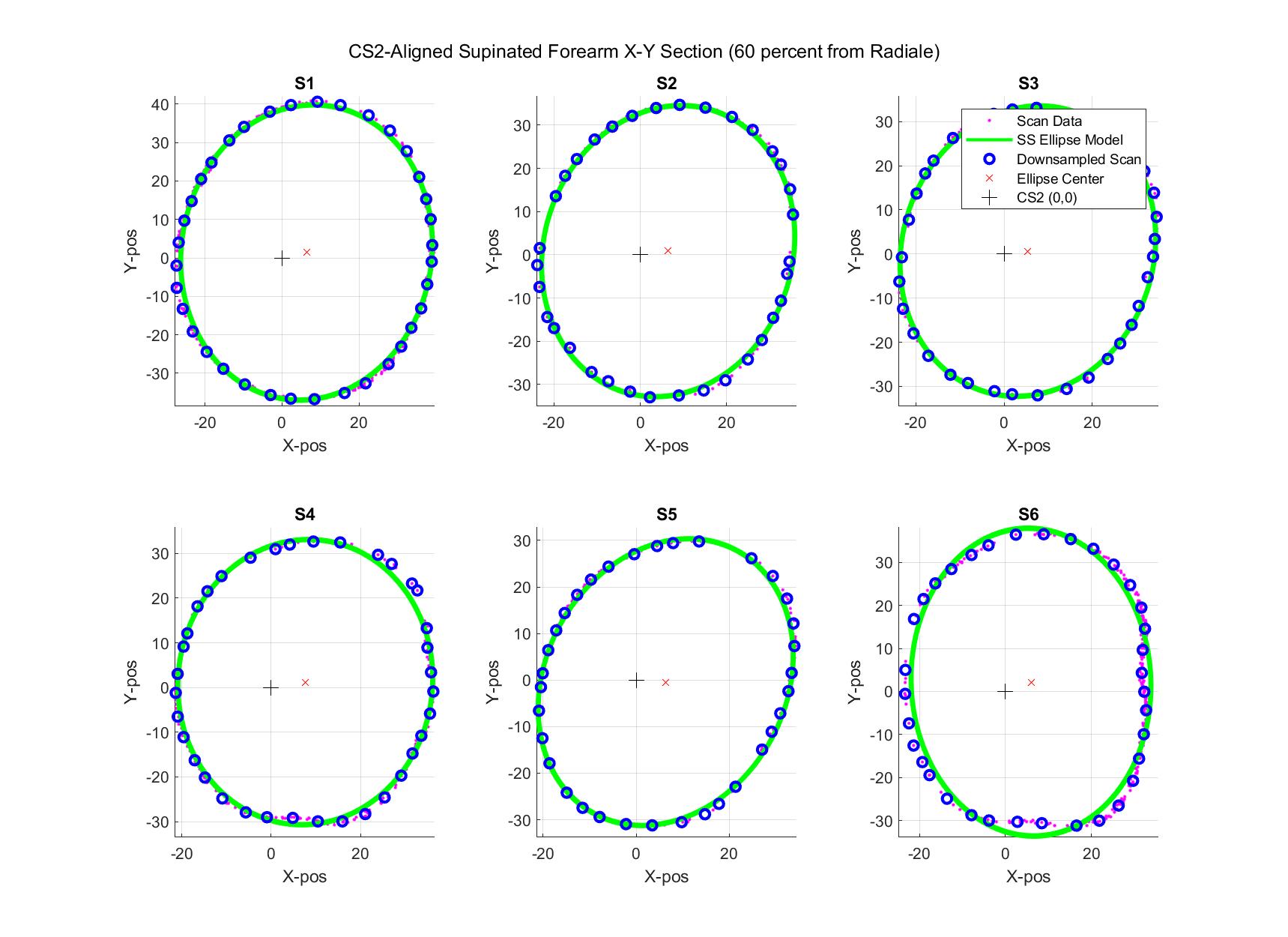

Supplement: Supplementary file 1 [file Data_Sheet_1.ZIP › SF21.9_SS_CS2_Downsampled_Cross-sectional Ellipse-Fit_60%RS_Sup.jpg]

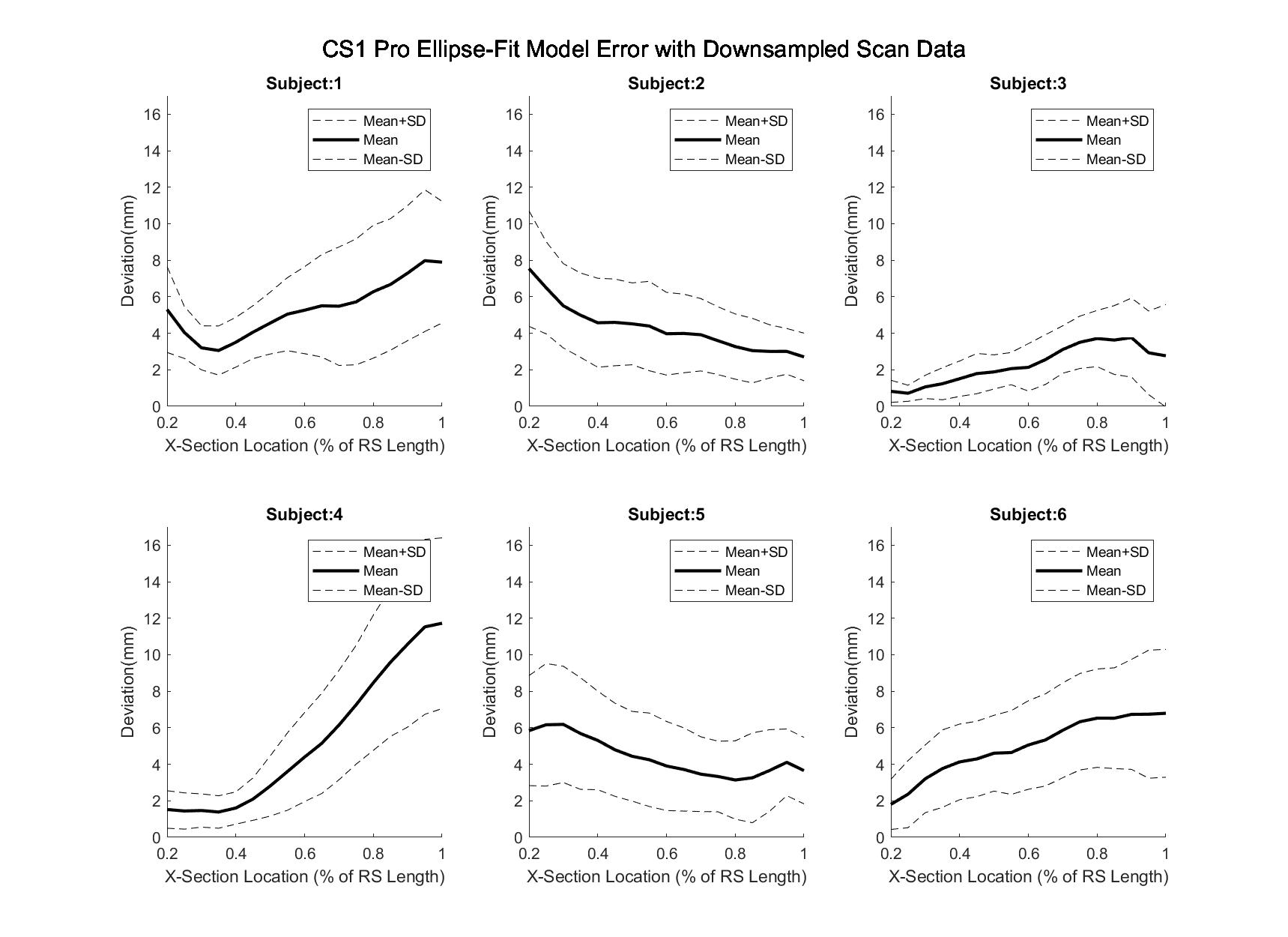

Supplement: Supplementary file 1 [file Data_Sheet_1.ZIP › SF22_CS1 Pro Model Error with Downsampled Scan Data.jpg]

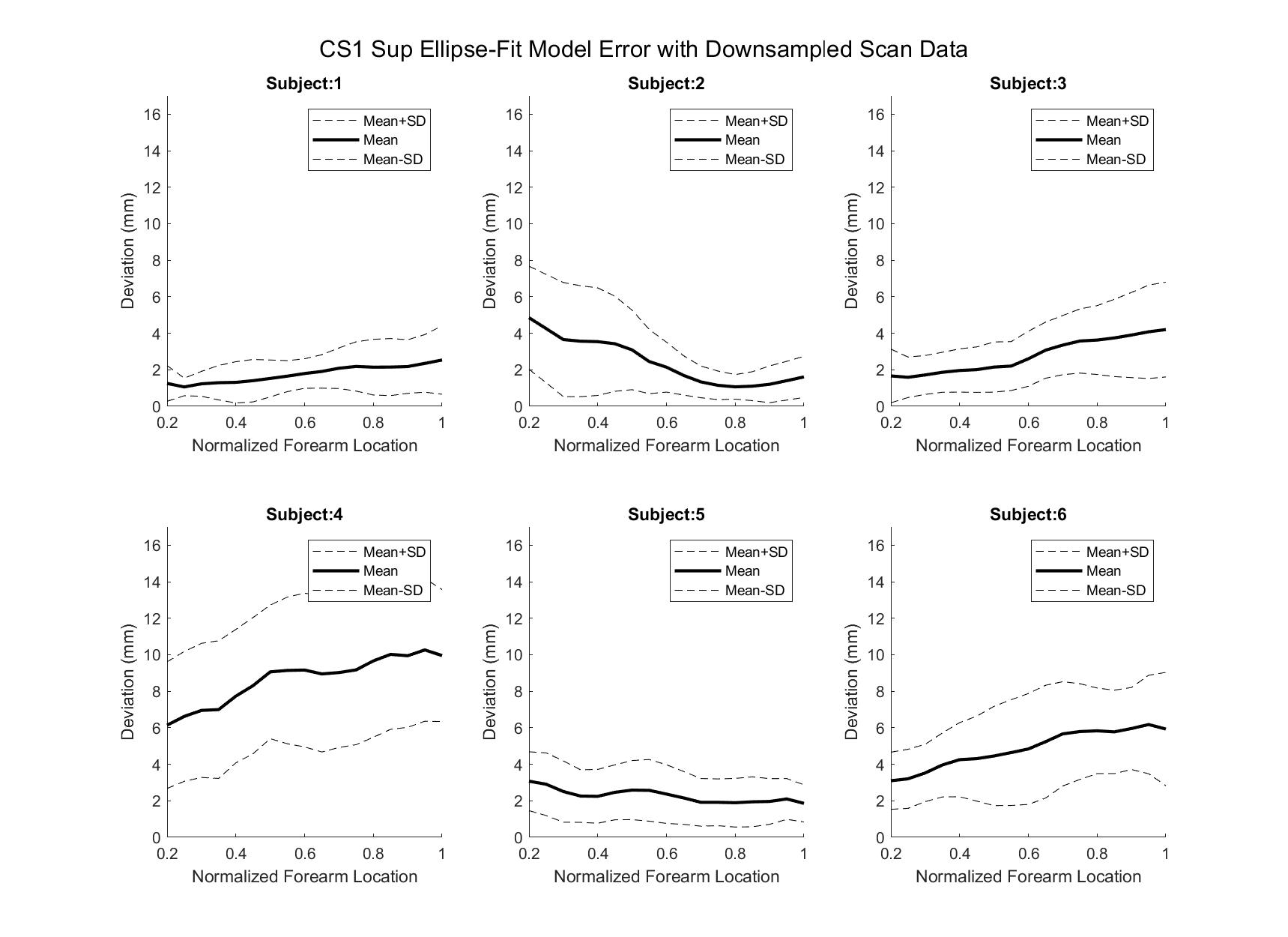

Supplement: Supplementary file 1 [file Data_Sheet_1.ZIP › SF23_CS1 Sup Model Error with Downsampled Scan Data.jpg]

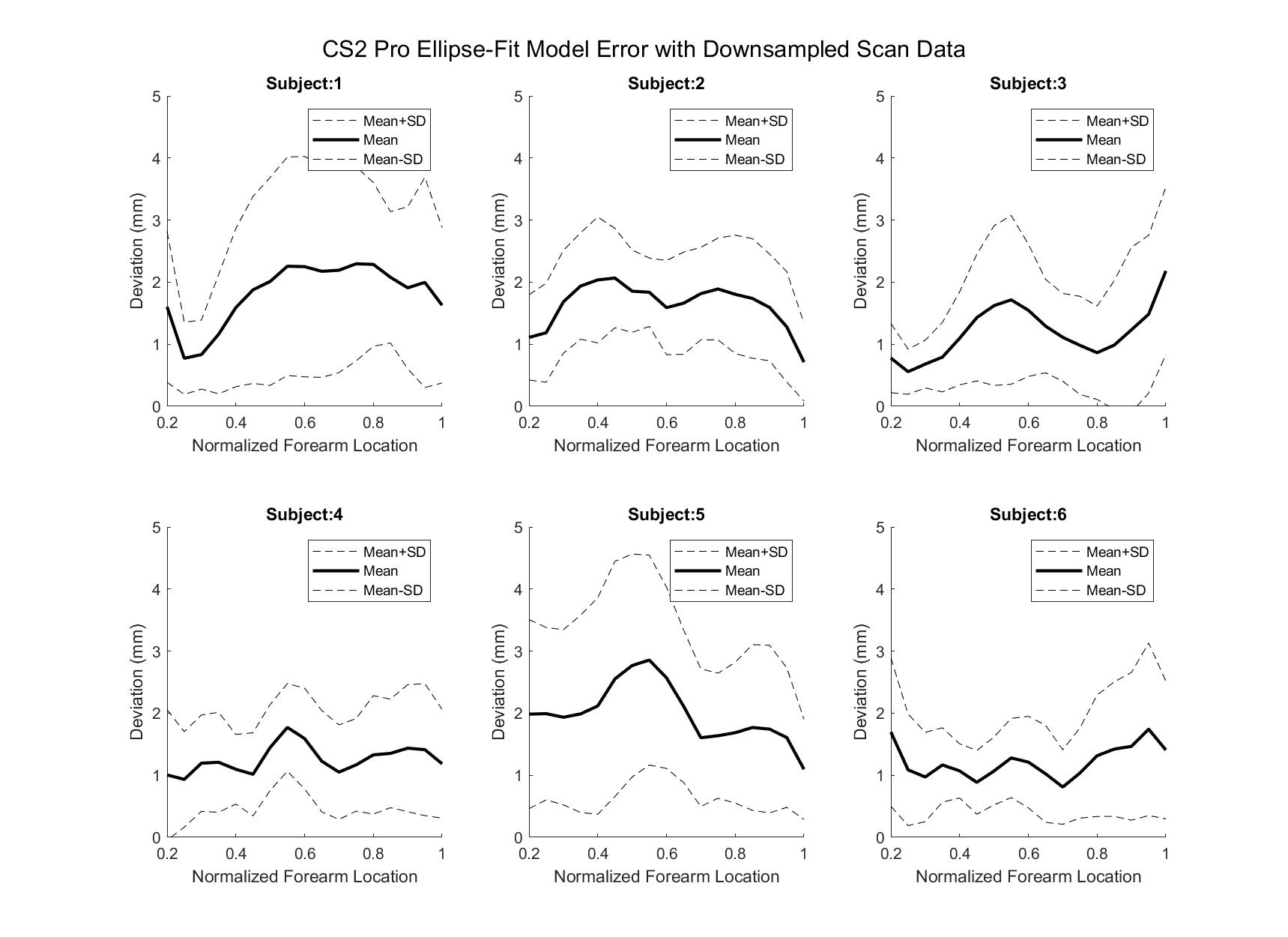

Supplement: Supplementary file 1 [file Data_Sheet_1.ZIP › SF24_CS2 Pro Model Error with Downsampled Scan Data.jpg]

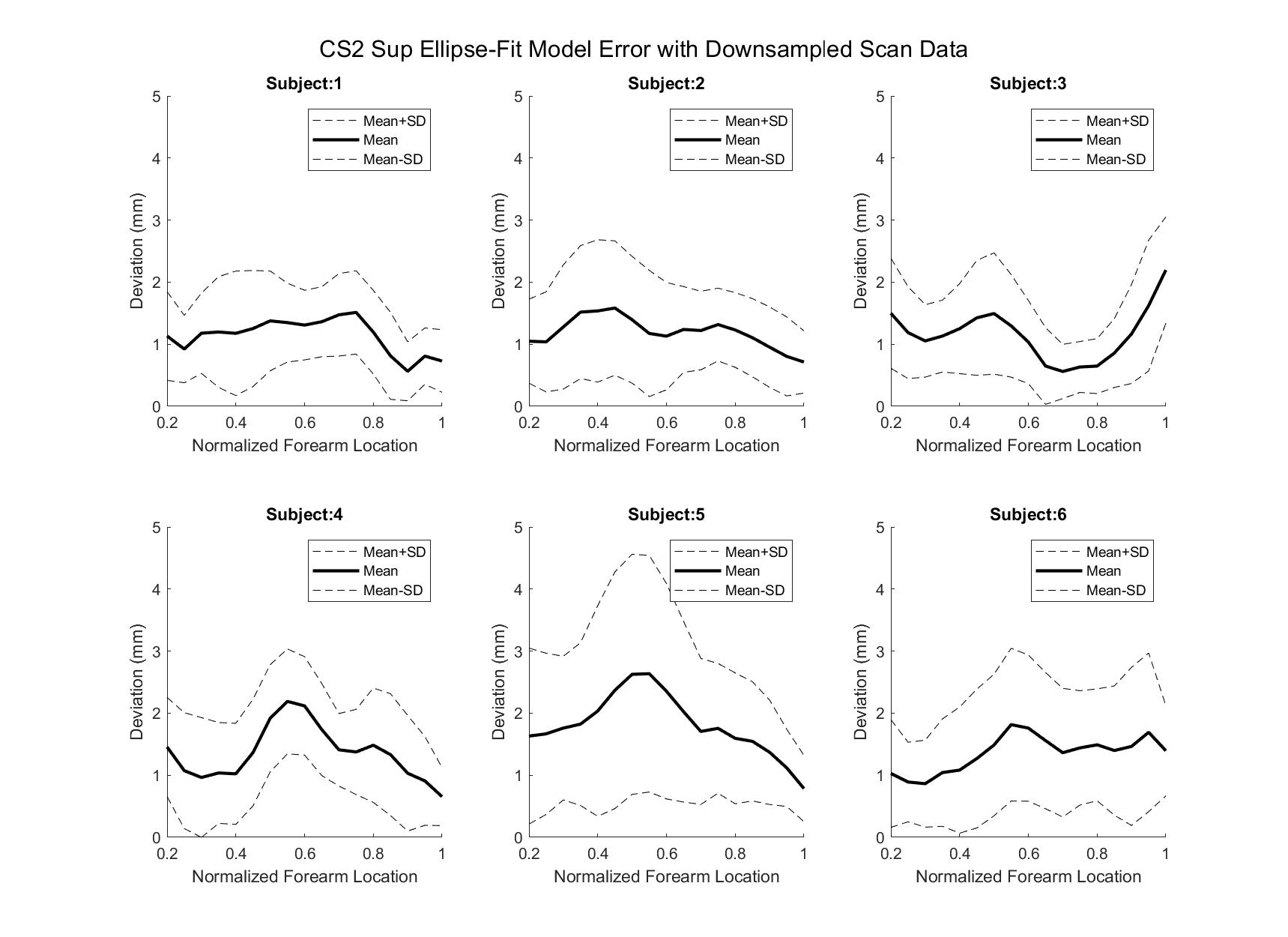

Supplement: Supplementary file 1 [file Data_Sheet_1.ZIP › SF25_CS2 Sup Model Error with Downsampled Scan Data.jpg]

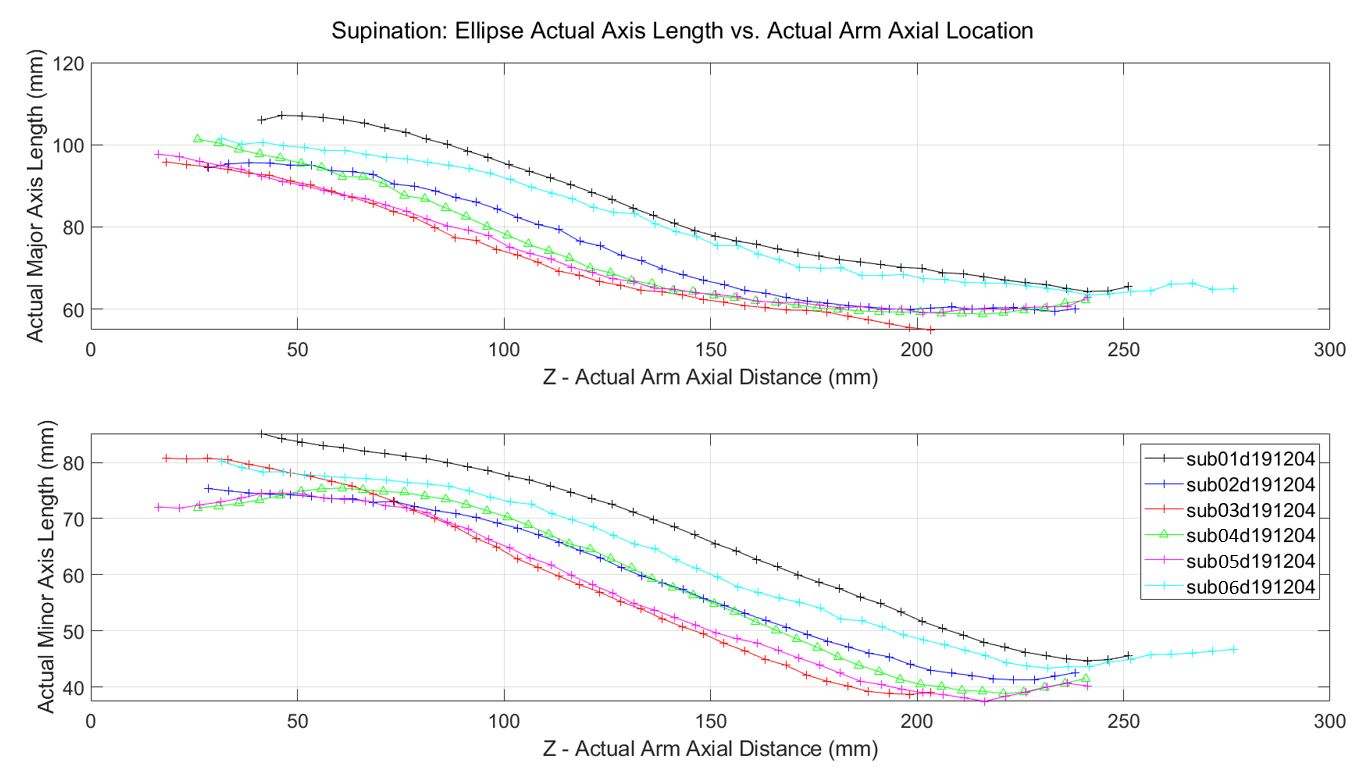

Supplement: Supplementary file 1 [file Data_Sheet_1.ZIP › SF3.1_Raw_AxisLengths_ALL_Sup.JPG]

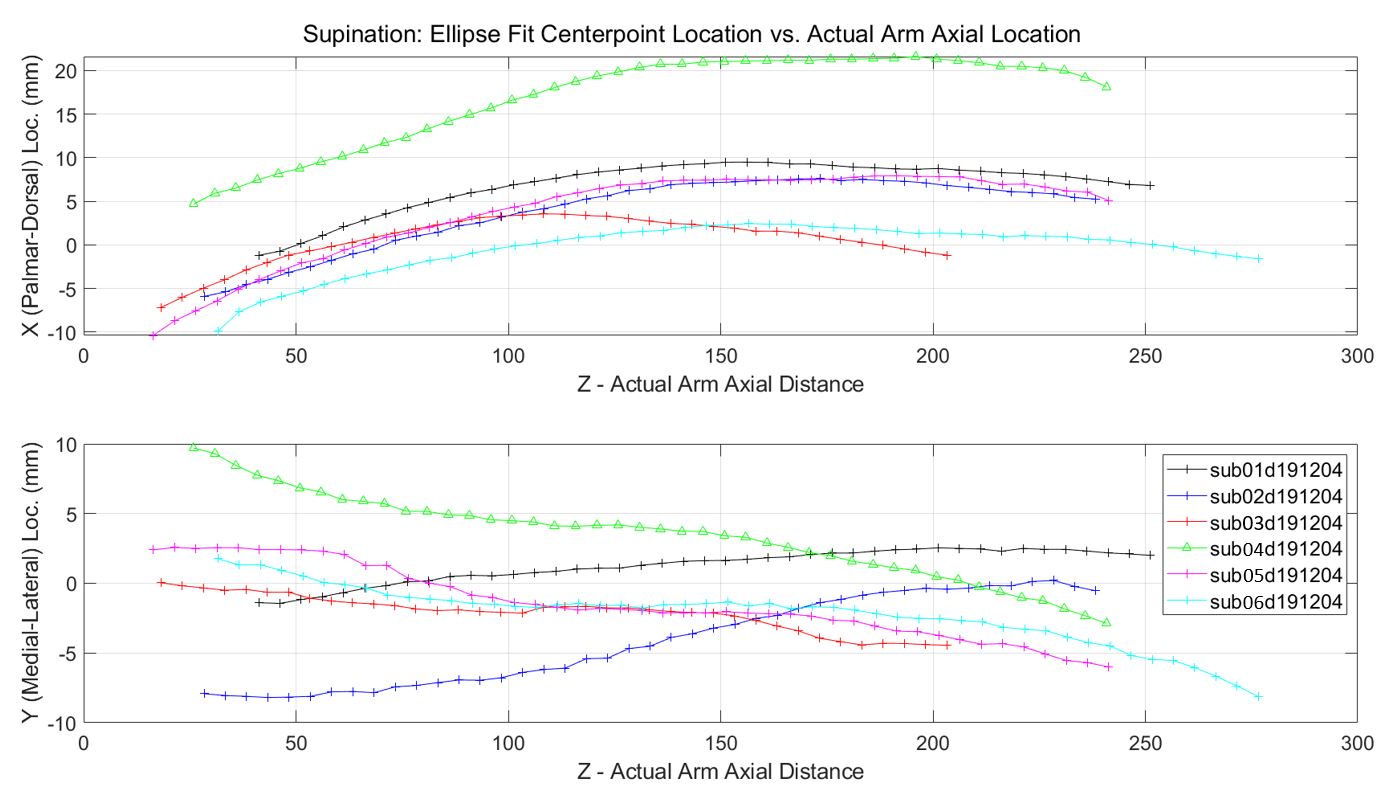

Supplement: Supplementary file 1 [file Data_Sheet_1.ZIP › SF3.2_Raw_CenterLocation_ALL_Sup.JPG]

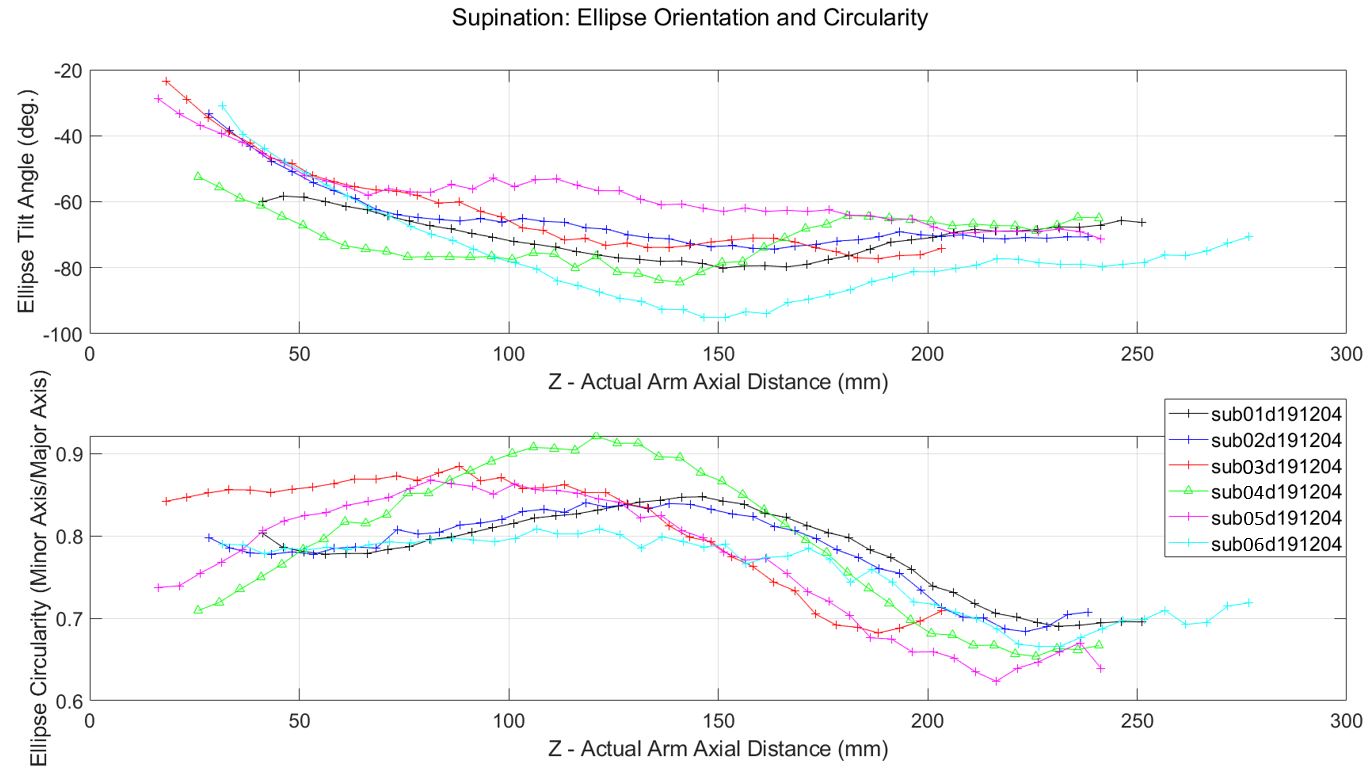

Supplement: Supplementary file 1 [file Data_Sheet_1.ZIP › SF3.3_Raw_TiltAndCircularity_ALL_Sup.JPG]

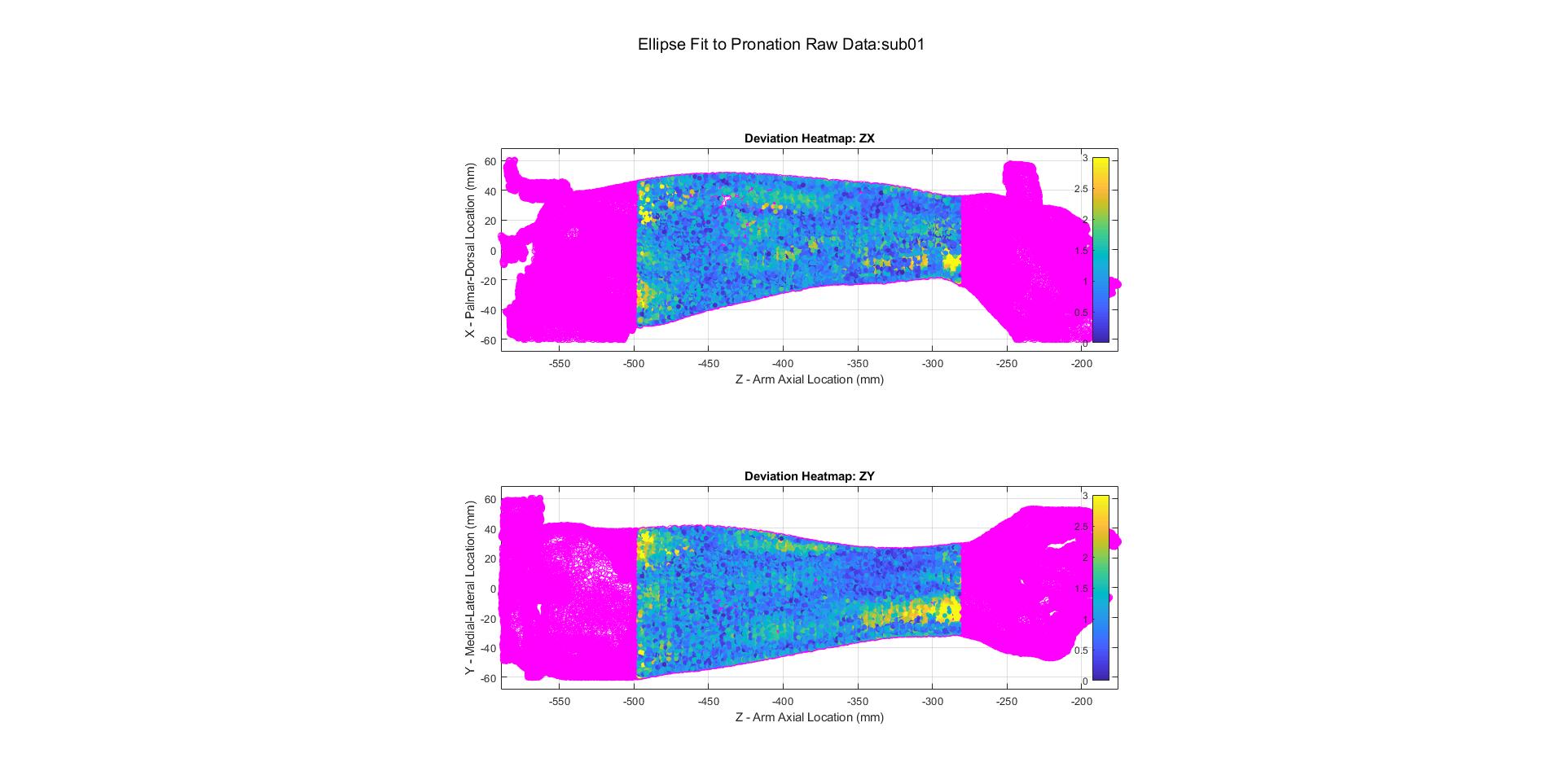

Supplement: Supplementary file 1 [file Data_Sheet_1.ZIP › SF4.1_Figure7_sub01_pro.jpg]

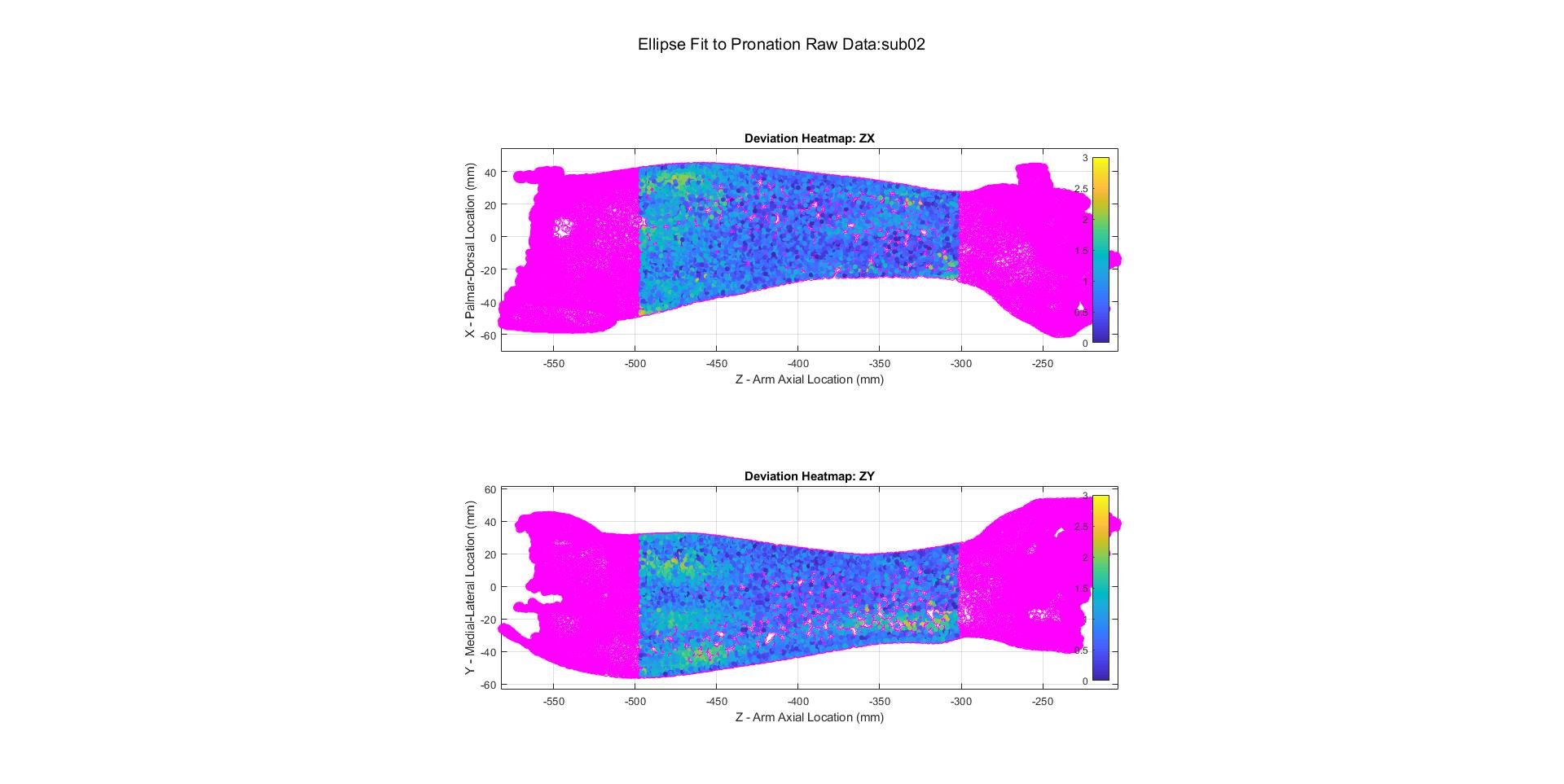

Supplement: Supplementary file 1 [file Data_Sheet_1.ZIP › SF4.2_Figure7_sub02_pro.jpg]

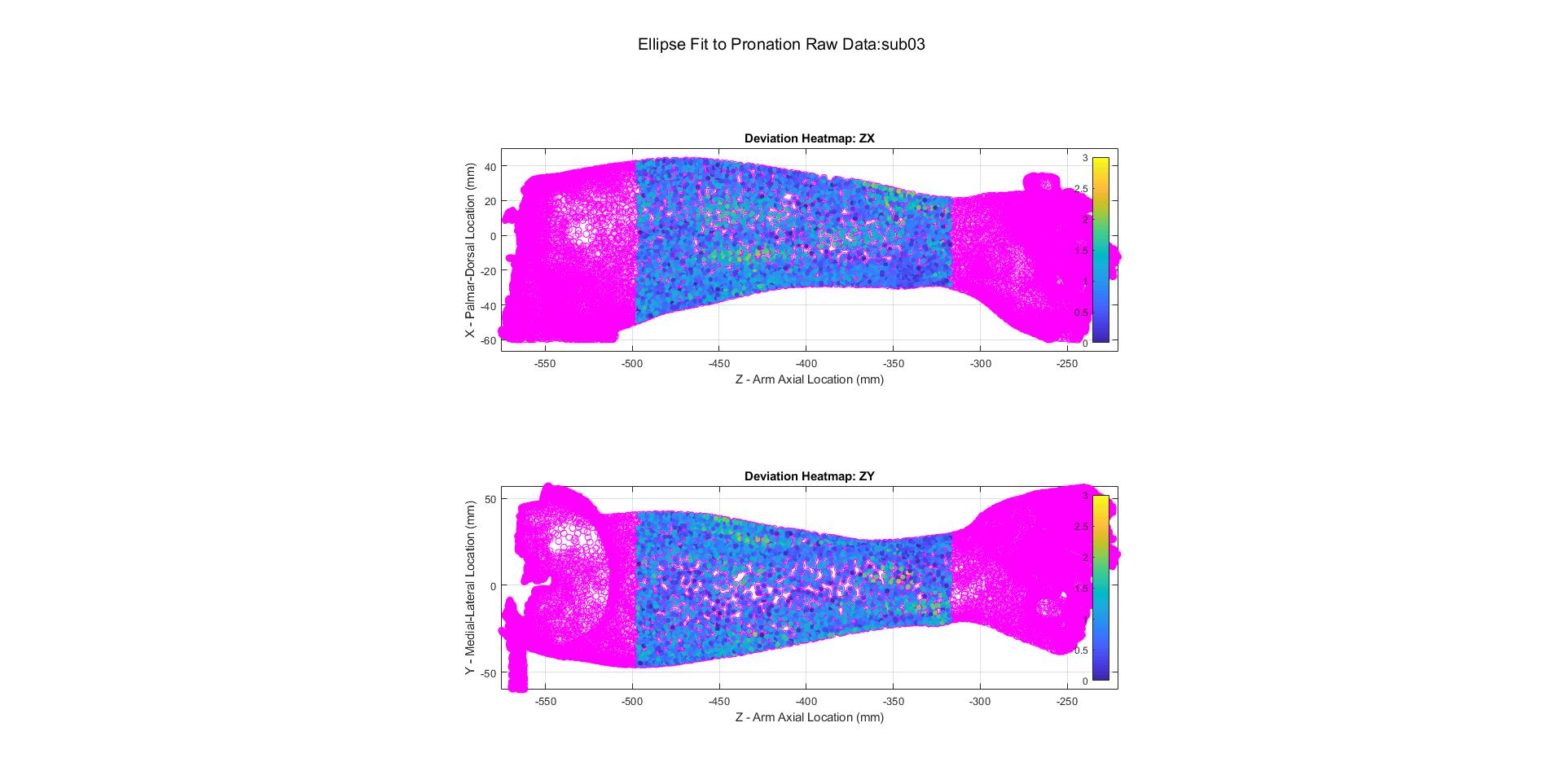

Supplement: Supplementary file 1 [file Data_Sheet_1.ZIP › SF4.3_Figure7_sub03_pro.jpg]

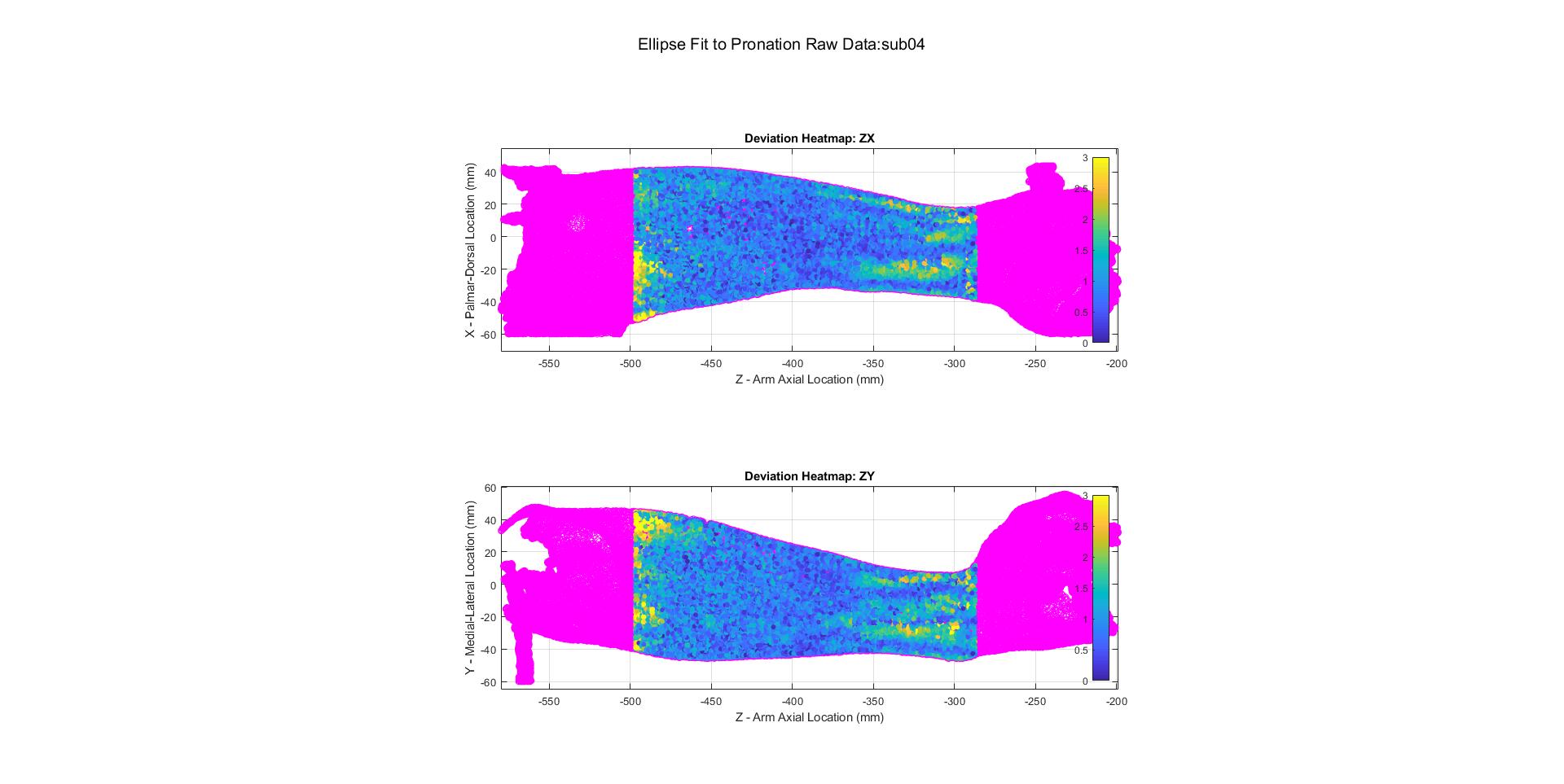

Supplement: Supplementary file 1 [file Data_Sheet_1.ZIP › SF4.4_Figure7_sub04_pro.jpg]

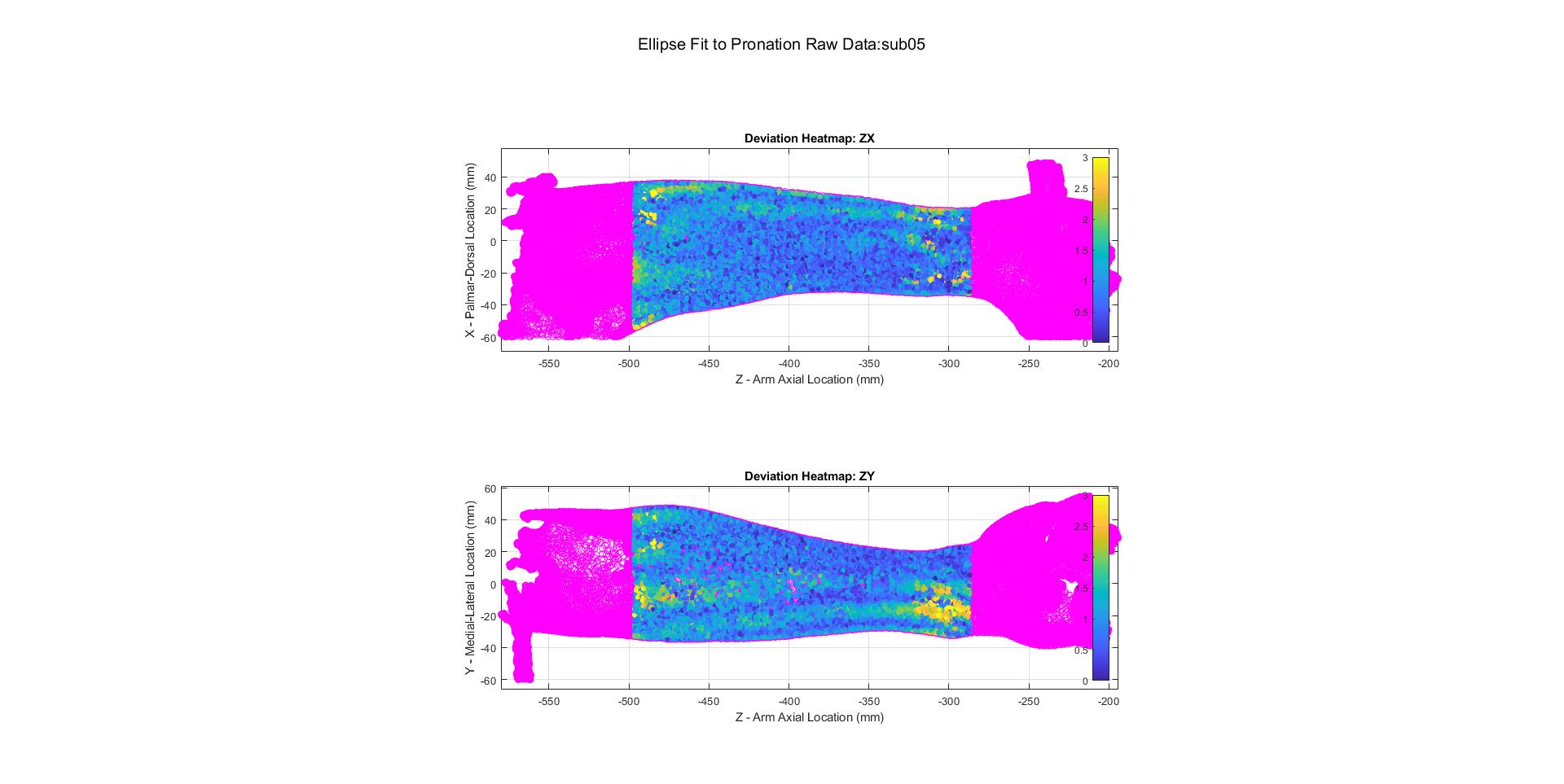

Supplement: Supplementary file 1 [file Data_Sheet_1.ZIP › SF4.5_Figure7_sub05_pro.jpg]

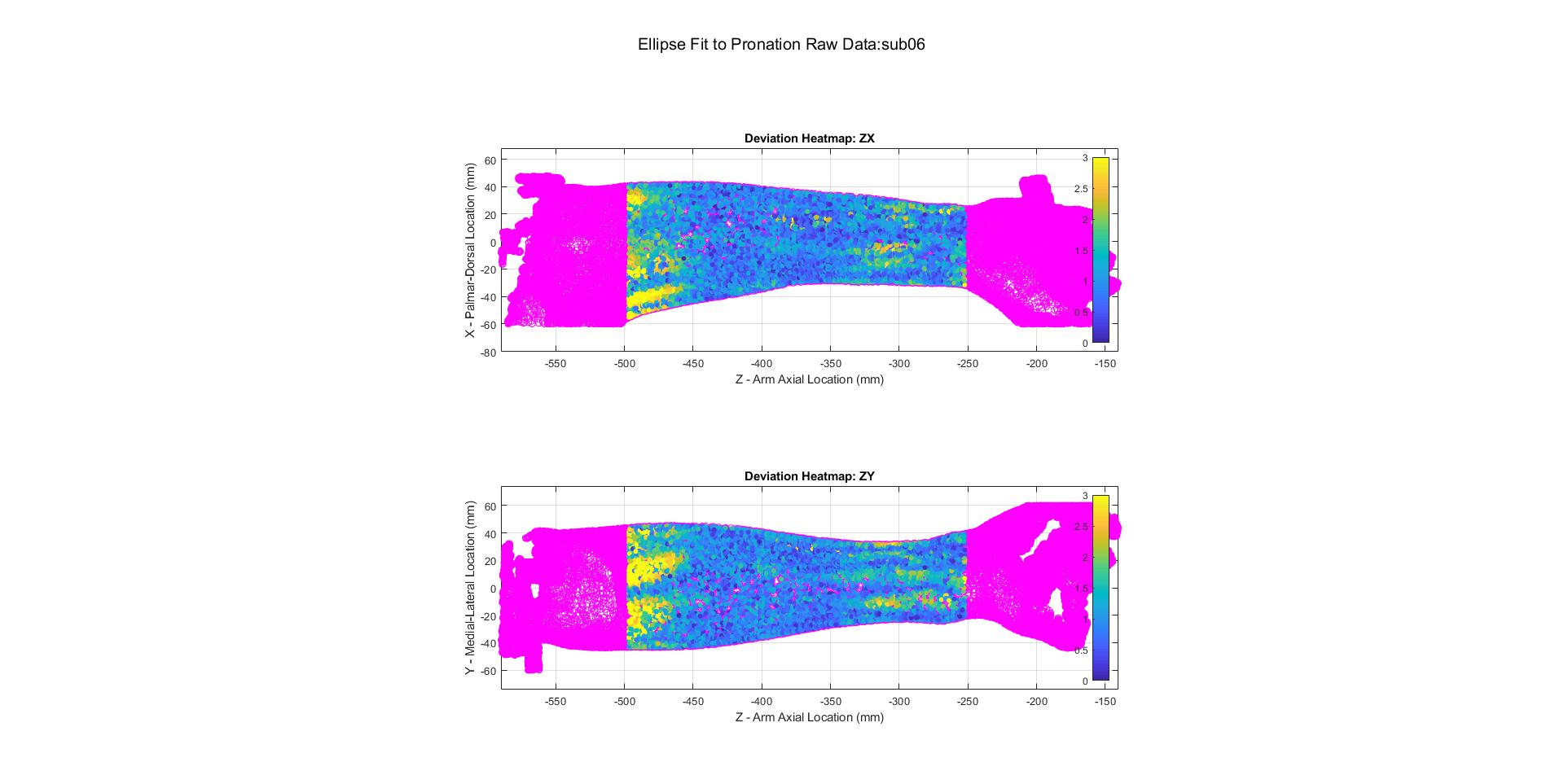

Supplement: Supplementary file 1 [file Data_Sheet_1.ZIP › SF4.6_Figure7_sub06_pro.jpg]

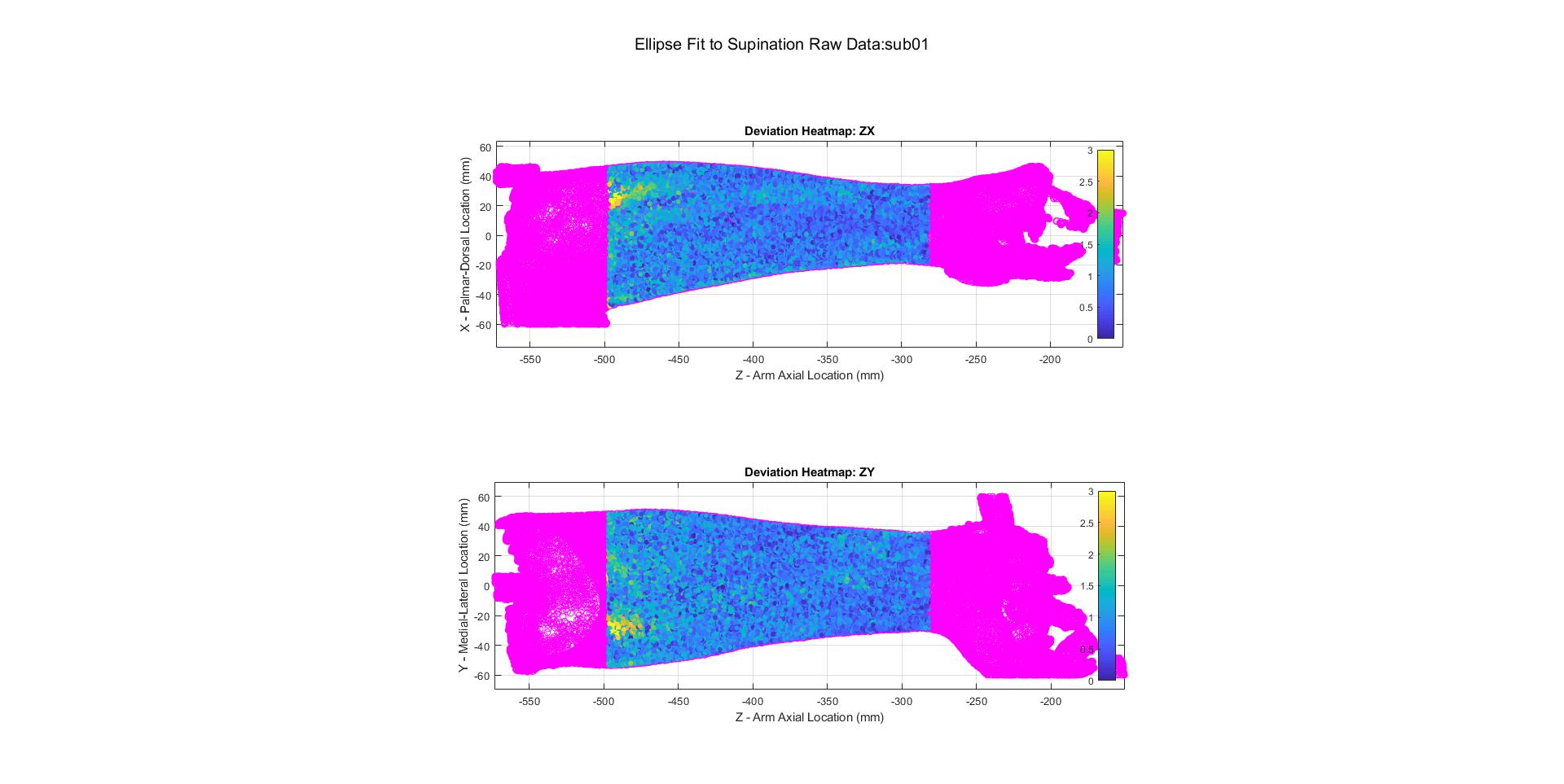

Supplement: Supplementary file 1 [file Data_Sheet_1.ZIP › SF5.1_Figure7_sub01_sup.jpg]

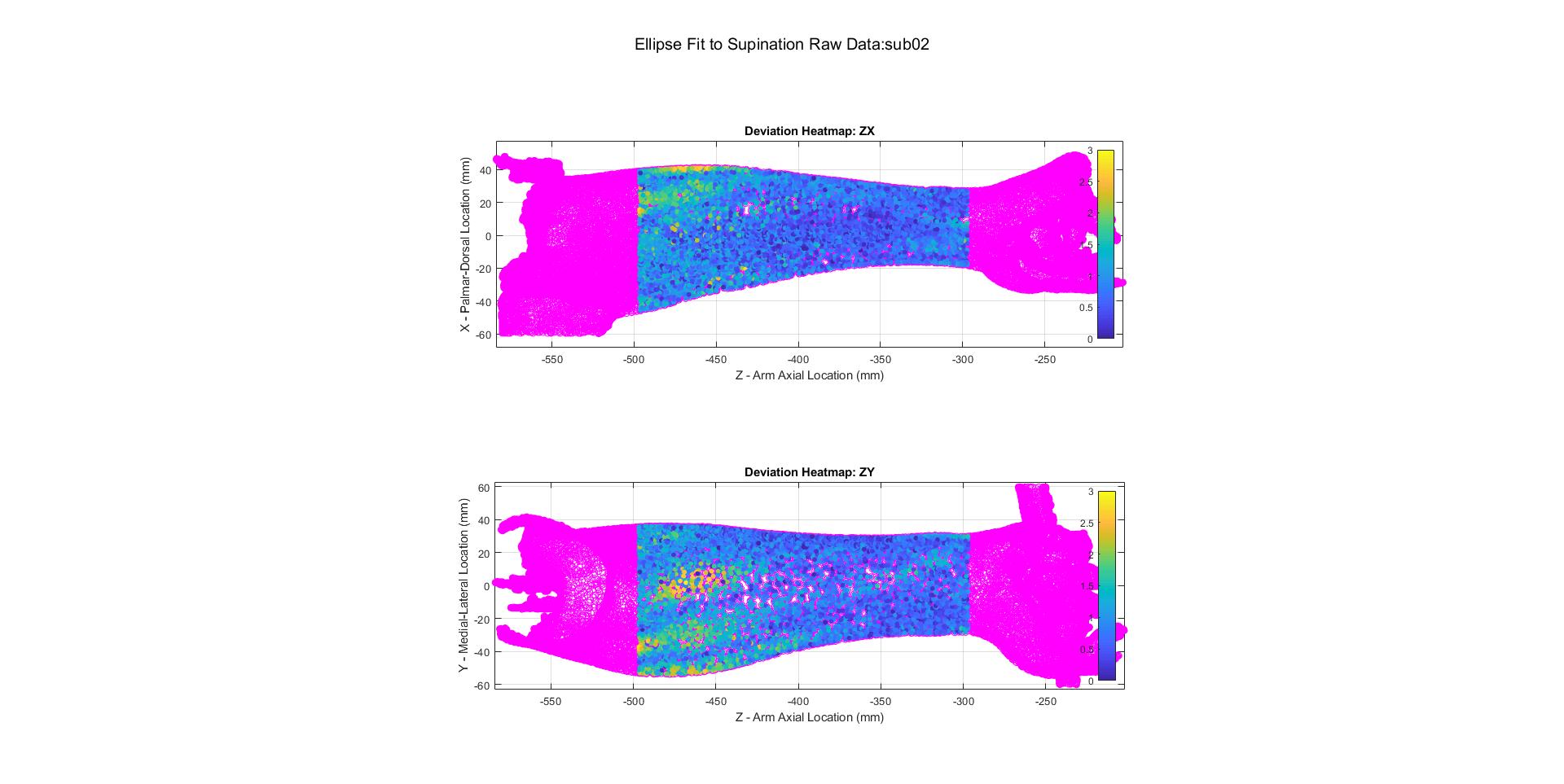

Supplement: Supplementary file 1 [file Data_Sheet_1.ZIP › SF5.2_Figure7_sub02_sup.jpg]
